# During bacteremia, *Pseudomonas aeruginosa* PAO1 adapts by altering the expression of numerous virulence genes including those involved in quorum sensing

**DOI:** 10.1371/journal.pone.0240351

**Published:** 2020-10-15

**Authors:** Kellsie L. Beasley, Shane A. Cristy, Moamen M. Elmassry, Nyaradzo Dzvova, Jane A. Colmer-Hamood, Abdul N. Hamood

**Affiliations:** 1 Department of Immunology and Molecular Microbiology, Texas Tech University Health Sciences Center, Lubbock, Texas, Untied States of America; 2 Honors College, Texas Tech University, Lubbock, Texas, Untied States of America; 3 Department of Biological Sciences, Texas Tech University, Lubbock, Texas, Untied States of America; 4 Department of Medical Education, Texas Tech University Health Sciences Center, Lubbock, Texas, Untied States of America; 5 Department of Surgery, Texas Tech University Health Sciences Center, Lubbock, Texas, Untied States of America; East Carolina University Brody School of Medicine, UNITED STATES

## Abstract

*Pseudomonas aeruginosa* is a Gram-negative opportunistic pathogen that produces numerous virulence factors and causes serious infections in trauma patients and patients with severe burns. We previously showed that the growth of *P*. *aeruginosa* in blood from severely burned or trauma patients altered the expression of numerous genes. However, the specific influence of whole blood from healthy volunteers on *P*. *aeruginosa* gene expression is not known. Transcriptome analysis of *P*. *aeruginosa* grown for 4 h in blood from healthy volunteers compared to that when grown in laboratory medium revealed that the expression of 1085 genes was significantly altered. Quorum sensing (QS), QS-related, and pyochelin synthesis genes were downregulated, while genes of the type III secretion system and those for pyoverdine synthesis were upregulated. The observed effect on the QS and QS-related genes was shown to reside within serum fraction: growth of PAO1 in the presence of 10% human serum from healthy volunteers significantly reduced the expression of QS and QS-regulated genes at 2 and 4 h of growth but significantly enhanced their expression at 8 h. Additionally, the production of QS-regulated virulence factors, including LasA and pyocyanin, was also influenced by the presence of human serum. Serum fractionation experiments revealed that part of the observed effect resides within the serum fraction containing <10-kDa proteins. Growth in serum reduced the production of many PAO1 outer membrane proteins but enhanced the production of others including OprF, a protein previously shown to play a role in the regulation of QS gene expression. These results suggest that factor(s) within human serum: 1) impact *P*. *aeruginosa* pathogenesis by influencing the expression of different genes; 2) differentially regulate the expression of QS and QS-related genes in a growth phase- or time-dependent mechanism; and 3) manipulate the production of *P*. *aeruginosa* outer membrane proteins.

## Introduction

*Pseudomonas aeruginosa* is a Gram-negative opportunistic pathogen that causes serious infections in immunocompromised hosts, including severely burned patients and HIV patients [1–3]. Although it grows ubiquitously in the environment and transiently colonizes the surface of the host’s body, *P*. *aeruginosa* colonizes nearly any site where there is a break in the host’s defenses (such as a surgical wound or burn infection) [1,4]. Once established within the wound environment, *P*. *aeruginosa* then translocates into the bloodstream, leading to sepsis and septic shock [5]. The ability to cause sepsis, combined with its increasing resistance to commonly used antibiotics and the possession of numerous virulence factors, renders *P*. *aeruginosa* a particularly difficult pathogen to treat in clinical settings [6]. *P*. *aeruginosa* is notorious for its ability to survive on different surfaces, especially medical devices such as indwelling urinary catheters and endotracheal tubes [7,8]. Therefore, patients hospitalized for prolonged periods are particularly susceptible to *P*. *aeruginosa* infection. *P*. *aeruginosa* is also one of the leading pathogens responsible for secondary infection in cystic fibrosis patients [9]. Although *P*. *aeruginosa* pathogenesis at different infection sites has been extensively analyzed, little is known regarding the pathogenesis of *P*. *aeruginosa* upon its entry into the bloodstream.

To survive within different host niches and to combat host defenses, *P*. *aeruginosa* adjusts the production of numerous extracellular and cell-associated virulence factors [10–15]. The production of different virulence factors is coordinated by the cell density-dependent signaling system known as quorum sensing (QS) [16,17]. This signaling system depends on the production of small diffusible *N*-acylhomoserine lactone (AHL) signaling molecules known as autoinducers [18]. *P*. *aeruginosa* possesses two well-characterized AHL-based QS systems, *las* and *rhl*. Each system utilizes a transcriptional activator (LasR, RhlR) and an autoinducer synthase (LasI, RhlI) [19]. The *las* autoinducer *N*-(3-oxododecanoyl)-L-homoserine lactone (3OC12-HSL) is produced by LasI, whereas RhlI is responsible for the production of *N*-(butyryl)-L-homoserine lactone (C4-HSL), the *rhl* autoinducer [16,20]. Besides *las* and *rhl*, *P*. *aeruginosa* also produces a quinolone-based intercellular signaling molecule (2-heptyl-3-hydroxy-4-quinolone) termed the *Pseudomonas* quinolone signal, or PQS [21]. The PQS autoinducer is synthesized by the *pqsA-E* operon, and binds to and activates the transcriptional regulator PqsR (also known as MvfR) [21,22]. The QS systems function in a hierarchy; experimental evidence suggests that LasR positively regulates the *rhl* and *pqs* branches [19,23–25], RhlR inhibits *pqs* while PqsR stimulates *rhl* [25–27], and the LasR-regulated gene *lasB* is induced by PQS autoinducer [17,20,21]. Together, the interconnected QS systems control the production of numerous virulence factors including exotoxin A, proteases and elastases (including LasA and LasB), rhamnolipids (including 3-(hydroxyalkanoyloxy)alkanoic acid, mono-rhamnolipids, and di-rhamnolipids produced by RhlA, RhlB, and RhlC, respectfully), and pyocyanin [12–15,28].

Another QS-related *P*. *aeruginosa* virulence factor is pyocyanin. Pyocyanin is a redox-active pigment that, as a zwitterion, can easily penetrate host biological membranes, where it generates reactive oxygen species that disrupt the electron transport chain of host tissues [29,30]. This disruption leads to the accumulation of toxic superoxides and cell injury [29,30]. Enzymes involved in pyocyanin production are encoded by two nearly identical operons, *phzA1-G1* and *phzA2-G2* [31]. Additional genes, *phzM*, *phzH*, and *phzS*, are involved in the processing and modification of pyocyanin and other related phenazine molecules [15,31]. Very little is known about the specific regulation of *phzA1-G1* and *pzhA2-G2*. However, experimental evidence and sequence analyses strongly suggest that they are regulated by the QS systems, including *las*, *rhl*, and *pqs* [32–35].

We previously investigated the effect of severe burn-induced and other trauma-induced changes in blood on the pathogenesis of *P*. *aeruginosa* during bacteremia [36,37]. Compared with its growth in whole blood from healthy volunteers (WBHVs), the growth of *P*. *aeruginosa* strain PA14 in whole blood from severely burned patients significantly decreased the expression of QS, QS-controlled, and pyoverdine synthesis genes and increased expression of type III secretion system (T3SS) genes [37]. Growth of PA14 in whole blood from trauma patients also downregulated expression of the pyoverdine synthesis genes but only a few T3SS or QS genes were affected; instead, multiple genes of the type VI secretion system were significantly downregulated while genes for malonate utilization and carbohydrate uptake were upregulated [36]. Genes of the malonate operon were also found to be upregulated in the study by Kruczek *et al*. [37]. These results suggest that other conditions associated with *P*. *aeruginosa* bacteremia (e.g., patients undergoing cancer chemotherapy) could alter *P*. *aeruginosa* pathogenesis in a specific manner. To establish a reference point for future studies on *P*. *aeruginosa* pathogenesis during bacteremia, it is essential to determine the influence that whole blood from healthy individuals would have on the expression of different *P*. *aeruginosa* genes. In this study, we addressed this by growing *P*. *aeruginosa* strain PAO1 in either laboratory medium or WBHV and assessing the expression of the PAO1 transcriptome.

## Materials and methods

### Ethics approval and consent to participate

This study was approved by the Texas Tech University Health Sciences Center Institutional Review Board. Written consent was obtained from healthy volunteers (HV) by an individual both trained and approved by the IRB for this particular IRB-approved protocol. A total of 25 mL of whole blood (WB) was collected by venipuncture from each HV, with a total of 3 healthy adult volunteers participating in this study. Blood was collected in three tubes (8.3 mL/tube) containing sodium polyanetholesulfonate as an anticoagulant (BD Vacutainer; Becton, Dickinson and Company, Franklin Lakes, NJ). Samples were identified only as HV1, HV2, and HV3.

### Bacterial strains, plasmids, media, and growth conditions

The prototrophic *P*. *aeruginosa* strain PAO1 [38] was used in all experiments. PAO1 cultures were grown overnight in Luria-Bertani broth (LBB) at 37°C with shaking at 200 RPM. Aliquots of the overnight cultures were pelleted by centrifugation at ~15000 × *g* for 1 min, the supernatant was discarded, and the pellet was resuspended in fresh LBB to a final OD_600_ of 0.02–0.03 (3.0 × 10^7^ to 5.0 × 10^7^ CFU/mL). For experiments involving WBHV, separate 5-mL aliquots were inoculated to a similar density (as determined by CFU/mL) with freshly diluted PAO1. All blood cultures were grown at 37°C under shaking conditions to 1.0 × 10^9^ CFU/mL (4 h of incubation post-inoculation).

For experiments involving pooled normal human serum (PHS) (MilliporeSigma, St. Louis, MO), cultures were grown in either LBB or LBB supplemented with 10% human serum (LBBS) under conditions described above for LBB, and samples were collected at specific time points within the growth cycle. For experiments involving human serum albumin (HSA), 17.5–25 mg of lyophilized HSA (MilliporeSigma, St. Louis, MO) was added to 5 mL of LBB for a final concentration of 3.5–5.0 mg/mL (LBBA). This concentration matches the concentration of HSA found in LBBS; that is, 10% of the concentration found in normal human serum, which is 35–50 mg/mL. Cultures were grown in either LBB or LBBA as described above for LBB; samples were collected at specific time points within the growth cycle.

For experiments involving PAO1 carrying plasmids, antibiotics–carbenicillin at 200 μg/mL (for pMW303) or streptomycin at 300 μg/mL (for pMP190::*pvdD-lacZ*)–were added to LBB, LBBS, or LBBA to maintain the plasmids in PAO1. Both plasmids are described below (β-Galactosidase assays for gene expression).

### RNA isolation

For isolation of RNA for RNA-Seq or qRT-PCR, cultures were harvested at specific time points, from 2 h to 12 h depending on the experiment, within the growth cycle of PAO1. For blood cultures, red and white blood cells were removed by differential centrifugation in lymphocyte separation medium (Lonza, Walkersville, MD) as previously described [37]. PAO1 cells were pelleted by centrifugation at 5,000 RPM for 10 min and resuspended in fresh LBB containing RNAprotect (QIAGEN, Valencia, CA). The supernatant was discarded, and pellets were stored at -80°C. RNA was extracted from these pellets using the RNAeasy Mini Kit (QIAGEN).

### RNA-Seq and qRT-PCR

Genomic DNA was digested from the samples using RNase-free DNase (QIAGEN). Purified RNA was then quantified by NanoDrop spectrophotometer (NanoDrop Technologies, Wilmington, DE). Integrity of the RNA was analyzed using RNA Nano Chip on an Agilent 2100 Bioanalyzer (Agilent, Palo Alto, CA). RNA samples with 1.8–2.2 ratios of absorbance at 260/280 nm were either prepared for RNA-Seq or converted to cDNA using the QuantiTect Reverse Transcription Kit (QIAGEN) for qRT-PCR as previously described [37].

For RNA-Seq, RNA was purified with an rRNA removal kit (Ribo-Zero; Epicentre Biotechnologies, Madison, WI). RNA-Seq libraries were constructed as described previously [37]. The cDNA libraries were constructed from the prepared RNA-Seq libraries, validated, and loaded onto MiSeq v2 300 cycle reagent cartridges for sequencing using a MiSeq Sequencer (Illumina, San Diego, CA). Paired end sequencing was performed to obtain 150 bp reads. Rockhopper 2 [39,40] was used in RNA-Seq data analysis, implementing reference-based transcript assembly with PAO1 as a reference genome as described previously [36]. Briefly, after reads alignment, transcript abundance was normalized by upper quartile normalization and quantified using reads per kilobase of transcript per million mapped reads (RPKM) method. Then, differential gene expression was determined using a negative binomial distribution as the statistical model to compute *P* value in Rockhopper 2. *P* values were corrected (*q* values) for false discovery rate using the Benjamini-Hochberg procedure [41]. Genes were identified as significantly differentially expressed if there was a ≥ twofold change in expression and a false discovery rate correction *q*-value of ≤ 0.05 for each of the three HV samples (S1 Table). The data for the samples have been deposited in the BioProject at NCBI (https://www.ncbi.nlm.nih.gov/bioproject/) under PRJNA287707.

For qRT-PCR, equal amounts of cDNA was mixed with SYBR Green PCR Master Mix (Life Technologies, Carlsbad, CA) together with 250 nM of specific primers for each gene examined. Amplification and detection was done using StepOne Plus real-time PCR system (Life Technologies). Each qRT-PCR experiment consisted of three independent biological replicates generated from RNA extraction and cDNA conversion as described above. Additionally, each biological replicate was analyzed in triplicate. Quantity of cDNA in the samples was normalized using the housekeeping 30S ribosomal RNA gene *rpsL*. Analysis of gene expression was done using StepOne Plus software (Life Technologies).

### Assays for staphylolytic activity, pyocyanin, and pyoverdine

For all assays, PAO1 was inoculated in LBB and LBBS or LBBA and grown as described above. For each assay, samples were collected at specific time points throughout the growth cycle and the supernatant fractions and cell pellets were collected by centrifugation at 12,000 RPM for 1 min.

**Staphylolytic activity** of the LasA protease was determined by the ability of PAO1 supernatant fractions to lyse boiled cells of *Staphylococcus aureus* subsp. *aureus* strain Newman D2C (ATCC 25904; ATCC, Manassas, VA) [42]. A 30-mL volume of an overnight culture of *S*. *aureus* Newman D2C was boiled for 10 min and then centrifuged for 10 min at 10,000 × *g*. The pellet was resuspended in 10 mM Na_2_PO_4_ (pH 7.5) to an OD_600_ of approximately 0.8. A 100-μL aliquot of bacterial supernatant was then added to 900 μL of *S*. *aureus* suspension, and the OD_600_ was determined at 5 min intervals for 60 min (beginning at time 0) and then at 90 and 120 min [43,44].

**Pyocyanin levels** within supernatant fractions of PAO1 were determined as previously described. Samples were collected at different time points from 4 to 72 h and the amount of pyocyanin was calculated using the formula OD_520_ × 17.072 = μg/mL of pyocyanin [45].

**Pyoverdine levels** were analyzed by measuring the OD_405_ values of each supernatant sample of PAO1. Samples were collected at different time points from 4 to 72 h. Values were adjusted by dividing OD_405_ readings by their corresponding OD_600_ values.

### β-Galactosidase assays for gene expression

Overnight cultures of PAO1 containing plasmid pMP190::*pvdD-lacZ* (a transcriptional fusion plasmid) [46] or pMW303 (pEX1.8 carrying a *phzA1B1C1-lacZ* translational fusion reporter) [47] were resuspended in fresh LBB, LBBS, or fractionated LBBS (see below) to a final OD_600_ of 0.02–0.03. Cultures were incubated at 37°C with shaking at 200 RPM. At specific time points between 4 and 16 h of growth, cells in 1-mL aliquots of the cultures were harvested by centrifugation at 4000 × *g* for 1 min. β-galactosidase assays were performed as previously described [48–50]. Cells were lysed and the units of β-galactosidase activity were determined. The calculation for units of activity includes the amount of growth [50].

### Fractionation of LBBS

LBBS was fractionated by centrifugation for 15 min at 8,000 × *g* using Vivaspin centrifugation columns with 50-, 30-, 10-, or 5-kDa molecular weight cutoffs (Vivaproducts, Littleton, MA) according to the manufacturer’s instructions. The filtrate (containing proteins smaller than the MWCO of the column) and the retentate (containing proteins larger than the MWCO of the column) were collected separately. As a control, LBB was fractionated in a similar manner.

To determine if the observed effect on *phzA1-G1* expression is due to a small peptide (<10 kDa), we boiled LBBS fraction containing those proteins (LS<10) for 5 min at 100°C and cooled the fraction to 22°C (LS<10-HI). To exclude the contribution of non-polar lipids such as hormones and steroids or cytokines that can be absorbed with charcoal [51–53], we added 50 mg/mL activated charcoal (MilliporeSigma) to LS<10, incubated the medium for 15 min at room temperature, and removed the charcoal by centrifugation for 10 min at 10,000 × *g* (LS<10-CT).

### Isolation of PAO1 outer membrane proteins and SDS-PAGE

LBB and LBBS were inoculated with PAO1 in triplicate as described above and grown at 37°C for 16 h with shaking at 200 RPM. Cultures were centrifuged and cells were washed and lysed by sonication (10 min pulses at 50% power 3 times) using a Kinematica Polytron P10-35 PCU-11 homogenizer) (Kinematica AG, Luzern, Switzerland). Lysed cells were pelleted by centrifugation for 20 min at 18,450 × *g*. The pellet (containing both inner and outer membranes) was then resuspended in sterile water, sarcosyl (MilliporeSigma) was added to a final 1% concentration, and the sample was incubated for 1 h rotating at room temperature to remove most of the inner membranes [54]. The samples underwent ultracentrifugation for 1 h at 100,000 × *g*, pellets were collected and washed with water, and then centrifuged for an additional 30 min at 100,000 × *g*. The resulting outer membrane protein (OMP)-rich pellet was resuspended in sterile water and the total protein content of each sample was determined using Bradford estimation (Bio-Rad, Hercules, CA). Equal amounts of the prepared OMP (50 μg) were separated by SDS-PAGE and gels were stained by silver staining to visualize the OMP [55].

### Statistical analyses

Statistical analyses of the results were done using GraphPad Prism 8.4.2 (GraphPad Software, La Jolla, CA). One-way analysis of variance (ANOVA) with Tukey’s multiple comparison posttest was used to analyze changes in levels across time. Two-tailed, unpaired *t-*tests were performed to determine significance between pairs of data. One-way ANOVA with Dunnett’s multiple comparison posttest was used to determine significance of different among fractions of LBB and LBBS using untreated LBB or LBBS as controls.

## Results

### Growth of PAO1 in WBHVs altered its transcriptome

**Fig 1:**
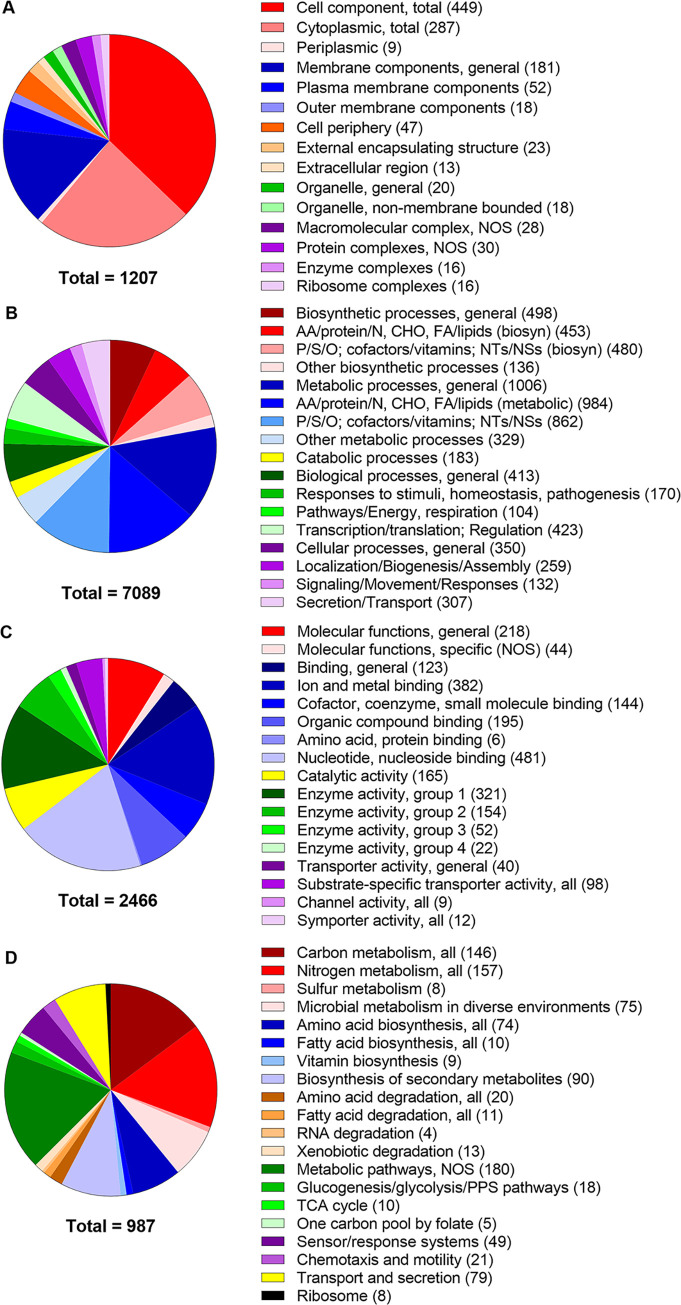
Gene Ontology (GO) functional terms assigned to the products of the 1085 differentially expressed genes. The 1085 genes whose expression was up- or downregulated by the growth of PAO1 in WBHVs compared to its growth LBB were analyzed by GO enrichment analysis using ShinyGO v0.61 and the PAO1 reference genome from the *Pseudomonas* Genome Database. The GO and KEGG pathway terms were considered to be significantly enriched if their false discovery rate *p* value was <0.05. (A) Cellular components (1207 terms out of 6533 terms were considered significant), (B) biological processes (7089 of 33620), (C) molecular functions (2466 of 12341), and (D) KEGG pathways (987 of 3711). In all four sections, some genes were assigned more than one term and most terms were assigned to more than one gene. AA, amino acids; CHO, carbohydrates; enzyme group 1: transferases, oxoreductases, hydrolases; enzyme group 2: peptidases, lyases, ligases, phosphorylases; enzyme group 3: racemases/epimerases, synthases, oxidases, dehydrogenases, dehydratases; enzyme group 4: carboxylases, isomerases, transaminases, dismutases; FA, fatty acids; N, nitrogen; NOS, not otherwise specified; NTs/NSs, nucleotides/nucleosides; PPS, pentose-phosphate shunt; P/S/O, phosphorus/sulfur/oxygen; TCA, tricarboxylic acid cycle.

Results from our previous investigations showed that severe burn- or trauma-induced changes in blood differentially altered the expression of numerous *P*. *aeruginosa* genes in diverse ways [36,37]. To determine the effect of whole blood from a healthy host on the *P*. *aeruginosa* transcriptome, we grew the fully virulent *P*. *aeruginosa* strain PAO1 [38] separately in WB from three HVs (obtained through a protocol approved by the Institutional Review Board at Texas Tech University Health Sciences Center) or the laboratory medium LBB. LBB and WBHV were inoculated and grown for 4 h as described in Materials and methods. Cells were harvested and RNA was obtained for analysis. Based on our RNA-Seq analysis, gene expression was considered significantly altered if the change in the level of expression was ≥ twofold. As seen in several studies that involve normal human subjects, variations among the blood samples obtained from the three HVs were expected to occur. Such variations are reflected in the variable values of expression of any single gene (S2 Table). Taking this into consideration with our analysis, we defined the expression of a gene as enhanced when the increase in the fold change was ≥ twofold in PAO1 that was grown in each of the three blood samples. Similarly, the expression was considered repressed when the reduction in expression was ≥ twofold in PAO1 grown in each of the three blood samples. Thus, our main focus was on the direction of the change in gene expression rather than the variations in the level of expression upon the growth of PAO1 in the three blood samples. The RNA-Seq analysis identified 1085 genes whose expression was considered significantly altered by the growth of PAO1 in WBHV compared to its growth in LBB. The data for the samples have been deposited in the BioProject at NCBI (https://www.ncbi.nlm.nih.gov/bioproject/) under PRJNA287707. Of these 1085 genes, 747 were significantly upregulated, while 338 genes were significantly downregulated. We categorized the gene products by gene ontology (GO) enrichment analysis using ShinyGO v0.61 (available at http://bioinformatics.sdstate.edu/go/) [56] and the *P*. *aeruginosa* PAO1 reference genome (*Pseudomonas* database; http://www.pseudomonas.com/) [57–59]. GO terms and terms associated with KEGG pathways were counted in this analysis if their enrichment false discovery rate *p* value was ≤0.05. The 1085 genes returned multiple terms considered significantly enriched within the three GO categories and among the KEGG pathways: cellular components (1207 terms), biological processes (7089), molecular functions (2466), and KEGG pathways (987) (Fig 1). Cellular components (not otherwise specified) and cytoplasmic and membrane proteins dominated the cellular components (Fig 1A), metabolic processes formed the majority of the biologic processes (Fig 1B), and binding functions led the molecular functions (Fig 1C). The KEGG pathway analysis was dominated by metabolism and metabolic pathways (Fig 1D).

### Growth of PAO1 in WBHVs downregulated the expression QS and QS-regulated virulence genes

**Fig 2:**
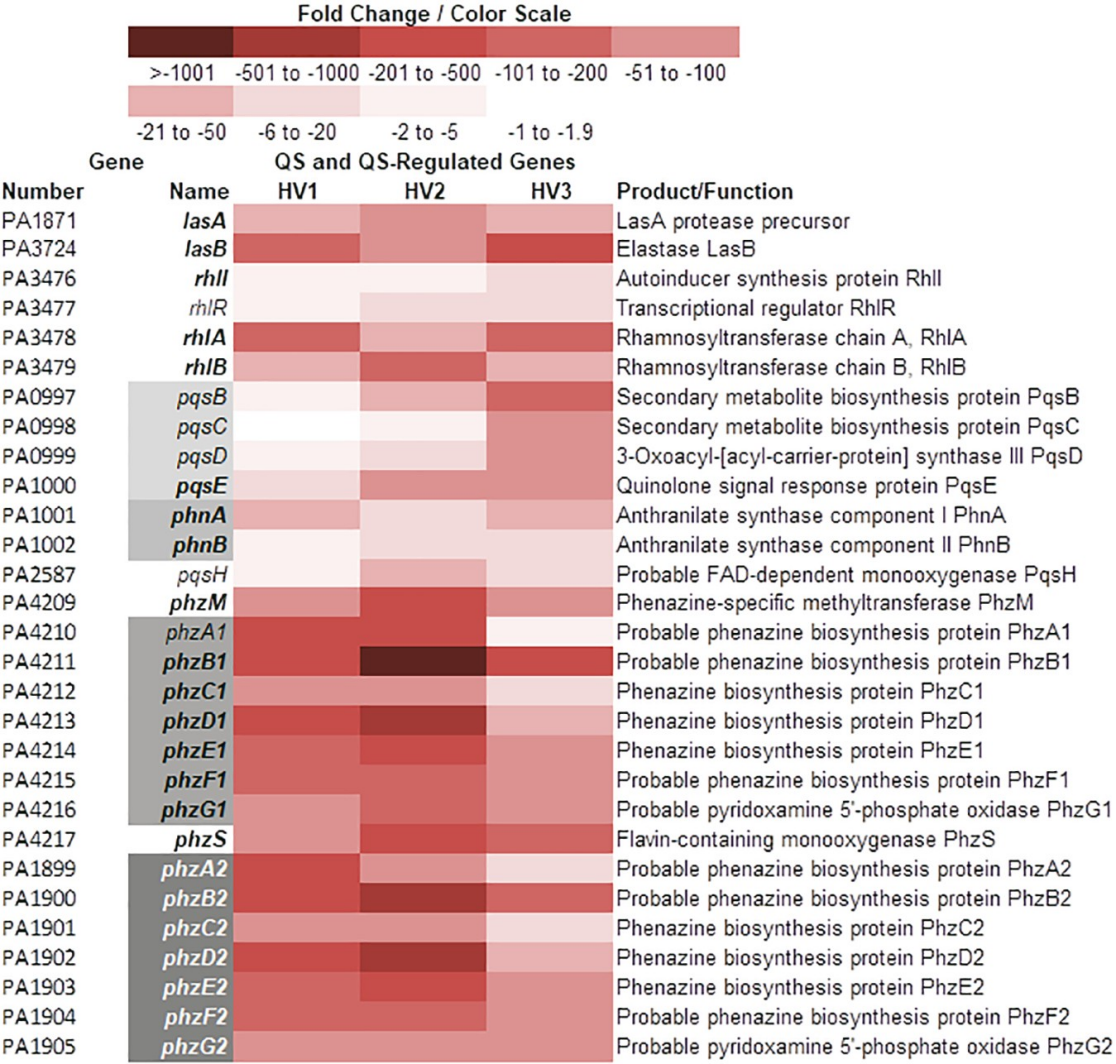
Color scale map depicting the changes in expression of genes involved in quorum sensing (QS). Expression of genes by PAO1 grown in WBHVs for 4 h was compared with their expression when PAO1 was grown in LBB for 4 h. Information regarding gene product functions as well as the organization of their operons was obtained from the *Pseudomonas* Genome Database. Red shading indicates levels of downregulation of the genes (WBHV compared to LBB); bold text indicates *q* value ≤ 0.05 and fold change ≥ 2.00 for all 3 samples; regular text, fold change ≥ 2.00 but *q* value > 0.05 for 1 sample; gray shading indicates genes composing operons.

The expression of many QS and QS-controlled genes was repressed by the growth of PAO1 in WBHVs. The reduction in the expression of several QS-related genes was pronounced (Fig 2). For example, *lasB*, *phzB1* and *phzB2* expression was reduced >200-fold and expression of *rhlA* and *rhlB* was reduced between 96- and 87-fold, respectively (Fig 2). Two almost identical *phz* operons, *phzA1-G1* and *phzA2-G2*, code for the enzymes that synthesize pyocyanin [31], a secondary metabolite that interferes with host cellular metabolism [29,30,60]. Overall, the expression of all seven genes of each *phz* operon was significantly reduced in parallel except the reduction in *phzB1* expression was about four times greater than that of *phzB2* (Fig 2). Additionally, the expression of the pyocyanin processing genes *phzH*, *phzM*, and *phzS* was reduced (Fig 2).

**Fig 3:**
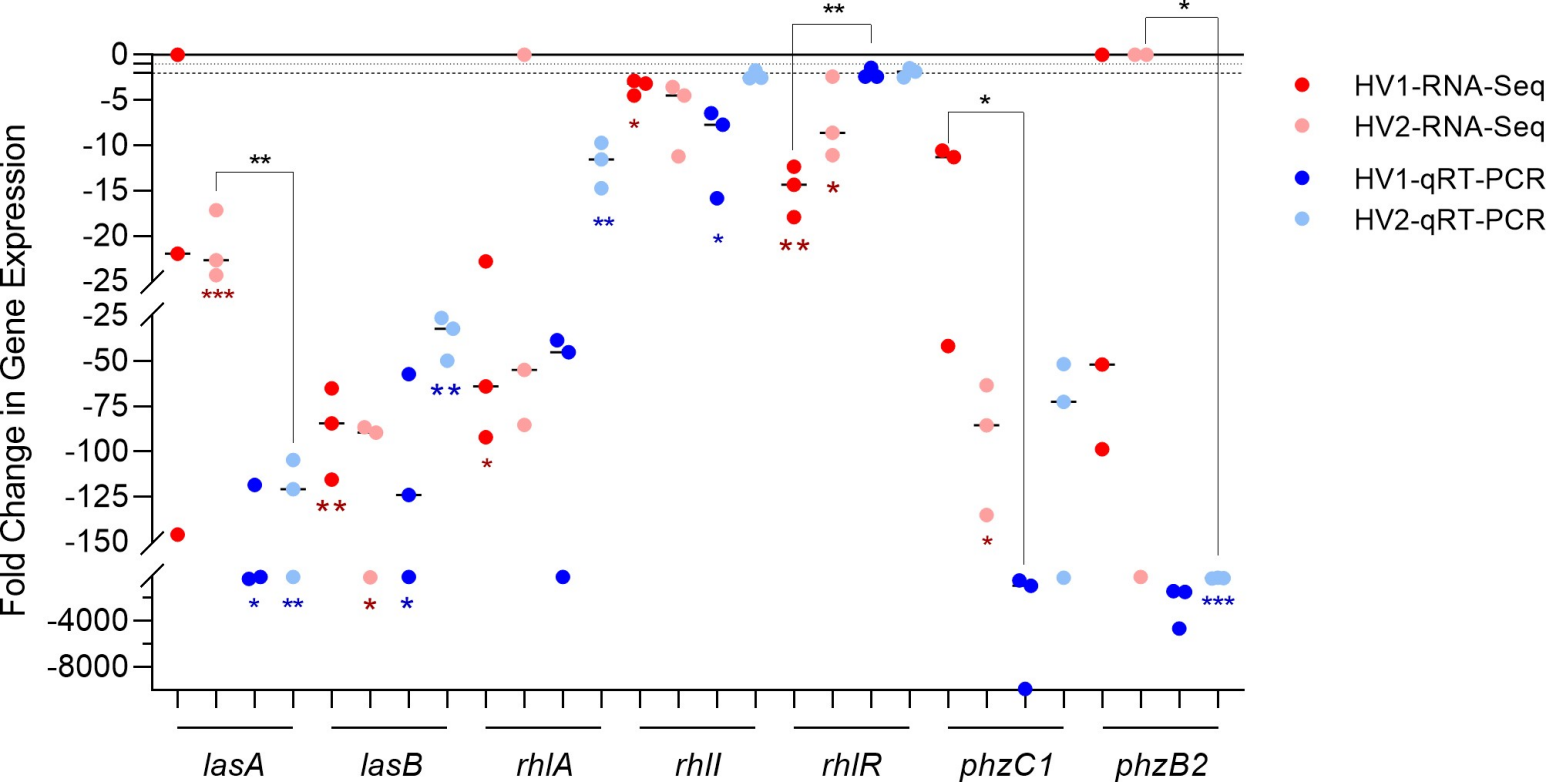
Confirmation of reduction in gene expression observed by RNA-Seq. The level of expression of the indicated genes was determined by qRT-PCR using the same RNA samples as templates. Only two of the three HV samples were sufficient for parallel testing, those from HV1 and HV2. PAO1 gene expression at 4 h post-inoculation in WBHV is relative to its expression in LBB at the same time point; dashed line, twofold change. Bars in each column indicate the median of 3 replicates on 2 independent samples. No significant differences were determined by two-tailed *t-*test.

To confirm the reduction in QS gene expression observed in the RNA-Seq analysis, we analyzed the expression of some of these genes by qRT-PCR using the same RNA samples as templates. Only two of the three HV samples were sufficient for parallel testing, those from HV1 and HV2. The samples were assayed individually and the results shown are the averages of three replicate tests on each sample, whether RNA-Seq or qRT-PCR. Expression of *lasA*, *lasB*, *rhlR*, *rhlI*, *rhlA*, *phzC1*, and *phzB2* was reduced (Fig 3). Similar to the results of the RNA-Seq analysis, the results of the qRT-PCR revealed that the expression of these genes is reduced upon the growth of PAO1 in whole blood (compared to its growth in LBB) (Fig 3). Compared to the level of reduction in expression based on the RNA-Seq analysis, the level of reduction based on the qRT-PCR was comparable and in the same direction. Evaluation of the genes originally found to be significantly up- or downregulated (1208 genes) revealed 123 genes whose pattern of expression was not consistent among PAO1 grown in each of the three blood samples (S2 Table). Among those genes are *pqsA* and *lasI* (S2 Table). However, the qRT-PCR analysis of the same RNA sample showed a reduction in *pqsA* expression in both samples analyzed while the expression of *lasI* was increased in both (S1 Fig). The level of *lasR* expression was not significant in the RNA-Seq; therefore, it was not examined by qRT-PCR. These results are puzzling, considering that the expression of both *lasB* and *lasA*, stringently controlled by the *las* QS system, was reduced significantly in both assays (Figs 2 and 3, S1 Table).

### Growth of PAO1 in WBHVs enhanced type III secretion system gene expression

**Fig 4:**
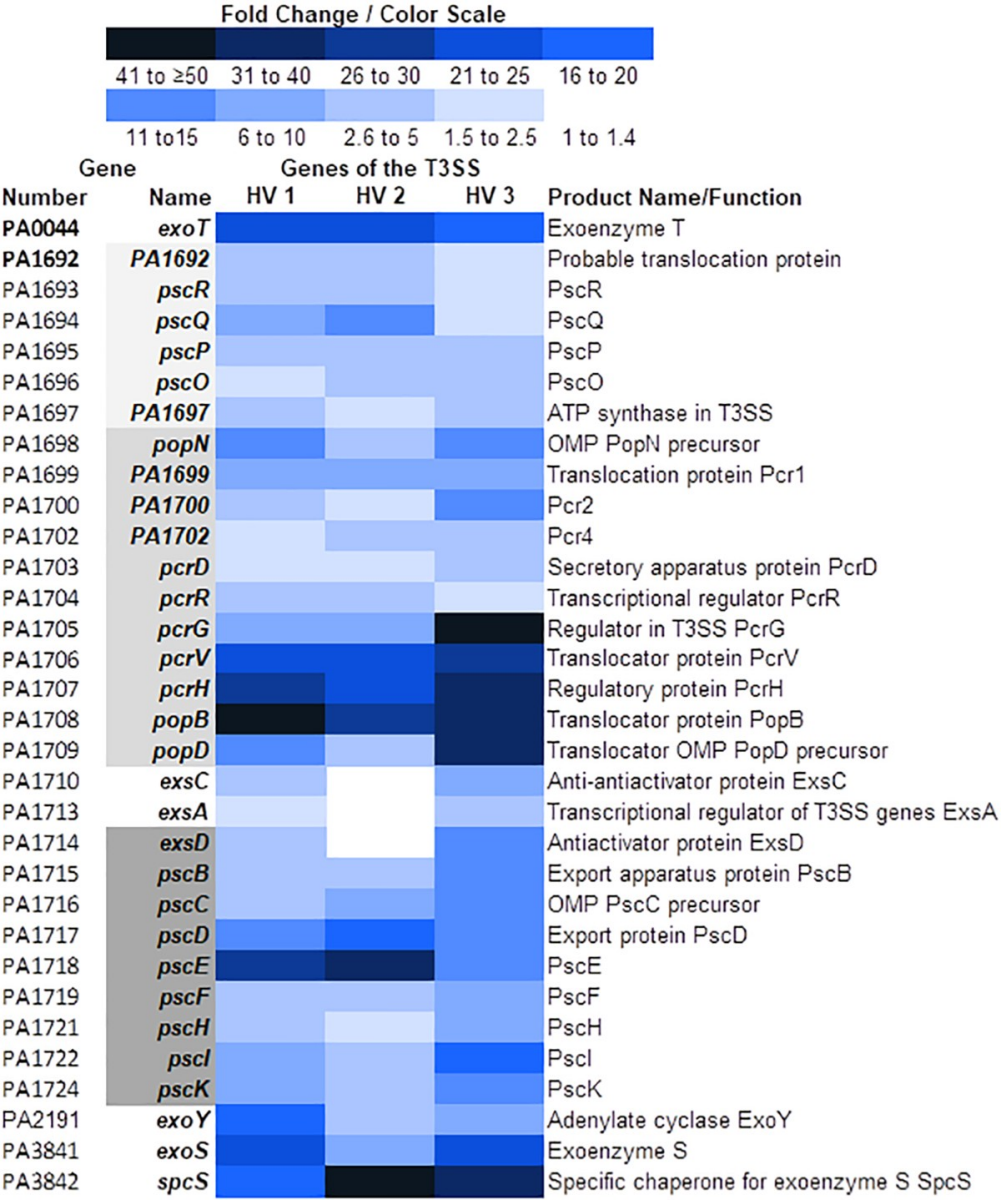
Color scale map depicting changes in expression of genes of type III secretion system. Expression of genes by PAO1 grown in WBHVs for 4 h was compared with their expression when PAO1 was grown in LBB for 4 h. Information regarding gene product functions as well as the organization of their operons was obtained from the *Pseudomonas* Genome Database. Blue shading indicates levels of upregulation of the genes (WBHV compared to LBB); bold text indicates *q* value ≤ 0.05 and fold change ≥ 2.00 for all three samples; regular text, fold change ≥ 2.00 but *q* value > 0.05 for one of the three samples; gray shading indicates genes composing operons; OMP, outer membrane protein; T3SS, type III secretion system.

The *P*. *aeruginosa* T3SS is a highly complex system that includes five components encoded by 40 genes in PAO1 [61]. The system includes the needle complex, the translocation apparatus, three effector proteins (exoenzymes S, T, and Y in strain PAO1), one chaperone specific for exoenzyme S, and proteins that regulate transcription of T3SS genes and secretion of the effector proteins [61]. Previous studies showed that the T3SS contributes to *P*. *aeruginosa* pathogenesis during infection, including burn wound infections and acute pneumonia [62,63]. We recently showed that compared with the growth in WBHVs, the growth of *P*. *aeruginosa* in whole blood from severely burned patients significantly enhanced the expression of numerous T3SS genes [37]. Similarly, compared to its growth in LBB, the growth of PAO1 in WBHVs significantly enhanced the expression of 32 of the 40 T3SS genes found within PAO1, with an average enhancement ranging from twofold to 37-fold (Fig 4). The greatest enhancement was detected among the genes of the *popNpcr1234DRGVHpopBD* operon, which encodes proteins of the translocation apparatus and regulatory proteins, and the gene for SpcS, the specific chaperone for exoenzyme S (Fig 4).

### Growth of PAO1 in WBHVs enhanced expression of pyoverdine siderophore genes

**Fig 5:**
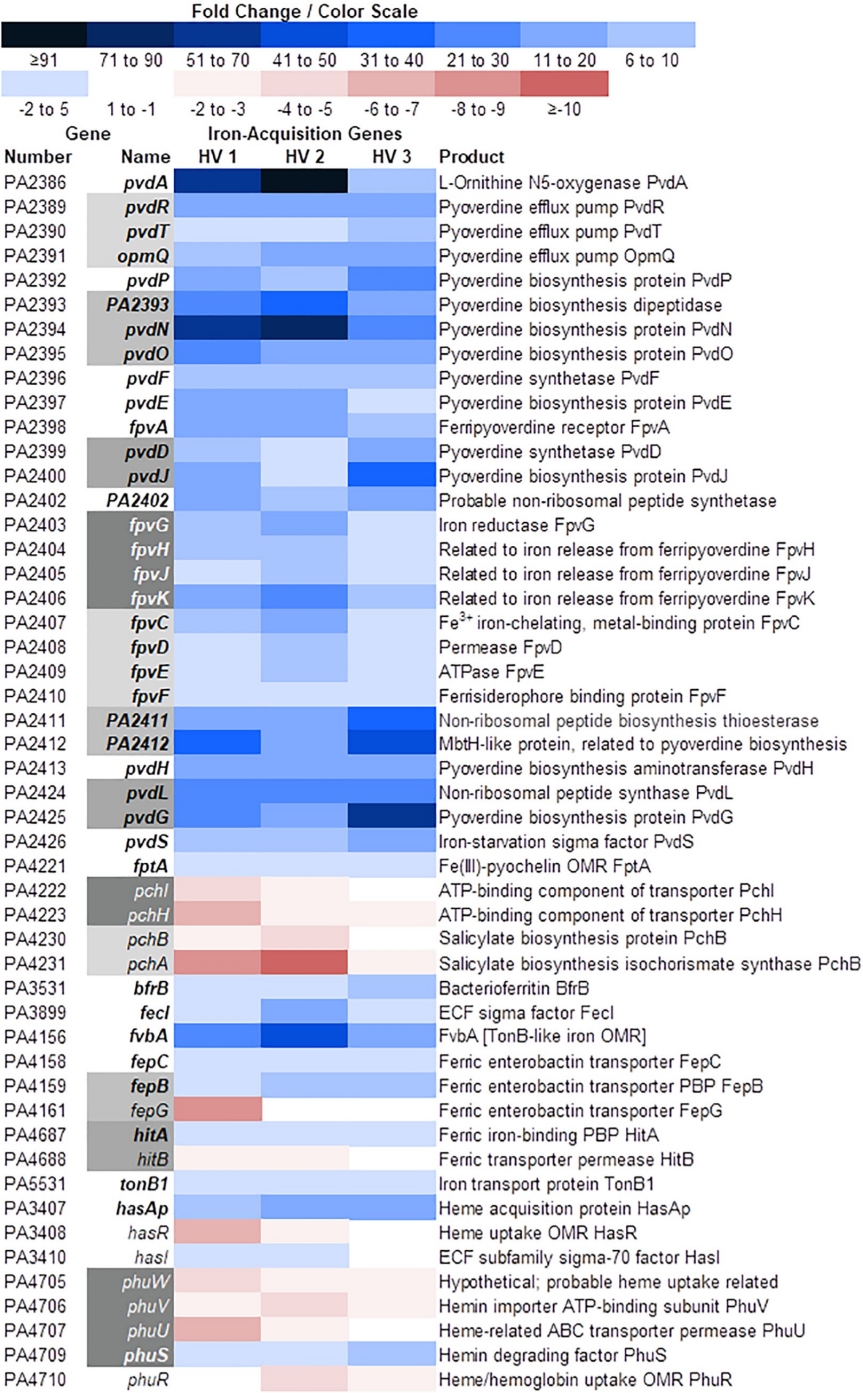
Color scale map depicting changes in expression of genes involved in iron acquisition. Expression of genes by PAO1 grown in WHBVs for 4 h was compared with their expression when PAO1 was grown in LBB for 4 h. Information regarding gene product functions as well as the organization of their operons was obtained from the *Pseudomonas* Genome Database. Blue shading indicates levels of upregulation of the genes and red shading indicates levels of downregulation of the genes (WBHV compared to LBB); bold text indicates *q* value ≤ 0.05 and fold change ≥ 2.00 for all three samples; regular text, fold change ≥ 2.00 but *q* value > 0.05 for one of the three samples; gray shading indicates genes composing operons; [], putative function; ECF, extracytoplasmic function; OMR, outer membrane receptor; PBP, periplasmic binding protein.

At different infection sites, free iron is sequestered within iron-binding proteins such as transferrin and lactoferrin [64]. In response, the infecting *P*. *aeruginosa* produces iron-scavenging molecules known as siderophores, the most characterized of which are pyoverdine and pyochelin [65]. Numerous *P*. *aeruginosa* genes encode the proteins required for the production and transport of each siderophore. During *P*. *aeruginosa* bloodstream infection in severely burned patients, expression of multiple pyoverdine- and pyochelin-related genes was significantly enhanced [37]. Thus, we examined expression of these genes when PAO1 was grown in blood from HVs. Compared to its growth in LBB, the growth of PAO1 in WBHVs significantly enhanced the expression of 15 pyoverdine synthesis genes, including *pvdA*, *pvdN*, and *pvdQ*, 11 genes related to efflux of pyoverdine and release of iron from ferripyoverdine, and the positive regulator for pyoverdine biosynthesis *pvdS*; expression of *fpvA* that encodes the ferripyoverdine receptor was also enhanced (Fig 5). In contrast, the expression of the pyochelin synthesis genes was reduced (Fig 5). Similar to *fpvA*, expression of the ferripyochelin receptor gene *fptA* was also enhanced (Fig 5). At this time, the cause of the variation in the level of expression between the pyoverdine and pyochelin synthesis genes is not known.

### Growth of PAO1 in the presence of human serum altered the expression of QS and QS-related virulence genes

**Fig 6:**
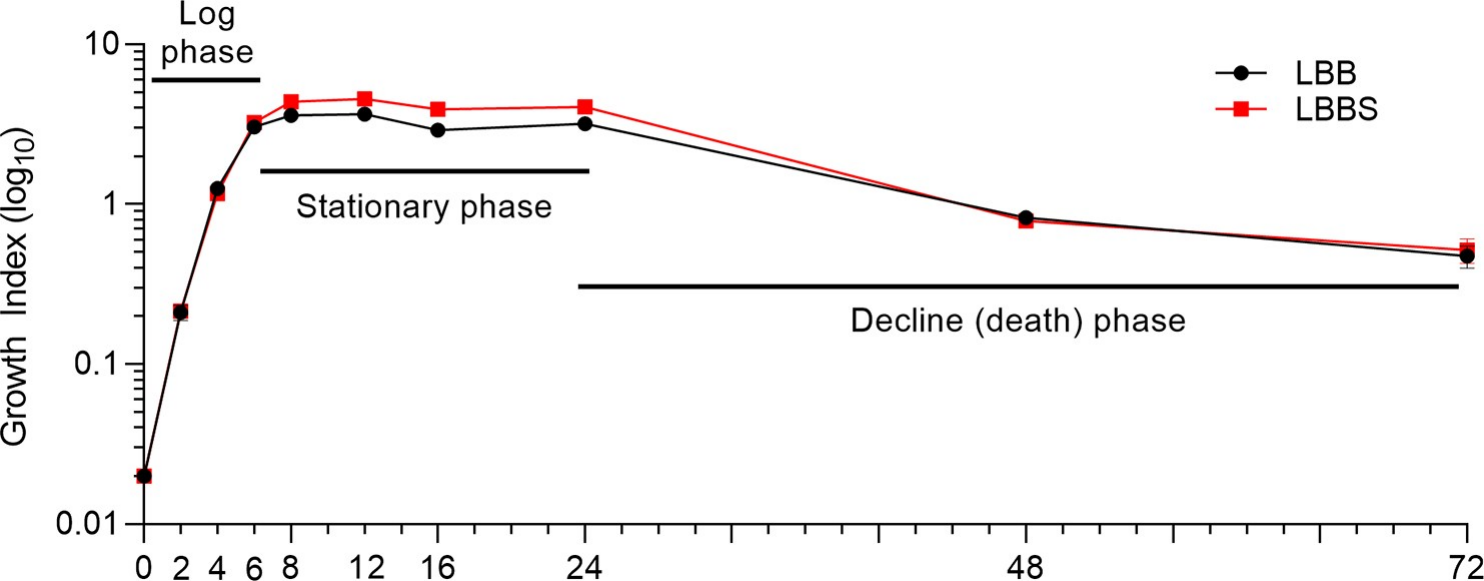
Growth of PAO1 in the presence of 10% PHS paralleled its growth in LBB. PAO1 was inoculated at OD_600_ ~0.020 into LBB or LBBS and incubated with shaking at 200 RPM to the time points indicated on the graph and the OD_600_, representative of the growth index, was determined. Data were log-transformed before graphing. Values represent the means of 3 independent experiments ± SEM. One-way ANOVA comparing pairs of time points revealed no significant differences between growth in LBB and LBBS.

Serum, the fluid left after clot formation in whole blood, contains many different proteins such as albumin, globulins (primarily immunoglobulins), transferrin, hormones, and enzymes (but no fibrinogen or clotting factors) as well as numerous small molecule metabolites and ions [66]. Potential factors within whole blood that influence *P*. *aeruginosa* gene expression are likely to be localized to the serum fraction. Besides the possibility of localizing the potential influencing factor, serum-based experiments offered key experimental advantages. First, in experiments using WB, our analysis was limited to a specific time point in the *P*. *aeruginosa* growth cycle (~ 4 h post inoculation) due to extensive lysis of the red blood cells at later time points (7% at 8 h and 71% by 12 h post inoculation), which interferes with growth of the organism and analysis of gene expression [37]. Use of serum-based experiments allowed extension of the experimental design to different time points throughout the *P*. *aeruginosa* growth cycle. Second, in experiments using WB, potential inherent variations between blood samples (including age and sex of HVs and their dietary habits) cannot be eliminated. These variations were eliminated in serum-based experiments by the use of commercially-available pooled serum prepared from at least 10 adult HVs (PHS) (MilliporeSigma, St. Louis, MO). We assessed the effect of 10% PHS on the expression of relevant genes by growing PAO1 in either LBB or LBB containing 10% PHS (LBBS) as previously described [67]. Using the same standardized PAO1 inoculum that was used for the transcriptome analysis, PAO1 consistently reached a similar growth index (OD_600_) from 2 to 72 h post-inoculation whether grown in LBB or LBBS (Fig 6). There were no significant differences at any time point (Fig 6). The figure clearly shows the log, stationary, and decline phases of growth (Fig 6).

**Fig 7:**
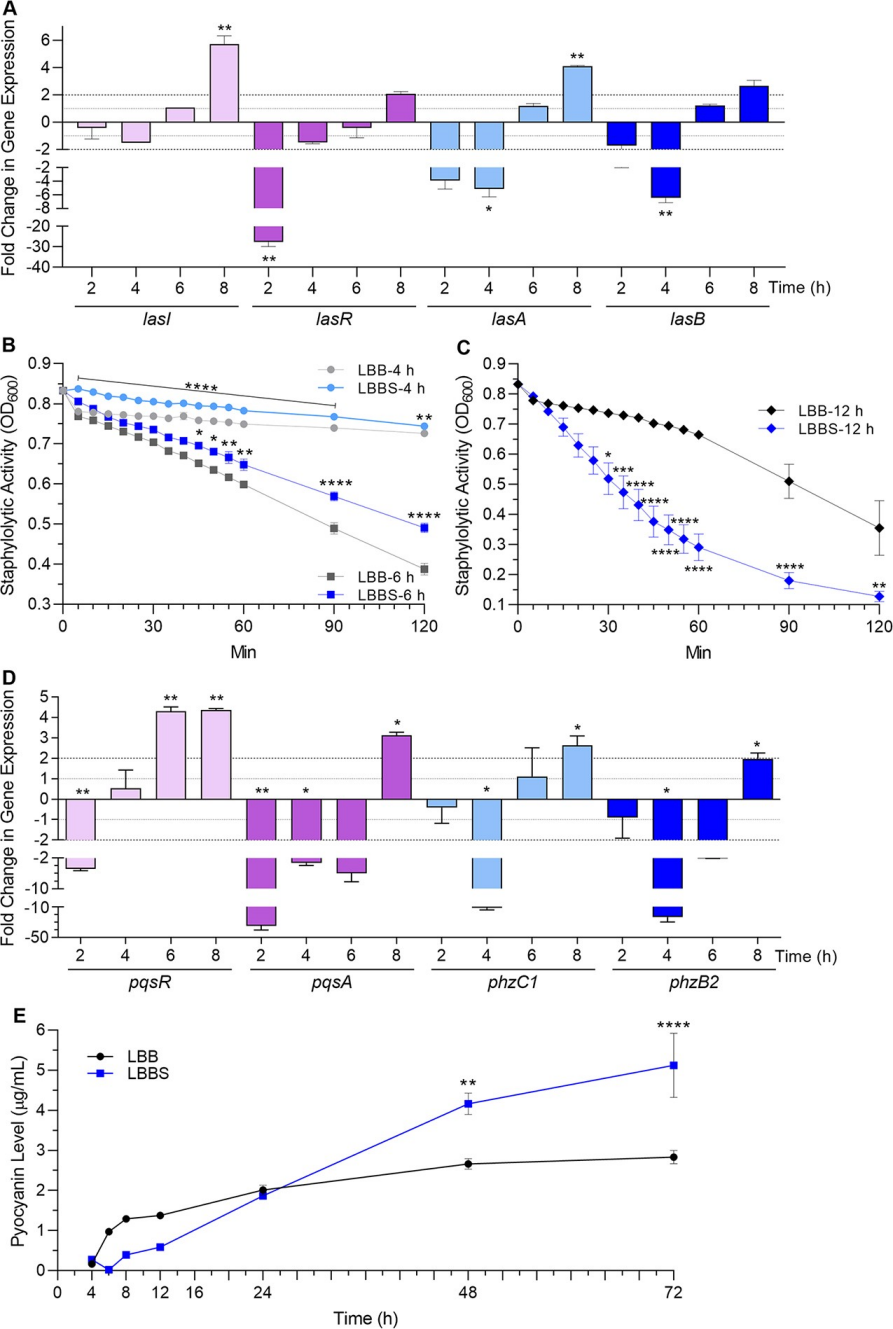
Growth in 10% PHS affected expression of QS genes and synthesis of QS-related virulence factors. For all experiments, PAO1 was grown in either LBB or LBBS and samples were collected at the time points indicated on each figure. Values represent the means of 3 independent experiments ± SEM for all experiments presented in the figure. (A) *las* system gene expression: the level of expression of the indicated genes was determined using qRT-PCR. Fold change in gene expression at each time point is relative to its expression in LBB at the same time point (onefold level of expression, dotted line). Significance was determined by two-tailed *t-*test and the cut-off of ≥ twofold (dashed line); *, *P* < 0.05; **, *P* < 0.01. (B and C) LasA activity: supernatant fractions were collected at (B) 4 h and 6 h post inoculation and at (C) 12 h post inoculation. Staphylolytic activity of LasA was determined as described in Materials and methods. Significance was determined by one-way ANOVA with Tukey’s multiple comparisons posttest; *, *P* < 0.05; **, *P* < 0.01; ***, *P* < 0.001; ****, *P* < 0.0001. (D) *pqs* system gene expression: PAO1 was grown and samples were collected and analyzed as described in (A). Significance was determined by two-tailed *t-*test and the cut-off of ≥ twofold (dashed line); *, *P* < 0.05; **, *P* < 0.01. (E) Pyocyanin production: supernatant fractions were collected throughout the growth cycle from 4 h to 72 h. Pyocyanin levels were assayed as previously described (Materials and methods). Significance was determined by one-way ANOVA with Tukey’s multiple comparisons posttest; **, *P* < 0.01; ****, *P* < 0.0001.

Using qRT-PCR to assess gene expression, we found that, as seen with its growth in whole blood, the relative expression of *lasR* and *lasI* in PAO1 grown in LBBS compared to LBB was reduced at early time points of growth (2 and 4 h) (Fig 7A). Expression of these genes increased by 6 h, and was significantly enhanced by 8 h (Fig 7A). Among the different *las* QS-controlled virulence factors, LasB and LasA are the most stringently controlled [68,69]. Expression of the *las-*regulated virulence factor genes *lasA* and *lasB* followed the same pattern of expression as *lasR* and *lasI* (Fig 7A). To determine whether LasB synthesis and secretion followed this established pattern, we used the previously described elastin Congo red assay [70]; however, serum by itself produced too much elastase activity to interpret the assay. As an alternative, we tried to detect the LasB protein using immunoblotting experiments with specific LasB polyclonal antibodies; again, elastase within the serum cross-reacted with the antibody and prevented determination of the presence of LasB. Next, we examined PAO1 LasA activity using the previously described staphylolytic assay [44]. Similar to the reduced expression of *lasA* seen at 2, 4, and 6 h of growth post-inoculation, human serum significantly repressed LasA production by PAO1 at 4 and 6 h (Fig 7A and 7B). While *lasA* gene expression was enhanced in the presence of serum at 8 h post-inoculation (Fig 7A), LasA production was significantly enhanced at 12 h post-inoculation (Fig 7C).

We then examined the expression of the *pqs* transcriptional activator *pqsR* and one of the PQS synthesis genes *pqsA*. Expression of *pqsR* was repressed at early stages of PAO1 growth in LBBS (2 and 4 h post-inoculation) and enhanced by 6 h post-inoculation while *pqsA* expression did not rise until 8 h post-inoculation (Fig 7D). Expression of *phzC1* and *phzB2*, representative of the two *phz* synthesis operons, was similar to that of *pqsA* (Fig 7D). The production of pyocyanin followed this pattern but remained reduced through 12 h post-inoculation, began to rise at 24 h, and became significantly enhanced only at the very late (48 and 72 h) stage of the PAO1 growth cycle in LBBS (Fig 7E). Thus, even though the expression of *phzA1-G1* and *phzA2-G2* was enhanced prior to the late stages of growth, synthesis and release of pyocyanin was delayed considerably beyond the expected time of 16 h [71,72].

### WB and 10% PHS regulate the expression of different regulators of the QS systems

**Fig 8:**
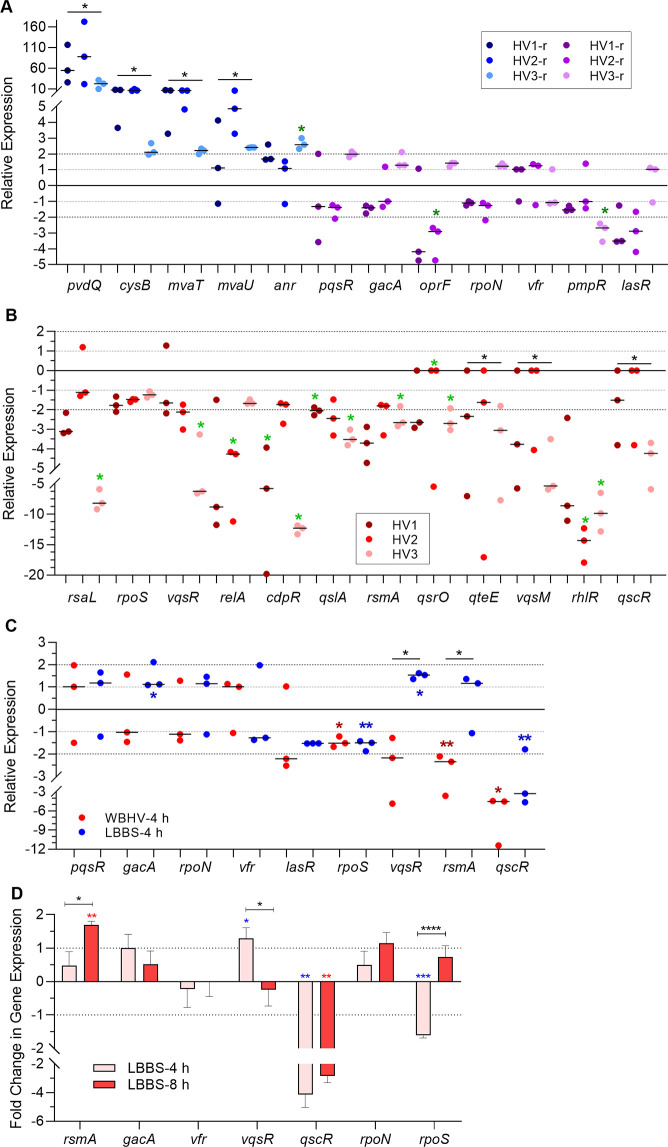
Growth in WBHVs or 10% PHS influenced the expression of QS regulatory genes. Fold change in gene expression at each time point is relative to its expression in LBB at the same time point (value of 1, dotted line). Dashed gray line indicates the cut-off for significance, fold change ≥ 2.00. (A and B) Fold change in expression at 4 h of growth post-inoculation of 18 genes that regulate the QS systems (expression in WBHVs compared to that in LBB). Values represent the means of 3 replicate tests from 3 independent samples (WBHV); the bar represents the median. The data shown are based on the complete RNA-Seq analysis; black asterisk * over a black bar indicates that change in gene expression was considered significant among all 3 individual HV samples using fold change ≥ 2.00 and *q* values ≤ 0.05; green asterisks indicate significant difference in gene expression for that individual sample. Values in shades of blue, all or 2 of 3 values for gene expression were above 0 among the three samples; values in shades of purple, some values for gene expression were above 0 and some values were below 0 (A); values in shades of red, all or 2 of 3 values for gene expression were 0 or below among the three samples (B). (C) Comparison of the effect of WBHVs with that of 10% PHS on the expression of selected QS regulatory genes at 4 h of growth of PAO1 post-inoculation. RNA-Seq analysis of the effect of WBHV is described in (A). To determine the effect of 10% PHS, PAO1 was grown in LBB or LBBS and the expression level was determined by qRT-PCR. Values represent the means of replicate tests from 3 independent samples; the bar represents the median. Significance was determined by unpaired two-tailed *t* test. Black asterisk over black bar, WBHV:LBBS; dark red asterisk, WBHV:LBB; dark blue asterisk, LBBS:LBB; *, *P* <0.05, **, *P* <0.01. (D) The effect of 10% PHS on the expression of selected QS regulatory genes in PAO1 grown in either LBB or LBBS for 4 h and 8 h post-inoculation. The level of expression of the indicated genes was determined using qRT-PCR. Values represent the means of 3 independent experiments ± SEM. Significance was determined by unpaired two-tailed *t* test; *, *P* <0.05; **, *P* < 0.01; ***, *P* < 0.001; ****, *P* <0.0001; LBBS:LBB at 4 h, blue *; LBBS:LBB at 8 h, red *; LBBS-4h:LBBS-8h, black *.

Besides *lasR*, *rhlR*, and *pqsR*, numerous genes that regulate one or more of the *P*. *aeruginosa* QS systems have been identified [17]. In the RNA-Seq analysis, expression of several genes was enhanced (*pvdQ*, *cysB*, *mvaT*, *mvaU*, and *anr*) (Fig 8A), expression of eight, including *pqsR*, *vfr*, *gacA*, and *lasR*, was relatively unaffected (≤ two-fold change) (Fig 8A); and that of 11 of these genes was repressed > twofold at 4 h of growth of PAO1 in WBHVs compared to its growth at 4 h in LBB (Fig 8B). Thus, it is possible that at the early stage of growth, WBHVs represses the expression of the QS genes either by repressing the expression of one or more of the positive regulatory genes (*oprF*, *rpoS*, *vqsR*, *relA*, and/or *vqsM*) (Fig 8B); or by enhancing the expression of the negative regulators *pvdQ*, *cysB*, *mvaT*, and *mvaU* (Fig 8A).

Similar to the effect of WBHV and at early time points of growth, 10% PHS significantly reduced the expression of *rpoS* and *qscR* QS genes (Fig 8C). Thus, the effect of PHS on the expression of one or more of the QS regulatory genes may parallel that of WB, suggesting that WB influences the expression of these genes through serum. Alternatively, the effect of either whole blood or serum may be unique. To examine that possibility, we determined, using qRT-PCR, the expression of several QS-regulatory genes of PAO1 that was grown in LBB or LBBS for 4 h post-inoculation. In contrast to the effect of WB, 10% PHS enhanced the expression of *gacA*, *rpoN*, *rsmA*, and *vqsR*, and reduced the expression of *vfr* (Fig 8C). However, similar to the effect of WB, 10% PHS reduced the effect of *rpoS* and *qscR* suggesting that these genes may be key regulators through which whole blood and serum influence the expression of the QS genes at early time points of growth (Fig 8C).

At different time points of PAO1 growth, human serum differentially regulated the expression of QS genes, significantly decreasing their expression at early stages of growth (2 and 4 h) but significantly enhancing it at later stages of growth (6 and 8 h) (Fig 7A and 7D). Thus, we determined if this influence occurs through one of the QS regulatory genes by comparing, using qRT-PCR, the expression of these genes in PAO1 that was grown to 4 h and 8 h post-inoculation in LBB and LBBS. Among all tested genes, only the expression of *rpoS* was significantly reduced at early stages of growth in either WB or LBBS (Fig 8C and 8D) but enhanced at later stages of growth in LBBS (Fig 8D).

### Growth of PAO1 in the presence of 10% PHS influenced the expression of siderophore-related genes

**Fig 9:**
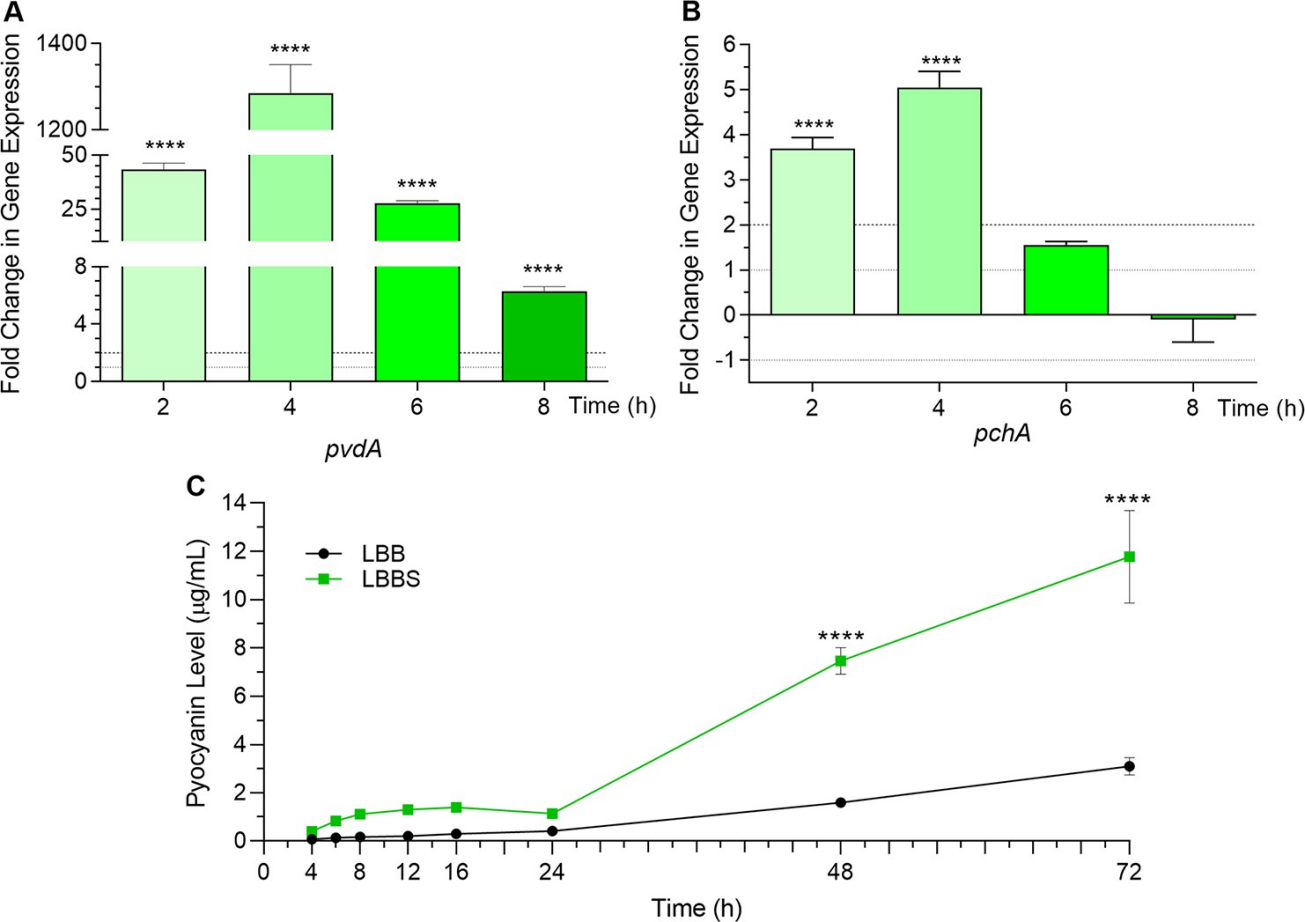
Growth in 10% PHS enhanced PAO1 expression of iron-acquisition genes. PAO1 was grown in either LBB or LBBS, and samples were collected at the time points indicated. Values represent the means of 3 independent experiments ± SEM for all experiments in the figure. The levels of expression of (A) *pvdA* and (B) *pchA* were determined by qRT-PCR. Fold change in gene expression at each time point is relative to its expression in LBB at the same time point (value of 1, dotted line). Significance was determined by two-tailed *t-*test and the cut-off of ≥ twofold (dashed line); ****, *P* <0.0001. (C) Compared with its growth in LBB, the growth of PAO1 in LBBS enhanced pyoverdine throughout the growth cycle. Supernatant fractions were collected at time points indicated and pyoverdine assays were conducted as described in Material and methods. Significant differences in pyoverdine levels were determined by one-way ANOVA with Tukey’s multiple comparison posttest; ****, *P* <0.0001.

The production of the siderophores pyoverdine and pyochelin by *P*. *aeruginosa* is suggested to be regulated by the QS system. The upstream region of the pyoverdine regulator *pvdS* contains a potential LasR binding site [17,73]. Additionally, PQS is known to bind iron, forming a PQS-iron complex that can transfer iron directly to the siderophores pyochelin and pyoverdine in iron retrieval [74–76]. Pyoverdine genes are upregulated in the absence of *pqsA*, suggesting a role for PQS in the negative regulation of *P*. *aeruginosa* iron-scavenging systems [75]. Results of the RNA-Seq analysis revealed that, while the expression of the pyoverdine synthesis genes was enhanced between five- and 58-fold by the growth of PAO1 in WBHVs, the expression of the pyochelin synthesis genes tended to be repressed (Fig 5). In the presence of 10% PHS, the expression of the pyoverdine gene *pvdA* was enhanced at 2, 4, 6, and 8 h post-inoculation of PAO1 in LBBS compared to LBB (Fig 9A). In contrast to the reduction produced by the growth of PAO1 in WBHV, expression of *pchA* was significantly enhanced by 3.5- and fivefold at 2 h and 4 h post-inoculation in LBBS, respectively (Fig 9B). At all time points, the increase in *pvdA* expression was considerably higher than that of *pchA* expression although expression of the two genes followed the same pattern–peaking at 4 h and decreasing after that (Fig 9A and 9B). Additional transcriptional analysis of the pyoverdine operon using a *pvdD*-*lacZ* transcriptional fusion system supported the above-described effect of serum on *pvdA* expression. The level of *pvdD* expression was significantly elevated from 4 h through 10 h post-inoculation and declined thereafter (at 12 and 16 h) (S2 Fig). Furthermore, pyoverdine production was increased throughout the growth cycle of PAO1; however, unlike *pvdA* and *pvdD* expression, pyoverdine production was greatest at the end of the growth cycle (Fig 9C). The variation between gene expression and pyoverdine production is expected as the pyoverdine molecule detected by the pyoverdine assay is synthesized by proteins encoded by the pyoverdine synthesis operon. It is possible that, rather than measuring the production of pyoverdine, we measured the accumulation of pyoverdine within the supernatant of PAO1 throughout the growth cycle. However, even if we consider this scenario of accumulated pyoverdine within PAO1 supernatant, the levels of pyoverdine were still significantly higher within the supernatant of PAO1 grown in LBBS.

### Growth of PAO1 in the presence of human serum albumin repressed the expression of QS and QS-related virulence genes

Serum contains several proteins that may differentially affect the expression of different PAO1 genes throughout its growth cycle. One of the most abundant serum proteins is albumin; a binding molecule that transports fatty acids and steroids throughout the bloodstream [77]. Smith *et al*. [78] recently demonstrated that the growth of *P*. *aeruginosa* in the presence of bovine serum albumin (BSA) reduced the expression of *lasI*, *rsaL*, *lasB*, and *rhlI* at 8, 12 and 24 h post-inoculation. Smith *et al*. [78] confirmed that BSA binds the *P*. *aeruginosa las* signaling molecule 3OC12-HSL and its degradation product C12-TA-HSL [78]. Based on these and other findings, Smith *et al*. [78] suggested that BSA interferes with the function of the QS system by directly sequestering 3OC12-HSL.

**Fig 10:**
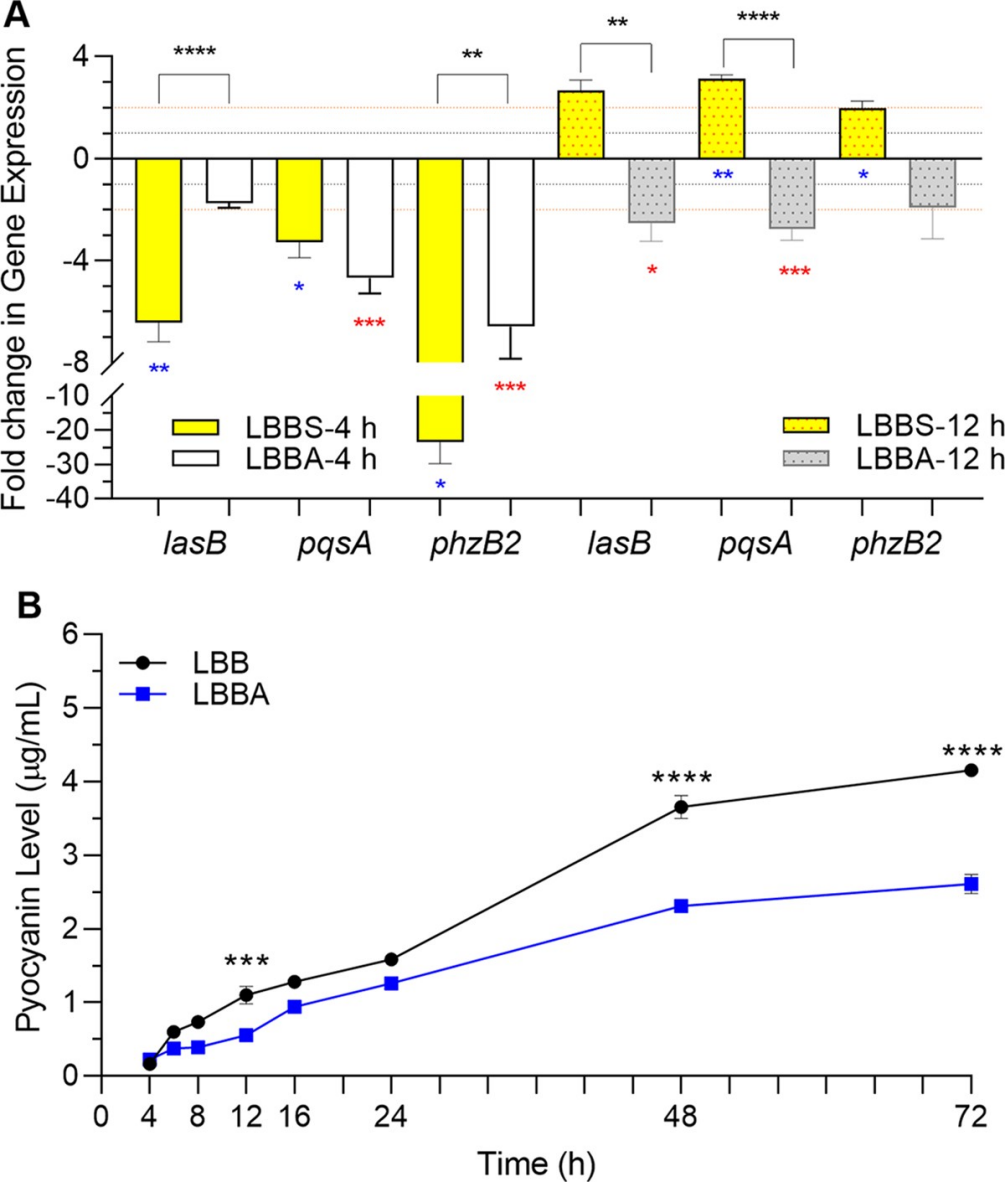
HSA at 10% physiological level regulated expression of *P*. *aeruginosa las-* and *pqs-*related genes. PAO1 was grown in LBB, LBBS, or LBBA and samples were collected at the time points indicated. Values represent the means of 3 independent experiments ± SEM for both experiments in the figure. (A) Expression of *lasB*, *pqsA*, and *phzB2* was determined by qRT-PCR. Fold change in gene expression at each time point is relative to its expression in LBB at the same time point (value of 1, dotted line). Significance was determined by two-tailed *t-*test and the cut-off of ≥ twofold (dashed line); *, *P* <0.05; **, *P* <0.01; ***, *P* <0.001; ****, *P* <0.0001. LBB:LBBA—red *; LBB:LBBS—blue *; LBBS:LBBA—black *. (B) Pyocyanin production is repressed by the presence of HSA. Pyocyanin assays were conducted on supernatant fractions as described in Methods. Significance was determined by one-way ANOVA with Tukey’s multiple comparison posttest; ***, *P* <0.001; ****, *P* <0.0001. The effect of LBBS is shown in Fig 7E.

As 10% PHS significantly influenced the expression of QS and QS-regulated genes at different time points throughout the growth cycle of PAO1 (Figs 7–9), we examined the effect of human serum albumin (HSA) (MilliporeSigma, St. Louis, MO), rather than BSA, on the expression of *lasB*, *phzB2*, and *pqsA*. Since we analyzed the effect of 10% serum in LBB, we adjusted the amount of added HSA to reflect 10% of the normal physiological levels (HSA-10%-pl) present within whole serum range (35–50 g/L) [79]; therefore, LBB plus HSA-10%-pl (LBBA) contained HSA at 3.5–5.0 g/L. There were no significant differences in PAO1 growth in LBBA compared to that in LBB (S3 Fig). The growth of PAO1 in LBBA significantly reduced *pqsA* and *phzB2* expression at 4 h post-inoculation; *lasB* expression was also reduced, but not significantly (Fig 10A). At 12 h post-inoculation, expression *lasB* and *pqsA* expression were significantly reduced; *phzB2* expression was also reduced, although not significantly (Fig 10A). We also examined the effect of HSA-10%-pl on the production of pyocyanin throughout the growth cycle of PAO1. HSA-10%-pl reduced pyocyanin production throughout the growth cycle of PAO1 (Fig 10B). Unlike the enhancement in pyocyanin levels observed at 48 and 72 h post-inoculation in the presence of 10% serum (Fig 7E), the presence of HSA-10%-pl significantly reduced pyocyanin production at 48 and 72 h post-inoculation (Fig 10B). Thus, in contrast to 10% PHS, which differentially regulated the expression of QS and QS-related genes, HSA-10%-pl produced a significant repressive effect throughout the PAO1 growth cycle, suggesting the presence of more than one component through which serum differentially regulates the expression of PAO1 genes. The mechanism by which HSA influences the expression of the QS genes is not known at this time.

### Serum factors <10-kDa in molecular weight enhanced PAO1 expression of *phz* genes at late stages of growth

**Fig 11:**
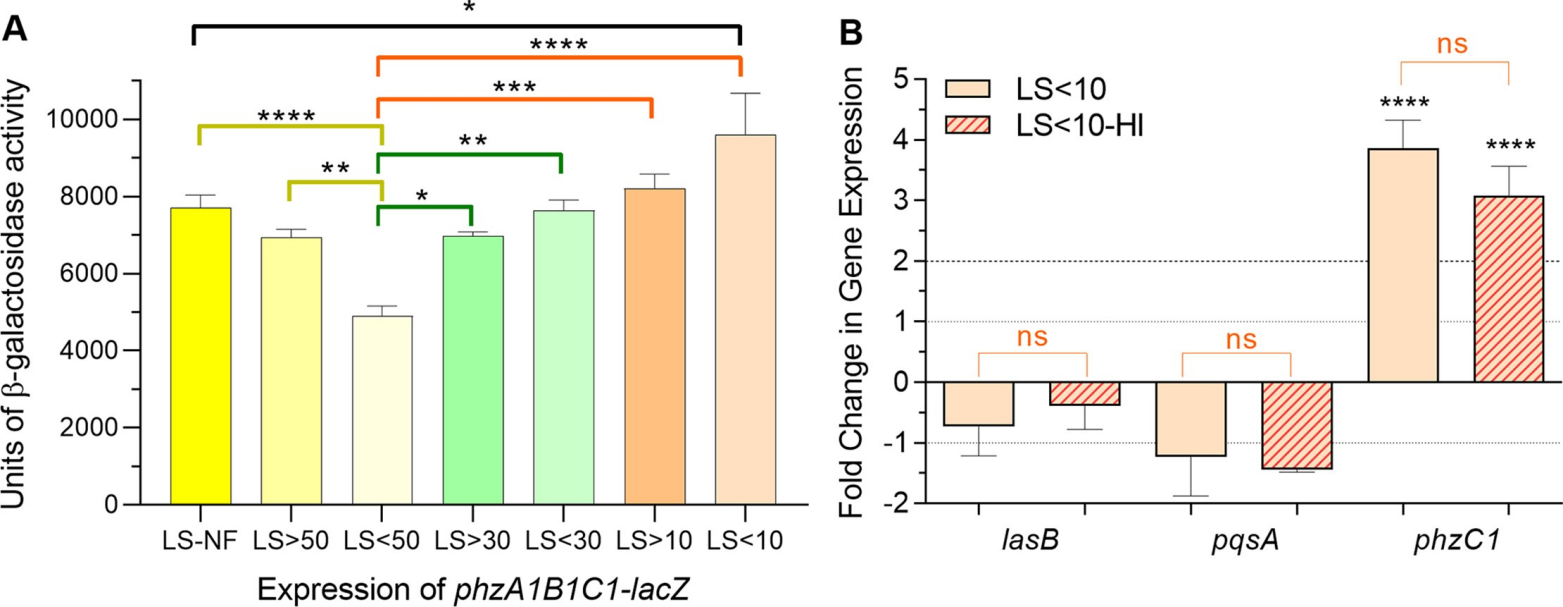
Different fractions of LBBS enhanced *phzA1B1C1* expression. (A) LBBS was fractionated using molecular weight cut-off columns of 50-, 30-, and 10-kDa. PAO1/pMW303, which carries a *phzA1B1C1-lacZ* transcriptional reporter fusion, was grown for 16 h post-inoculation in nonfractionated LBBS (LS-NF) or fractionated LBBS (LS>50, LS<50, LS>30, LS<30, LS>10 and LS<10). Cell pellets were collected and lysed, and β-galactosidase activity within the lysates was determined. Values represent the means of 3 independent experiments ± SEM. Significance was determined by one-way ANOVA with Dunnett’s multiple comparisons posttest using LB<50 as the control; *, *P* <0.05; **, *P* <0.01; ***, *P* <0.001; ****, *P* <0.0001. (B) Fraction LS<10 enhanced expression of *phzC1* but not *lasB* or *pqsA*. PAO1 was grown for 8 h post-inoculation in LBB, LS<10, or LS<10 that had been heat inactivated by boiling for 15 min (LS<10-HI). Expression of *lasB*, *pqsA*, and *phzB2* was determined by qRT-PCR. Fold change in gene expression at each time point is relative to its expression in LBB at the same time point (value of 1, dotted line). Values represent the means of 3 independent experiments ± SEM. Significance was determined by two-tailed *t-*test and the cut-off of ≥ twofold (dashed line); ns, no significant difference; ****, *P* <0.0001.

Besides albumin, serum contains numerous proteins, peptides, and factors that may influence (negatively or positively) the expression of different PAO1 QS and QS-related genes. At this stage, we were specifically interested in identifying a potential serum factor(s) responsible for the observed positive regulation of PAO1 phenazine genes at later time points of the growth cycle. As a first step of the identification process (due to the complexity of the human serum), we fractionated LBBS using 50-, 30-, and 10-kDa size exclusion columns. As a control, we also fractionated LBB. We analyzed the effect of the different fractions on the expression of the *phzA1-G1* operon (*phz*) using plasmid pMW303, which contains a *phzA1B1C1-lacZ* transcriptional fusion [47]. Compared with unfractionated LBB and its fractions, all the LBBS fractions significantly enhanced *phz* expression at 16 h of PAO1 growth (S4A Fig). However, expression of *phz* in fractions LS>50 and LS<50 was significantly reduced compared to the level observed in unfractionated LBBS (LS-NF) (Fig 11A). In contrast, there was a small, but significant, stepwise increase in *phz* expression with the each of the remaining fractions (LS>30, LS<30, LS>10, and LS<10) over that seen with LS<50 (Fig 11A). The exact cause of these differences in *phz* expression related to these fractions is not known at this time, but there is a correlation between the reduction in the MW cutoff of the fraction and the increase in the expression of *phZ* (S4B Fig). Since we were interested in a protein or other molecule within serum that enhances *phz* expression, we then focused on fraction LS<10, which was the only fraction that significantly enhanced *phz* expression over that seen in LS-NF (Fig 11A), and examined its effect on expression of QS genes using qRT-PCR. As the previously observed enhancement in *phzC1* expression occurred in PAO1 grown in LBBS to 8 h post-inoculation (Fig 7), we conducted the qRT-PCR experiments using LS<10 on PAO1 grown to the same time point. As with LS-NF, the growth of PAO1 in LS<10 significantly enhanced the expression of *phzC1* (Fig 11B). We also examined the effect of LS<10 on the expression of other QS genes. To our surprise, *lasB* expression and *pqsA* expression were reduced rather than enhanced (Fig 11B). These results suggest that human serum factor(s) of <10-kDa MW uniquely enhance the expression of *phzA1-G1*. Whether the originally observed serum-induced enhancement in the expression of other QS and QS related genes is localized to other serum fractions is yet to be determined.

We then conducted preliminary experiments to explore the nature of the factor(s) within LS<10 that enhanced the expression of the *phzA1-G1* operon. Previous studies demonstrated that high temperatures (≥100°C) denature most proteins and renders them non-functional [80,81]. Heat inactivation of LS<10 by boiling for 15 min did not alter the enhancement of *phC1* expression nor change the expression of *lasB* or *pqsA* at 8 h of growth post-inoculation (Fig 11B) or the expression of *phz* at 16 h of growth (S4C Fig). Among different serum components are non-polar lipid factors such as cytokines and hormones [82]. To assess if any of these components contributes to the observed enhancement in *phzA-G1* expression, we treated the LS<10 with charcoal, which has been shown previously to absorb these components from serum [53]. As we saw with heat inactivation, charcoal treatment did not affect the expression of *phz* expression at 16 h of growth post-inoculation (S4C Fig). These results suggest that small peptides, hormones, and cytokines within human serum do not influence the expression of the phenazine genes at later stages of growth of PAO1. Future experiments are needed to determine the nature of the potential factor(s).

The experiments described in Fig 11A and S4C Fig were conducted using PAO1 carrying the *phz* fusion plasmid pMW303. Although the experimental conditions were similar (cultures were grown in the presence of LS<10), the two experiments were not conducted simultaneously on the same day. Therefore, in one experiment (Fig 11A), we detected 9600 units of β-galactosidase activity from *phz* but 6250 units in the other experiment (S4C Fig). We compared the conditions in each experiment in relationship to other conditions within the same experiment. Thus, even though the level of β-galactosidase activity between the two experiments varied, the trend remained the same; fraction LS<10 increased *phz* expression.

### Growth in LBBS altered the production of several PAO1 outer membrane proteins

**Fig 12:**
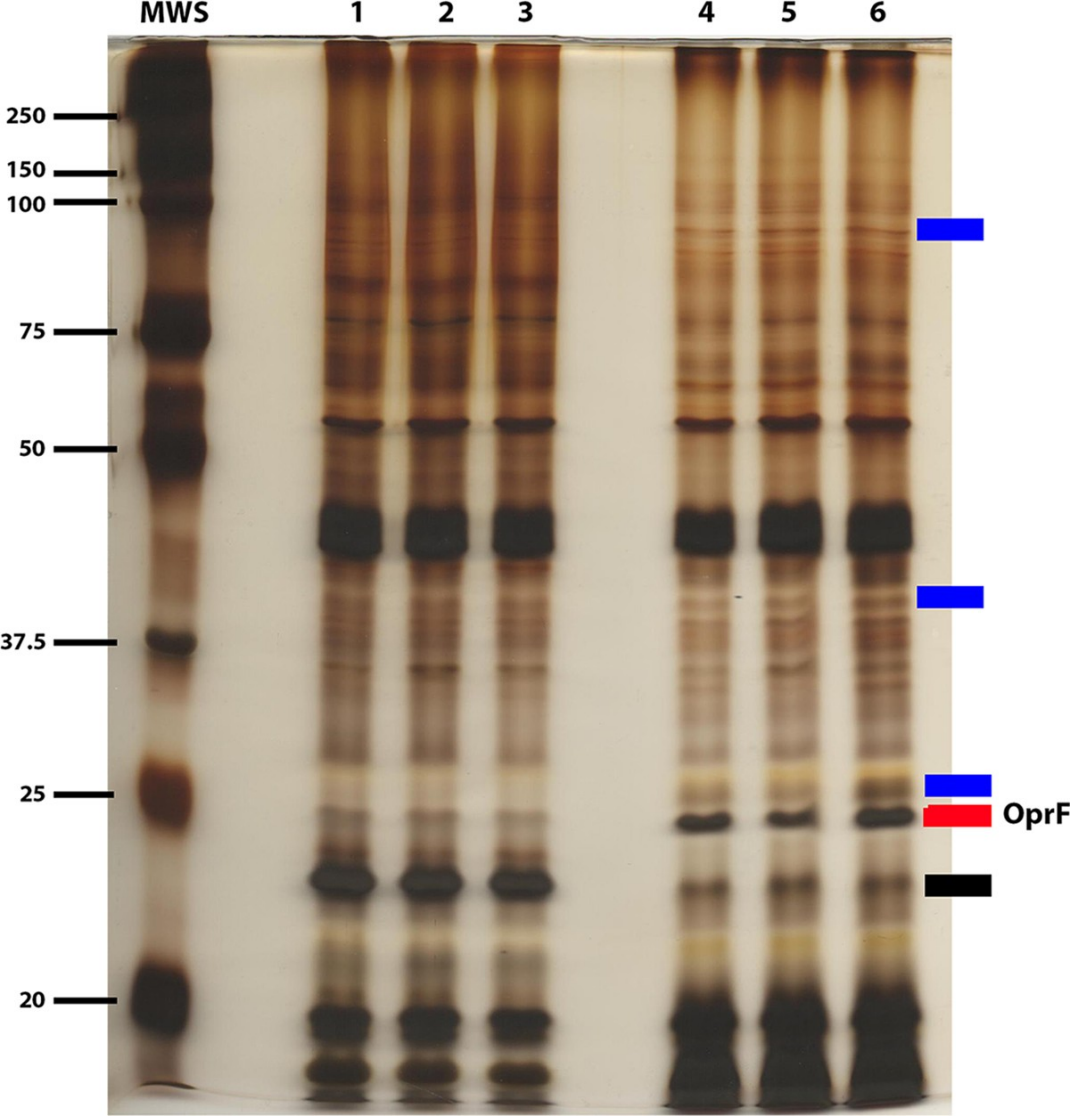
Growth of PAO1 in 10% PHS altered the production of outer membrane proteins. PAO1 was grown in either LBB or LBBS for 16 h post-inoculation. Samples were harvested, lysed, and enriched for OMPs. Samples containing 50 μg of OMPs were separated by SDS-PAGE and stained with silver stain. Lanes: MWS, (molecular mass standards); 1–3, OMPs from PAO1 grown in LBB; 4–6, OMPs from PAO1 grown in LBBS. Blue and red bars, proteins enhanced by growth in LBBS; red bar, OprF; black bar, protein reduced by growth in LBBS.

Factors within serum may affect the expression of different PAO1 genes through one or more outer membrane proteins (OMPs) that function as receptors with which the potential serum factor(s) interacts. Alternatively, factors within serum may influence (positively or negatively) the production of specific PAO1 OMPs. To explore the influence of human serum on PAO1 OMPs, we grew PAO1 for 16 h post-inoculation in both LBB and LBBS. Cells were harvested and the OMPs were extracted and enriched as previously described [54]. Equal amounts of OMPs were then separated by SDS-PAGE and the gel was stained with silver stain. Compared with its growth in LBB, the growth of PAO1 for 16 h post-inoculation in LBBS induced the production of some OMPs (Fig 12, blue and red bars) and reduced the production of others (Fig 12, black bar). We selected a 35-kDa band that appeared to be more strongly enhanced than the others, excised the band, eluted the protein, and determined the amino acid sequence of the protein with LC-MS/MS (Texas Tech University Center for Biotechnology and Genomics, Lubbock, TX). The protein was identified in the initial analysis as OprF (28 peptide match, 70.9% coverage) (Fig 12, red bar). Interestingly, OprF has been shown to affect QS and *P*. *aeruginosa* virulence [83,84]. Additional experiments are underway to determine if OprF plays a role in the induction of QS and QS-related gene expression by serum. We also plan to identify the ~30-kDa protein whose synthesis was significantly reduced in the presence of serum (Fig 12, black bar).

## Discussion

In this study, we demonstrated the influence of WBHVs on the expression of different PAO1 genes. Compared with its growth in LBB, the growth of PAO1 in WBHVs significantly enhanced the expression of 747 genes and significantly reduced the expression of 338 genes (1085 total). As expected, numerous genes involved in metabolism and biosynthesis were affected (Fig 1) (S3 Table). Among these genes, five genes whose products are related to urea metabolism were enhanced (S3A in S3 Table); as were 44 genes related to metabolism and biosynthesis of coenzymes, cofactors and vitamins (S3B in S3 Table); and four genes related to lactate metabolism (S3C in S3 Table). Many additional genes for amino acid (129) and protein (62) metabolism (S3A in S3 Table); carbohydrate metabolism (55) (including gluconeogenesis and glycolysis) and lipid metabolism (59) (S3C in S3 Table); and respiration and energy transfer (74) (S3D in S3 Table) were differentially expressed in PAO1 grown in WBHVs compared to LBB. Changes in expression of these genes reflect the adjustment required of PAO1 in its metabolism/catabolism following its transfer from one environment to another. Numerous genes related to *P*. *aeruginosa* virulence and pathogenesis (S4 Table); cell wall, LPS, and O antigen synthesis (S5 Table); the types VI and II secretion systems (S6 Table); motility, adhesion and chemotaxis (S7 Table); and Mex-related efflux systems related to drug resistance (S8 Table) were also differentially expressed. Strikingly, genes related to all three QS systems were reduced (Figs 2 and 3), while genes of the T3SS (Fig 4) and genes for pyoverdine biosynthesis and uptake (Fig 5) were enhanced.

### WBHV affected all three QS systems, multiple QS-regulated genes, and genes encoding regulators of QS

We previously assessed the influence of adult bovine serum (ABS) on the expression of QS and QS-related genes at two specific time points of growth of PAO1; early and late [48]. Compared to its growth in LBB, the growth of PAO1 in LBB containing 10% ABS significantly repressed the expression of QS and QS-related genes at early stages of growth but significantly enhanced it at late stages of growth [48]. In this study and as an extension of our analysis of the effect of WBHV on the expression of different PAO1 genes, we analyzed the effect of 10% PHS on the expression of QS and QS-related genes at different time points within the growth cycle of PAO1. Similarly, expression of several *las*, *pqs*, and *phz* genes were repressed (>onefold change) by 10% PHS at 2 h (*lasI*, *lasA*, *pqsR*, and *pqsA*) and 4 h (all except *pqsR*) of growth post-inoculation and all genes were significantly induced by 8 h of growth post-inoculation (Fig 7A and 7D). Analysis of expression of these genes at the different time points suggests that the progression from log phase (2 to 6 h post-inoculation) to the entry into stationary phase (by 8 h of growth post-inoculation) (Fig 6), is the critical point at which the genes recover from their serum-repressed state and enter their serum-induced state. The extended growth analysis revealed that a transition in expression from repressed to induced occurs at 6 h post-inoculation for the *las* genes, while the transition was earlier for *pqsR* (between 2 and 4 h) and later for *pqsA* and *phzB2* (between 6 and 8 h) (Fig 7A and 7D). In the presence of 10% PHS and at 6 h post-inoculation, several QS genes were neither repressed nor enhanced (*lasR*, *lasI*, *lasA*, *lasB*, and *phzC1*) (Fig 7A and 7D). The simplest explanation for this observation is that during the progression from log phase to early stationary phase (2 to 8 h), the bacteria enter a state of quorum. As they enter the state of quorum, the bacteria overcame the serum-induced repressing signal of the QS and QS-related genes and responded strongly to the serum-induced enhancing signal. This switching phenomenon is likely limited to the QS genes. Other *P*. *aeruginosa* virulence genes/operons such as the siderophore genes as well as the genes for the T3SS were significantly induced by serum throughout the growth cycle of PAO1 (unpublished observations, C. Kruczek and A. N. Hamood) [48]. The specific mechanism though which these QS genes transit from a serum-repressed to a serum-induced is yet to be determined.

Regulation of the QS systems in *P*. *aeruginosa* is complicated and involves numerous positive and negative regulators [17,85]. Thus, whole blood and serum may differentially regulate the expression of PAO1 QS genes at early and late stages of growth through one or more of these regulators. We previously showed that ABS significantly reduced the expression of the *P*. *aeruginosa* global regulator *vfR* at early stages of growth of PAO1 but enhanced it at late stages of growth [48]. However, in this study, *vfR* expression at 4 h of PAO1 growth post-inoculation was essentially unchanged in WBHV and LBBS (Fig 8A and 8B). Therefore, we searched specifically for a potential positive regulator(s) whose expression is significantly reduced at 4 h of growth in WBHV and in LBBS but significantly induced at 8 h of growth in LBBS. We identified numerous regulators whose expression was decreased at 4 h of growth in WBHV (Fig 8A). However, among several selected regulators, there were only two, *qscR* and *rpo*S, whose expression was also decreased at 4 h of growth in LBBS (Fig 8B). Additionally, of these two genes, the expression of *rpoS* only was enhanced at 8 h of growth in LBBS (Fig 8C). The *qscR* regulatory gene is a less likely possibility, as previous studies showed that the gene counter-regulates *P*. *aeruginosa* QS systems [34,86]. Deletion of *qscR* led to early synthesis of pyocyanin as well as the autoinducers 3OC12-HSL and C4-HSL [86]. The stationary phase sigma factor RpoS positively regulates *lasR* and *rhlR* [47,87]. However, compared with its parent strain, the *rpoS* mutant of PAO1 produced significantly higher levels of pyocyanin [47]. We are currently analyzing the effect of growth in LBBS on the expression of QS and QS regulated genes, including *phzA1-G1* and *phzA2-G2*, at early and later stages of growth of PAO1 and its *rpoS* mutant.

Another possibility is that instead of a single regulator, WBHV or PHS may influence the expression of QS genes through multiple regulators. Thus, for example, the significant increases in the expression of *pvdQ*, *cysB*, *mvaU*, and *mvaT* upon the growth of PAO1 in WBHV (Fig 8A) may reduce the expression of different QS genes. All four genes negatively regulate certain aspects of the QS systems. PvdQ is an acylase that functions as a quorum quencher by degrading 3OC12-HSL and decreasing the production of QS related violence factors [88,89]. CysB represses *pqsR* transcription and PQS production [90] while MvaU and MvaT repress the expression of *lasR*, *pqsR*, and the pyocyanin synthesis operons [85,91,92]. On the other hand, the significant decrease in *vqsM* expression (Fig 8B) may reduce the expression of the QS genes. VqsM is an AraC-type global regulator of QS in *P*. *aeruginosa* [93,94]. The expression of *lasI*, *rhlI*, *rhlR*, *pqsR*, and other QS regulars is suppressed in a *vqsM* mutant [93]. The reduction in the expression of *rsaL*, *qteE*, *cdpR*, *qscR*, and *qsrO* is less likely to be a contributing factor as these genes negatively influence the QS systems [17,85,86,94–98].

We previously showed that, compared with its growth in WBHV, the growth of *P*. *aeruginosa* in WB from severely burned patients significantly enhanced the expression of TTSS, pyochelin, and pyoverdine genes compared to the level of their expression in WBHV, but significantly repressed the expression of QS and QS-related virulence genes [37]. Comparing the results of our current study with that of Kruczek et al. leads us to the conclusion that the expression of the above genes and operons is influenced by two potential signals; one within the WBHV and the other due to the changes in WB induced by severe burn injury. However, such a conclusion is not possible at this time since the studies were conducted using two different *P*. *aeruginosa strains*; this study was conducted using PAO1 while that of Kruczek et al. [37] was conducted using strain PA14. Our recent preliminary analysis of gene expression of PA14 that was grown in LBB and LBBS supports this argument. Regulation of certain virulence genes in PA14 in response to PHS is different from their regulation in PAO1 (unpublished observations, K. L. Beasley and A. N. Hamood). Thus, a comprehensive analysis of the PA14 transcriptome during its growth in WBHV is essential to identify different *P*. *aeruginosa* genes whose expression is the same in both strains.

### Growth of PAO1 in HSA at 10% physiological level affected expression of the QS genes

One major component of serum that may influence the expression of PAO1 QS genes at early and/or late stages of growth is albumin. Albumin is one of the largest components within human serum, with normal physiological levels ranging from 35–50 g/L [79]. Identification of structural similarities between bovine serum albumin (BSA) ligands and the QS molecules led to the suggestion that BSA influences the QS systems [78]. It was further shown that BSA reduced the expression of multiple QS and QS-related genes throughout the growth cycle of *P*. *aeruginosa*, suggesting that BSA interferes with QS activities by binding to and sequestering *P*. *aeruginosa* QS signaling molecules [78]. Therefore, we examined the effect of human serum albumin (HSA) on the expression of PAO1 QS genes. As expected, HSA at physiological levels found in 10% PHS repressed the expression of *lasB*, *pqsA*, and *phzB2* at both 4 h and 12 h of growth of PAO1 post-inoculation although this repression was significantly less for *lasB* and *phzB2* than that produced by 10% PHS (Fig 10A). Additionally, HSA-10%-pl repressed pyocyanin production throughout the growth cycle of PAO1, even at 72 h post-inoculation (Fig 10B). It is likely that, similar to BSA, HSA affects the QS genes during the log and stationary phases of PAO1 by binding directly to QS signaling molecules. Using the modeling program Molecular Operating Environment (https://www.chemcomp.com/Products.htm), we illustrated specific sites within the HSA molecule where *P*. *aeruginosa* PQS is predicted to interact (S4 Fig). The contrast between the effect of PHS and HSA on the expression of different QS genes at 12 h of growth post-inoculation (Fig 10A) suggests that serum components other than albumin significantly induced the expression of QS genes. The effect of these potential components appeared to be strong enough to overcome the negative effect of HSA (Figs 7 and 10).

Fractionation experiments using columns with different MWCO to identify the serum component(s) that influence the expression of QS and QS-related virulence genes at either 8 h or 16 h of growth post-inoculation suggested the presence of more than one potential serum factor that affected expression of the *phz* genes. Compared to the LBB controls, all fractions significantly enhanced *phz* expression (S4A Fig). Compared to nonfractionated LBBS (LS-NF), the fraction of LBBS containing proteins <50-kDa (LS<50) significantly reduced *phz* expression while the fraction containing <10-kDa proteins (LS<10) significantly enhanced *phz* expression (Fig 11A). The presence of more than one serum factor is not surprising as serum is complicated and contains numerous proteins and molecules [82]. What was surprising, however, was the apparent restriction of the effect of the LS<10 fraction to the *phzA1-G1* operon only. Unlike the induction of *phzC1*, a gene within the *phzA1-G1* operon, the LS<10 fraction repressed the expression of *pqsA* (and possibly the entire *pqsA-E* operon) as well as *lasB* at 16 h of growth (Fig 11B). Compared with the extensive analysis of different components of the *P*. *aeruginosa* QS systems, available information regarding the regulation of the *phzA1-G1* and *phzA2-G2* operons is limited. Experimental data, as well as sequence analyses, suggest potential roles for LasR, RhlR, and PqsR in the regulation of the operons. Identification of a lux-box-like sequence within the upstream region of *phzA1*, indicated a potential role for regulation of *phzA1-G1* by either LasR or RhlR (or both) [35]. Additionally, deletion of the *pqsR* gene resulted in a severe reduction of pyocyanin levels, suggesting PqsR is necessary for full expression of the *phz* operons [26]. Our results suggest that the component(s) within fraction LS<10 responsible for induction of the *phzA1-G1* operon does not work through the typical hierarchical *las* or *pqs* QS systems. To explore the nature of the potential serum factor(s) within the LS<10, we used heat inactivation and charcoal treatment and determined that neither treatment eliminated the ability of LS<10 to induce *phz* expression (S4C Fig). Besides the factor(s) within LS<10, the nature of the potential factor(s) within the other serum fractions and how they regulate QS genes is yet to be determined.

Besides determining the serum factor that regulates *phzA1-G1* and the QS systems, it is important to determine the bacterial mechanism through which this regulation occurs. We hypothesized that a serum component may manipulate the QS system by initially interacting with a potential outer membrane protein (OMP). Through enhancement or reduction of the synthesis of such a protein, thus, providing a sufficient or a limited amount of receptor with which the serum factor can interact, the function of the QS systems may either be augmented or limited. Analysis of OMPs from PAO1 that was grown for 16 h post-inoculation in LBBS supported this hypothesis. PHS at 10% increased the synthesis of certain OMPs while reducing the synthesis of others (Fig 12). Among the OMPs whose synthesis was considerably increased in the presence of 10% PHS is OprF (Fig 12). Fito-Boncompte *et al*. [83] previously showed that OprF is important for *P*. *aeruginosa* virulence as it influences the production of different virulence factors. A *P*. *aeruginosa oprF* deletion mutant was compromised in its adhesion to eukaryotic cells as well as in its ability to produce T3SS toxins and QS-controlled virulence factors including LasB, pyocyanin, and lectin PA-1L [83]. Further, the mutation reduced 3OC12-HSL synthesis, delayed C4-HSL synthesis, and interfered with PQS secretion [83]. Compared with its parent strain, the level of PQS within the supernatant of the *oprF* deletion mutant was significantly reduced while the intracellular level of the PQS precursor HHQ was significantly increased [83]. Our data also suggest the possibility that PHS affects the QS systems through OprF. The delay in the production/release of pyocyanin (Fig 7D) could be due to lower levels of OprF resulting in failure of the conversion of HHQ to PQS at earlier stages of *P*. *aeruginosa* growth despite the enhanced expression of *pqsA-E* by early stationary phase (Fig 7C). Further analyses, including the utilization of PAOΔ*oprF*, are essential to delineate the role of OprF in the observed effect of serum.

An additional role for OprF in the response of *P*. *aeruginosa* to the host immune response was reported by Wu *et al*. [99] who showed that *P*. *aeruginosa* alters its virulence in response to interferon-gamma (IFN-γ). Only IFN-γ (but not other tested cytokines) significantly increased the expression of PA-1L lectin as well as pyocyanin production by *P*. *aeruginosa* [99]. This effect, which required functional *rhlI* and *rhlR*, was growth phase dependent; it was observed at early stationary but not late log phase of growth [99]. Further analysis showed that IFN-γ specifically binds to OprF [99]. More importantly, Wu *et al*. [99] provided evidence that IFN-γ influences the *P*. *aeruginosa* QS systems through OprF, as IFN-γ failed to enhance PA-1L expression in a *P*. *aeruginosa* OprF deletion mutant. Based on these results, Wu *et al*. [99] suggested that *P*. *aeruginosa* has evolved a contingency-based mechanism to mount an effective countermeasure to immune activation by the host. Our observed changes in *pqsR* expression (at 4 h) and *rhlI/R* expression (at 8 h) followed by a later increase in OprF synthesis (at 16 h) concomitantly with the enhanced release of pyocyanin (from 24 h to 72 h) supports the potential role for the OprF in the *P*. *aeruginosa* response to the host’s immune response. Since we detected the enhancement in the *phz* genes within LS-NF as well as all the LS fractions (compared to LBB), the potential factor may reside in any of the treated fractions. In this regard, during bacteremia, and as part of its strategy of maintaining a countermeasure to the host response, *P*. *aeruginosa* would augment OprF production in response to a signal generated by a potential serum factor. Increased OprF would allow sufficient protein for interaction with IFN-γ, which may be increased above baseline levels during Gram-negative bacteremia [100]. This interaction would lead to an increase in the expression of QS and QS-related genes. Alternatively, OprF has been shown to bind to serum amyloid A, an acute phase protein elevated during the innate immune response [101], and to the complement component C3b produced by activation of complement on the surface of the bacterium [102]. Although the pathway(s) subsequently activated within *P*. *aeruginosa* have not been determined, OprF binding with either or both of these proteins may be the mechanism by which *P*. *aeruginosa* orchestrates part of its response to the host.

### Growth of PAO1 in WBHVs affected T3SS genes

Our results suggest that at 4 h of growth, whole blood regulates PAO1 T3SS genes at the transcriptional level (Fig 4). This regulation probably occurs through *exsA*, the master regulator of T3SS. The product of *exsA*, ExsA, upregulates expression of the genes within all four T3SS operons, the genes encoding the exoenzyme effector proteins, and their specific chaperones [103]. This regulation includes the additional T3SS regulatory genes *exsD* (anti-activator), *exsC* (anti-anti-activator), and *exsE* (secreted/translocated ExsE, partner of ExsC and ExsD) [104] as expression of all of these genes was enhanced (Fig 4). Among the different signals that regulate the expression of different *P*. *aeruginosa* T3SS genes are calcium levels within the surrounding medium and bacterial contact with target eukaryotic cells via the type IV pilus [105]. These signals enhance *exsA* transcription through the global regulator Vfr [104]. However, this is less likely to be the mechanism through which WBHV upregulates the expression of the T3SS genes as the expression of *vfr* in WBHV or in LBBS was essentially unchanged (< onefold change) (Fig 8). Another possible mechanism through which WBHV may upregulate the expression of T3SS genes is through its negative effect on the expression of QS genes, specifically *rhlI*, at early stages of growth of PAO1 (Fig 2). However, this is also less likely to be the mechanism since *rhlI* negatively regulates the expression of the T3SS genes under low Ca^2+^ conditions, and this regulation does not include the T3SS main regulatory gene *exsA* [106]. A third possibility is that WBHV may influence the T3SS by significantly reducing the level of the QS autoinducer molecule PQS, which is produced by the *pqs* QS system. However, such a scenario would occur through the secretion of T3SS cytotoxins rather than the expression of the T3SS genes. Singh *et al*. [107] previously suggested that the *P*. *aeruginosa* QS autoinducer molecule PQS regulates secretion of the T3SS cytotoxins post-translationally by direct or indirect inhibition. Although increased levels of PQS had no effect on the amount of synthesized T3SS cytotoxins, it inhibited their secretion [107].

With respect to the potential blood component that influences the expression of the T3SS genes, serum albumin may be the potential contributing factor. It has been previously suggested that, through its calcium-binding capacity, serum albumin enhances the release of intracellularly accumulated effectors of the T3SS but does not enhance the expression of T3SS genes directly [108]. Such an effect is thought to occur through the PopN outer membrane protein component of the T3SS [108]. However, through the enhanced release of intracellularly accumulated T3SS proteins, serum albumin may indirectly enhance the expression of T3SS genes by influencing the interaction between the anti-activator (ExsD) and the anti-anti-activator (ExsC) [104]. Under conditions in which the T3SS is repressed, ExsE binds ExsC leaving the anti-activator ExsD to bind to ExsA, the transcriptional activator of *exsA*, thus reducing the expression of T3SS genes[104]. Secretion of ExsE (possibly through PopN) triggered by serum albumin releases ExsC, which then binds ExsD; ExsD releases ExsA, allowing it to transactivate expression of *exsA* and the other T3SS genes [104]. Further studies are required to explore this possibility. Regardless of the potential mechanism through which blood regulates the PAO1 T3SS genes, the enhancement of expression in 32 of 40 T3SS genes in blood from three different healthy volunteers suggests that the T3SS plays a critical role in the pathogenesis of *P*. *aeruginosa* infection at early stages of bacteremia.

### Growth of PAO1 in WBHV and 10% PHS differentially regulated the expression of the siderophore genes

Iron has been shown to repress the expression of different *P*. *aeruginosa* iron acquisition genes; therefore, the growth of *P*. *aeruginosa* in iron-limited conditions enhances the expression of these genes [11]. Compared with its growth in LBB, the growth of PAO1 in WBHV significantly enhanced the expression of genes for pyoverdine synthesis, uptake, and release of iron from ferripyoverdine, as well as the gene encoding the ferripyochelin receptor FptA (Fig 5). Additionally, expression of the genes encoding the hemin degrading factor PhuS, the heme acquisition protein HasAP, and bacterioferritin plus genes for the uptake of ferric enterobactin (*fep* genes, *fbvA* and *tonB1*) was enhanced (Fig 5). Within human blood, free iron is limited by its sequestration within the plasma protein transferrin [109]. Thus, the iron-limited environment within the blood may have induced expression of the PAO1 iron-repressed genes. Our analysis using 10% PHS supported this possibility. The expression of the pyoverdine synthesis gene *pvdA* was significantly enhanced at 2, 4, 6 and 8 h post-inoculation in PAO1 grown in LBBS compared to its expression in PAO1 grown in LBB (Fig 9A). We previously showed that the addition of ABS to the iron-deficient medium trypticase soy broth dialysate (TSB-DC) significantly enhanced the expression of numerous iron acquisition genes [67]. Further analysis revealed that the expression of two main transcriptional regulators of iron acquisition genes, *pvdS* and *toxR*, was significantly increased by the addition of the serum iron-binding protein apotransferrin to TSB-DC [67]. We also provided evidence suggesting that serum albumin enhances the expression of iron-regulated genes through a mechanism independent of iron acquisition as absorption of albumin from either ABS or PHS eliminated the enhancement in *pvdS* and *toxR* expression [67]. In the present study, serum iron-binding proteins may bind iron within LBB and convert the medium (LBBS) into an iron-deficient environment. Alternatively, albumin within PHS may enhance the expression of the siderophore genes through an iron-independent mechanism as we previously demonstrated [67].

In contrast to the enhancement in pyoverdine synthesis gene expression, growth of PAO1 for 4 h in whole blood repressed the expression of genes within the pyochelin synthesis operons *pchDCBA* and *pchEFGHI* (Fig 5). Such an effect is limited to WBHV. The expression of *pchA* (and likely the entire *pchDCBA* operon) was induced by PHS at 2, 4, and 6 h post-inoculation in LBBS, but unchanged at 8 h (Fig 9B). Although these results are limited at this time, they raise several questions that need to be answered by detailed future analyses. First, is the reduction in the expression of the pyochelin genes due to a component(s) of whole blood such as RBC or WBC that counteracts the induction of these genes by serum? Second, why is this effect of WBHV unique to pyochelin genes? The expression of both the pyochelin and pyoverdine genes in *P*. *aeruginosa* is stringently controlled by the iron-activated ferric uptake regulator Fur [65,110]. Finally, is the effect of WBHV limited to 4 h of growth post-inoculation or does it occur throughout the growth cycle of PAO1?

### WB influenced gene expression in *P*. *aeruginosa* and other bacterial pathogens

Previous studies described the influence of whole blood on gene expression in different pathogenic bacteria [111–116], who examined *Bacillus anthracis*, *Bordetella pertussis*, *Streptococcus pyogenes*, *Staphylococcus aureus*, *Streptococcus agalactiae*, and *Enterococcus faecalis*, respectively. The type of blood used ranged from bovine [111] to horse [112] to sheep [116] to human [113–115]. Additionally, the expression of the genes of the target organism were examined at radically different times, most ranging from time of inoculation (time 0) to 120 min [112–116] to as long as 4 h [111]. The closest of those studies to ours is that of Carlson *et al*. [111] who analyzed the expression of *B*. *anthracis* genes by a low inoculum of the bacteria in whole blood for 4 h at 37°C. Despite the differences, our study and all above described studies (except the one by Graham *et al*. [113]) revealed the upregulation of iron acquisition and uptake genes, particularly those related to siderophores and heme (Fig 5). In addition, and similar to Vebo *et al*. (2009) who reported differential expression of virulence factors, we found that QS-related virulence factor genes were downregulated, while others such as pyoverdine, the T3SS effector toxins, and QS-independent hemolysins were upregulated. Furthermore, results of our study and that of Carlson *et al*. (2015), whose experimental conditions were similar to ours, revealed that whole blood differentially regulated the expression of 62 amino acid transport and biosynthesis genes, with 37 upregulated and 25 downregulated. Similarly, we found that, of 157 genes related to amino acid metabolism/biosynthesis and transport, 123 were upregulated and 34 were downregulated (S1 and S2 Tables). Additionally, in both studies, expression of the phenazine genes (Fig 2), genes coding for penicillin-binding proteins, and the gene encoding alkaline phosphatase were downregulated (S4 Table) [111].

**Fig 13:**
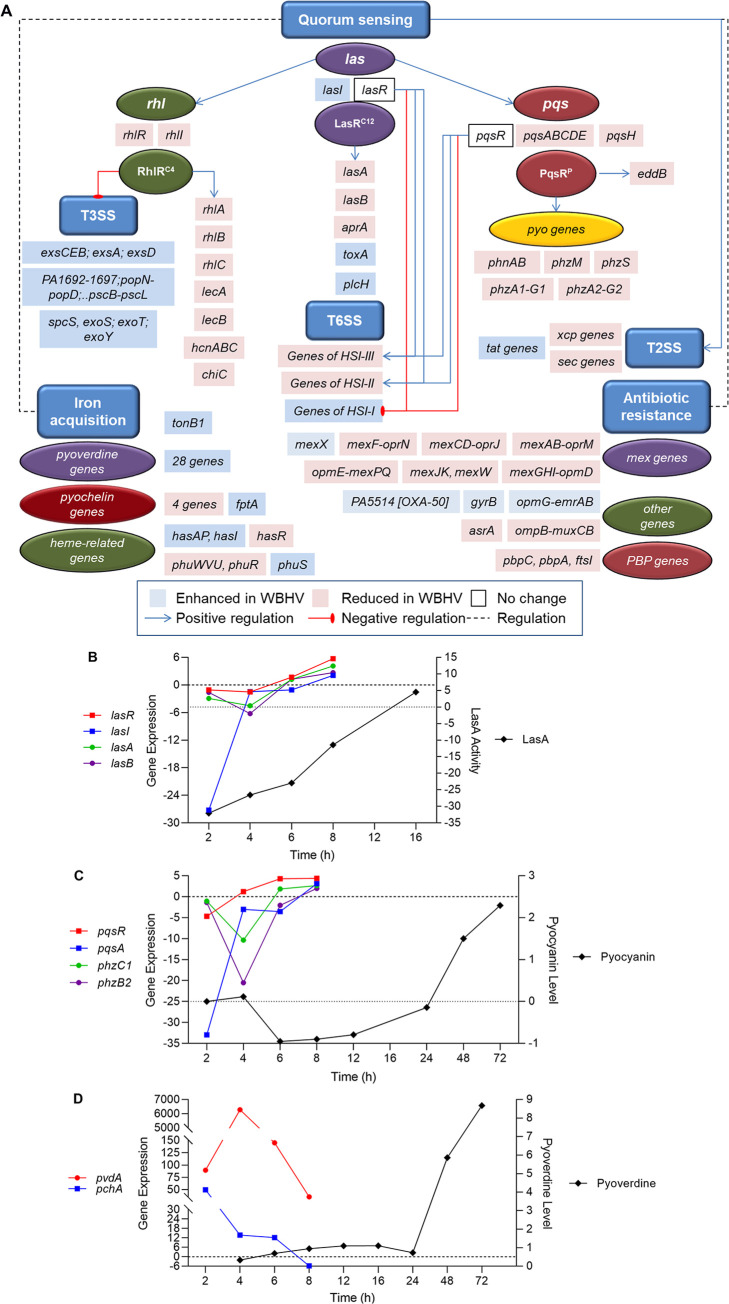
Summary diagrams of the QS and QS-related genes and systems affected by growth of *P*. *aeruginosa* in WBHV or PHS. (A) Growth in WBHV for 4 h affected expression of genes of the *las*, *rhl*, and *pqs* QS systems and their related virulence genes as well as genes of the T2SS, T3SS, and T6SS, genes for iron acquisition, and genes for antibiotic resistance. See embedded legend for description of symbols used in figure. HSI-I, HSI-II, HSI-III: Hcp secretion islands I, II, and III, respectively. (B, C, D) Growth in PHS revealed temporal changes in genes expression and production of virulence factors. Gene expression (left *y-*axis, 0 indicated by dashed line) is shown as fold change (WBHV to LBB). The related virulence factor (right *y-*axis, 0 indicated by dotted line) is shown as change in production in WBHV over (or under) the production of that factor in LBBS. (B) Expression of genes of the *las* system and LasA activity. (C) Expression of genes of the *pqs* system and pyocyanin production. (D) Expression of genes for pyoverdine and pyochelin synthesis and pyoverdine production.

In summary, the growth of *P*. *aeruginosa* in WB and in the presence of PHS significantly impacts its pathogenesis. Within a few hours of its growth in WBHV, *P*. *aeruginosa* adopts a unique strategy that involves the differential expression of numerous genes including virulence and virulence-related genes (Fig 13A). While *P*. *aeruginosa* reduced the expression of multiple QS and QS-related genes, it significantly enhanced the expression of most of the genes involved in the T3SS (Fig 13A). Another unique aspect of this adaptation is the differential regulation of numerous iron-acquisition genes. While the growth of *P*. *aeruginosa* in WB (an iron-restricted environment) induced the expression of the pyoverdine operon, it repressed the expression of genes involved in pyochelin synthesis (Fig 13A). The intricate mechanism of this differential regulation in iron-scavenging gene expression is yet to be determined.

Similar to its strategy upon its growth in WB, *P*. *aeruginosa* repressed the expression of several QS and QS-related genes upon its growth in the presence of PHS at early stages of growth (2–4 h) (Fig 13B and 13C). In contrast, at later stages of growth (6–8 h) and in the presence of PHS, *P*. *aeruginosa* significantly induced the expression of the same QS and QS-related genes (Fig 13B). Concomitantly with the increase in expression of the QS genes at later stages of growth, we also observed an increase in the production of LasA and pyocyanin at late time points, further into the growth cycle than observed with gene expression (16 h for LasA to 48–72 h for pyocyanin) (Fig 13B and 13C). Our evidence suggests that the induction of these genes at late stages of growth is a complicated phenomenon that involves serum components of variable molecular weights (Fig 11). In contrast to the observed decrease in expression of pyochelin genes in WBHV, expression of *pchA* was increased early in the growth cycle (2–6 h) and decreased by 8 h (Fig 13D). Similar to the expression of the pyoverdine genes in WBHV, expression of *pvdA* was increased at all tested time points (Fig 13D). As observed with pyocyanin production, pyoverdine was produced at the later stages of growth (48–72 h) (Fig 13D).

## Conclusions

The present study provides a reference point of comparison for future studies focused on *P*. *aeruginosa* bacteremia induced by different immunocompromising conditions including diabetes and cancer. Our results strongly suggest that the influence of whole blood and/or serum from healthy volunteers on the expression of different *P*. *aeruginosa* genes is complex, comprehensive, and variable. While the expression of the T3SS operons and genes was induced at the 4 h time point (Fig 4) and several operons/genes for iron acquisition were induced throughout the growth cycle of *P*. *aeruginosa* (Figs 5 and 9, S2 Fig), that of others including QS and QS-related virulence genes was variable; repressed during early stages but enhanced at later stages of growth (Figs 2, 7 and 8). The study also suggests that several potential serum components manipulate the expression of *P*. *aeruginosa* genes (Figs 10 and 11). Finally, the study suggests that outer membrane proteins represent the link through which whole blood or serum influences the expression of *P*. *aeruginosa* virulence genes (Fig 12).

## Supporting information

S1 Fig PAO1 expression of *lasI* and *pqsA* was inconsistent in WBHVs by RNA-Seq, but consistent by qRT-PCR. The level of expression of the indicated genes was determined by qRT-PCR using the same RNA samples as templates. Only two of the three HV samples were sufficient for parallel testing, those from HV1 and HV2. PAO1 gene expression at 4 h post-inoculation in WBHV is relative to its expression in LBB at the same time point; dotted lines indicate onefold level of expression. Values represent the means of 3 sets of 3 replicates on 2 independent samples; bar indicates median.
(PDF)

S2 Fig Growth of PAO1 in the presence of 10% PHS enhanced the expression of the pyoverdine synthesis gene *pvdD* at 4 to 16 h post-inoculation. PAO1/pMP190::*pvdD-lacZ* (transcriptional fusion) was grown in LBB or LBBS and samples were collected every 2 h from 4–12 h and at 16 h. Cell pellets were collected and lysed, and β-galactosidase activity within the lysates was determined. Values represent the means of 3 independent experiments ± SEM. Significance was determined by two-tailed *t-*test; ***, *P* < 0.01; ****, *P* <0.0001.
(PDF)

S3 Fig Growth of PAO1 in the presence of HSA at 10% physiological level (LBBA) paralleled its growth in LBB. PAO1 was inoculated at OD_600_ ~0.020 into LBB or LBBA and incubated with shaking at 200 RPM to the time points indicated on the graph and the OD_600_, representative of the growth index, was determined. Data were log-transformed before graphing. Values represent the means of 3 independent experiments ± SEM. One-way ANOVA comparing pairs of time points revealed no significant differences between growth in LBB and LBBA.
(PDF)

S4 Fig Different fractions of LBBS enhanced *phzA1B1C1* expression. (A) Both LBB and LBBS were fractionated using molecular weight cut-off columns of 50-, 30-, and 10-kDa. PAO1/pMW303, which carries a *phzA1B1C1-lacZ* transcriptional reporter fusion, was grown for 16 h post-inoculation in nonfractionated LBB (LB-NF), fractionated LBB (LB<30, LB<10), nonfractionated LBBS (LS-NF) and/or fractionated LBBS (LS>50, LS<50, LS>30, LS<30, LS>10 and LS<10). Cell pellets were collected and lysed, and β-galactosidase activity within the lysates was determined. Values represent the means of 3 independent experiments ± SEM. Significance was determined by one-way ANOVA with Dunnett’s multiple comparisons posttest using LB-NF, LB<30, and LB<10 as controls (dashed lines) or LS-NF as the control (solid lines); *, *P* <0.05; ****, *P* <0.0001. (B) Simple linear regression analysis of the LS fractions versus their β-galactosidase activity. There is a linear relationship between the values; as *x* increases, *y* decreases. Dotted lines represent the 95% confidence interval for the regression line. (C) Inactivation treatments did not alter *phzA1B1C1* expression. LS<10 was subjected to heat inactivation (LS<10-HI) by boiling for 15 min or charcoal treatment (LS<10-CT). PAO1/pMW303 was grown for 16 h post-inoculation in LS<10, LS<10-CT, or LS<10-HI. Cell pellets were collected and lysed, and β-galactosidase activity within the lysates was determined. Values represent the means of 3 independent experiments ± SEM. No significant differences were found by two-tailed *t-*test.
(PDF)

S5 Fig Diagram depicting potential PQS autoinducer binding sites to human serum albumin (HSA). Potential binding sites for HSA within the PQS autoinducer were determined using the modeling software Molecular Operating Environment (MOE) version 2019.01 [117] available at http://www.chemcomp.com. The crystal structure for HSA (PDB ID 1AO6) [118] was downloaded from the Protein Data Bank [119] available at http://www.rcsb.org/pdb.
(PDF)

S6 Fig Original gel silver stained. (TIF)

S7 Fig Original silver stained gel. (TIF)

S1 Table PAO1 genes significantly upregulated or downregulated by growth in WBHVs compared to growth in LBB. (PDF)

S2 Table PAO1 genes whose expression was inconsistent following growth in WBHVs compared to growth in LBB. (PDF)

S3 Table PAO1 genes for metabolic and biosynthetic processes that were up- or downregulated by growth in WBHVs compared to growth in LBB. A. Nitrogen metabolism: amino acids, proteins, urea, and heterocyclic compounds. B. Sulfur metabolism and coenzyme, cofactor, and vitamin metabolism and biosynthesis. C. Carbon metabolism: glucose, other carbohydrates, tricarboxylic acid cycle, fatty acids and lipids, and carbon (general). D. Energy metabolism, respiration, and stress responses.
(PDF)

S4 Table PAO1 genes encoding virulence factors upregulated or downregulated by growth in WBHVs compared to growth in LBB. (PDF)

S5 Table PAO1 genes for cell wall/LPS/O antigen synthesis upregulated or downregulated by growth in WBHVs compared to growth in LBB. (PDF)

S6 Table PAO1 genes of the type VI and type II secretion systems upregulated or downregulated by growth in WBHVs compared to growth in LBB. (PDF)

S7 Table PAO1 genes related to motility, chemotaxis, and adhesion that were upregulated or downregulated by growth in WBHVs compared to growth in LBB. (PDF)

S8 Table PAO1 *mex* genes related to multidrug efflux were predominantly downregulated by growth in WBHVs compared to growth in LBB. (PDF)
