# Supplementary material for: During bacteremia, Pseudomonas aeruginosa PAO1 adapts by altering the expression of numerous virulence genes including those involved in quorum sensing
Source: PLoS One. 2020 Oct 15;15(10):e0240351. doi: 10.1371/journal.pone.0240351 (PMC7561203; doi:10.1371/journal.pone.0240351)
Supplement: S1 Fig — The level of expression of the indicated genes was determined by qRT-PCR using the same RNA samples as templates. Only two of the three HV samples were sufficient for parallel testing, those from HV1 and HV2. PAO1 gene expression at 4 h post-inoculation in WBHV is relative to its expression in LBB at the same time point; dotted lines indicate onefold level of expression. Values represent the means of 3 sets of 3 replicates on 2 independent samples; bar indicates median. (PDF) [file pone.0240351.s001.pdf]

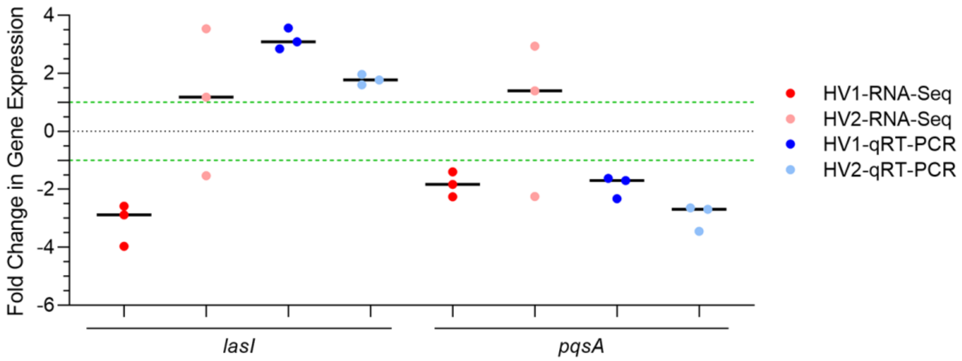

S1 Fig PAO1 expression of *lasI* and *pqsA* was inconsistent in WBHVs by RNA-Seq, but consistent by qRT-PCR. The level of expression of the indicated genes was determined by qRT-PCR using the same RNA samples as templates. Only two of the three HV samples were sufficient for parallel testing, those from HV1 and HV2. PAO1 gene expression at 4 h post-inoculation in WBHV is relative to its expression in LBB at the same time point; dotted lines indicate onefold level of expression. Values represent the means of 3 replicates on 2 independent samples ± SEM.
