# Supplementary material for: During bacteremia, Pseudomonas aeruginosa PAO1 adapts by altering the expression of numerous virulence genes including those involved in quorum sensing
Source: PLoS One. 2020 Oct 15;15(10):e0240351. doi: 10.1371/journal.pone.0240351 (PMC7561203; doi:10.1371/journal.pone.0240351)
Supplement: S2 Fig — PAO1/pMP190::pvdD-lacZ (transcriptional fusion) was grown in LBB or LBBS and samples were collected every 2 h from 4–12 h and at 16 h. Cell pellets were collected and lysed, and β-galactosidase activity within the lysates was determined. Values represent the means of 3 independent experiments ± SEM. Significance was determined by two-tailed t-test; ***, P < 0.01; ****, P <0.0001. (PDF) [file pone.0240351.s002.pdf]

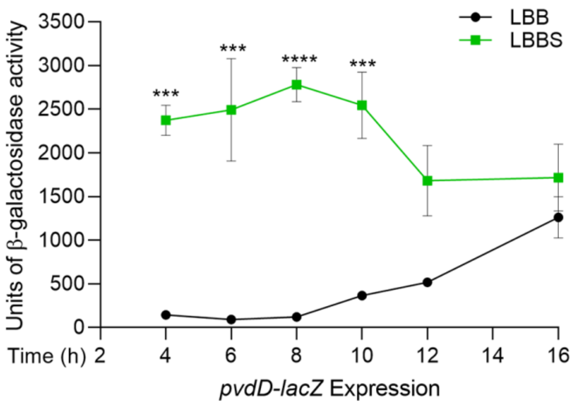

S2 Fig Growth of PAO1 in the presence of 10% PHS enhanced the expression of the pyoverdine synthesis gene *pvdD* at 4 to 16 h post-inoculation. PAO1/pMP190::*pvdD-lacZ* (transcriptional fusion) was grown in LBB or LBBS and samples were collected every 2 h from 4-12 h and at 16 h. Cell pellets were collected and lysed, and β-galactosidase activity within the lysates was determined. Values represent the means of 3 independent experiments ± SEM. Significance was determined by two-tailed *t*-test; ***, $P < 0.01$; ****, $P < 0.0001$.
