# Supplementary material for: During bacteremia, Pseudomonas aeruginosa PAO1 adapts by altering the expression of numerous virulence genes including those involved in quorum sensing
Source: PLoS One. 2020 Oct 15;15(10):e0240351. doi: 10.1371/journal.pone.0240351 (PMC7561203; doi:10.1371/journal.pone.0240351)
Supplement: S3 Fig — PAO1 was inoculated at OD600 ~0.020 into LBB or LBBA and incubated with shaking at 200 RPM to the time points indicated on the graph and the OD600, representative of the growth index, was determined. Data were log-transformed before graphing. Values represent the means of 3 independent experiments ± SEM. One-way ANOVA comparing pairs of time points revealed no significant differences between growth in LBB and LBBA. (PDF) [file pone.0240351.s003.pdf]

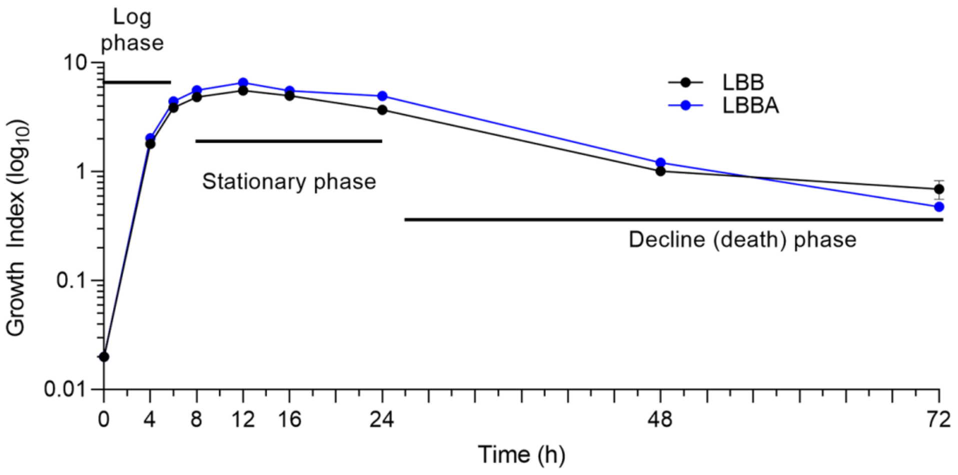

S3 Fig Growth of PAO1 in the presence of HSA at 10% physiological level (LBBA) paralleled its growth in LBB. PAO1 was inoculated at OD_{600} ~0.020 into LBB or LBBA and incubated with shaking at 200 RPM to the time points indicated on the graph and the OD_{600}, representative of the growth index, was determined. Data were log-transformed before graphing. Values represent the means of 3 independent experiments ± SEM. One-way ANOVA comparing pairs of time points revealed no significant differences between growth in LBB and LBBA.
