# Supplementary material for: During bacteremia, Pseudomonas aeruginosa PAO1 adapts by altering the expression of numerous virulence genes including those involved in quorum sensing
Source: PLoS One. 2020 Oct 15;15(10):e0240351. doi: 10.1371/journal.pone.0240351 (PMC7561203; doi:10.1371/journal.pone.0240351)
Supplement: S4 Fig — (A) Both LBB and LBBS were fractionated using molecular weight cut-off columns of 50-, 30-, and 10-kDa. PAO1/pMW303, which carries a phzA1B1C1-lacZ transcriptional reporter fusion, was grown for 16 h post-inoculation in nonfractionated LBB (LB-NF), fractionated LBB (LB<30, LB<10), nonfractionated LBBS (LS-NF) and/or fractionated LBBS (LS>50, LS<50, LS>30, LS<30, LS>10 and LS<10). Cell pellets were collected and lysed, and β-galactosidase activity within the lysates was determined. Values represent the means of 3 independent experiments ± SEM. Significance was determined by one-way ANOVA with Dunnett’s multiple comparisons posttest using LB-NF, LB<30, and LB<10 as controls (dashed lines) or LS-NF as the control (solid lines); *, P <0.05; ****, P <0.0001. (B) Simple linear regression analysis of the LS fractions versus their β-galactosidase activity. There is a linear relationship between the values; as x increases, y decreases. Dotted lines represent the 95% confidence interval for the regression line. (C) Inactivation treatments did not alter phzA1B1C1 expression. LS<10 was subjected to heat inactivation (LS<10-HI) by boiling for 15 min or charcoal treatment (LS<10-CT). PAO1/pMW303 was grown for 16 h post-inoculation in LS<10, LS<10-CT, or LS<10-HI. Cell pellets were collected and lysed, and β-galactosidase activity within the lysates was determined. Values represent the means of 3 independent experiments ± SEM. No significant differences were found by two-tailed t-test. (PDF) [file pone.0240351.s004.pdf]

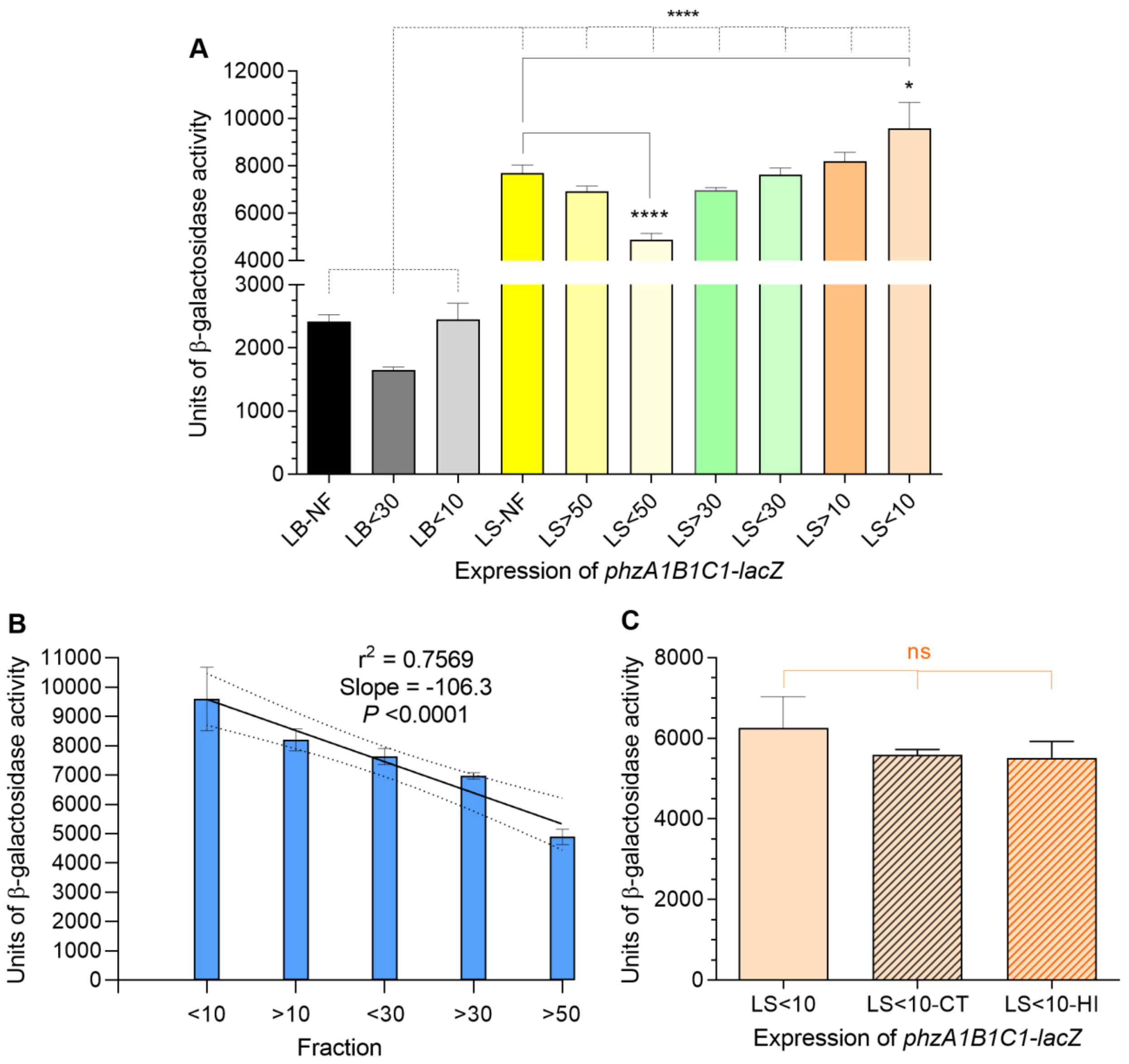

S4 Fig Different fractions of LBBS enhanced *phzA1B1C1* expression. (A) Both LBB and LBBS were fractionated using molecular weight cut-off columns of 50-, 30-, and 10-kDa. PAO1/pMW303, which carries a *phzA1B1C1-lacZ* transcriptional reporter fusion, was grown for 16 h post-inoculation in nonfractionated LBB (LB-NF), fractionated LBB (LB<30, LB<10), nonfractionated LBBS (LS-NF) and/or fractionated LBBS (LS>50, LS<50, LS>30, LS<30, LS>10 and LS<10). Cell pellets were collected and lysed, and β-galactosidase activity within the lysates was determined. Values represent the means of 3 independent experiments ± SEM. Significance was determined by one-way ANOVA with Dunnett's multiple comparisons posttest using LB-NF, LB<30, and LB<10 as controls (dashed lines) or LS-NF as the control (solid lines); *, *P* <0.05; ****, *P* <0.0001. (B) Simple linear regression analysis of the LS fractions versus their β-galactosidase activity. There is a linear relationship between the values; as *x* increases, *y* decreases. Dotted lines represent the 95% confidence interval for the regression line. (C)

Inactivation treatments did not alter *phzA1B1C1* expression. LS<10 was subjected to heat inactivation (LS<10-HI) by boiling for 15 min or charcoal treatment (LS<10-CT). PAO1/pMW303 was grown for 16 h post-inoculation in LS<10, LS<10-CT, or LS<10-HI. Cell pellets were collected and lysed, and β-galactosidase activity within the lysates was determined. Values represent the means of 3 independent experiments ± SEM. No significant differences were found by two-tailed *t*-test.
