# Supplementary material for: During bacteremia, Pseudomonas aeruginosa PAO1 adapts by altering the expression of numerous virulence genes including those involved in quorum sensing
Source: PLoS One. 2020 Oct 15;15(10):e0240351. doi: 10.1371/journal.pone.0240351 (PMC7561203; doi:10.1371/journal.pone.0240351)
Supplement: S5 Fig — Potential binding sites for HSA within the PQS autoinducer were determined using the modeling software Molecular Operating Environment (MOE) version 2019.01 [117] available at http://www.chemcomp.com. The crystal structure for HSA (PDB ID 1AO6) [118] was downloaded from the Protein Data Bank [119] available at http://www.rcsb.org/pdb. (PDF) [file pone.0240351.s005.pdf]

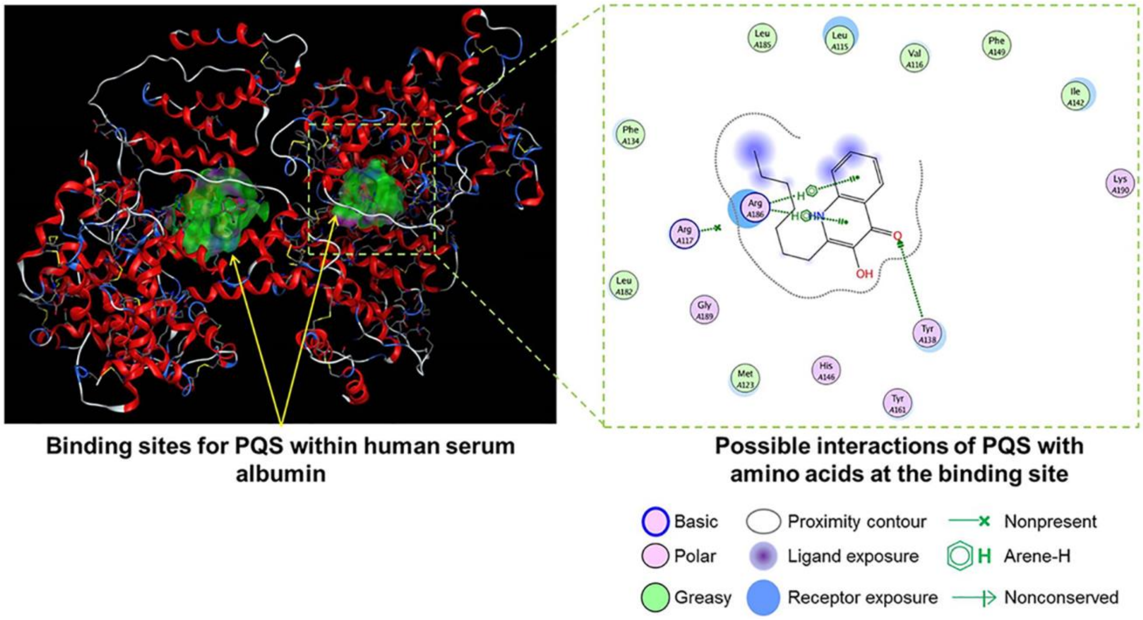

S5 Fig. Diagram depicting potential PQS autoinducer binding sites to human serum albumin (HSA). Potential binding sites for HSA within the PQS autoinducer were determined using the modeling software Molecular Operating Environment (MOE) version 2019.01 [1] available at http://www.chemcomp.com. The crystal structure for HSA (PDB ID 1AO6) [2] was downloaded from the Protein Data Bank [3] available at http://www.rcsb.org/pdb.

References

1. MOE (The Molecular Operating Environment). 2019.01 ed. Montreal, Canada: Chemical Computing Group; 2019.
2. Sugio S, Kashima A, Mochizuki S, Noda M, Kobayashi K. Crystal structure of human serum albumin at 2.5 A resolution. Protein Eng. 1999 Jun;12(6):439-46. https://doi.org/10.1093/protein/12.6.439. PubMed PMID: 10388840. Epub 1999/07/02.
3. Berman H, Henrick K, Nakamura H. Announcing the worldwide Protein Data Bank. Nat Struct Biol. 2003 Dec;10(12):980. https://doi.org/10.1038/nsb1203-980. PubMed PMID: 14634627. Epub 2003/11/25.
