# Supplementary figures and images for: During bacteremia, Pseudomonas aeruginosa PAO1 adapts by altering the expression of numerous virulence genes including those involved in quorum sensing
Source: PLoS One. 2020 Oct 15;15(10):e0240351. doi: 10.1371/journal.pone.0240351 (PMC7561203; doi:10.1371/journal.pone.0240351)

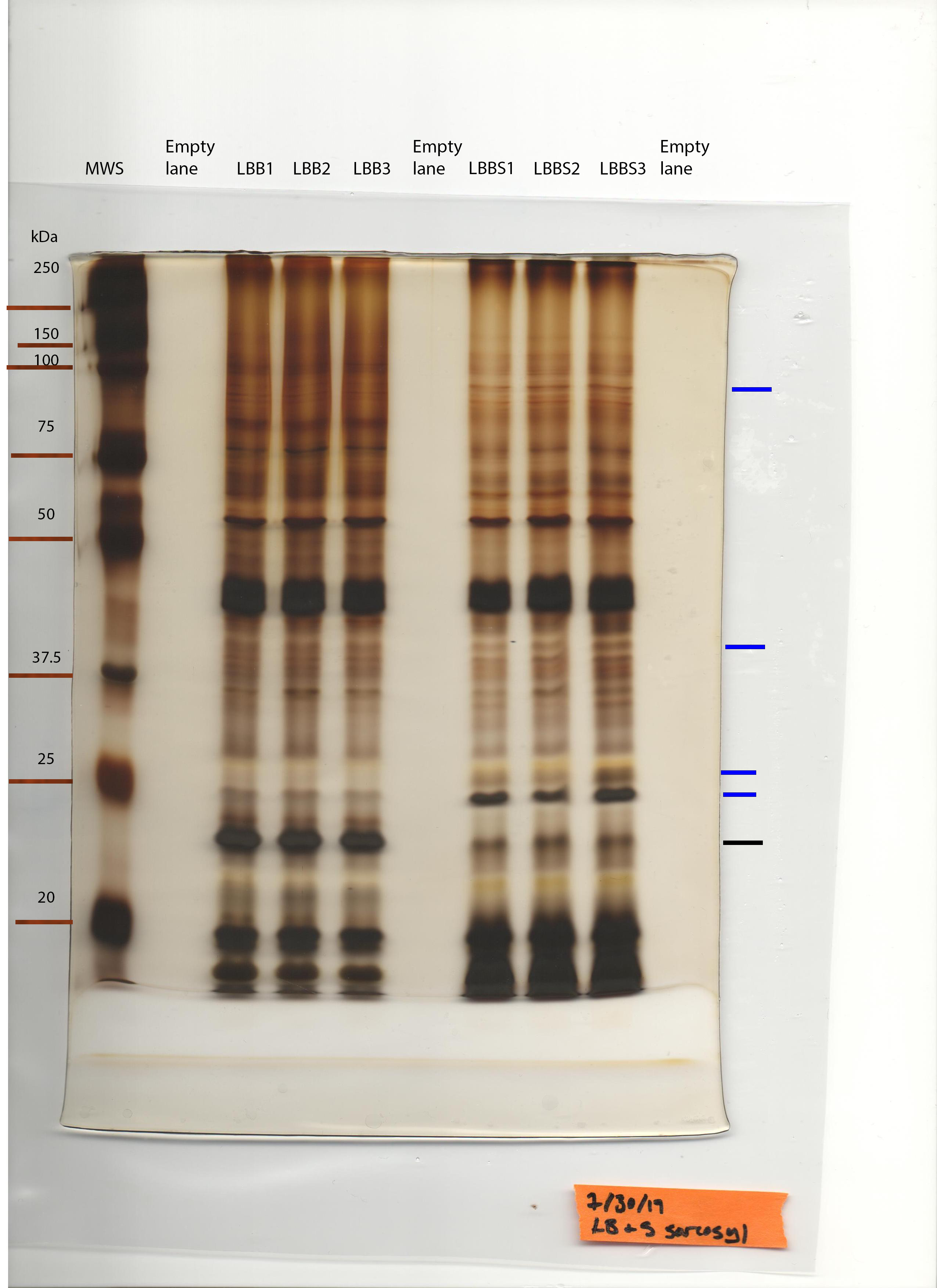

Supplement: S6 Fig — (TIF) [file pone.0240351.s006.tif]

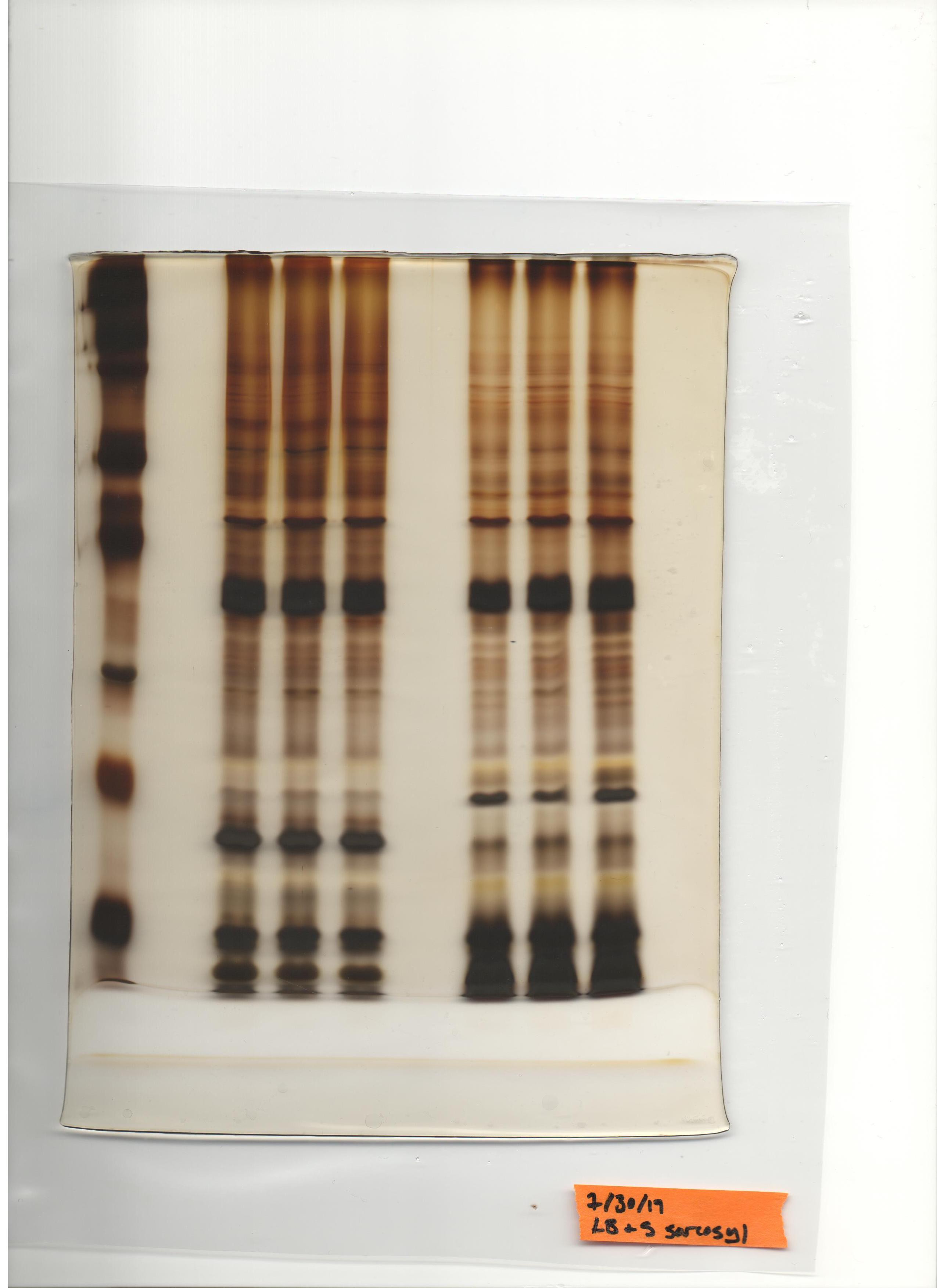

Supplement: S7 Fig — (TIF) [file pone.0240351.s007.tif]
