# Supplementary material for: During bacteremia, Pseudomonas aeruginosa PAO1 adapts by altering the expression of numerous virulence genes including those involved in quorum sensing
Source: PLoS One. 2020 Oct 15;15(10):e0240351. doi: 10.1371/journal.pone.0240351 (PMC7561203; doi:10.1371/journal.pone.0240351)
Supplement: S1 Table — (PDF) [file pone.0240351.s008.pdf]

S1 Table. PAO1 genes significantly upregulated or downregulated by growth in WBHVs compared to growth in LBB

Gene Number*	Gene Name*	Product*	Relative Expression WBHV:LBB			*q* Value			Fold Change WBHV:LBB			
			HV1	HV2	HV3	LBB:HV1	LBB:HV2	LBB:HV3	HV1	HV2	HV3	AVERAGE
PA0004	*gyrB*	DNA gyrase subunit B	2.9409	2.1355	1.5493	1.44E-126	1.57E-59	7.94E-28	2.941	2.135	1.549	2.21
PA0007	-	Hypothetical protein	0.1000	0.1250	0.3875	6.48E-14	2.52E-13	4.57E-08	-10.000	-8.000	-2.581	-6.86
PA0018	*fmt*	Methionyl-tRNA formyltransferase	1.6176	1.7647	3.0294	6.17E-07	4.96E-08	4.37E-24	1.618	1.765	3.029	2.14
PA0020	*tsaP*	T4P secretin-associated protein TsaP	4.8313	3.3813	1.2000	1.46E-215	2.40E-92	6.74E-06	4.831	3.381	1.200	3.14
PA0029	-	Probable sulfate transporter	0.0769	0.1538	0.3077	1.03E-09	5.88E-03	1.80E-10	-13.000	-6.500	-3.250	-7.58
PA0035	*trpA*	Tryptophan synthase alpha chain {subunit alpha}[†]	2.2623	2.4918	5.4262	1.63E-18	1.55E-22	6.05E-140	2.262	2.492	5.426	3.39
PA0036	*trpB*	Tryptophan synthase beta chain {subunit beta}	6.6000	5.8833	5.9333	2.28E-298	2.88E-226	2.62E-230	6.600	5.883	5.933	6.14
PA0043	-	Hypothetical protein	2.9091	6.4545	4.5455	6.32E-14	5.28E-87	7.44E-36	2.909	6.455	4.545	4.64
PA0044	*exoT*	Exoenzyme T	21.6222	23.8667	19.8444	0.00E+00	0.00E+00	0.00E+00	21.622	23.867	19.844	21.78
PA0045	-	Hypothetical protein	11.2381	7.0476	3.6667	0.00E+00	1.01E-111	3.85E-22	11.238	7.048	3.667	7.32
PA0046	-	Hypothetical protein	2.3448	1.7586	3.7241	3.12E-07	2.96E-04	3.50E-17	2.345	1.759	3.724	2.61
PA0048		Probable transcriptional regulator	0.1333	0.0571	0.2143	1.66E-07	1.00E-41	4.25E-36	-7.500	-17.500	-4.667	-9.89
PA0049	-	Hypothetical protein	0.6531	0.7906	0.0594	2.73E-04	6.77E-05	0.00E+00	-1.531	-1.265	-16.842	-6.55
PA0050	-	Hypothetical protein	0.1106	0.0363	0.0819	4.37E-13	4.32E-151	0.00E+00	-9.038	-27.535	-12.206	-16.26
PA0055	-	Hypothetical protein	6.2212	4.2596	1.3173	6.32E-207	4.08E-84	2.16E-03	6.221	4.260	1.317	3.93
PA0070	*tagQ1*	T6SS TagQ1	2.1706	1.5249	2.8845	4.96E-44	3.53E-19	1.61E-84	2.171	1.525	2.885	2.19
PA0071	*tagR1*	T6SS TagR1	2.5400	5.3600	2.3200	2.90E-34	7.05E-197	8.47E-25	2.540	5.360	2.320	3.41
PA0074	*ppkA [tagE1]* [‡]	T6SS serine/threonine protein kinase PpkA [TagE1][‡]	1.0645	3.1613	2.3226	6.44E-06	1.10E-62	7.45E-28	1.065	3.161	2.323	2.18
PA0075	*pppA [tagG1]*	T6SS PppA protein [TagG1] {serine/threonine phosphatase}	4.2500	3.3056	3.1389	6.26E-51	1.21E-28	3.75E-23	4.250	3.306	3.139	3.56
PA0077	*icmF1 [tssM1]*	T6SS IcmF protein [TssM1]	2.1277	1.2128	4.0213	2.66E-30	7.01E-08	1.30E-131	2.128	1.213	4.021	2.45
PA0078	*tssL1*	T6SS TssL1	4.9048	1.8810	4.5952	8.52E-126	2.23E-13	6.15E-106	4.905	1.881	4.595	3.79
PA0079	*tssK1*	T6SS TssK1	9.4898	4.0000	6.2449	0.00E+00	1.18E-84	1.78E-241	9.490	4.000	6.245	6.58
PA0080	*tssJ1*	T6SS TssJ1	3.9375	3.4063	6.3594	1.22E-46	2.41E-33	1.42E-142	3.938	3.406	6.359	4.57
PA0081	*fha1*	T6SS Fha domain-containing protein	8.0625	8.4688	4.5313	0.00E+00	0.00E+00	1.91E-85	8.063	8.469	4.531	7.02
PA0082	*tssA1*	T6SS TssA1	21.9583	10.7083	17.1250	0.00E+00	0.00E+00	0.00E+00	21.958	10.708	17.125	16.60
PA0083	*tssB1*	T6SS TssB1	24.5083	15.9417	9.5958	0.00E+00	0.00E+00	0.00E+00	24.508	15.942	9.596	16.68
PA0084	*tssC1*	T6SS TssC1	9.8168	3.6040	9.6881	0.00E+00	1.96E-142	0.00E+00	9.817	3.604	9.688	7.70
PA0086	*tagJ1 [hsiE1]*	T6SS TagJ1	12.1190	5.4048	8.6667	0.00E+00	1.11E-110	0.00E+00	12.119	5.405	8.667	8.73
PA0087	*tssE1*	T6SS TssE1	24.6400	9.2400	12.8400	0.00E+00	3.54E-192	0.00E+00	24.640	9.240	12.840	15.57
PA0088	*tssF1*	T6SS TssF1	6.0286	3.0571	6.2857	3.04E-224	3.40E-45	6.50E-247	6.029	3.057	6.286	5.12
PA0089	*tssG1*	T6SS TssG1	2.0000	2.6667	5.5833	2.82E-09	6.99E-17	3.79E-91	2.000	2.667	5.583	3.42
PA0090	*clpV1*	T6SS protein ClpV1	1.8929	2.6071	4.1786	1.06E-23	3.90E-49	3.98E-146	1.893	2.607	4.179	2.89
PA0093	*tse6*	T6SS Tse6	5.2609	3.5652	4.4348	6.26E-94	1.75E-37	1.99E-60	5.261	3.565	4.435	4.42
PA0094	*eagT6*	T6SS EagT6	30.8000	26.5333	8.8000	0.00E+00	0.00E+00	2.13E-167	30.800	26.533	8.800	22.04
PA0095	*vgrG1b*	VgrG1b	3.6038	2.7736	3.9434	3.41E-95	1.09E-51	1.42E-116	3.604	2.774	3.943	3.44
PA0096	-	Hypothetical protein	2.8947	1.3947	5.7368	8.27E-16	1.32E-03	3.69E-73	2.895	1.395	5.737	3.34
PA0099	-	Type VI effector protein	1.4255	1.4894	3.0851	1.73E-07	1.12E-07	5.79E-38	1.426	1.489	3.085	2.00
PA0109	-	Hypothetical protein	0.0739	0.1080	0.5568	5.52E-22	2.61E-09	7.34E-03	-13.538	-9.263	-1.796	-8.20
PA0119	-	Probable C4-dicarboxylate transporter	4.4545	3.5455	6.9091	5.15E-35	5.86E-21	1.63E-96	4.455	3.545	6.909	4.97
PA0120	-	Probable transcriptional regulator	3.5385	5.1538	2.0769	1.65E-33	1.20E-80	1.21E-07	3.538	5.154	2.077	3.59
PA0122	*rahU*	RahU {hypothetical protein}	0.0290	0.1702	0.0245	0.00E+00	4.64E-16	0.00E+00	-34.514	-5.875	-40.738	-27.04
PA0123	-	Probable transcriptional regulator	0.0966	0.1739	0.0628	4.04E-18	2.62E-08	0.00E+00	-10.350	-5.750	-15.923	-10.67
PA0127	-	Hypothetical protein	2.9143	1.8857	2.7714	4.37E-16	2.08E-06	3.06E-12	2.914	1.886	2.771	2.52
PA0129	*bauD {gabP}*	Amino acid permease {gamma-aminobutyrate permease}	4.0000	8.6818	13.5909	3.73E-51	0.00E+00	0.00E+00	4.000	8.682	13.591	8.76
PA0130	*bauC*	3-Oxopropanoate dehydrogenase	4.8542	4.0000	23.7500	1.57E-140	1.95E-89	0.00E+00	4.854	4.000	23.750	10.87
PA0131	*bauB*	BauB {beta-alanine degradation protein}	18.9167	17.2917	29.1250	0.00E+00	0.00E+00	0.00E+00	18.917	17.292	29.125	21.78
PA0132	*bauA [oapT]*	Beta-alanine:pyruvate transaminase [omega-amino acid--pyruvate transaminase]	18.8800	13.2000	40.8400	0.00E+00	0.00E+00	0.00E+00	18.880	13.200	40.840	24.31

PA0136	-	Probable ATP-binding component of ABC transporter	3.0000	3.0000	7.0000	1.23E-02	9.52E-03	2.04E-11	3.000	3.000	7.000	4.33
PA0141	-	Conserved hypothetical protein	2.2719	2.1475	1.7972	1.28E-51	3.01E-45	9.04E-28	2.272	2.147	1.797	2.07
PA0144	-	Hypothetical protein	0.0909	0.0404	0.1919	6.26E-16	7.09E-56	1.57E-37	-11.000	-24.750	-5.211	-13.65
PA0146	-	Conserved hypothetical protein	1.6000	2.8000	2.2000	1.60E-02	4.62E-06	1.87E-02	1.600	2.800	2.200	2.20
PA0155	*pcaR*	Transcriptional regulator PcaR	7.1333	4.8667	3.1000	7.00E-173	2.61E-70	6.70E-22	7.133	4.867	3.100	5.03
PA0165	-	Hypothetical protein	3.7500	2.8750	3.1250	1.21E-13	1.81E-07	2.01E-07	3.750	2.875	3.125	3.25
PA0167	-	Probable transcriptional regulator	2.2308	1.8718	1.9231	8.18E-12	5.14E-08	4.30E-06	2.231	1.872	1.923	2.01
PA0174	-	Conserved hypothetical protein	0.0682	0.2045	0.2500	1.78E-17	2.31E-02	2.57E-16	-14.667	-4.889	-4.000	-7.85
PA0177	-	Probable purine-binding chemotaxis protein	0.0276	0.1353	0.1754	3.27E-282	1.91E-14	2.27E-76	-36.273	-7.389	-5.700	-16.45
PA0178	-	Probable two-component sensor [probable chemotaxis protein sensory transduction histidine kinase]	0.0206	0.0782	0.1358	0.00E+00	4.13E-68	6.83E-132	-48.600	-12.789	-7.364	-22.92
PA0179	-	Probable two-component response regulator [probable chemotaxis response regulator]	0.0479	0.1129	0.1277	1.88E-99	2.01E-28	2.32E-172	-20.881	-8.859	-7.830	-12.52
PA0201	-	Hypothetical protein	2.4659	2.0568	7.2727	2.17E-23	4.05E-15	1.56E-296	2.466	2.057	7.273	3.93
PA0211	*mdcD*	Malonate decarboxylase beta subunit	1.4353	1.0353	7.9059	3.91E-09	4.73E-04	0.00E+00	1.435	1.035	7.906	3.46
PA0217	*[mdcR]*	Probable transcriptional regulator	1.8750	1.5625	4.7500	7.99E-06	3.49E-04	9.64E-40	1.875	1.563	4.750	2.73
PA0235	*pcaK*	4-Hydroxybenzoate transporter PcaK	2.0000	2.5000	2.5000	3.70E-02	6.97E-04	2.56E-02	2.000	2.500	2.500	2.33
PA0236	-	Probable transcriptional regulator	0.1176	0.1176	0.1765	3.16E-08	1.71E-03	2.96E-25	-8.500	-8.500	-5.667	-7.56
PA0262	*vgrG2b*	T6SS VgrG2b	3.6429	1.7857	2.7857	1.82E-47	2.46E-09	1.34E-22	3.643	1.786	2.786	2.74
PA0263	*hcpC*	T6SS secreted protein Hcp	8.7692	28.0000	3.9231	5.44E-105	0.00E+00	1.58E-14	8.769	28.000	3.923	13.56
PA0263.1	-	tRNA-Arg	1.6667	2.3333	4.6667	1.93E-01	5.06E-02	1.23E-05	1.667	2.333	4.667	2.89
PA0265	*davD [gabD]*	Glutaric semialdehyde dehydrogenase [succinate-semialdehyde dehydrogenase]	10.1269	7.8192	4.8308	0.00E+00	0.00E+00	6.64E-301	10.127	7.819	4.831	7.59
PA0266	*davT [gabT]*	Delta-aminobutyrate aminotransferase [4-aminobutyrate aminotransferase, GabA transaminase]	6.6456	4.9409	3.0570	0.00E+00	0.00E+00	3.12E-128	6.646	4.941	3.057	4.88
PA0267	-	Hypothetical protein	0.1714	0.1143	0.4857	4.05E-03	1.18E-08	4.46E-04	-5.833	-8.750	-2.059	-5.55
PA0270	-	Hypothetical protein	0.0699	0.0420	0.2238	1.40E-27	4.02E-83	3.23E-28	-14.300	-23.833	-4.469	-14.20
PA0277	-	Conserved hypothetical protein	3.8000	4.5000	6.8000	2.47E-15	4.17E-23	1.15E-55	3.800	4.500	6.800	5.03
PA0291	*oprE*	Anaerobically-induced outer membrane porin OprE [Porin E1]	13.3543	8.6800	5.4743	0.00E+00	0.00E+00	0.00E+00	13.354	8.680	5.474	9.17
PA0292	*aguA*	Agmatine deiminase [agmatine iminohydrolase]	4.8451	6.2817	1.5634	2.67E-152	7.61E-281	3.30E-08	4.845	6.282	1.563	4.23
PA0295	-	Probable periplasmic polyamine binding protein	7.3684	11.5000	2.9474	1.37E-251	0.00E+00	1.04E-26	7.368	11.500	2.947	7.27
PA0296	*spuI [pau1]*	Glutamylpolyamine synthetase	3.2582	3.7992	6.6270	2.42E-112	2.73E-162	0.00E+00	3.258	3.799	6.627	4.56
PA0297	*spuA [ycjL, pauD1]*	Probable glutamine amidotransferase	3.0000	4.8387	6.9570	2.63E-45	2.04E-139	0.00E+00	3.000	4.839	6.957	4.93
PA0298	*spuB [pauA2]*	Glutamylpolyamine synthetase	6.1628	8.9225	8.5504	0.00E+00	0.00E+00	0.00E+00	6.163	8.922	8.550	7.88
PA0299	*spuC*	Polyamine:pyruvate transaminase	5.4336	4.0000	5.4196	0.00E+00	2.69E-197	0.00E+00	5.434	4.000	5.420	4.95
PA0300	*spuD [potF2]*	Polyamine transporter protein {putrescine ABC transporter substrate-binding protein SpuD}	2.7414	3.5799	2.9765	1.00E-101	3.92E-192	4.30E-123	2.741	3.580	2.976	3.10
PA0301	*spuE [potF3]*	Polyamine transporter protein {spermidine ABC transporter substrate-binding protein SpuE}	8.7757	7.2056	3.8692	0.00E+00	0.00E+00	1.46E-109	8.776	7.206	3.869	6.62
PA0302	*spuF [potG]*	Polyamine transporter PotG	3.0135	3.8649	2.7905	1.51E-70	1.33E-126	4.07E-57	3.014	3.865	2.791	3.22
PA0307	-	Hypothetical protein	0.0488	0.1220	0.3171	8.50E-49	7.08E-05	3.19E-09	-20.500	-8.200	-3.154	-10.62
PA0335	-	Hypothetical protein	3.2821	1.3590	1.4872	2.75E-28	2.74E-04	7.86E-03	3.282	1.359	1.487	2.04
PA0353	*ilvD*	Dihydroxy-acid dehydratase	3.5556	2.7475	2.2828	9.04E-104	1.04E-56	4.23E-35	3.556	2.747	2.283	2.86
PA0358	-	Hypothetical protein	1.7222	2.2778	2.0556	5.48E-04	1.35E-06	1.23E-03	1.722	2.278	2.056	2.02
PA0359	-	Hypothetical protein	4.7198	4.7637	2.3901	3.60E-122	8.09E-125	3.17E-22	4.720	4.764	2.390	3.96
PA0372	-	Probable zinc protease	7.4400	10.1600	1.4200	0.00E+00	0.00E+00	8.27E-06	7.440	10.160	1.420	6.34
PA0385	-	Hypothetical protein	5.1579	3.6316	1.8421	1.41E-28	5.12E-13	3.82E-02	5.158	3.632	1.842	3.54
PA0395	*pilT*	Twitching motility protein PilT [type 4 fimbrial biogenesis protein PilT]	4.8017	4.5776	1.3190	2.14E-186	2.59E-166	1.53E-06	4.802	4.578	1.319	3.57
PA0397	*[czcD]*	Probable cation efflux system protein	2.5385	1.5128	2.1795	4.66E-19	4.84E-06	3.01E-11	2.538	1.513	2.179	2.08
PA0402	*pyrB*	Aspartate carbamoyltransferase [aspartate transcarbamoylase]	3.2804	4.4766	2.8598	1.38E-72	3.15E-152	6.54E-51	3.280	4.477	2.860	3.54

PA0403	*pyrR*	Transcriptional regulator PyrR [uracil phosphoribosyltransferase PyrR]	13.5902	20.6721	4.4754	0.00E+00	0.00E+00	1.86E-62	13.590	20.672	4.475	12.91
PA0404	*[yqgF]*	Conserved hypothetical protein {Holliday junction resolvase}	3.2444	6.7333	3.5333	1.90E-22	4.14E-124	8.00E-25	3.244	6.733	3.533	4.50
PA0405	*algH [yqgE]*	AlgH	1.9398	4.2169	3.3373	6.72E-13	9.02E-76	2.52E-41	1.940	4.217	3.337	3.16
PA0407	*gshB*	Glutathione synthetase [GSH-II]	2.9389	3.9924	1.9847	1.06E-59	3.85E-123	1.04E-21	2.939	3.992	1.985	2.97
PA0409	*pilH*	Twitching motility protein PilH [type 4 fimbrial biogenesis protein PilH]	3.9644	4.5668	2.1454	1.65E-120	1.05E-169	1.90E-26	3.964	4.567	2.145	3.56
PA0420	*bioA*	Adenosylmethionine-8-amino-7-oxononanoate aminotransferase [7,8-diamino-pelargonic acid aminotransferase DAPA aminotransferase]	3.8462	3.0923	1.0308	1.56E-95	4.16E-57	2.77E-02	3.846	3.092	1.031	2.66
PA0423	*pasP [yceI]*	PasP {hypothetical protein}	0.5899	0.1427	0.6990	5.73E-04	1.06E-26	2.00E-02	-1.695	-7.008	-1.431	-3.38
PA0428	*[rhlE]*	Probable ATP-dependent RNA helicase	0.1053	0.1871	0.3099	6.77E-14	3.87E-09	2.55E-13	-9.500	-5.344	-3.226	-6.02
PA0457	-	Hypothetical protein	3.2381	1.4762	2.9048	4.67E-15	2.37E-03	5.46E-10	3.238	1.476	2.905	2.54
PA0463	*creB*	Two-component response regulator CreB	2.1852	2.8148	1.9259	3.88E-09	1.26E-15	1.42E-04	2.185	2.815	1.926	2.31
PA0482	*glcB [aceB]*	Malate synthase G	5.3062	6.5787	1.8146	0.00E+00	0.00E+00	2.72E-38	5.306	6.579	1.815	4.57
PA0486	*[yihE]*	Conserved hypothetical protein {serine/threonine protein kinase}	2.6923	2.0769	2.6154	4.91E-17	9.75E-10	1.20E-13	2.692	2.077	2.615	2.46
PA0491	-	Probable transcriptional regulator	1.9286	2.7857	1.5714	7.31E-06	7.31E-13	2.43E-02	1.929	2.786	1.571	2.10
PA0500	*bioB*	Biotin synthase [biotin synthetase]	14.4276	9.9145	3.1776	0.00E+00	0.00E+00	2.05E-77	14.428	9.914	3.178	9.17
PA0501	*bioF*	8-amino-7-oxononanoate synthase [7-KAP synthetase, L-alanine--pimelyl CoA ligase, 7-keto-8-amino-pelargonic acid synthetase]	6.7083	4.7917	3.2917	1.39E-157	1.50E-71	3.79E-27	6.708	4.792	3.292	4.93
PA0506	-	Probable acyl-CoA dehydrogenase	20.6064	16.7872	1.9894	0.00E+00	0.00E+00	6.69E-25	20.606	16.787	1.989	13.13
PA0508	-	Probable acyl-CoA dehydrogenase	7.3824	6.2647	1.7941	0.00E+00	1.07E-233	6.41E-10	7.382	6.265	1.794	5.15
PA0528	-	Probable transcriptional regulator	1.4000	2.2000	4.0000	4.76E-02	5.30E-04	4.33E-10	1.400	2.200	4.000	2.53
PA0529	-	Conserved hypothetical protein	1.6129	2.6452	5.3548	1.76E-05	5.92E-15	3.63E-73	1.613	2.645	5.355	3.20
PA0530	-	Probable class III pyridoxal phosphate-dependent	1.1765	2.0588	7.5294	3.36E-03	1.81E-08	1.63E-156	1.176	2.059	7.529	3.59
PA0541	-	Hypothetical protein	0.1413	0.0435	0.3315	9.07E-07	1.16E-66	6.60E-13	-7.077	-23.000	-3.016	-11.03
PA0547	-	Probable transcriptional regulator	0.0195	0.0423	0.1596	0.00E+00	3.21E-152	1.09E-97	-51.167	-23.615	-6.265	-27.02
PA0562	-	Probable hydrolase	4.1200	2.0800	2.8800	4.18E-34	9.59E-08	4.72E-13	4.120	2.080	2.880	3.03
PA0563	-	Conserved hypothetical protein	16.5696	8.3797	22.2658	0.00E+00	4.46E-268	0.00E+00	16.570	8.380	22.266	15.74
PA0578	-	Conserved hypothetical protein	2.6154	4.8077	1.8269	1.07E-15	3.61E-63	8.37E-05	2.615	4.808	1.827	3.08
PA0589	*[glpE]*	Conserved hypothetical protein {thiosulfate sulfurtransferase}	2.5714	2.9286	2.5893	4.94E-13	2.10E-17	5.50E-11	2.571	2.929	2.589	2.70
PA0592	*ksgA*	rRNA (adenine-N6,N6)-dimethyltransferase	6.5556	5.9259	1.9630	4.24E-121	1.82E-95	2.55E-05	6.556	5.926	1.963	4.81
PA0599	-	Hypothetical protein	2.5143	3.6857	1.3143	2.25E-19	2.85E-46	1.80E-02	2.514	3.686	1.314	2.50
PA0603	*agtA*	AgtA {ABC transporter ATP-binding protein}	4.6087	3.4565	8.0435	6.87E-100	1.55E-50	0.00E+00	4.609	3.457	8.043	5.37
PA0604	*agtB*	AgtB {ABC transporter}	2.6639	1.2521	8.0588	2.31E-47	6.26E-08	0.00E+00	2.664	1.252	8.059	3.99
PA0605	*agtC*	AgtC {ABC transporter permease}	5.7586	2.4138	6.5862	2.95E-131	3.21E-17	5.00E-178	5.759	2.414	6.586	4.92
PA0620	-	Probable bacteriophage protein	0.0769	0.0385	0.8681	1.30E-29	3.29E-193	8.92E-04	-13.000	-26.000	-1.152	-13.38
PA0625	-	Hypothetical protein	0.0429	0.0143	0.9071	3.61E-97	0.00E+00	9.29E-04	-23.333	-70.000	-1.102	-31.48
PA0649	*trpG [pabA]*	Anthranilate synthase component II	2.6452	4.5161	2.4355	1.04E-21	4.55E-75	1.77E-15	2.645	4.516	2.435	3.20
PA0659	-	Hypothetical protein	11.0000	4.8182	1.8364	0.00E+00	7.62E-189	3.36E-17	11.000	4.818	1.836	5.88
PA0660	-	NAD(P)H:quinone reductase	5.1833	2.8750	1.9417	1.34E-223	4.13E-55	1.08E-19	5.183	2.875	1.942	3.33
PA0661	-	Conserved hypothetical protein	8.0952	8.8889	2.2540	1.73E-240	7.36E-300	5.92E-10	8.095	8.889	2.254	6.41
PA0668.2	-	tRNA-Ile	0.0335	0.0063	0.1862	2.95E-142	0.00E+00	3.58E-33	-29.875	-159.333	-5.371	-64.86
PA0672	*hemO [pigA]*	Heme oxygenase	3.7308	2.8846	9.5385	2.69E-27	4.18E-15	9.67E-242	3.731	2.885	9.538	5.38
PA0713	-	Hypothetical protein	0.0365	0.0052	0.0885	5.20E-117	0.00E+00	2.96E-166	-27.429	-192.000	-11.294	-76.91
PA0714.1	*phrD*	PhrD ncRNA	24.1818	143.3636	2.6364	0.00E+00	0.00E+00	8.11E-03	24.182	143.364	2.636	56.73
PA0716	-	Hypothetical protein	0.1000	0.0714	0.4143	2.05E-14	2.11E-23	3.54E-07	-10.000	-14.000	-2.414	-8.80
PA0718	-	Hypothetical protein of bacteriophage Pf1	1.7000	3.5000	2.7000	1.79E-02	6.23E-09	2.41E-04	1.700	3.500	2.700	2.63
PA0730	-	Probable transferase {(R)-3-hydroxydecanoyl-ACP:CoA transacylase}	3.5313	4.2188	1.4688	1.23E-35	4.70E-54	7.79E-03	3.531	4.219	1.469	3.07
PA0734	-	Hypothetical protein	4.9333	4.2667	2.5333	4.25E-22	3.80E-16	2.31E-04	4.933	4.267	2.533	3.91
PA0735	-	Hypothetical protein	2.7667	2.2667	2.0333	2.26E-18	8.56E-12	4.28E-07	2.767	2.267	2.033	2.36

PA0736	-	Hypothetical protein	3.5926	3.0000	2.5556	9.19E-37	6.03E-24	1.99E-14	3.593	3.000	2.556	3.05
{PA0736a}	-	Intergenic region {YP_008719735.1 Hypothetical protein}	2.0820	2.2951	4.6885	5.78E-06	2.57E-07	1.74E-32	2.082	2.295	4.689	3.02
PA0737	-	Hypothetical protein	4.3125	4.8125	9.4375	3.06E-20	5.32E-26	2.14E-123	4.313	4.813	9.438	6.19
PA0738	-	Conserved hypothetical protein	2.0909	1.4545	5.6364	2.09E-03	4.91E-02	1.53E-23	2.091	1.455	5.636	3.06
PA0741	-	Conserved hypothetical protein	1.7333	2.0000	3.4667	4.28E-04	2.06E-05	2.09E-14	1.733	2.000	3.467	2.40
PA0749	-	Hypothetical protein	1.8462	2.3846	1.8462	1.65E-04	1.22E-07	8.63E-03	1.846	2.385	1.846	2.03
PA0763	*mucA*	Anti-sigma factor MucA {sigma factor AlgU negative regulator MucA}	2.2638	5.6521	3.0227	4.18E-49	0.00E+00	3.88E-96	2.264	5.652	3.023	3.65
PA0774	*[yecP]*	Conserved hypothetical protein {tRNA (mo5U34)-methyltransferase}	0.0625	0.1563	0.4063	1.88E-31	3.08E-04	3.50E-06	-16.000	-6.400	-2.462	-8.29
PA0781	-	Hypothetical protein	19.5000	12.5000	4.7500	0.00E+00	1.32E-229	1.34E-22	19.500	12.500	4.750	12.25
PA0791	-	Probable transcriptional regulator	0.1200	0.1067	0.4000	8.32E-08	1.10E-10	4.19E-07	-8.333	-9.375	-2.500	-6.74
PA0796	*prpB [bcpA]*	Carboxyphosphonoenolpyruvate phosphonomutase [CPEP phosphonomutase] {2-methylisocitrate lyase}	0.2045	0.1894	0.4091	2.25E-02	5.82E-05	4.19E-07	-4.889	-5.280	-2.444	-4.20
PA0798	*pmtA*	Phospholipid methyltransferase	0.0196	0.0196	0.2549	2.85E-169	4.66E-261	1.10E-16	-51.000	-51.000	-3.923	-35.31
PA0799	-	Probable helicase	0.1667	0.1667	0.5556	1.18E-03	7.00E-05	1.66E-02	-6.000	-6.000	-1.800	-4.60
PA0826	-	Hypothetical protein	2.6087	2.0870	3.6957	3.88E-11	4.99E-07	1.18E-21	2.609	2.087	3.696	2.80
PA0833	-	Hypothetical protein	2.1233	3.8867	2.2633	1.67E-33	4.09E-138	6.02E-37	2.123	3.887	2.263	2.76
PA0838	*[btuE]*	Probable glutathione peroxidase	1.8357	1.0918	3.0870	2.66E-18	3.26E-05	1.65E-58	1.836	1.092	3.087	2.00
PA0839	-	Probable transcriptional regulator	2.3333	3.6667	4.1667	5.92E-04	1.24E-09	2.88E-11	2.333	3.667	4.167	3.39
PA0840	-	Probable oxidoreductase	2.3000	1.8000	2.0500	2.46E-11	1.46E-06	2.70E-06	2.300	1.800	2.050	2.05
PA0845	*cerN*	CerN {neutral ceramidase}	1.0000	6.3333	2.1667	4.22E-02	1.92E-74	3.69E-05	1.000	6.333	2.167	3.17
PA0854	*fumC2*	Fumarate hydratase [fumarase]	2.2400	3.1040	1.2080	2.94E-35	3.45E-75	4.95E-06	2.240	3.104	1.208	2.18
PA0855	-	Hypothetical protein	3.6400	3.7200	1.3200	5.75E-35	3.03E-37	4.90E-02	3.640	3.720	1.320	2.89
PA0856	-	Hypothetical protein	2.3983	3.5678	1.4195	7.66E-38	6.88E-96	9.76E-09	2.398	3.568	1.419	2.46
PA0857	*bolA*	Morphogene protein BolA	3.1818	5.4675	1.3701	1.87E-39	1.35E-141	1.47E-03	3.182	5.468	1.370	3.34
PA0879	-	Probable acyl-CoA dehydrogenase	2.5000	5.2500	2.2500	1.91E-05	3.21E-26	7.36E-04	2.500	5.250	2.250	3.33
PA0887	*acsA*	Acetyl-CoA synthetase	9.1811	4.2913	26.1732	0.00E+00	7.00E-197	0.00E+00	9.181	4.291	26.173	13.22
PA0891	*[aotO]*	Hypothetical protein	0.2000	0.2381	0.4667	1.96E-02	3.35E-03	5.49E-04	-5.000	-4.200	-2.143	-3.78
PA0892	*aotP*	Arginine/ornithine transporter AotP	0.1789	0.0826	0.3257	3.15E-03	1.09E-33	5.16E-13	-5.590	-12.111	-3.070	-6.92
PA0906	*alpR*	Lysis phenotype represssor, AlpR	2.0952	5.2381	3.7143	2.83E-07	1.01E-54	4.46E-22	2.095	5.238	3.714	3.68
PA0921	-	Hypothetical protein	4.0822	3.7260	2.5205	1.01E-46	8.36E-38	4.22E-13	4.082	3.726	2.521	3.44
{PA0951a}	-	Intergenic region {YP_008719737.1 hypothetical protein PA0951a}	5.5045	5.9136	7.7273	1.35E-127	2.47E-151	6.57E-285	5.505	5.914	7.727	6.38
PA0952	-	Hypothetical protein	3.7470	3.5904	7.2892	6.97E-44	3.10E-39	1.55E-207	3.747	3.590	7.289	4.88
PA0961	-	Probable cold-shock protein	2.5556	4.6111	2.0000	1.53E-08	3.60E-32	1.38E-03	2.556	4.611	2.000	3.06
PA0968	*[ybgC]*	Conserved hypothetical protein	0.0496	0.1348	0.5887	7.29E-53	2.73E-07	2.28E-02	-20.143	-7.421	-1.699	-9.75
PA0976.1	-	tRNA-Lys	2.3182	9.6136	2.2045	2.93E-04	6.33E-96	5.59E-03	2.318	9.614	2.205	4.71
PA1000	*pqsE*	Quinolone signal response protein PqsE {thioesterase PqsE}	0.0859	0.0115	0.0153	1.13E-22	0.00E+00	0.00E+00	-11.644	-87.333	-65.500	-54.83
PA1001	*phnA*	Anthranilate synthase component I [phenazine biosynthesis protein PhnA]	0.1259	0.0291	0.0218	4.79E-08	0.00E+00	0.00E+00	-7.942	-34.417	-45.889	-29.42
PA1002	*phnB*	Anthranilate synthase component II [phenazine biosynthesis protein PhnB]	0.1778	0.0496	0.3294	3.11E-03	2.30E-86	1.45E-12	-5.623	-20.176	-3.035	-9.61
PA1009	-	Hypothetical protein	4.0673	4.3173	1.2981	3.23E-82	3.44E-94	1.30E-03	4.067	4.317	1.298	3.23
PA1011	-	Hypothetical protein	0.5233	0.2432	0.7961	8.75E-03	4.54E-05	2.55E-03	-1.911	-4.111	-1.256	-2.43
PA1041	-	Probable outer membrane protein precursor	0.1395	0.0533	0.2431	1.20E-05	5.36E-192	1.72E-21	-7.167	-18.775	-4.114	-10.02
PA1044	-	Hypothetical protein	3.2105	2.6842	1.7368	4.69E-12	1.72E-08	3.35E-02	3.211	2.684	1.737	2.54
PA1051	-	Probable transporter	2.7500	1.2500	2.6250	1.94E-10	1.02E-02	1.55E-07	2.750	1.250	2.625	2.21
PA1053	*[slyB]*	Conserved hypothetical protein	9.9407	17.2462	2.6764	0.00E+00	0.00E+00	7.94E-83	9.941	17.246	2.676	9.95
PA1066	-	Probable short-chain dehydrogenase	1.9310	1.6379	3.3448	4.31E-11	1.98E-07	5.32E-35	1.931	1.638	3.345	2.30
PA1080	*flgE*	Flagellar hook protein FlgE	3.5000	10.9890	2.3676	9.26E-140	0.00E+00	3.02E-54	3.500	10.989	2.368	5.62
PA1081	*flgF*	Flagellar basal body rod protein FlgF	1.7340	2.0213	2.9787	2.71E-13	5.81E-18	1.01E-42	1.734	2.021	2.979	2.24
PA1082	*flgG*	Flagellar basal body rod protein FlgG	2.1471	3.1471	2.1078	6.43E-32	5.82E-78	8.72E-29	2.147	3.147	2.108	2.47

PA1091	*fgtA*	Flagellar glycosyl transferase, FgtA	0.1224	0.1020	0.7075	1.32E-07	5.23E-49	1.91E-02	-8.167	-9.800	-1.413	-6.46
PA1094	*fliD*	Flagellar capping protein FliD [hook-associated protein] {B-type flagellar hook-associated protein}	2.5576	3.6369	1.4350	5.53E-81	5.65E-186	1.60E-20	2.558	3.637	1.435	2.54
PA1104	*fliI*	Flagellum-specific ATP synthase	0.1067	0.0533	0.4933	1.99E-10	8.61E-55	2.18E-03	-9.375	-18.750	-2.027	-10.05
PA1130	*rhlC*	Rhamnosyltransferase 2	0.0350	0.0909	0.0839	9.84E-124	1.20E-23	1.17E-271	-28.600	-11.000	-11.917	-17.17
PA1134	-	Hypothetical protein	2.8333	2.6667	7.1667	1.13E-05	9.13E-05	1.01E-38	2.833	2.667	7.167	4.22
PA1149	-	Hypothetical protein	6.8000	14.2000	4.0000	3.19E-28	3.95E-160	1.32E-08	6.800	14.200	4.000	8.33
PA1159	-	Probable cold-shock protein	0.1742	0.0158	0.3253	1.81E-03	0.00E+00	9.11E-13	-5.739	-63.471	-3.074	-24.09
PA1162	*dapE*	Succinyl-diaminopimelate desuccinylase [*N*-succinyl-diaminopimelate deacylase]	4.4857	3.5714	1.7143	3.24E-77	8.94E-45	3.69E-06	4.486	3.571	1.714	3.26
PA1179	*phoP*	Two-component response regulator PhoP	7.3590	3.4577	4.2462	0.00E+00	1.77E-162	1.18E-264	7.359	3.458	4.246	5.02
PA1180	*phoQ*	Two-component sensor PhoQ	3.0728	2.8079	2.6358	1.87E-76	7.71E-62	1.92E-51	3.073	2.808	2.636	2.84
PA1183	*dctA*	C4-dicarboxylate transporter DctA	48.7778	35.1111	62.6667	0.00E+00	0.00E+00	0.00E+00	48.778	35.111	62.667	48.85
PA1187	-	Probable acyl-CoA dehydrogenase	15.2000	9.2000	2.2000	0.00E+00	1.77E-110	9.16E-04	15.200	9.200	2.200	8.87
PA1195	*ddaH*	Dimethylarginine dimethylaminohydrolase DdaH	3.7500	5.5000	3.1250	1.89E-14	2.14E-35	3.26E-08	3.750	5.500	3.125	4.13
PA1198	-	Conserved hypothetical protein	0.1494	0.0714	0.5065	1.18E-05	4.60E-45	1.31E-02	-6.696	-14.000	-1.974	-7.56
PA1200	-	Conserved hypothetical protein	0.1923	0.2179	0.5513	5.58E-03	2.62E-02	7.41E-03	-5.200	-4.588	-1.814	-3.87
PA1204	*[yieF]*	NAD(P)H quinone oxidoreductase {NAD(P)H-dependent FMN reductase}	2.3158	1.7368	1.9342	1.25E-17	3.85E-09	3.29E-09	2.316	1.737	1.934	2.00
PA1213	-	Hypothetical protein	0.0541	0.0811	0.0541	3.50E-39	7.12E-18	8.62E-277	-18.500	-12.333	-18.500	-16.44
PA1215	-	Hypothetical protein	0.0351	0.0175	0.0175	5.24E-58	0.00E+00	0.00E+00	-28.500	-57.000	-57.000	-47.50
PA1217	-	Probable 2-isopropylmalate synthase	0.0429	0.0061	0.0675	8.72E-109	0.00E+00	0.00E+00	-23.286	-163.000	-14.818	-67.03
PA1218	-	Hypothetical protein	0.0896	0.0373	0.0373	3.87E-18	1.26E-91	0.00E+00	-11.167	-26.800	-26.800	-21.59
PA1220	-	Hypothetical protein	0.0435	0.0217	0.0217	1.84E-80	3.53E-224	0.00E+00	-23.000	-46.000	-46.000	-38.33
PA1243	-	Probable sensor/response regulator hybrid	0.1061	0.2727	0.2727	1.15E-14	1.77E-02	3.20E-21	-9.429	-3.667	-3.667	-5.59
PA1248	*aprF*	Alkaline protease secretion OMP AprF	0.2097	0.0968	0.2097	2.99E-02	1.10E-12	6.24E-41	-4.769	-10.333	-4.769	-6.62
PA1249	*aprA*	Alkaline metalloproteinase precursor	0.1420	0.1736	0.0347	1.54E-03	2.27E-12	0.00E+00	-7.043	-5.760	-28.800	-13.87
PA1250	*aprI*	Alkaline proteinase inhibitor AprI	0.2180	0.0521	0.0853	3.11E-02	1.64E-49	5.44E-220	-4.587	-19.182	-11.722	-11.83
PA1251	-	Probable chemotaxis transducer [probable (methyl-accepting chemotaxis) protein]	0.1509	0.1698	0.1509	1.54E-05	2.33E-05	5.32E-76	-6.625	-5.889	-6.625	-6.38
PA1254	*lhpC*	Delta1-pyrroline-4-hydroxy-2-carboxylate deaminase, LhpC {dihydrodipicolinate synthetase}	5.3333	39.3333	5.6667	1.51E-23	0.00E+00	1.63E-24	5.333	39.333	5.667	16.78
PA1255	*lhpK*	D-Hydroxyproline epimerase, LhpK {trans-3-hydroxy-L-proline dehydratase}	3.6667	14.6667	9.0000	3.61E-08	4.80E-192	3.01E-61	3.667	14.667	9.000	9.11
PA1256	*lhpO*	ABC transporter ATP-binding protein, LhpO	1.8000	13.8000	6.8000	7.15E-03	1.80E-225	3.51E-43	1.800	13.800	6.800	7.47
PA1296	-	Probable 2-hydroxyacid dehydrogenase	2.0000	6.3438	2.6250	3.11E-10	1.29E-142	1.09E-15	2.000	6.344	2.625	3.66
PA1297	-	Probable metal transporter	8.9091	10.7273	1.9091	2.78E-157	2.10E-244	2.95E-03	8.909	10.727	1.909	7.18
PA1317	*cyoA*	Cytochrome o ubiquinol oxidase subunit II	0.0870	0.1739	0.3478	8.25E-15	3.79E-02	1.38E-07	-11.500	-5.750	-2.875	-6.71
PA1319	*cyoC*	Cytochrome o ubiquinol oxidase subunit III	0.2000	0.1500	0.2000	2.72E-02	3.59E-03	3.91E-15	-5.000	-6.667	-5.000	-5.56
PA1320	*cyoD*	Cytochrome o ubiquinol oxidase subunit IV	0.0645	0.1613	0.4194	3.62E-31	8.22E-04	8.06E-04	-15.500	-6.200	-2.385	-8.03
PA1338	*ggt*	Gamma-glutamyltranspeptidase precursor	1.7283	2.6304	2.5978	3.01E-19	3.10E-50	6.03E-47	1.728	2.630	2.598	2.32
PA1341	*aatQ*	AatQ {amino acid ABC transporter permease}	2.9435	1.6129	3.8629	2.73E-51	2.46E-12	1.88E-95	2.944	1.613	3.863	2.81
PA1342	*aatJ*	AatJ {ABC transporter}	3.6697	3.8608	2.9114	2.87E-203	3.18E-230	2.71E-117	3.670	3.861	2.911	3.48
PA1343	-	Hypothetical protein	24.4189	51.0000	6.6622	0.00E+00	0.00E+00	8.64E-176	24.419	51.000	6.662	27.36
PA1344	*[yvaG]*	Probable short-chain dehydrogenase	4.4808	3.1474	1.8397	2.06E-161	7.81E-70	1.50E-17	4.481	3.147	1.840	3.16
PA1353	-	Hypothetical protein	0.1579	0.0526	0.1930	4.70E-04	2.23E-45	1.20E-22	-6.333	-19.000	-5.182	-10.17
PA1354	-	Hypothetical protein	0.1373	0.0392	0.2549	1.24E-04	1.11E-60	3.79E-13	-7.286	-25.500	-3.923	-12.24
PA1357	-	Conserved hypothetical protein	0.1429	0.0168	0.3613	3.90E-06	0.00E+00	1.07E-09	-7.000	-59.500	-2.767	-23.09
PA1371	-	Hypothetical protein	0.1081	0.0090	0.3153	1.47E-11	0.00E+00	1.59E-14	-9.250	-111.000	-3.171	-41.14
PA1376	*aceK*	Bifunctional isocitrate dehydrogenase kinase/phosphatase	4.8571	7.7460	2.0794	1.69E-185	0.00E+00	1.47E-22	4.857	7.746	2.079	4.89
PA1396	-	Probable two-component sensor	11.0000	3.8889	6.8889	4.77E-150	3.80E-28	2.01E-99	11.000	3.889	6.889	7.26
PA1413	*[yneJ]*	Probable transcriptional regulator	3.0000	1.7143	5.0000	7.09E-08	6.25E-03	2.34E-21	3.000	1.714	5.000	3.24
PA1415	-	Hypothetical protein	2.7937	2.6032	5.3968	3.93E-28	1.04E-23	7.99E-130	2.794	2.603	5.397	3.60

PA1416	-	Conserved hypothetical protein	1.4000	2.7000	2.9000	2.86E-03	1.71E-11	2.98E-11	1.400	2.700	2.900	2.33
PA1418	-	Probable sodium:solute symport protein	3.0000	4.0000	2.6667	5.35E-06	7.91E-11	2.97E-03	3.000	4.000	2.667	3.22
PA1423	*bdlA*	BdlA {biofilm dispersion protein}	3.5556	3.8519	8.0000	4.13E-41	8.90E-49	2.60E-272	3.556	3.852	8.000	5.14
PA1425	-	Probable ATP-binding component of ABC transporter	1.6000	1.6000	2.8000	4.20E-03	2.29E-03	1.94E-06	1.600	1.600	2.800	2.00
PA1428	*[yjaB]*	Conserved hypothetical protein	2.0000	2.2000	11.0000	1.12E-02	4.71E-03	1.77E-88	2.000	2.200	11.000	5.07
PA1440	-	Hypothetical protein	3.0044	1.6903	2.6062	5.66E-65	1.23E-16	5.65E-45	3.004	1.690	2.606	2.43
PA1462	-	Probable plasmid partitioning protein	1.8425	5.1969	1.2835	2.42E-18	2.70E-205	2.79E-05	1.843	5.197	1.283	2.77
PA1482	*ccmH [ccl2, cycL]*	Cytochrome c biogenesis protein CcmH	0.1737	0.2216	0.3234	7.37E-04	5.81E-03	1.61E-13	-5.759	-4.514	-3.093	-4.45
PA1483	*cycH*	Cytochrome c biogenesis protein CycH	0.1772	0.1139	0.3038	2.76E-03	1.16E-10	1.00E-15	-5.643	-8.778	-3.292	-5.90
PA1487	-	Probable carbohydrate kinase	3.2727	3.1818	2.0000	4.25E-20	4.40E-19	3.24E-05	3.273	3.182	2.000	2.82
PA1488	-	Hypothetical protein	3.8333	2.4167	1.6667	2.38E-28	6.19E-10	5.73E-03	3.833	2.417	1.667	2.64
PA1491	-	Probable transporter	2.8333	10.3333	3.5000	1.47E-07	3.81E-146	7.41E-10	2.833	10.333	3.500	5.56
PA1511	*vgrG2a*	T6SS VgrG2a	2.6842	1.2632	2.1053	8.33E-27	9.39E-05	5.99E-13	2.684	1.263	2.105	2.02
PA1512	*hcpA*	T6SS secreted protein Hcp	5.1429	16.2381	2.7619	2.15E-42	0.00E+00	2.31E-08	5.143	16.238	2.762	8.05
PA1518	-	Conserved hypothetical protein [transthyretin family protein] {5-hydroxyisourate hydrolase}	2.5000	5.2083	2.7500	1.50E-07	4.34E-39	1.76E-07	2.500	5.208	2.750	3.49
PA1521	*[gda1]*	Guanine deaminase	4.3913	2.0435	4.2609	1.67E-60	1.59E-10	2.58E-53	4.391	2.043	4.261	3.57
PA1522	-	Hypothetical protein	2.3125	1.1563	3.5000	6.92E-13	4.44E-03	3.08E-30	2.313	1.156	3.500	2.32
PA1527	-	Conserved hypothetical protein	0.1553	0.1650	0.8932	1.47E-04	1.30E-11	7.73E-04	-6.438	-6.059	-1.120	-4.54
PA1538	-	Probable flavin-containing monooxygenase	3.1429	6.8571	1.5714	5.75E-14	1.66E-84	3.53E-02	3.143	6.857	1.571	3.86
PA1540	-	Conserved hypothetical protein {multidrug efflux system protein MdtI}	1.9583	1.6250	3.8333	2.47E-04	2.96E-03	1.91E-15	1.958	1.625	3.833	2.47
PA1541	-	Probable drug efflux transporter	7.7143	6.2857	10.0000	2.91E-48	3.17E-31	7.04E-89	7.714	6.286	10.000	8.00
PA1543	*apt*	Adenine phosphoribosyltransferase	3.9600	4.8400	2.0800	1.69E-59	4.60E-95	4.89E-11	3.960	4.840	2.080	3.63
PA1546	*hemN*	Oxygen-dependent coproporphyrinogen III oxidase	1.8814	2.3434	2.7717	2.07E-42	1.81E-70	4.26E-104	1.881	2.343	2.772	2.33
PA1562	*acnA [can]*	Aconitate hydratase 1 [aconitase]	2.0722	4.3042	2.1293	9.05E-53	6.29E-296	1.12E-55	2.072	4.304	2.129	2.84
PA1566	*pauA3*	Glutamylpolyamine synthetase	9.2500	5.7500	14.0000	5.39E-91	2.15E-29	1.07E-235	9.250	5.750	14.000	9.67
PA1580	*gltA [cisY]*	Citrate synthase	15.4213	12.4619	1.2944	0.00E+00	0.00E+00	1.22E-16	15.421	12.462	1.294	9.73
PA1592	-	Hypothetical protein	1.8133	2.0533	3.1265	2.73E-37	1.98E-49	1.56E-132	1.813	2.053	3.127	2.33
PA1596	*htpG*	Heat shock protein HtpG [chaperone Hsp90] {chaperone HptG}	0.1645	0.0459	0.0309	2.51E-02	0.00E+00	0.00E+00	-6.079	-21.804	-32.355	-20.08
PA1597	-	Hypothetical protein	0.1381	0.0994	0.2541	2.39E-07	6.91E-19	1.00E-27	-7.240	-10.056	-3.935	-7.08
PA1598	-	Conserved hypothetical protein {3-methyl-2-oxobutanoate hydroxymethyltransferase}	0.1321	0.0566	0.2830	6.85E-06	2.26E-36	4.60E-16	-7.571	-17.667	-3.533	-9.59
PA1600	-	Probable cytochrome c	8.3333	10.0000	14.3333	5.22E-97	7.21E-149	0.00E+00	8.333	10.000	14.333	10.89
PA1601	-	Probable aldehyde dehydrogenase	19.7143	12.4286	14.8571	0.00E+00	0.00E+00	0.00E+00	19.714	12.429	14.857	15.67
PA1602	-	Probable oxidoreductase	21.9286	26.7143	18.8571	0.00E+00	0.00E+00	0.00E+00	21.929	26.714	18.857	22.50
PA1603	-	Probable transcriptional regulator	1.9130	5.1304	2.2174	9.37E-05	3.06E-39	1.59E-04	1.913	5.130	2.217	3.09
PA1607	-	Conserved hypothetical protein	0.1716	0.1124	0.1953	9.20E-04	1.41E-11	4.67E-43	-5.828	-8.895	-5.121	-6.61
PA1608	-	Probable chemotaxis transducer [probable methyl-accepting chemotaxis protein]	5.6111	7.1111	3.0556	4.87E-108	1.63E-190	5.39E-24	5.611	7.111	3.056	5.26
PA1609	*fabB*	Beta-oxoacyl-ACP synthase I FabB [KAS I] {3-oxoacyl-ACP synthase}	5.0499	4.2207	1.3532	0.00E+00	1.51E-281	8.46E-18	5.050	4.221	1.353	3.54
PA1610	*fabA*	Beta-hydroxydecanoyl-ACP dehydrase {3-hydroxydecanoyl-ACP dehydratase}	9.6053	3.5757	2.3059	0.00E+00	5.46E-105	6.74E-36	9.605	3.576	2.306	5.16
PA1619	-	Probable transcriptional regulator	1.4167	2.2500	3.7500	7.61E-03	2.72E-06	1.93E-16	1.417	2.250	3.750	2.47
PA1621	-	Probable hydrolase	2.2453	1.4528	2.4528	9.05E-17	2.83E-06	1.66E-17	2.245	1.453	2.453	2.05
PA1622	-	Probable hydrolase	1.6410	1.3590	3.2564	2.68E-07	7.40E-05	5.73E-30	1.641	1.359	3.256	2.09
PA1627	-	Probable transcriptional regulator	1.7273	2.0000	2.9091	3.40E-03	4.67E-04	1.13E-06	1.727	2.000	2.909	2.21
PA1636	*kdpD*	Two-component sensor KdpD	0.2000	0.1000	0.4500	6.78E-03	1.64E-12	2.36E-04	-5.000	-10.000	-2.222	-5.74
PA1643	*[ybbB]*	Conserved hypothetical protein {tRNA 2-selenouridine synthase}	0.1862	0.1241	0.3862	5.78E-03	3.50E-15	1.25E-07	-5.370	-8.056	-2.589	-5.34
PA1646	-	Probable chemotaxis transducer [probable methyl-accepting chemotaxis protein]	0.0769	0.1282	0.5385	2.53E-24	4.25E-06	4.74E-03	-13.000	-7.800	-1.857	-7.55

PA1662	*clpV2*	T6SS ClpA/B-type protease ClpV2	0.1642	0.2687	0.3433	6.75E-04	1.27E-02	3.56E-11	-6.091	-3.722	-2.913	-4.24
PA1663	*sfa2*	T6SS Sfa2 transcriptional regulator	0.1698	0.2075	0.2075	3.56E-03	4.96E-03	4.28E-40	-5.889	-4.818	-4.818	-5.18
PA1664	*orfX*	T6SS protein OrfX	0.1321	0.1321	0.2453	1.31E-05	7.78E-07	4.34E-09	-7.571	-7.571	-4.077	-6.41
PA1670	*stp1*	T6SS serine/threonine phosphoprotein phosphatase Stp1	0.0189	0.0566	0.1509	1.03E-289	1.37E-28	4.08E-51	-53.000	-17.667	-6.625	-25.76
PA1671	*stk1*	T6SS serine-threonine kinase Stk1	0.0323	0.9355	0.4194	3.54E-95	2.46E-02	8.52E-06	-31.000	-1.069	-2.385	-11.48
PA1676		Hypothetical protein	2.2308	3.9231	6.2308	4.03E-04	4.94E-13	4.60E-35	2.231	3.923	6.231	4.13
PA1677	-	Conserved hypothetical protein	3.1316	5.2193	4.4825	3.00E-49	1.39E-164	2.29E-113	3.132	5.219	4.482	4.28
PA1680	-	Hypothetical protein	2.0000	2.6000	2.6000	8.64E-04	5.24E-07	1.96E-05	2.000	2.600	2.600	2.40
PA1681	*aroC*	Chorismate synthase [5-enolpyruvylshikimate-3-phosphate phospholyase]	4.3373	2.5301	1.5422	1.08E-124	7.15E-35	9.20E-09	4.337	2.530	1.542	2.80
PA1692	*[pscS]*	T3SS probable translocation protein	3.0000	3.0000	2.2222	1.87E-05	3.18E-05	2.17E-02	3.000	3.000	2.222	2.74
PA1693	*pscR*	T3SS translocation protein PscR	4.3529	5.0588	2.4706	8.61E-28	7.29E-40	2.80E-06	4.353	5.059	2.471	3.96
PA1694	*pscQ*	T3SS translocation protein PscQ	8.7273	11.2727	2.1818	2.50E-140	3.96E-256	2.40E-04	8.727	11.273	2.182	7.39
PA1695	*pscP*	T3SS translocation protein PscP	5.4286	5.1429	2.8571	3.74E-37	2.26E-33	8.07E-07	5.429	5.143	2.857	4.48
PA1696	*pscO*	T3SS translocation protein PscO	1.6364	2.6364	2.6364	9.91E-03	4.28E-06	9.91E-05	1.636	2.636	2.636	2.30
PA1697	*[pscN]*	T3SS ATP synthase {T3SS ATPase}	2.7143	2.2857	4.8571	8.15E-08	3.21E-06	5.48E-27	2.714	2.286	4.857	3.29
PA1698	*popN*	T3SS OMP PopN precursor	12.5000	5.3333	11.5000	1.81E-184	2.63E-25	3.12E-149	12.500	5.333	11.500	9.78
PA1699	*pcr1*	T3SS translocation protein Pcr1	10.0000	9.2857	9.4286	1.56E-66	1.64E-56	3.51E-57	10.000	9.286	9.429	9.57
PA1700	*pcr2*	T3SS translocation protein Pcr2	3.6667	2.5000	12.1667	1.47E-07	8.07E-04	4.75E-112	3.667	2.500	12.167	6.11
PA1703	*pcrD*	T3SS secretory apparatus protein PcrD	2.3750	1.8750	5.0000	1.72E-09	5.61E-06	1.33E-46	2.375	1.875	5.000	3.08
PA1704	*pcrR*	T3SS transcriptional regulator PcrR	3.3333	2.6667	2.3333	2.99E-08	1.75E-05	3.36E-03	3.333	2.667	2.333	2.78
PA1705	*pcrG*	T3SS regulator PcrG	8.0000	10.3333	41.6667	7.86E-39	1.85E-71	0.00E+00	8.000	10.333	41.667	20.00
PA1706	*pcrV*	T3SS translocator protein PcrV	24.4500	23.3500	28.3000	0.00E+00	0.00E+00	0.00E+00	24.450	23.350	28.300	25.37
PA1707	*pcrH*	T3SS regulatory protein PcrH	28.4500	23.8000	34.3000	0.00E+00	0.00E+00	0.00E+00	28.450	23.800	34.300	28.85
PA1708	*popB [pepB]*	T3SS translocator protein PopB	47.9130	30.1957	33.2609	0.00E+00	0.00E+00	0.00E+00	47.913	30.196	33.261	37.12
PA1709	*popD [pepD]*	T3SS translocator OMP PopD precursor	15.4667	4.0444	35.2000	0.00E+00	2.43E-61	0.00E+00	15.467	4.044	35.200	18.24
PA1710	*exsC*	T3SS exoenzyme S synthesis protein C precursor ExsC	2.6000	1.0190	6.4286	4.17E-24	4.04E-03	3.50E-200	2.600	1.019	6.429	3.35
PA1713	*exsA*	T3SS transcriptional regulator ExsA [S synthesis trans-regulatory protein ExsA]	1.8400	1.2800	4.5200	6.67E-07	1.97E-03	6.03E-48	1.840	1.280	4.520	2.55
PA1714	*exsD*	T3SS anti-activator protein ExsD	3.0000	1.1304	11.3696	1.31E-28	1.58E-03	0.00E+00	3.000	1.130	11.370	5.17
PA1715	*pscB*	T3SS export apparatus protein PscB	5.3636	5.3636	13.1818	1.53E-29	1.50E-29	2.83E-234	5.364	5.364	13.182	7.97
PA1716	*pscC*	T3SS OMP PscC precursor	4.1818	7.7273	11.7273	2.99E-38	1.73E-159	0.00E+00	4.182	7.727	11.727	7.88
PA1717	*pscD*	T3SS export protein PscD	12.0000	20.6667	13.5000	1.26E-209	0.00E+00	7.62E-276	12.000	20.667	13.500	15.39
PA1718	*pscE*	T3SS export protein PscE	29.7813	31.5625	11.4375	0.00E+00	0.00E+00	1.02E-195	29.781	31.563	11.438	24.26
PA1719	*pscF*	T3SS export protein PscF	5.0870	2.7391	10.7826	5.76E-44	4.35E-11	2.25E-255	5.087	2.739	10.783	6.20
PA1721	*pscH*	T3SS export protein PscH	4.2143	1.7857	10.8571	1.57E-17	2.30E-03	8.39E-157	4.214	1.786	10.857	5.62
PA1722	*pscI*	T3SS export protein PscI	7.0000	4.5000	17.0000	1.72E-46	2.78E-17	0.00E+00	7.000	4.500	17.000	9.50
PA1724	*pscK*	T3SS export protein PscK	6.6667	4.0000	11.3333	2.76E-24	9.11E-09	2.14E-81	6.667	4.000	11.333	7.33
PA1736	-	Probable acetyl-CoA acetyltransferase	4.6667	2.9000	2.8333	5.74E-78	1.27E-25	1.24E-21	4.667	2.900	2.833	3.47
PA1737	-	Probable 3-hydroxyacyl-CoA dehydrogenase	4.9394	4.5758	2.0000	1.74E-146	3.01E-123	2.35E-15	4.939	4.576	2.000	3.84
PA1742	*pauD2*	Glutamine amidotransferase class I {amidotransferase}	0.6448	0.2897	0.9966	6.45E-03	1.77E-02	2.18E-03	-1.551	-3.452	-1.003	-2.00
PA1747	-	Hypothetical protein	3.7206	6.6618	9.8676	5.82E-23	1.57E-89	4.46E-224	3.721	6.662	9.868	6.75
PA1754	*cysB*	Transcriptional regulator CysB	6.3378	7.1472	2.2308	0.00E+00	0.00E+00	1.07E-43	6.338	7.147	2.231	5.24
PA1757	*thrH*	Homoserine kinase {phosphoserine phosphatase}	5.6154	3.1538	1.3846	4.03E-95	9.11E-25	3.50E-02	5.615	3.154	1.385	3.38
PA1763	-	Hypothetical protein	1.1250	2.7500	2.7500	3.98E-02	3.04E-09	5.31E-07	1.125	2.750	2.750	2.21
PA1768	-	Hypothetical protein	1.5400	3.9000	1.8600	9.28E-06	2.38E-42	1.04E-05	1.540	3.900	1.860	2.43
PA1776	*sigX*	ECF sigma factor SigX {RNA polymerase sigma factor SigX}	0.1386	0.0950	0.4393	1.83E-06	5.28E-40	1.58E-03	-7.213	-10.525	-2.277	-6.67
PA1779	*[nasC, nasA]*	Assimilatory nitrate reductase	1.6667	2.0000	2.6667	2.62E-03	1.45E-03	4.02E-05	1.667	2.000	2.667	2.11
PA1780	*nirD [nasE]*	Assimilatory nitrite reductase small subunit	7.6667	3.0000	2.6667	1.08E-27	8.75E-04	1.24E-02	7.667	3.000	2.667	4.44
PA1784	-	Hypothetical protein	0.0532	0.8830	0.1277	1.01E-46	1.05E-02	2.31E-90	-18.800	-1.133	-7.833	-9.26
PA1786	*nasS [nasF]*	NasS [probable nitrate-binding protein]	2.3333	2.0000	2.6667	2.52E-04	3.28E-03	5.84E-04	2.333	2.000	2.667	2.33
PA1791	-	Hypothetical protein	11.5000	5.5000	2.5000	1.04E-299	8.24E-54	9.47E-07	11.500	5.500	2.500	6.50

PA1796	*folD*	Bifunctional 5,10-methylene-tetrahydrofolate/5,10-methylene-tetrahydrofolate cyclohydrolase	3.7419	3.5645	1.1613	4.30E-63	6.82E-56	2.91E-02	3.742	3.565	1.161	2.82
PA1801	*clpP*	ClpP {ATP-dependent Clp protease proteolytic subunit}	2.0150	2.1257	1.8649	6.11E-37	1.94E-41	9.33E-29	2.015	2.126	1.865	2.00
PA1813	*[gloB]*	Probable hydroxyacylglutathione hydrolase [probable glyoxalase II]	2.3415	4.8293	1.9756	5.79E-15	8.15E-79	9.32E-08	2.341	4.829	1.976	3.05
PA1837	-	Hypothetical protein	0.2034	0.0339	0.2542	1.04E-02	1.74E-138	5.72E-25	-4.917	-29.500	-3.933	-12.78
PA1839	-	Hypothetical protein {RNA methyltransferase}	0.0645	0.0645	0.3548	4.87E-25	6.09E-16	9.32E-08	-15.500	-15.500	-2.818	-11.27
PA1843	*metH*	Methionine synthase [5-methyltetrahydrofolate--homocysteine *S*-methyltransferase] {B12-dependent methionine synthase}	2.8750	2.5469	2.0156	5.03E-72	2.92E-54	9.53E-30	2.875	2.547	2.016	2.48
PA1845	*tsi1*	T6SS immunity protein Tsi1	63.0000	34.5000	12.3333	0.00E+00	0.00E+00	4.09E-146	63.000	34.500	12.333	36.61
PA1847	*nfuA [yhgI]*	NfuA {Fe/S biogenesis protein NfuA}	2.1679	3.5474	2.1971	1.12E-32	3.61E-103	6.52E-32	2.168	3.547	2.197	2.64
PA1863	*modA*	Molybdate-binding periplasmic protein ModA	3.8000	4.5333	2.6000	2.92E-21	5.12E-33	1.59E-07	3.800	4.533	2.600	3.64
PA1864	-	Probable transcriptional regulator	5.0000	12.0000	9.0000	4.76E-05	4.51E-35	2.77E-17	5.000	12.000	9.000	8.67
PA1871	*lasA*	LasA protease precursor	0.0478	0.0159	0.0289	1.02E-103	0.00E+00	0.00E+00	-20.939	-62.818	-34.550	-39.44
PA1874	-	Hypothetical protein	0.1440	0.6480	0.0720	1.68E-04	3.25E-03	0.00E+00	-6.944	-1.543	-13.889	-7.46
PA1875	*[opmL]*	Probable OMP precursor	0.0244	0.0488	0.0976	3.64E-236	1.32E-29	3.16E-117	-41.000	-20.500	-10.250	-23.92
PA1876	-	Probable ATP-binding/permease fusion ABC transporter	0.0196	0.0196	0.1176	5.72E-181	9.10E-170	6.44E-142	-51.000	-51.000	-8.500	-36.83
PA1877	-	Probable secretion protein	0.0645	0.0645	0.0968	5.79E-17	5.23E-20	4.25E-135	-15.500	-15.500	-10.333	-13.78
PA1878	-	Hypothetical protein	0.8750	0.0750	0.3750	3.29E-02	6.36E-18	5.30E-06	-1.143	-13.333	-2.667	-5.71
PA1880	-	Probable oxidoreductase	0.1600	0.2100	0.3300	4.78E-04	3.89E-05	7.85E-12	-6.250	-4.762	-3.030	-4.68
PA1887	-	Hypothetical protein	0.0283	0.0849	0.1321	5.23E-217	3.05E-18	5.06E-84	-35.333	-11.778	-7.571	-18.23
PA1892	-	Hypothetical protein	0.0294	0.0882	0.2353	1.50E-213	9.26E-14	4.79E-16	-34.000	-11.333	-4.250	-16.53
PA1898	*qscR*	Quorum-sensing control repressor	0.1935	0.0645	0.1935	4.19E-02	8.30E-18	3.15E-19	-5.167	-15.500	-5.167	-8.61
PA1899	*phzA2*	Probable phenazine biosynthesis protein PhzA2	0.0108	0.1047	0.0036	0.00E+00	7.21E-18	0.00E+00	-92.333	-9.552	-277.000	-126.30
PA1900	*phzB2*	Probable phenazine biosynthesis protein PhzB2	0.0016	0.0099	0.0036	0.00E+00	0.00E+00	0.00E+00	-639.667	-101.000	-274.143	-338.27
PA1901	*phzC2*	Phenazine biosynthesis protein PhzC2	0.0112	0.0664	0.0138	0.00E+00	1.45E-184	0.00E+00	-89.412	-15.050	-72.381	-58.95
PA1902	*phzD2*	Phenazine biosynthesis protein PhzD2	0.0017	0.0398	0.0040	0.00E+00	0.00E+00	0.00E+00	-586.333	-25.129	-251.286	-287.58
PA1903	*phzE2*	Phenazine biosynthesis protein PhzE2	0.0029	0.0130	0.0058	0.00E+00	0.00E+00	0.00E+00	-347.000	-77.111	-173.500	-199.20
PA1904	*phzF2*	Probable phenazine biosynthesis protein PhzF2 {trans-2,3-dihydro-3-hydroxyanthranilate isomerase}	0.0062	0.0155	0.0093	0.00E+00	0.00E+00	0.00E+00	-161.667	-64.667	-107.778	-111.37
PA1905	*phzG2*	Probable pyridoxamine 5'-phosphate oxidase	0.0105	0.0119	0.0171	0.00E+00	0.00E+00	0.00E+00	-94.875	-84.333	-58.385	-79.20
PA1906	-	Hypothetical protein	0.1387	0.0173	0.1329	6.31E-07	0.00E+00	3.14E-98	-7.208	-57.667	-7.522	-24.13
PA1907	-	Hypothetical protein	0.0345	0.0345	0.1034	5.93E-59	4.67E-101	4.08E-111	-29.000	-29.000	-9.667	-22.56
PA1913	-	Hypothetical protein	0.0615	0.1385	0.3538	8.67E-30	2.80E-05	1.87E-09	-16.250	-7.222	-2.826	-8.77
PA1914	*[hvn]*	Conserved hypothetical protein	0.0781	0.8281	0.0469	2.92E-19	1.62E-02	0.00E+00	-12.800	-1.208	-21.333	-11.78
PA1922	*[cirA/feuA]*	Probable TonB-dependent receptor	5.5000	2.0000	3.5000	7.91E-16	1.54E-02	5.29E-05	5.500	2.000	3.500	3.67
PA1927	*metE*	5-Methyltetrahydropteroyltriglutamate-homocysteine *S*-methyltransferase	94.1111	16.7778	66.3333	0.00E+00	0.00E+00	0.00E+00	94.111	16.778	66.333	59.07
PA1933	*[yagR]*	Probable hydroxylase large subunit	0.1600	0.1200	0.8900	1.56E-04	2.64E-18	2.83E-02	-6.250	-8.333	-1.124	-5.24
PA1934	-	Hypothetical protein	6.0833	6.0500	3.7833	1.06E-110	3.06E-109	5.38E-34	6.083	6.050	3.783	5.31
PA1946	*rbsB*	Ribose ABC transporter substrate-binding protein	0.1324	0.1096	0.1963	2.46E-07	3.14E-23	2.83E-57	-7.552	-9.125	-5.093	-7.26
PA1947	*rbsA*	Ribose transport protein RbsA	0.0889	0.1222	0.2667	1.49E-18	4.54E-14	2.86E-24	-11.250	-8.182	-3.750	-7.73
PA1948	*rbsC*	Ribose ABC transporter permease	0.1020	0.0306	0.3163	2.64E-15	2.04E-131	4.41E-15	-9.800	-32.667	-3.161	-15.21
PA1949	*rbsR*	Ribose operon repressor RbsR	0.1000	0.0846	0.5231	3.96E-14	1.61E-25	1.42E-02	-10.000	-11.818	-1.912	-7.91
PA1950	*rbsK*	Ribokinase	0.1069	0.0818	0.4969	3.92E-13	1.38E-30	4.55E-03	-9.353	-12.231	-2.013	-7.87
PA1964	*[ybiT]*	Probable ATP-binding component of ABC transporter {ABC-F family ATPase}	1.6897	1.6207	3.1034	4.58E-09	4.94E-08	3.27E-32	1.690	1.621	3.103	2.14
PA1978	*erbR [agmR]*	Response regulator ErbR {glycerol metabolism activator}	3.5000	9.1667	7.0000	2.15E-08	1.83E-73	1.47E-37	3.500	9.167	7.000	6.56
PA1994	-	Hypothetical protein	1.5333	2.1667	2.5333	1.55E-04	8.58E-09	1.57E-09	1.533	2.167	2.533	2.08
PA1998	*dhcR*	Transcriptional regulator DhcR	2.7727	2.1818	2.2727	1.68E-16	1.16E-09	5.23E-08	2.773	2.182	2.273	2.41
PA2001	*atoB*	Acetyl-CoA acetyltransferase	0.1751	0.0503	0.1444	2.46E-02	1.18E-255	4.31E-100	-5.713	-19.870	-6.924	-10.84
PA2004	-	Conserved hypothetical protein	0.1481	0.0741	0.3333	5.92E-05	3.54E-10	8.38E-09	-6.750	-13.500	-3.000	-7.75
PA2007	*maiA*	Maleylacetoacetate isomerase	0.1172	0.1198	0.0938	2.89E-10	2.20E-21	0.00E+00	-8.533	-8.348	-10.667	-9.18

PA2010	*hmgR*	Transcriptional regulator HmgR	0.1148	0.0164	0.3115	1.73E-09	0.00E+00	1.89E-12	-8.714	-61.000	-3.211	-24.31
PA2011	*liuE [gnyL, mvaB]*	3-Hydroxy-3-methylglutaryl-CoA lyase {3-hydroxy-3-isohexenylglutaryl-CoA/hydroxy-methylglutaryl-CoA lyase}	0.1659	0.1572	0.8821	5.41E-04	4.06E-11	4.54E-02	-6.026	-6.361	-1.134	-4.51
PA2012	*liuD [mccA, gnyA, accA]*	Methylcrotonyl-CoA carboxylase alpha subunit (biotin containing)	0.1779	0.1250	0.9375	5.87E-03	7.44E-24	3.70E-05	-5.622	-8.000	-1.067	-4.90
PA2015	*liuA [ivd, gnyD]*	Putative isovaleryl-CoA dehydrogenase	0.1855	0.6588	0.3369	1.84E-02	6.16E-03	1.54E-07	-5.391	-1.518	-2.968	-3.29
PA2019	*mexX [mexG, amrA]*	RND multidrug efflux MFP MexX precursor {multidrug efflux lipoprotein}	2.7500	9.2500	5.0000	6.52E-07	3.63E-104	6.15E-23	2.750	9.250	5.000	5.67
PA2025	*gor*	Glutathione reductase	2.5105	2.1474	3.3737	1.33E-56	2.62E-39	2.14E-112	2.511	2.147	3.374	2.68
PA2026	*[yfeH]*	Conserved hypothetical protein	3.9000	3.4500	3.8500	1.61E-33	1.69E-25	8.76E-30	3.900	3.450	3.850	3.73
PA2027	-	Hypothetical protein	30.8333	11.8333	2.1667	0.00E+00	2.73E-123	2.00E-02	30.833	11.833	2.167	14.94
PA2030	-	Hypothetical protein	0.0823	0.0127	0.0759	1.65E-16	0.00E+00	2.05E-226	-12.154	-79.000	-13.167	-34.77
PA2031	-	Hypothetical protein	0.2240	0.1360	0.0640	3.73E-02	1.21E-05	3.92E-221	-4.464	-7.353	-15.625	-9.15
PA2033	-	Hypothetical protein	2.2000	2.6667	9.6667	1.65E-07	2.31E-11	1.91E-213	2.200	2.667	9.667	4.84
PA2040	*pauA4*	Glutamylpolyamine synthetase {glutamine synthetase}	8.4896	8.5729	9.1250	0.00E+00	0.00E+00	0.00E+00	8.490	8.573	9.125	8.73
PA2041	-	Amino acid permease	10.1250	14.0000	11.5000	0.00E+00	0.00E+00	0.00E+00	10.125	14.000	11.500	11.88
PA2045	*[yidD]*	Conserved hypothetical protein {membrane protein insertion efficiency factor}	2.6667	3.4444	3.3333	1.68E-04	2.06E-07	3.89E-06	2.667	3.444	3.333	3.15
PA2066	-	Hypothetical protein	0.0120	0.0241	0.1928	0.00E+00	8.89E-275	8.16E-37	-83.000	-41.500	-5.188	-43.23
PA2072	-	Conserved hypothetical protein	0.0488	0.2439	0.2927	2.30E-71	1.52E-02	5.97E-16	-20.500	-4.100	-3.417	-9.34
PA2076	-	Probable transcriptional regulator	0.8621	0.1379	0.3793	3.03E-02	2.75E-04	5.50E-06	-1.160	-7.250	-2.636	-3.68
PA2100	-	Probable transcriptional regulator	4.0313	3.3125	5.6563	2.02E-64	7.97E-41	6.12E-142	4.031	3.313	5.656	4.33
PA2108	-	Probable decarboxylase {thiamine pyrophosphate protein}	0.0625	0.1875	0.3750	1.47E-08	7.45E-03	3.71E-06	-16.000	-5.333	-2.667	-8.00
PA2109	-	Hypothetical protein	0.0536	0.0268	0.1161	1.85E-44	4.43E-145	9.91E-100	-18.667	-37.333	-8.615	-21.54
PA2110	-	Hypothetical protein	0.0461	0.0177	0.0284	2.53E-96	0.00E+00	0.00E+00	-21.692	-56.400	-35.250	-37.78
PA2111	-	Hypothetical protein	0.1086	0.0579	0.0410	5.92E-12	2.58E-147	0.00E+00	-9.211	-17.271	-24.382	-16.95
PA2116	-	Conserved hypothetical protein	0.0281	0.0159	0.0281	0.00E+00	0.00E+00	0.00E+00	-35.609	-63.000	-35.609	-44.74
PA2117	-	Hypothetical protein	0.0930	0.0407	0.0523	7.49E-19	2.73E-185	0.00E+00	-10.750	-24.571	-19.111	-18.14
PA2118	*ada*	O^6-methylguanine-DNA methyltransferase	0.1346	0.0385	0.0962	1.04E-06	1.86E-101	2.11E-130	-7.429	-26.000	-10.400	-14.61
PA2122	-	Hypothetical protein	1.8750	3.2500	4.1250	2.85E-04	6.24E-12	8.73E-18	1.875	3.250	4.125	3.08
PA2142	*[yhxC]*	Probable short-chain dehydrogenase	0.0800	0.1600	0.4400	2.42E-18	4.95E-03	1.80E-04	-12.500	-6.250	-2.273	-7.01
PA2160	*[glgX]*	Probable glycosyl hydrolase	1.2500	2.6250	2.3750	2.90E-03	1.21E-11	4.28E-07	1.250	2.625	2.375	2.08
PA2190	-	Conserved hypothetical protein	0.1429	0.0429	0.5714	3.75E-05	1.05E-55	1.78E-02	-7.000	-23.333	-1.750	-10.69
PA2191	*exoY*	T3SS adenylate cyclase ExoY	16.0000	3.1667	7.8333	0.00E+00	4.51E-09	1.38E-64	16.000	3.167	7.833	9.00
PA2193	*hcnA*	Hydrogen cyanide synthase subunit HcnA	0.0176	0.0088	0.0053	0.00E+00	0.00E+00	0.00E+00	-56.900	-113.800	-189.667	-120.12
PA2194	*hcnB*	Hydrogen cyanide synthase subunit HcnB	0.0101	0.0232	0.0101	0.00E+00	0.00E+00	0.00E+00	-98.714	-43.188	-98.714	-80.21
PA2195	*hcnC*	Hydrogen cyanide synthase subunit HcnC	0.0089	0.0168	0.0059	0.00E+00	0.00E+00	0.00E+00	-112.556	-59.588	-168.833	-113.66
PA2199	-	Probable dehydrogenase	0.1656	0.0927	0.2384	4.41E-04	2.78E-21	6.84E-32	-6.040	-10.786	-4.194	-7.01
PA2200	-	Cyclic-guanylate-specific phosphodiesterase	0.1200	0.2000	0.2800	4.04E-07	3.57E-02	3.54E-15	-8.333	-5.000	-3.571	-5.63
PA2210	-	Probable MFS transporter	1.6667	5.3333	2.6667	2.12E-02	2.35E-24	4.56E-04	1.667	5.333	2.667	3.22
PA2211	-	Conserved hypothetical protein	4.6000	1.6000	2.6000	1.34E-20	8.06E-03	5.64E-05	4.600	1.600	2.600	2.93
PA2223	-	Hypothetical protein	0.0826	0.1927	0.1101	1.60E-20	1.94E-04	5.46E-148	-12.111	-5.190	-9.083	-8.79
PA2227	*vqsM*	AraC family transcriptional regulator VqsM {HTH-type transcriptional regulator VqsM}	0.1282	0.0513	0.2051	3.49E-05	3.70E-23	5.07E-26	-7.800	-19.500	-4.875	-10.73
PA2252	-	Probable AGCS sodium/alanine/glycine symporter	4.1250	1.2500	2.2500	4.53E-24	1.75E-02	1.05E-04	4.125	1.250	2.250	2.54
PA2253	*ansA*	L-Asparaginase I	2.4615	1.6154	2.0769	2.25E-09	3.50E-04	2.83E-04	2.462	1.615	2.077	2.05
PA2267	-	Probable transcriptional regulator	0.1628	0.2093	0.5116	1.25E-03	1.43E-02	2.18E-03	-6.143	-4.778	-1.955	-4.29
PA2272	*pbpC*	Penicillin-binding protein 3A	0.1875	0.0625	0.5000	1.72E-02	7.75E-26	7.20E-04	-5.333	-16.000	-2.000	-7.78
PA2274	-	Hypothetical protein	0.0923	0.0154	0.0923	2.42E-10	0.00E+00	7.55E-101	-10.833	-65.000	-10.833	-28.89
PA2292	-	Hypothetical protein	2.0000	8.5000	3.5000	4.22E-02	2.42E-23	2.37E-04	2.000	8.500	3.500	4.67
PA2294	-	Probable ATP-binding component of ABC transporter	2.0000	2.0000	3.6667	4.05E-03	1.04E-03	8.61E-10	2.000	2.000	3.667	2.56
PA2296	-	Hypothetical protein	3.3333	3.3333	3.3333	1.55E-06	1.55E-06	1.28E-05	3.333	3.333	3.333	3.33
PA2302	*ambE*	AmbE	0.1667	0.1133	0.0133	1.27E-02	7.73E-42	0.00E+00	-6.000	-8.824	-75.000	-29.94

PA2304	*ambC*	AmbC	0.5671	0.1646	0.0427	3.89E-02	2.53E-09	0.00E+00	-1.763	-6.074	-23.429	-10.42
PA2309	-	Hypothetical protein	12.7500	2.2500	3.7500	5.64E-169	3.12E-04	6.09E-09	12.750	2.250	3.750	6.25
{PA2312a}	-	No gene {YP_008719754.1 hypothetical protein PA2312a}	4.4000	3.1143	2.7714	2.33E-42	2.21E-19	2.44E-12	4.400	3.114	2.771	3.43
PA2317	-	Probable oxidoreductase	2.7273	4.4545	1.6364	7.42E-13	1.96E-37	9.34E-03	2.727	4.455	1.636	2.94
PA2321	*gntK*	Gluconokinase GntK	5.6667	11.3333	4.3333	6.15E-14	1.16E-62	5.47E-07	5.667	11.333	4.333	7.11
PA2323	*gapN [gapB]*	Glyceraldehyde-3-phosphate dehydrogenase GapN	11.2500	3.8750	5.1875	0.00E+00	6.73E-41	8.59E-79	11.250	3.875	5.188	6.77
PA2326	-	Hypothetical protein	1.7500	2.7500	1.7500	3.30E-03	1.99E-07	2.04E-02	1.750	2.750	1.750	2.08
PA2337	*mtlR*	Transcriptional regulator MtlR	4.1538	3.0769	2.0000	6.38E-29	2.25E-14	3.96E-04	4.154	3.077	2.000	3.08
PA2361	*icmF3*	T6SS protein IcmF3	0.0571	0.2571	0.2000	5.45E-30	1.73E-02	3.83E-42	-17.500	-3.889	-5.000	-8.80
PA2362	*dotU3*	T6SS protein DotU3	0.1316	0.0263	0.2895	1.33E-05	1.98E-72	7.13E-12	-7.600	-38.000	-3.455	-16.35
PA2363	*hsiJ3*	T6SS protein HsiJ3	0.0714	0.0357	0.3929	1.65E-34	5.95E-126	5.41E-08	-14.000	-28.000	-2.545	-14.85
PA2366	*hsiC3 [puuD]*	T6SS protein HsiC3, probable uricase	0.1569	0.8676	0.2157	2.31E-04	1.28E-04	3.31E-41	-6.375	-1.153	-4.636	-4.05
PA2367	*hsiF3*	T6SS protein HsiF3	0.1092	0.2096	0.0786	1.74E-12	5.91E-04	6.44E-291	-9.160	-4.771	-12.722	-8.88
PA2371	*clpV3*	T6SS probable ClpA/B-type protease ClpV3	0.0541	0.1216	0.1216	2.69E-55	6.98E-16	4.61E-139	-18.500	-8.222	-8.222	-11.65
PA2373	*vgrG3*	T6SS protein VgrG3	0.0571	0.0714	0.1143	1.19E-54	1.53E-34	1.15E-154	-17.500	-14.000	-8.750	-13.42
PA2374	*tseF*	T6SS effector protein TseF	0.0952	0.1190	0.3810	4.69E-08	4.58E-07	4.59E-06	-10.500	-8.400	-2.625	-7.18
PA2377	-	Hypothetical protein	136.0000	68.0000	95.0000	0.00E+00	0.00E+00	0.00E+00	136.000	68.000	95.000	99.67
PA2379	-	Probable oxidoreductase	0.1279	0.0930	0.5814	7.62E-07	3.72E-11	1.67E-02	-7.818	-10.750	-1.720	-6.76
PA2381	-	Hypothetical protein	0.1102	0.0729	0.0623	2.45E-12	1.12E-74	0.00E+00	-9.073	-13.726	-16.060	-12.95
PA2383	-	Probable transcriptional regulator	3.1667	5.3333	6.1667	3.78E-08	7.61E-26	3.63E-33	3.167	5.333	6.167	4.89
PA2385	*pvdQ*	3-Oxo-C12-homoserine lactone acylase PvdQ {acyl-homoserine lactone acylase PvdQ}	46.3333	60.3333	16.3333	0.00E+00	0.00E+00	0.00E+00	46.333	60.333	16.333	41.00
PA2386	*pvdA*	L-Ornithine N5-oxygenase	52.0000	106.6970	7.2121	0.00E+00	0.00E+00	8.46E-251	52.000	106.697	7.212	55.30
PA2389	*pvdR*	Pyoverdine biosynthesis efflux pump PvdR {pyoverdine biosynthesis protein PvdR}	14.7500	18.5000	10.7500	0.00E+00	0.00E+00	2.48E-217	14.750	18.500	10.750	14.67
PA2390	*pvdT*	Pyoverdine biosynthesis efflux pump PvdT {pyoverdine biosynthesis protein PvdT}	4.5000	4.6250	6.3750	9.72E-38	1.24E-40	1.96E-84	4.500	4.625	6.375	5.17
PA2391	*opmQ*	Pyoverdine biosynthesis probable OMP OpmQ	10.4000	14.6000	10.8000	2.01E-138	2.92E-311	2.12E-146	10.400	14.600	10.800	11.93
PA2392	*pvdP*	Pyoverdine biosynthesis protein PvdP	12.8000	8.6000	22.2000	2.41E-304	5.12E-118	0.00E+00	12.800	8.600	22.200	14.53
PA2393	-	Pyoverdine biosynthesis putative dipeptidase	29.5714	30.8571	14.5714	0.00E+00	0.00E+00	0.00E+00	29.571	30.857	14.571	25.00
PA2394	*pvdN*	Pyoverdine biosynthesis protein PvdN	62.5000	89.0000	22.2500	0.00E+00	0.00E+00	0.00E+00	62.500	89.000	22.250	57.92
PA2395	*pvdO*	Pyoverdine biosynthesis protein PvdO	25.2500	17.7500	16.8750	0.00E+00	0.00E+00	0.00E+00	25.250	17.750	16.875	19.96
PA2396	*pvdF*	Pyoverdine synthetase PvdF	9.9286	8.0000	9.5357	0.00E+00	6.41E-215	0.00E+00	9.929	8.000	9.536	9.15
PA2397	*pvdE*	Pyoverdine biosynthesis protein PvdE	10.7222	14.1667	4.5000	0.00E+00	0.00E+00	7.61E-60	10.722	14.167	4.500	9.80
PA2398	*fpvA*	Ferripyoverdine receptor	11.8571	15.3333	5.5714	0.00E+00	0.00E+00	1.39E-148	11.857	15.333	5.571	10.92
PA2399	*pvdD*	Pyoverdine synthetase PvdD	5.9500	3.1000	17.9000	0.00E+00	4.18E-69	0.00E+00	5.950	3.100	17.900	8.98
PA2400	*pvdJ*	Pyoverdine biosynthesis protein PvdJ	13.4615	3.5385	30.7692	0.00E+00	1.13E-71	0.00E+00	13.462	3.538	30.769	15.92
PA2402	-	Probable non-ribosomal peptide synthetase {peptide synthase}	13.7000	9.2000	17.0000	0.00E+00	0.00E+00	0.00E+00	13.700	9.200	17.000	13.30
PA2403	*fpvG*	Iron reductase FpvG	9.0000	18.8750	5.3750	1.08E-137	0.00E+00	4.64E-39	9.000	18.875	5.375	11.08
PA2404	*fpvH*	FpvH, related to iron release from ferripyoverdine	6.7500	7.7500	2.9167	1.10E-54	2.42E-76	1.34E-06	6.750	7.750	2.917	5.81
PA2405	*fpvJ*	FpvJ, related to iron release from ferripyoverdine	3.8750	6.5000	4.8750	2.70E-14	5.15E-47	3.22E-22	3.875	6.500	4.875	5.08
PA2406	*fpvK*	FpvK, related to iron release from ferripyoverdine	14.2500	21.5000	7.2500	1.89E-139	0.00E+00	1.66E-27	14.250	21.500	7.250	14.33
PA2407	*fpvC*	Fe^{3+} iron-chelating, metal-binding protein FpvC {adhesion protein}	5.8182	15.3636	2.0909	6.34E-54	0.00E+00	3.29E-04	5.818	15.364	2.091	7.76
PA2408	*fpvD*	FpvD permease, probable ATP-binding component of ABC transporter	2.4286	7.7143	3.2857	6.24E-05	1.75E-59	4.70E-07	2.429	7.714	3.286	4.48
PA2409	*fpvE*	FpvE ATPase {ABC transporter permease}	4.8000	9.2000	3.6000	6.97E-18	3.94E-81	5.47E-09	4.800	9.200	3.600	5.87
PA2410	*fpvF*	FpvF ferrisiderophore binding protein	2.8667	5.6000	2.1333	1.05E-12	1.44E-59	6.26E-05	2.867	5.600	2.133	3.53
PA2411	-	Probable thioesterase	13.3478	13.4348	21.5217	0.00E+00	0.00E+00	0.00E+00	13.348	13.435	21.522	16.10
PA2412	-	Conserved hypothetical protein	31.1481	18.8519	48.6296	0.00E+00	0.00E+00	0.00E+00	31.148	18.852	48.630	32.88
PA2413	*pvdH*	L-2,4-Diaminobutyrate:2-ketoglutarate 4-aminotransferase, PvdH {diaminobutyrate--2-oxoglutarate aminotransferase}	13.6875	10.8125	14.8125	0.00E+00	0.00E+00	0.00E+00	13.688	10.813	14.813	13.10
PA2424	*pvdL*	Non-ribosomal peptide synthase PvdL {peptide synthase}	24.1667	28.1667	23.8333	0.00E+00	0.00E+00	0.00E+00	24.167	28.167	23.833	25.39

PA2425	*pvdG*	Pyoverdine biosynthesis protein PvdG	29.0000	15.0000	70.0000	0.00E+00	5.43E-83	0.00E+00	29.000	15.000	70.000	38.00
PA2426	*pvdS*	Iron-starvation sigma factor PvdS {ECF sigma-70 factor}	8.3333	6.1111	17.6667	5.65E-81	4.84E-40	0.00E+00	8.333	6.111	17.667	10.70
PA2427	-	Hypothetical protein	12.2500	2.0000	104.0000	5.73E-87	2.67E-02	0.00E+00	12.250	2.000	104.000	39.42
PA2428	-	Hypothetical protein	22.0000	11.5000	6.0000	4.81E-286	1.32E-64	9.70E-13	22.000	11.500	6.000	13.17
PA2431	-	Hypothetical protein	0.1765	0.1176	0.3529	2.05E-02	8.59E-04	1.25E-06	-5.667	-8.500	-2.833	-5.67
PA2437	-	Hypothetical protein	18.0000	3.0000	14.0000	2.85E-111	2.93E-03	1.21E-56	18.000	3.000	14.000	11.67
PA2438	-	Hypothetical protein	24.0000	14.0000	21.0000	5.71E-147	3.07E-42	3.38E-106	24.000	14.000	21.000	19.67
PA2439	-	Hypothetical protein	4.5000	3.7500	5.2500	8.79E-23	1.19E-15	4.74E-29	4.500	3.750	5.250	4.50
PA2440	-	Hypothetical protein	2.8571	5.1429	4.1429	1.10E-07	9.14E-28	1.20E-14	2.857	5.143	4.143	4.05
PA2446	*gcvH2*	Glycine cleavage system protein H2	5.4745	4.2869	1.3512	3.56E-278	9.25E-156	5.59E-08	5.475	4.287	1.351	3.70
PA2447	-	Probable transcriptional regulator	2.0000	3.0000	4.5000	3.22E-03	3.99E-06	3.35E-14	2.000	3.000	4.500	3.17
PA2450	-	Hypothetical protein	3.9375	4.1875	3.5000	1.99E-29	8.05E-34	1.08E-20	3.938	4.188	3.500	3.88
PA2458	-	Hypothetical protein	0.1765	0.0196	0.5490	7.60E-04	0.00E+00	6.79E-03	-5.667	-51.000	-1.821	-19.50
PA2464	-	Hypothetical protein	5.9286	5.0714	3.6667	2.61E-100	6.40E-69	8.20E-30	5.929	5.071	3.667	4.89
PA2470	*gtdA*	Gentisate 1,2-dioxygenase	3.6000	2.8000	2.2000	5.83E-11	8.06E-07	6.94E-03	3.600	2.800	2.200	2.87
PA2471	-	Conserved hypothetical protein	2.8333	2.6667	3.5000	5.83E-07	2.05E-06	3.02E-09	2.833	2.667	3.500	3.00
PA2473	-	Maleylpyruvate isomerase {glutathione *S*-transferase}	2.5000	4.0000	3.5000	1.62E-02	2.22E-05	1.94E-04	2.500	4.000	3.500	3.33
PA2481	-	Hypothetical protein	3.3696	3.1522	7.9130	1.92E-40	3.32E-34	7.54E-300	3.370	3.152	7.913	4.81
PA2482	-	Probable cytochrome C	4.4464	11.3929	5.6250	4.90E-70	0.00E+00	2.35E-119	4.446	11.393	5.625	7.15
PA2485		Hypothetical protein	2.7963	7.4815	1.7407	9.95E-14	9.00E-133	3.31E-03	2.796	7.481	1.741	4.01
PA2486	*ptrC*	Pseudomonas type III repressor gene C, PtrC	2.3310	4.8169	3.0634	9.70E-14	2.70E-72	9.48E-23	2.331	4.817	3.063	3.40
PA2487	-	Hypothetical protein	1.5714	6.4286	5.4286	2.04E-02	3.60E-38	2.70E-24	1.571	6.429	5.429	4.48
PA2489	-	Probable transcriptional regulator	2.6000	4.0000	2.0000	1.08E-07	5.53E-20	2.34E-03	2.600	4.000	2.000	2.87
PA2496	-	Hypothetical protein	1.8750	3.2500	2.1250	2.60E-03	1.18E-08	5.59E-03	1.875	3.250	2.125	2.42
PA2501	-	Hypothetical protein	1.0780	3.9740	1.2411	3.36E-05	1.43E-89	1.09E-03	1.078	3.974	1.241	2.10
PA2507	*catA*	Catechol 1,2-dioxygenase	6.0000	7.0000	3.5000	7.51E-13	6.94E-19	1.29E-04	6.000	7.000	3.500	5.50
PA2510	*catR*	Transcriptional regulator CatR	2.5833	1.7500	2.7500	1.50E-08	2.57E-04	9.20E-08	2.583	1.750	2.750	2.36
PA2525	*opmB*	OpmB	0.1333	0.1667	0.5000	1.53E-05	1.45E-03	1.88E-03	-7.500	-6.000	-2.000	-5.17
PA2531	-	Probable aminotransferase	7.1667	4.3333	3.0000	1.09E-65	1.22E-20	7.38E-08	7.167	4.333	3.000	4.83
PA2537	-	Probable acyltransferase	1.3922	1.0196	3.6471	3.51E-05	1.21E-02	7.58E-38	1.392	1.020	3.647	2.02
PA2538	-	Hypothetical protein	2.4000	1.1500	4.4500	4.42E-07	3.47E-02	1.54E-25	2.400	1.150	4.450	2.67
PA2540	-	Conserved hypothetical protein	16.0238	9.7143	7.6667	0.00E+00	0.00E+00	0.00E+00	16.024	9.714	7.667	11.13
PA2541	-	Probable CDP-alcohol phosphatidyltransferase	6.8478	3.4130	5.4783	1.30E-170	5.56E-33	7.61E-98	6.848	3.413	5.478	5.25
PA2550	-	Probable acyl-CoA dehydrogenase	3.7143	7.2143	3.5714	1.53E-28	3.85E-136	1.94E-23	3.714	7.214	3.571	4.83
PA2560	-	Hypothetical protein	1.1014	1.2754	3.6812	7.65E-03	2.46E-03	6.48E-28	1.101	1.275	3.681	2.02
PA2562	-	Hypothetical protein	2.3033	1.4699	4.5765	4.63E-38	4.62E-13	2.89E-190	2.303	1.470	4.577	2.78
PA2564	*[tam]*	Hypothetical protein {trans-aconitate 2-methyltransferase}	0.0127	0.0063	0.0633	0.00E+00	0.00E+00	0.00E+00	-79.000	-158.000	-15.800	-84.27
PA2565	-	Hypothetical protein	0.0583	0.0667	0.0417	2.83E-34	7.58E-23	0.00E+00	-17.143	-15.000	-24.000	-18.71
PA2566	-	Hypothetical protein	0.0167	0.0722	0.0333	0.00E+00	1.27E-50	0.00E+00	-60.000	-13.846	-30.000	-34.62
PA2567	-	Hypothetical protein	4.1087	4.3152	2.7065	2.38E-146	5.38E-164	1.08E-52	4.109	4.315	2.707	3.71
PA2578	-	Probable acetyltransferase	0.1489	0.0213	0.3830	5.52E-04	0.00E+00	9.64E-07	-6.714	-47.000	-2.611	-18.78
PA2593	*qteE*	Quorum threshold expression element, QteE	0.1458	0.1875	0.2708	5.73E-05	1.48E-02	1.46E-11	-6.857	-5.333	-3.692	-5.29
PA2603.1	-	tRNA-Ser	7.0000	7.0000	3.0000	1.69E-06	2.80E-06	2.35E-02	7.000	7.000	3.000	5.67
PA2622	*cspD*	Cold-shock protein CspD	0.5268	0.2490	0.9116	1.27E-02	1.60E-04	1.00E-04	-1.898	-4.016	-1.097	-2.34
PA2624	*idh*	Isocitrate dehydrogenase	8.0000	10.4957	7.7304	0.00E+00	0.00E+00	0.00E+00	8.000	10.496	7.730	8.74
PA2625	-	Conserved hypothetical protein	8.0667	9.0667	9.6667	3.05E-84	6.72E-111	3.12E-125	8.067	9.067	9.667	8.93
PA2650	*[ybaJ]*	Conserved hypothetical protein	4.0769	1.3846	1.7692	1.10E-22	6.93E-03	1.77E-02	4.077	1.385	1.769	2.41
PA2652	-	Methyl-accepting chemotaxis protein	2.8793	3.1379	1.4138	1.27E-49	4.16E-60	3.99E-07	2.879	3.138	1.414	2.48
PA2654	*tlpQ*	Probable chemotaxis transducer	3.4286	2.8776	3.0408	1.94E-79	1.41E-52	3.45E-58	3.429	2.878	3.041	3.12
PA2666	*[ptpS]*	Probable 6-pyruvoyl tetrahydrobiopterin synthase {6-carboxytetrahydropterin synthase QueD}	2.6875	5.3125	1.9375	3.57E-07	1.19E-31	1.58E-02	2.688	5.313	1.938	3.31
PA2667	*mvaU*	Transcriptional regulator MvaU	2.0097	4.7143	2.4092	9.78E-27	2.11E-197	7.69E-39	2.010	4.714	2.409	3.04
PA2684	*tse5*	T6SS effector protein Tse5	2.3529	1.3824	3.4412	1.17E-35	2.93E-10	6.11E-84	2.353	1.382	3.441	2.39

PA2685	*vgrG4 [vgrG1c]*	T6SS protein VgrG4	3.0303	1.9697	4.0000	7.16E-48	2.32E-17	1.14E-89	3.030	1.970	4.000	3.00
PA2702	*tse2*	T6SS effector protein Tse2	11.6111	13.1111	3.7222	4.60E-246	0.00E+00	1.79E-15	11.611	13.111	3.722	9.48
PA2711	*[potF4]*	Probable periplasmic spermidine/putrescine-binding protein	1.0000	1.1579	4.6316	1.58E-02	6.73E-03	2.73E-50	1.000	1.158	4.632	2.26
PA2718	-	Probable transcriptional regulator	2.1163	2.7209	2.1395	2.01E-09	4.18E-16	4.48E-07	2.116	2.721	2.140	2.33
PA2720	-	Hypothetical protein	2.6296	1.5926	1.9259	1.86E-12	1.23E-04	2.92E-04	2.630	1.593	1.926	2.05
PA2724	-	Hypothetical protein	3.0000	4.0000	3.0000	5.08E-04	1.25E-06	6.91E-04	3.000	4.000	3.000	3.33
PA2742	*rpmI*	50S ribosomal protein L35	0.4523	0.0772	0.8625	2.97E-02	9.23E-89	7.51E-05	-2.211	-12.946	-1.159	-5.44
{PA2763a}	-	Intergenic region {YP_008719761.1 hypothetical protein PA2763a}	0.1471	0.2059	0.2059	3.76E-05	4.39E-02	3.23E-09	-6.800	-4.857	-4.857	-5.50
PA2769	-	Hypothetical protein	0.1077	0.0615	0.2462	1.71E-07	1.71E-25	1.41E-15	-9.286	-16.250	-4.063	-9.87
PA2776	*pauB3*	FAD-dependent oxidoreductase	16.7059	11.4510	13.9020	0.00E+00	0.00E+00	0.00E+00	16.706	11.451	13.902	14.02
PA2796	*tal*	Transaldolase B	2.1818	2.9949	1.0202	1.59E-33	5.71E-70	1.96E-03	2.182	2.995	1.020	2.07
PA2801	-	Hypothetical protein	0.1695	0.0508	0.3390	4.02E-03	1.07E-40	8.36E-08	-5.900	-19.667	-2.950	-9.51
PA2814	-	Hypothetical protein	3.5185	7.2222	2.2963	5.90E-26	1.11E-139	9.14E-08	3.519	7.222	2.296	4.35
PA2834	-	Probable transcriptional regulator	2.0000	1.1875	2.8125	2.02E-06	1.09E-02	9.19E-11	2.000	1.188	2.813	2.00
PA2840	*[deaD]*	Probable ATP-dependent RNA helicase	2.6000	3.4000	2.9000	4.72E-13	1.52E-23	1.63E-14	2.600	3.400	2.900	2.97
PA2857	-	Probable ATP-binding component of ABC transporter	2.6667	3.2778	2.1111	1.91E-10	4.61E-16	2.22E-04	2.667	3.278	2.111	2.69
PA2862	*lipA*	Lactonizing lipase precursor	5.9444	25.8889	3.9444	1.20E-82	0.00E+00	7.37E-30	5.944	25.889	3.944	11.93
PA2869	-	Hypothetical protein	2.8333	5.1111	1.9444	2.68E-09	6.46E-36	6.99E-03	2.833	5.111	1.944	3.30
PA2873	*tgpA*	Transglutaminase protein A, TgpA {protein-glutamine gamma-glutamyltransferase}	0.0909	0.1364	0.2727	2.92E-07	1.33E-05	3.37E-16	-11.000	-7.333	-3.667	-7.33
PA2888	*atuC*	Geranyl-CoA carboxylase subunit beta	0.0909	0.2121	0.1212	6.91E-12	3.36E-02	6.45E-96	-11.000	-4.714	-8.250	-7.99
PA2897	-	Probable transcriptional regulator	1.8963	2.0519	2.8815	1.35E-24	3.09E-29	5.23E-63	1.896	2.052	2.881	2.28
PA2907	*cobL [cbiE, cbiT]*	Precorrin-6y-dependent methyltransferase CobL	1.7500	2.5833	2.0000	7.79E-05	1.29E-10	2.93E-04	1.750	2.583	2.000	2.11
PA2912	-	Probable ATP-binding component of ABC transporter	2.6667	11.3333	3.3333	1.14E-04	1.78E-100	2.21E-06	2.667	11.333	3.333	5.78
PA2918	-	Probable short-chain dehydrogenase	1.7241	1.2414	3.1724	1.60E-06	2.19E-03	1.40E-21	1.724	1.241	3.172	2.05
PA2939	*[pepB]*	Probable aminopeptidase [PepB]	0.0269	0.0457	0.0134	0.00E+00	0.00E+00	0.00E+00	-37.200	-21.882	-74.400	-44.49
PA2942.1	*P15*	P15 (ncRNC)	23.4286	41.2857	3.5714	0.00E+00	0.00E+00	1.38E-06	23.429	41.286	3.571	22.76
PA2946	-	Hypothetical protein	2.3939	2.2576	2.4242	2.41E-20	1.84E-17	2.84E-18	2.394	2.258	2.424	2.36
PA2951	*etfA*	Electron transfer flavoprotein subunit alpha	0.4756	0.2875	0.3920	2.30E-02	3.01E-02	3.24E-02	-2.103	-3.478	-2.551	-2.71
PA2956	-	Conserved hypothetical protein	0.1957	0.0870	0.4565	9.82E-03	2.69E-13	9.63E-05	-5.111	-11.500	-2.190	-6.27
PA2957	-	Probable transcriptional regulator	0.1811	0.1496	0.3858	3.46E-03	2.16E-06	1.23E-08	-5.522	-6.684	-2.592	-4.93
PA2975	*rluC [yceC]*	Ribosomal large subunit pseudouridine synthase C	0.7324	0.2113	2.4789	1.46E-02	4.45E-03	2.58E-26	-1.365	-4.733	-0.403	-2.17
PA2978	*ptpA*	Phosphotyrosine protein phosphatase	0.0805	0.0345	0.5632	4.54E-16	1.84E-70	8.32E-03	-12.429	-29.000	-1.776	-14.40
PA3001	-	Probable glyceraldehyde-3-phosphate dehydrogenase	0.4656	0.2667	0.3386	3.21E-02	4.17E-03	5.14E-04	-2.148	-3.750	-2.953	-2.95
PA3018	-	Hypothetical protein	1.4138	2.7241	3.0690	3.97E-04	2.26E-15	5.07E-18	1.414	2.724	3.069	2.40
PA3021	-	Hypothetical protein	4.8632	5.9829	1.3077	3.90E-166	2.39E-271	3.40E-05	4.863	5.983	1.308	4.05
PA3024	-	Probable carbohydrate kinase	1.8750	1.8333	3.3750	3.34E-10	2.29E-09	1.08E-34	1.875	1.833	3.375	2.36
PA3025	*[glpD2]*	Probable FAD-dependent glycerol-3-phosphate dehydrogenase	1.4706	1.2941	4.8235	3.23E-05	6.80E-04	9.07E-65	1.471	1.294	4.824	2.53
PA3026	-	Conserved hypothetical protein	4.4783	4.7826	8.0870	5.94E-72	2.81E-84	2.43E-292	4.478	4.783	8.087	5.78
PA3030	*mobA*	Molybdopterin-guanine dinucleotide biosynthesis protein MobA {molybdenum cofactor guanylyltransferase}	3.5000	4.0000	2.1667	2.79E-08	1.48E-11	1.20E-02	3.500	4.000	2.167	3.22
PA3031	-	Hypothetical protein	0.7768	0.2328	2.0567	5.21E-06	6.08E-06	4.05E-47	-1.287	-4.296	-0.486	-2.02
PA3032	*snr1*	Cytochrome c Snr1	0.0485	0.0303	0.0545	1.00E-75	2.23E-201	0.00E+00	-20.625	-33.000	-18.333	-23.99
PA3038	*opdQ [occK6]*	Porin OpdQ	10.0928	13.7010	12.0412	0.00E+00	0.00E+00	0.00E+00	10.093	13.701	12.041	11.95
PA3041	*[yqjE]*	Hypothetical protein	0.1267	0.1050	0.4455	2.01E-08	1.19E-23	2.60E-04	-7.891	-9.528	-2.244	-6.55
PA3042	-	Hypothetical protein	0.0389	0.1625	0.3781	6.07E-108	3.78E-06	3.03E-09	-25.727	-6.152	-2.645	-11.51
PA3043	-	Conserved hypothetical protein {deoxyguanosinetriphosphate triphosphohydrolase}	1.9444	3.1481	1.8519	3.68E-17	3.10E-51	1.76E-12	1.944	3.148	1.852	2.31
PA3053	-	Probable hydrolytic enzyme {hydrolase}	3.5417	4.0000	1.6667	2.81E-32	3.43E-42	9.21E-04	3.542	4.000	1.667	3.07
PA3064	*pelA*	PelA protein	2.0000	2.5000	3.5000	6.18E-04	8.51E-05	1.15E-08	2.000	2.500	3.500	2.67
PA3068	*gdhB*	NAD-dependent glutamate dehydrogenase	0.2813	0.2447	0.1734	4.61E-02	1.07E-07	3.67E-42	-3.555	-4.087	-5.767	-4.47
PA3069	-	Hypothetical protein	0.1667	0.1667	0.3333	4.96E-03	2.19E-03	7.63E-07	-6.000	-6.000	-3.000	-5.00

PA3079	-	Hypothetical protein	1.4643	3.4643	3.4286	2.37E-09	5.24E-61	6.96E-58	1.464	3.464	3.429	2.79
PA3081	-	Conserved hypothetical protein	17.0000	20.4286	4.3810	0.00E+00	0.00E+00	1.66E-53	17.000	20.429	4.381	13.94
PA3082	*gbt*	Glycine betaine transmethylase	16.4091	25.3636	4.4545	0.00E+00	0.00E+00	3.10E-74	16.409	25.364	4.455	15.41
PA3084	-	Hypothetical protein	2.4872	2.9103	1.1410	2.32E-27	1.28E-38	2.13E-02	2.487	2.910	1.141	2.18
PA3085	-	Hypothetical protein	1.9744	1.5513	2.8333	5.22E-08	6.67E-05	5.43E-15	1.974	1.551	2.833	2.12
PA3092	*fadH1*	2,4-Dienoyl-CoA reductase FadH1	8.5000	8.4500	1.3000	0.00E+00	0.00E+00	1.18E-02	8.500	8.450	1.300	6.08
PA3096	*xcpY*	General secretion pathway protein L {T2SS pathway protein L}	0.2027	0.0405	0.4189	1.07E-02	1.80E-88	1.36E-06	-4.933	-24.667	-2.387	-10.66
PA3097	*xcpX*	General secretion pathway protein K {T2SS pathway protein K}	0.0513	0.1538	0.3077	7.35E-54	8.06E-04	4.89E-12	-19.500	-6.500	-3.250	-9.75
PA3098	*xcpW [pddD]*	General secretion pathway protein J {T2SS pathway protein J}	0.1026	0.0256	0.2308	5.34E-13	1.96E-203	1.37E-26	-9.750	-39.000	-4.333	-17.69
PA3117	*asd*	Aspartate-semialdehyde dehydrogenase	10.7130	3.8870	1.4261	0.00E+00	1.32E-116	9.98E-09	10.713	3.887	1.426	5.34
PA3118	*leuB*	3-Isopropylmalate dehydrogenase	30.5181	6.6386	1.9518	0.00E+00	0.00E+00	9.23E-17	30.518	6.639	1.952	13.04
PA3119	*[yafE]*	Conserved hypothetical protein	25.3462	3.1154	3.3846	0.00E+00	5.38E-21	7.01E-23	25.346	3.115	3.385	10.62
PA3120	*leuD*	3-Isopropylmalate dehydratase small subunit	23.1842	2.6711	3.3289	0.00E+00	1.54E-26	3.30E-42	23.184	2.671	3.329	9.73
PA3121	*leuC*	3-Isopropylmalate dehydratase large subunit	23.5060	5.1687	2.3373	0.00E+00	5.03E-221	7.28E-32	23.506	5.169	2.337	10.34
PA3126	*ibpA [hslT]*	Heat-shock protein IbpA	0.1834	0.0718	0.0285	1.11E-02	5.92E-90	0.00E+00	-5.453	-13.928	-35.030	-18.14
PA3127	-	Hypothetical protein	2.4872	1.3846	2.6410	7.85E-17	7.73E-05	9.75E-17	2.487	1.385	2.641	2.17
PA3134	*gltX*	Glutamyl-tRNA synthetase {glutamate--tRNA ligase}	0.5367	0.2599	0.4463	2.42E-02	2.33E-03	8.85E-04	-1.863	-3.848	-2.241	-2.65
PA3135	-	Probable transcriptional regulator	2.9231	2.0000	1.9231	2.84E-12	9.21E-06	2.56E-03	2.923	2.000	1.923	2.28
PA3139	*[aspC, tyrB]*	Probable amino acid aminotransferase {aspartate aminotransferase}	4.7542	5.9385	1.2737	3.76E-222	0.00E+00	3.31E-08	4.754	5.939	1.274	3.99
PA3146	*wbpK*	Probable NAD-dependent epimerase/dehydratase WbpK	0.0694	0.0694	0.2083	3.58E-38	4.12E-49	1.09E-48	-14.400	-14.400	-4.800	-11.20
PA3147	*wbpJ*	Probable glycosyltransferase WbpJ	0.0858	0.0901	0.2060	4.72E-23	1.86E-38	2.23E-47	-11.650	-11.095	-4.854	-9.20
PA3148	*wbpI*	UDP-*N*-acetylglucosamine 2-epimerase WbpI {UDP-2,3-diacetamido-2,3-dideoxy-D-glucuronate 2-epimerase}	0.1012	0.0568	0.1474	3.21E-14	1.39E-150	6.91E-105	-9.877	-17.594	-6.783	-11.42
PA3149	*wbpH*	Probable glycosyltransferase WbpH	0.0602	0.0402	0.1968	4.27E-56	5.85E-156	9.40E-55	-16.600	-24.900	-5.082	-15.53
PA3150	*wbpG*	LPS biosynthesis protein WbpG	0.1823	0.1302	0.1953	9.68E-03	1.74E-23	1.20E-51	-5.486	-7.680	-5.120	-6.10
PA3151	*hisF2*	Imidazoleglycerol-phosphate synthase, cyclase subunit HisF2	0.0900	0.0500	0.1533	4.12E-22	1.06E-96	6.62E-106	-11.111	-20.000	-6.522	-12.54
PA3152	*hisH2*	Glutamine amidotransferase HisH2 {imidazole glycerol phosphate synthase subunit HisH}	0.1342	0.0805	0.2081	4.31E-07	6.86E-24	3.78E-42	-7.450	-12.417	-4.806	-8.22
PA3153	*wzx [wbpF, rfbX]*	O-antigen translocase	0.1273	0.0727	0.3091	2.34E-08	3.81E-19	3.33E-15	-7.857	-13.750	-3.235	-8.28
PA3154	*wzy [rfc]*	B-band O-antigen polymerase	0.0952	0.0119	0.3929	4.43E-14	0.00E+00	1.90E-08	-10.500	-84.000	-2.545	-32.35
PA3156	*wbpD*	UDP-2-acetamido-3-amino-2,3-dideoxy-D-glucuronic acid *N*-acetyltransferase WbpD {UDP-2-acetamido-2-deoxy-3-oxo-D-glucuronate aminotransferase}	0.2040	0.1893	0.4540	4.28E-02	1.40E-08	4.18E-03	-4.901	-5.282	-2.202	-4.13
PA3170	-	Conserved hypothetical protein {*N*-ethylammeline chlorohydrolase}	2.5238	3.0952	2.2381	6.42E-27	2.64E-42	2.22E-17	2.524	3.095	2.238	2.62
PA3175	*hutE*	Formimidoylglutamase HutE	5.6667	4.3333	9.3333	1.50E-16	1.83E-09	1.65E-51	5.667	4.333	9.333	6.44
PA3181	*[edaA]*	2-Keto-3-deoxy-6-phosphogluconate aldolase {2-dehydro-3-deoxy-phosphogluconate aldolase}	6.1538	5.9487	2.9231	5.83E-123	6.54E-113	5.00E-19	6.154	5.949	2.923	5.01
PA3182	*pgl*	6-Phosphogluconolactonase	9.7742	6.6774	3.2258	0.00E+00	1.47E-133	6.95E-22	9.774	6.677	3.226	6.56
PA3183	*zwf*	Glucose-6-phosphate 1-dehydrogenase	6.9167	5.9375	4.4583	0.00E+00	5.67E-233	4.88E-116	6.917	5.938	4.458	5.77
PA3186	*oprB*	Glucose/carbohydrate outer membrane porin OprB precursor {porin B}	1.7980	3.8485	7.8182	4.57E-20	2.30E-116	0.00E+00	1.798	3.848	7.818	4.49
PA3187	*[gltK]*	Probable ATP-binding component of ABC transporter	3.5556	2.6667	7.2222	1.24E-53	3.42E-27	1.23E-287	3.556	2.667	7.222	4.48
PA3194	*edd*	Phosphogluconate dehydratase	9.5870	7.1087	5.1304	0.00E+00	0.00E+00	4.83E-177	9.587	7.109	5.130	7.28
PA3195	*gapA*	Glyceraldehyde 3-phosphate dehydrogenase	3.7200	2.3000	3.3600	1.33E-59	2.17E-19	1.13E-44	3.720	2.300	3.360	3.13
PA3196	-	Hypothetical protein	2.4839	2.7419	1.5806	6.33E-11	9.60E-14	1.69E-02	2.484	2.742	1.581	2.27
PA3205	-	Hypothetical protein	1.1856	5.1237	1.2990	1.46E-04	1.25E-110	1.18E-02	1.186	5.124	1.299	2.54
PA3220	-	Probable transcriptional regulator	3.7222	3.9444	2.6667	1.72E-24	2.11E-28	1.90E-09	3.722	3.944	2.667	3.44
PA3229	-	Hypothetical protein	0.0528	0.0310	0.1720	3.71E-76	6.30E-238	2.27E-83	-18.957	-32.296	-5.813	-19.02
PA3230	-	Hypothetical protein	0.1648	0.1319	0.4945	3.09E-04	5.96E-09	1.22E-03	-6.067	-7.583	-2.022	-5.22
PA3233	-	Hypothetical protein	8.9333	5.0667	31.8000	2.32E-307	1.69E-79	0.00E+00	8.933	5.067	31.800	15.27
PA3234	*[yjcG]*	Probable sodium:solute symporter {acetate permease}	10.6216	8.9730	32.7297	0.00E+00	0.00E+00	0.00E+00	10.622	8.973	32.730	17.44

PA3235	*[yjcH]*	Conserved hypothetical protein	9.4625	7.7875	30.6875	0.00E+00	5.02E-211	0.00E+00	9.463	7.788	30.688	15.98
PA3238	-	Hypothetical protein	4.9744	4.8462	1.4103	3.67E-121	2.59E-113	3.33E-04	4.974	4.846	1.410	3.74
PA3262	-	Probable peptidyl-prolyl cis-trans isomerase, FkbP-type	3.7523	3.0093	1.3529	5.73E-141	1.18E-83	2.12E-11	3.752	3.009	1.353	2.70
PA3262.1	-	tRNA-Asp	1.4419	3.4419	4.0930	4.76E-02	4.34E-09	5.87E-12	1.442	3.442	4.093	2.99
PA3267	-	Hypothetical protein	3.1818	1.0682	3.3636	5.52E-57	1.56E-04	2.61E-63	3.182	1.068	3.364	2.54
PA3272	*[lhr]*	Probable ATP-dependent DNA helicase	0.1429	0.0476	0.5238	1.52E-04	3.77E-64	4.84E-02	-7.000	-21.000	-1.909	-9.97
PA3283	-	Conserved hypothetical protein	21.6000	9.5000	21.6000	0.00E+00	6.56E-135	0.00E+00	21.600	9.500	21.600	17.57
PA3284	-	Hypothetical protein	5.1707	1.3415	14.3415	5.18E-50	4.06E-03	0.00E+00	5.171	1.341	14.341	6.95
PA3305.1	*phrS*	PhrS	2.7501	1.4987	3.4767	1.55E-102	1.05E-24	2.14E-178	2.750	1.499	3.477	2.58
PA3307	-	Hypothetical protein	1.8542	4.4167	2.2708	8.56E-06	4.71E-38	1.86E-06	1.854	4.417	2.271	2.85
PA3313	-	Hypothetical protein	0.8239	0.9773	0.1307	2.01E-04	8.48E-05	3.83E-140	-1.214	-1.023	-7.652	-3.30
PA3316	-	Probable ABC transporter permease	0.1000	0.0500	0.2500	2.08E-11	6.17E-25	6.86E-17	-10.000	-20.000	-4.000	-11.33
PA3326	*clpP2*	Probable ATP-dependent Clp protease proteolytic subunit ClpP2	0.1004	0.0521	0.1936	1.68E-14	2.80E-259	4.40E-38	-9.957	-19.176	-5.165	-11.43
PA3327	-	Probable non-ribosomal peptide synthetase	0.0268	0.0179	0.0134	0.00E+00	0.00E+00	0.00E+00	-37.333	-56.000	-74.667	-56.00
PA3328	-	Probable FAD-dependent monooxygenase	0.0125	0.0031	0.0031	0.00E+00	0.00E+00	0.00E+00	-79.750	-319.000	-319.000	-239.25
PA3329	-	Hypothetical protein	0.0149	0.0348	0.0149	0.00E+00	1.07E-204	0.00E+00	-67.000	-28.714	-67.000	-54.24
PA3330	-	Probable short-chain dehydrogenase	0.0174	0.0029	0.0029	0.00E+00	0.00E+00	0.00E+00	-57.333	-344.000	-344.000	-248.44
PA3331	-	Cytochrome P450	0.0233	0.0769	0.0070	0.00E+00	4.21E-73	0.00E+00	-42.900	-13.000	-143.000	-66.30
PA3332	-	Conserved hypothetical protein	0.0207	0.0385	0.0030	0.00E+00	7.53E-179	0.00E+00	-48.286	-26.000	-338.000	-137.43
PA3333	*fabH2*	3-Oxoacyl-[acyl-carrier-protein] synthase III	0.0249	0.0673	0.0125	0.00E+00	4.50E-86	0.00E+00	-40.100	-14.852	-80.200	-45.05
PA3334	*acp3*	Probable acyl carrier protein Acp3	0.0182	0.0024	0.0146	0.00E+00	0.00E+00	0.00E+00	-54.933	-412.000	-68.667	-178.53
PA3335	-	Hypothetical protein	0.0029	0.0434	0.0231	0.00E+00	6.70E-132	0.00E+00	-346.000	-23.067	-43.250	-137.44
PA3351	*flgM*	FlgM	1.2316	4.5093	1.9978	2.93E-12	1.52E-242	1.31E-33	1.232	4.509	1.998	2.58
PA3355	-	Hypothetical protein	2.2564	1.7436	2.0000	4.09E-19	2.01E-10	1.22E-11	2.256	1.744	2.000	2.00
PA3356	*pauA5*	Glutamylpolyamine synthetase	5.3147	4.3916	4.7972	1.38E-272	1.78E-173	3.48E-212	5.315	4.392	4.797	4.83
PA3362	*[amiS]*	Hypothetical protein {transporter protein AmiS}	0.0233	0.1318	0.1163	9.54E-266	8.76E-08	5.24E-110	-43.000	-7.588	-8.600	-19.73
PA3363	*amiR*	Aliphatic amidase regulator	0.0879	0.0502	0.1004	4.67E-22	1.08E-72	8.40E-220	-11.381	-19.917	-9.958	-13.75
PA3364	*amiC*	Aliphatic amidase expression-regulating protein	0.1302	0.0651	0.1006	1.67E-07	2.16E-50	3.48E-234	-7.682	-15.364	-9.941	-11.00
PA3365	*[amiB]*	Probable chaperone	0.0232	0.0232	0.0695	0.00E+00	0.00E+00	0.00E+00	-43.167	-43.167	-14.389	-33.57
PA3366	*amiE*	Aliphatic amidase {acylamide amidohydrolase}	0.0351	0.0129	0.0738	7.66E-205	0.00E+00	0.00E+00	-28.526	-77.429	-13.550	-39.83
PA3372	*[phnP]*	Conserved hypothetical protein {carbon-phosphorus lyase complex accessory protein}	0.1190	0.0476	0.4048	5.42E-07	1.84E-54	1.45E-05	-8.400	-21.000	-2.471	-10.62
PA3380	*[phnG]*	Conserved hypothetical protein	3.0000	4.0000	3.0000	2.75E-02	3.31E-03	1.33E-02	3.000	4.000	3.000	3.33
PA3387	*rhlG*	Beta-ketoacyl reductase {3-oxoacyl-ACP reductase}	8.2500	13.2500	3.0000	2.87E-45	2.20E-139	2.17E-04	8.250	13.250	3.000	8.17
PA3388	*[yaeB]*	Conserved hypothetical protein {*S*-adenosylmethionine-dependent methyltransferase RcsF}	3.0000	5.0000	2.0000	6.21E-07	1.36E-20	8.21E-03	3.000	5.000	2.000	3.33
PA3390	-	Hypothetical protein	4.0000	4.5000	4.5000	9.89E-05	2.15E-06	5.22E-06	4.000	4.500	4.500	4.33
PA3407	*hasAp*	Heme acquisition protein HasAp	6.3143	11.5429	19.6286	4.86E-112	0.00E+00	0.00E+00	6.314	11.543	19.629	12.50
PA3413	*[yebG]*	Conserved hypothetical protein	3.4800	5.2160	1.5920	5.06E-38	7.27E-98	3.11E-04	3.480	5.216	1.592	3.43
PA3418	*ldh*	Leucine dehydrogenase	0.1981	0.7150	0.5121	2.03E-02	4.12E-02	2.30E-02	-5.049	-1.399	-1.953	-2.80
PA3427	-	Probable short-chain dehydrogenase	8.6071	20.0000	3.9286	3.36E-270	0.00E+00	1.90E-40	8.607	20.000	3.929	10.85
PA3430	-	Probable aldolase	2.6667	3.4762	2.8810	2.40E-20	2.41E-37	1.19E-21	2.667	3.476	2.881	3.01
PA3437	*folM [ydgB]*	Dihydromonapterin reductase, FolM {short-chain dehydrogenase}	4.1111	7.0000	1.4444	1.60E-37	5.80E-131	3.67E-02	4.111	7.000	1.444	4.19
PA3438	*folE1*	GTP cyclohydrolase I precursor	3.0870	4.2609	1.4783	2.97E-31	4.93E-66	1.09E-03	3.087	4.261	1.478	2.94
PA3439	*folX*	D-Erythro-7,8-dihydroneopterin triphosphate epimerase	6.3158	12.6754	1.7895	2.58E-182	0.00E+00	2.69E-07	6.316	12.675	1.789	6.93
PA3440	-	Conserved hypothetical protein	2.4848	4.4621	1.2955	4.10E-20	2.22E-77	1.45E-02	2.485	4.462	1.295	2.75
{PA3440a}	-	No gene {YP_008719767.1 type III effector protein}	2.8316	4.1474	1.3053	5.22E-23	9.64E-56	3.91E-02	2.832	4.147	1.305	2.76
PA3441	*[ssuF]*	Probable molybdopterin-binding protein	23.9867	1.1867	8.1600	0.00E+00	1.32E-03	5.09E-277	23.987	1.187	8.160	11.11
PA3444	*[ssuD]*	Conserved hypothetical protein {alkanesulfonate monooxygenase}	24.6667	1.8333	5.6667	0.00E+00	9.83E-06	3.14E-62	24.667	1.833	5.667	10.72
PA3445	-	Conserved hypothetical protein	10.2368	2.8947	1.2632	0.00E+00	6.27E-26	4.45E-02	10.237	2.895	1.263	4.80

PA3449	-	Conserved hypothetical protein	3.0000	4.5000	2.5000	5.41E-04	1.60E-08	3.98E-02	3.000	4.500	2.500	3.33
PA3452	*mqoA*	Malate:quinone oxidoreductase	2.8462	3.2308	3.8462	1.81E-26	5.71E-35	1.04E-50	2.846	3.231	3.846	3.31
PA3461	*[yhfE]*	Conserved hypothetical protein	1.1781	2.4932	2.6438	9.39E-07	1.47E-33	3.00E-36	1.178	2.493	2.644	2.11
PA3464	-	Hypothetical protein	6.5714	8.8571	6.7143	7.35E-58	1.97E-119	2.39E-60	6.571	8.857	6.714	7.38
PA3476	*rhlI*	Autoinducer synthesis protein RhlI {acyl-homoserine-lactone synthase}	0.1926	0.2905	0.0743	1.19E-02	3.04E-02	0.00E+00	-5.193	-3.442	-13.455	-7.36
PA3478	*rhlB*	Rhamnosyltransferase chain B (RhlB)	0.0217	0.0054	0.0326	0.00E+00	0.00E+00	0.00E+00	-46.000	-184.000	-30.667	-86.89
PA3479	*rhlA*	Rhamnosyltransferase chain A (RhlA)	0.0090	0.0225	0.0075	0.00E+00	0.00E+00	0.00E+00	-111.167	-44.467	-133.400	-96.34
PA3481	*[mrp]*	Conserved hypothetical protein	2.1915	1.7340	2.6064	2.61E-27	1.52E-15	9.19E-39	2.191	1.734	2.606	2.18
PA3483	*tse3*	T6SS effector protein Tse3	4.9070	2.4419	2.4419	1.50E-84	1.31E-16	2.54E-14	4.907	2.442	2.442	3.26
PA3484	*tsi3*	T6SS immunity protein Tsi3	19.7500	10.9500	4.7500	0.00E+00	0.00E+00	2.84E-58	19.750	10.950	4.750	11.82
PA3485	*vgrG4b*	T6SS protein VgrG4b	6.2857	2.1429	4.0476	3.29E-61	7.82E-06	8.45E-20	6.286	2.143	4.048	4.16
PA3493	*[rnfG]*	Conserved hypothetical protein {electron transport complex subunit G}	0.0741	0.0741	0.1481	1.63E-18	4.26E-11	2.32E-29	-13.500	-13.500	-6.750	-11.25
PA3499	-	Hypothetical protein	2.0000	10.4444	2.6667	1.63E-03	1.28E-123	1.10E-04	2.000	10.444	2.667	5.04
PA3500	-	Conserved hypothetical protein	1.3333	8.0833	3.0833	3.94E-03	3.83E-135	3.67E-12	1.333	8.083	3.083	4.17
PA3501	-	Hypothetical protein	2.0000	3.8750	4.5000	1.11E-02	9.22E-09	9.97E-12	2.000	3.875	4.500	3.46
PA3502	-	Hypothetical protein	6.0000	20.0000	5.2500	1.69E-16	2.99E-250	2.41E-11	6.000	20.000	5.250	10.42
PA3506	-	Probable decarboxylase	4.2500	13.0000	6.2500	3.44E-19	6.36E-253	1.33E-42	4.250	13.000	6.250	7.83
PA3507	-	Probable short-chain dehydrogenase	54.0000	86.0000	24.0000	0.00E+00	0.00E+00	7.19E-118	54.000	86.000	24.000	54.67
PA3508	-	Probable transcriptional regulator	1.9000	2.1000	4.7000	3.49E-04	5.68E-05	7.95E-24	1.900	2.100	4.700	2.90
PA3509	-	Probable hydrolase	5.0000	4.6667	6.3333	2.88E-15	2.85E-14	2.30E-27	5.000	4.667	6.333	5.33
PA3510	-	Hypothetical protein	25.6667	33.6667	9.5000	0.00E+00	0.00E+00	2.57E-85	25.667	33.667	9.500	22.94
PA3511	-	Probable short-chain dehydrogenase	8.3333	13.0000	5.6667	2.02E-37	3.92E-103	2.03E-13	8.333	13.000	5.667	9.00
PA3512	-	Probable ABC transporter permease	1.7500	2.0000	4.6250	3.42E-03	3.48E-04	1.27E-20	1.750	2.000	4.625	2.79
PA3513	-	Hypothetical protein	7.2500	12.5000	7.7500	2.96E-44	8.82E-160	5.34E-49	7.250	12.500	7.750	9.17
PA3514	-	Probable ATP-binding component of ABC transporter	8.2500	11.7500	7.7500	5.38E-64	1.08E-151	7.55E-54	8.250	11.750	7.750	9.25
PA3515	-	Hypothetical protein	0.0172	0.0690	0.1552	9.78E-177	2.75E-23	3.31E-55	-58.000	-14.500	-6.444	-26.31
PA3516	-	Probable lyase {adenylosuccinate lyase}	0.0714	0.2381	0.1429	1.46E-21	2.42E-02	3.55E-61	-14.000	-4.200	-7.000	-8.40
PA3517	-	Probable lyase {adenylosuccinate lyase}	0.0244	0.1463	0.0732	8.48E-123	4.89E-05	9.05E-221	-41.000	-6.833	-13.667	-20.50
PA3519	-	Hypothetical protein	0.0097	0.0097	0.0194	0.00E+00	0.00E+00	0.00E+00	-103.000	-103.000	-51.500	-85.83
PA3522	*mexQ*	RND efflux transporter MexQ	0.1200	0.0400	0.2000	2.95E-05	1.03E-26	5.93E-41	-8.333	-25.000	-5.000	-12.78
PA3523	*mexP*	RND efflux MFP MexP	0.0192	0.0577	0.0577	4.96E-230	2.74E-49	0.00E+00	-52.000	-17.333	-17.333	-28.89
PA3525	*argG*	Argininosuccinate synthase	7.3273	6.5030	2.5576	0.00E+00	0.00E+00	5.40E-48	7.327	6.503	2.558	5.46
PA3527	*pyrC*	Dihydroorotase	3.6129	2.8226	2.1452	3.99E-67	2.07E-37	1.83E-17	3.613	2.823	2.145	2.86
PA3529	*[tsaA]*	Alkylhydroperoxide reductase C {peroxidase}	0.4199	0.5860	0.1610	2.83E-02	9.06E-03	3.86E-42	-2.381	-1.707	-6.210	-3.43
PA3531	*bfrB*	Bacterioferritin	4.0990	1.5308	6.3596	7.38E-236	3.12E-23	0.00E+00	4.099	1.531	6.360	4.00
PA3532	-	Hypothetical protein	4.5882	3.9412	13.1176	4.30E-50	3.25E-36	0.00E+00	4.588	3.941	13.118	7.22
PA3552	*arnB [pmrH]*	ArnB {UDP-4-amino-4-deoxy-L-arabinose--oxoglutarate aminotransferase}	59.2800	52.4600	6.4200	0.00E+00	0.00E+00	4.98E-238	59.280	52.460	6.420	39.39
PA3553	*arnC [pmrF]*	ArnC, putative glycosyl transferase {undecaprenyl-phosphate 4-deoxy-4-formamido-L-arabinose transferase}	66.5636	52.7091	3.7818	0.00E+00	0.00E+00	3.46E-65	66.564	52.709	3.782	41.02
PA3554	*arnA [prmL]*	ArnA, {bifunctional UDP-glucuronic acid decarboxylase/UDP-4-amino-4-deoxy-L-arabinose formyltransferase}	24.6458	24.0521	4.0417	0.00E+00	0.00E+00	3.30E-139	24.646	24.052	4.042	17.58
PA3555	*arnD [amrJ]*	ArnD {4-deoxy-4-formamido-L-arabinose-phosphoundecaprenol deformylase ArnD}	15.6563	13.5938	5.8125	0.00E+00	0.00E+00	6.88E-112	15.656	13.594	5.813	11.69
PA3556	*arnT [pqaB]*	Inner membrane L-Ara4N transferase ArnT {4-amino-4-deoxy-L-arabinose lipid A transferase}	3.8485	5.4848	1.9091	7.02E-67	4.14E-155	1.30E-10	3.848	5.485	1.909	3.75
PA3559	-	Probable nucleotide sugar dehydrogenase	4.4472	2.8211	2.9024	4.74E-179	4.34E-61	3.04E-63	4.447	2.821	2.902	3.39
PA3560	*fruA*	Phosphotransferase system transporter fructose-specific IIBC component, FruA	6.0000	1.5000	8.8571	1.12E-111	2.86E-05	1.89E-277	6.000	1.500	8.857	5.45
PA3562	*fruI [ptsI]*	Phosphotransferase system transporter enzyme I, FruI {PTS system fructose-specific transporter subunit FruI}	8.1667	2.0000	8.6667	4.68E-163	1.87E-07	1.41E-184	8.167	2.000	8.667	6.28

PA3563	*fruR*	Fructose transport system repressor FruR {FruR family transcriptional regulator}	6.6400	10.2400	2.6400	1.52E-140	0.00E+00	4.02E-14	6.640	10.240	2.640	6.51
PA3565	-	Probable transcriptional regulator	2.4545	2.8182	3.0000	3.29E-08	4.29E-11	2.45E-10	2.455	2.818	3.000	2.76
PA3566	*[ycnE]*	Conserved hypothetical protein	11.0476	15.0000	3.2857	1.36E-181	0.00E+00	1.53E-09	11.048	15.000	3.286	9.78
PA3570	*mmsA*	Methylmalonate-semialdehyde dehydrogenase	0.9217	0.1869	0.3658	2.81E-09	8.20E-12	1.63E-02	-1.085	-5.350	-2.734	-3.06
PA3574	*nalD*	NalD {transcriptional regulator}	0.0279	0.0349	0.1023	2.52E-304	1.22E-199	1.42E-265	-35.833	-28.667	-9.773	-24.76
{PA3574a}	-	Possibly 3574.1 {YP_008719768.1 copper chaperone CopZ}	0.0221	0.0068	0.0311	0.00E+00	0.00E+00	0.00E+00	-45.282	-147.167	-32.109	-74.85
PA3580	*[ybaK]*	Conserved hypothetical protein	6.6000	5.2000	2.2667	4.73E-53	2.85E-30	1.27E-03	6.600	5.200	2.267	4.69
PA3581	*glpF*	Glycerol uptake facilitator protein	51.5000	49.0000	42.0000	0.00E+00	0.00E+00	0.00E+00	51.500	49.000	42.000	47.50
PA3582	*glpK*	Glycerol kinase	6.2400	2.7200	4.0800	5.98E-158	4.52E-23	6.61E-56	6.240	2.720	4.080	4.35
PA3584	*glpD*	Glycerol-3-phosphate dehydrogenase	19.7778	14.1111	31.0000	0.00E+00	0.00E+00	0.00E+00	19.778	14.111	31.000	21.63
PA3587	*metR*	Transcriptional regulator MetR	1.5313	1.8438	4.7813	6.20E-06	3.39E-08	1.75E-68	1.531	1.844	4.781	2.72
PA3599	-	Probable transcriptional regulator	2.0000	1.7500	3.3000	5.44E-07	3.05E-05	1.33E-17	2.000	1.750	3.300	2.35
PA3600	*[rpl36]*	{50S ribosomal protein L36}	8.5000	3.3889	24.4444	3.97E-64	5.19E-08	0.00E+00	8.500	3.389	24.444	12.11
PA3601	*[ykgM]*	{50S ribosomal protein L31}	32.5238	53.6667	27.0000	0.00E+00	0.00E+00	0.00E+00	32.524	53.667	27.000	37.73
PA3614	-	Hypothetical protein	1.4735	2.0390	2.5933	6.37E-22	1.02E-45	6.78E-80	1.474	2.039	2.593	2.04
PA3619	-	Hypothetical protein	8.5000	8.4167	2.1667	1.70E-137	1.02E-134	1.42E-04	8.500	8.417	2.167	6.36
PA3629	*adhC*	Alcohol dehydrogenase class III	0.7114	0.7207	0.2621	1.89E-05	3.39E-04	3.46E-17	-1.406	-1.387	-3.815	-2.20
PA3630	*gfnR*	Glutathione-dependent formaldehyde neutralization regulator GfnR	3.1667	2.2500	2.4167	3.50E-14	8.14E-07	6.10E-06	3.167	2.250	2.417	2.61
PA3639	*accA*	Acetyl-CoA carboxylase carboxyltransferase subunit alpha	2.9921	1.9370	1.2795	2.41E-81	2.93E-29	1.43E-09	2.992	1.937	1.280	2.07
PA3645	*fabZ [sefA]*	(3R)-hydroxymyristoyl-[acyl carrier protein] dehydratase	3.2143	3.9396	1.0769	4.00E-58	5.20E-94	1.86E-02	3.214	3.940	1.077	2.74
PA3654	*pyrH [smbA]*	Uridylate kinase	0.6386	0.2169	0.5382	1.07E-02	1.08E-04	4.77E-02	-1.566	-4.611	-1.858	-2.68
PA3657	*map*	Methionine aminopeptidase	2.8326	3.9657	1.1545	3.00E-61	3.82E-135	3.40E-05	2.833	3.966	1.155	2.65
PA3658	*glnD [nfrX]*	Protein-PII uridylyltransferase {bifunctional uridylyltransferase/uridylyl-removing protein}	3.2750	2.4000	1.4250	1.04E-71	5.20E-34	1.10E-07	3.275	2.400	1.425	2.37
PA3659	*[dapC]*	Probable succinyldiaminopimelate transaminase	2.6000	2.4667	1.2000	2.51E-27	1.84E-23	1.32E-02	2.600	2.467	1.200	2.09
PA3661	-	Hypothetical protein	48.2500	193.7500	18.7500	0.00E+00	0.00E+00	3.07E-191	48.250	193.750	18.750	86.92
PA3664	*[yffB]*	Conserved hypothetical protein	2.8983	2.3390	2.3390	3.29E-18	1.57E-11	5.76E-09	2.898	2.339	2.339	2.53
PA3665	-	Hypothetical protein	1.4000	2.7500	3.2000	3.51E-03	1.47E-11	1.23E-13	1.400	2.750	3.200	2.45
PA3667	-	Probable pyridoxal-phosphate dependent protein {cysteine desulfurase}	0.1020	0.1837	0.5510	5.46E-11	1.28E-03	1.02E-02	-9.800	-5.444	-1.815	-5.69
PA3668	-	Conserved hypothetical protein	0.1951	0.0122	0.4634	5.60E-03	0.00E+00	1.69E-04	-5.125	-82.000	-2.158	-29.76
PA3669	-	Hypothetical protein	0.1549	0.1690	0.1972	4.75E-05	7.68E-05	2.45E-38	-6.455	-5.917	-5.071	-5.81
PA3676	*mexK*	RND efflux transporter MexK	0.2075	0.2264	0.3019	3.72E-02	1.64E-03	6.41E-15	-4.818	-4.417	-3.313	-4.18
PA3691	-	Hypothetical protein	4.7678	5.7116	4.0936	3.71E-226	0.00E+00	3.62E-156	4.768	5.712	4.094	4.86
PA3692	*lptF*	Lipotoxon F, LptF {outer membrane porin F}	5.6342	5.2017	5.3915	0.00E+00	0.00E+00	0.00E+00	5.634	5.202	5.391	5.41
PA3704	*wspE*	Probable chemotaxis sensor/effector fusion protein	0.1446	0.0482	0.3012	1.34E-05	1.13E-65	6.25E-16	-6.917	-20.750	-3.320	-10.33
PA3706	*wspC*	Probable protein methyltransferase {biofilm formation methyltransferase WspC}	0.1132	0.1321	0.5849	2.12E-09	9.35E-08	2.65E-02	-8.833	-7.571	-1.710	-6.04
PA3707	*wspB*	Hypothetical protein	0.1957	0.0435	0.5761	8.48E-03	6.32E-59	2.16E-02	-5.111	-23.000	-1.736	-9.95
PA3710	-	Probable GMC-type oxidoreductase	0.1667	0.0606	0.3333	1.12E-03	2.89E-49	3.45E-13	-6.000	-16.500	-3.000	-8.50
PA3711	-	Probable transcriptional regulator	0.2143	0.1667	0.5357	3.51E-02	9.41E-05	5.34E-03	-4.667	-6.000	-1.867	-4.18
PA3724	*lasB*	Elastase LasB	0.0090	0.0119	0.0021	0.00E+00	0.00E+00	0.00E+00	-111.559	-84.289	-474.125	-223.32
PA3729	-	Conserved hypothetical protein	2.8243	1.2905	3.7095	1.90E-79	1.33E-12	2.60E-150	2.824	1.291	3.709	2.61
PA3730	-	Hypothetical protein	6.8837	2.3953	4.6977	9.68E-168	1.79E-14	1.72E-65	6.884	2.395	4.698	4.66
PA3731	*[yjfJ]*	Conserved hypothetical protein	5.1684	1.3520	5.3827	1.51E-236	6.67E-10	6.27E-259	5.168	1.352	5.383	3.97
PA3732	*[yjfI]*	Uncharacterized protein {hypothetical protein}	5.9415	1.6374	5.6257	2.93E-294	6.31E-14	3.72E-257	5.942	1.637	5.626	4.40
PA3733	-	Hypothetical protein	2.3673	2.1429	1.6939	9.00E-24	1.42E-18	2.12E-08	2.367	2.143	1.694	2.07
PA3749	*[yhjE]*	Probable MFS transporter	2.0000	4.3333	2.6667	2.47E-03	6.41E-15	4.45E-05	2.000	4.333	2.667	3.00
PA3757	*nagR*	Transcriptional regulator of *N*-acetylglucosamine catabolism operon	8.6667	16.3333	3.0000	1.82E-49	8.97E-224	8.35E-05	8.667	16.333	3.000	9.33
PA3761	*nagE*	*N*-acetyl-D-glucosamine phosphotransferase system transporter	0.0476	0.0476	0.3810	1.48E-16	2.41E-23	1.16E-06	-21.000	-21.000	-2.625	-14.88

PA3779	-	Hypothetical protein	7.3333	5.6667	1.8333	3.45E-64	1.20E-35	2.31E-02	7.333	5.667	1.833	4.94
PA3790	*oprC*	Putative copper transport outer membrane porin OprC	2.4211	4.2105	1.1754	1.49E-37	8.76E-138	1.50E-04	2.421	4.211	1.175	2.60
PA3792	*leuA*	2-Isopropylmalate synthase	24.6939	15.9388	1.9388	0.00E+00	0.00E+00	1.54E-31	24.694	15.939	1.939	14.19
PA3796	-	Hypothetical protein	0.7182	0.2000	0.4273	1.98E-02	4.41E-03	5.68E-06	-1.392	-5.000	-2.340	-2.91
PA3809	*fdx2*	Ferredoxin [2Fe-2S]	0.1835	0.1899	0.5696	1.87E-03	3.55E-03	1.60E-02	-5.448	-5.267	-1.756	-4.16
PA3810	*hscA*	Heat shock protein HscA {chaperone protein HscA}	0.1667	0.1429	0.4881	9.79E-04	4.17E-11	4.03E-03	-6.000	-7.000	-2.049	-5.02
PA3819	*[ycfJ]*	Conserved hypothetical protein	2.7057	2.6099	4.5140	1.25E-80	3.12E-74	1.09E-271	2.706	2.610	4.514	3.28
PA3830	-	Probable transcriptional regulator	0.1034	0.1379	0.4138	4.75E-09	5.95E-05	3.91E-05	-9.667	-7.250	-2.417	-6.44
PA3841	*exoS*	T3SS exoenzyme S ADP-ribosyltransferase	17.0568	6.1818	22.8295	0.00E+00	0.00E+00	0.00E+00	17.057	6.182	22.830	15.36
PA3842	*spcS [orf1]*	T3SS specific *Pseudomonas* chaperone for ExoS, SpcS	53.6000	38.4667	16.6667	0.00E+00	0.00E+00	0.00E+00	53.600	38.467	16.667	36.24
PA3850	-	Hypothetical protein	2.6857	1.1429	3.3714	1.87E-32	1.20E-04	5.49E-53	2.686	1.143	3.371	2.40
PA3852	-	Hypothetical protein	3.1758	1.7912	3.9451	1.79E-58	3.55E-15	1.06E-95	3.176	1.791	3.945	2.97
PA3865	-	Probable amino acid binding protein	3.8235	5.3235	5.4118	3.85E-41	4.16E-88	1.99E-90	3.824	5.324	5.412	4.85
PA3870	*moaA1*	Molybdopterin biosynthetic protein A1 {molybdenum cofactor biosynthesis protein A}	6.9000	1.7000	6.2000	2.88E-72	7.27E-04	3.29E-55	6.900	1.700	6.200	4.93
PA3871	*[nifM]*	Probable PpiC-type peptidyl-prolyl cis-trans isomerase	13.0000	3.7778	15.7778	7.81E-269	2.69E-15	0.00E+00	13.000	3.778	15.778	10.85
PA3872	*narI*	Respiratory nitrate reductase gamma chain {subunit gamma}	29.0000	12.2857	20.1429	0.00E+00	1.80E-196	0.00E+00	29.000	12.286	20.143	20.48
PA3873	*narJ*	Respiratory nitrate reductase delta chain {subunit delta}	32.5000	4.3333	28.3333	0.00E+00	2.26E-15	0.00E+00	32.500	4.333	28.333	21.72
PA3874	*narH*	Respiratory nitrate reductase beta chain {subunit beta}	22.3333	4.8333	26.5000	0.00E+00	1.40E-55	0.00E+00	22.333	4.833	26.500	17.89
PA3875	*narG*	Respiratory nitrate reductase alpha chain {subunit alpha}	25.5000	8.6250	26.3750	0.00E+00	6.47E-271	0.00E+00	25.500	8.625	26.375	20.17
PA3876	*narK2*	Nitrite extrusion protein 2	4.8000	1.4000	13.3000	2.17E-40	3.15E-03	0.00E+00	4.800	1.400	13.300	6.50
PA3881	-	Hypothetical protein	3.8438	1.2500	3.3750	4.62E-28	6.01E-03	7.03E-19	3.844	1.250	3.375	2.82
PA3888	*opuCD*	OpuC ABC transporter, permease protein, OpuCD	3.6667	4.6667	1.8889	4.65E-13	9.31E-23	2.08E-02	3.667	4.667	1.889	3.41
PA3894	*[opmI]*	Probable outer membrane protein precursor	0.0769	0.0769	0.2308	2.63E-17	8.07E-10	1.95E-11	-13.000	-13.000	-4.333	-10.11
PA3895	-	Probable transcriptional regulator	3.6000	4.0400	4.0400	5.46E-33	1.68E-42	1.59E-40	3.600	4.040	4.040	3.89
PA3896	-	Probable 2-hydroxyacid dehydrogenase	2.3000	2.0000	2.3667	1.45E-13	6.32E-10	6.92E-12	2.300	2.000	2.367	2.22
PA3899	*fecI*	Probable RNA polymerase sigma factor	3.5000	11.1250	2.1250	4.54E-10	1.89E-139	8.01E-03	3.500	11.125	2.125	5.58
PA3920	*copA1 [yvgX, cueA]*	Probable metal transporting P-type ATPase CopA1	0.0588	0.0406	0.0304	2.56E-61	0.00E+00	0.00E+00	-17.000	-24.650	-32.867	-24.84
PA3921	-	Probable transcriptional regulator	0.1250	0.2045	0.3068	7.09E-09	1.31E-05	1.23E-14	-8.000	-4.889	-3.259	-5.38
PA3922	-	Conserved hypothetical protein	4.4317	7.0383	8.5683	1.14E-212	0.00E+00	0.00E+00	4.432	7.038	8.568	6.68
PA3923	-	Hypothetical protein	4.8308	20.7846	7.5923	5.67E-260	0.00E+00	0.00E+00	4.831	20.785	7.592	11.07
PA3924	-	Probable medium-chain acyl-CoA ligase {long-chain-fatty-acid-CoA ligase}	2.7917	5.8542	3.7917	5.79E-42	1.81E-241	2.36E-84	2.792	5.854	3.792	4.15
PA3934	-	Conserved hypothetical protein	0.1648	0.0989	0.3626	3.55E-04	2.71E-24	3.95E-09	-6.067	-10.111	-2.758	-6.31
PA3935	*tauD*	Taurine dioxygenase	11.8750	2.3750	3.3750	1.45E-215	3.71E-06	2.08E-10	11.875	2.375	3.375	5.88
PA3942	*tesB*	Acyl-CoA thioesterase II	3.8286	6.2000	3.9714	3.22E-44	1.09E-136	2.78E-45	3.829	6.200	3.971	4.67
PA3959	-	Hypothetical protein	2.4000	2.2000	2.4000	1.45E-04	5.88E-04	1.42E-03	2.400	2.200	2.400	2.33
PA3970	*amn*	AMP nucleosidase	2.4247	2.3288	2.0137	3.62E-36	3.29E-32	6.90E-21	2.425	2.329	2.014	2.26
PA3975	*thiD*	Phosphomethylpyrimidine kinase	2.2549	2.3529	2.2745	4.36E-16	2.16E-17	1.19E-13	2.255	2.353	2.275	2.29
PA3976	*thiE [thiC]*	Thiamine-phosphate pyrophosphorylase	4.5667	4.4333	2.7000	7.61E-47	2.71E-44	4.40E-12	4.567	4.433	2.700	3.90
PA3977	*hemL*	Glutamate-1-semialdehyde 2,1-aminomutase {glutamate-1-semialdehyde aminotransferase}	2.4941	2.9706	1.0118	3.73E-49	9.72E-74	9.20E-04	2.494	2.971	1.012	2.16
PA3986	-	Hypothetical protein	0.1075	0.0430	0.3226	7.04E-10	6.42E-64	2.38E-11	-9.300	-23.250	-3.100	-11.88
PA4010	-	Hypothetical protein {3-methyladenine DNA glycosylase}	4.2368	2.9474	1.9868	4.02E-86	2.58E-36	3.09E-12	4.237	2.947	1.987	3.06
PA4011	-	Hypothetical protein	1.9091	2.9697	1.1717	1.92E-22	3.55E-62	1.11E-04	1.909	2.970	1.172	2.02
PA4023	*eat [eutP]*	Ethanolamine transporter, Eat	2.6923	2.2308	5.7692	4.85E-15	6.86E-10	1.24E-81	2.692	2.231	5.769	3.56
PA4026	-	Probable acetyltransferase	2.5769	2.4103	2.8269	8.73E-33	1.37E-27	7.46E-38	2.577	2.410	2.827	2.60
PA4034	*aqpZ [bniP]*	Aquaporin Z	2.5000	1.7222	2.0556	1.47E-09	1.08E-04	2.38E-04	2.500	1.722	2.056	2.09
PA4036	-	Probable two-component sensor	0.1053	0.1579	0.4737	1.26E-05	2.32E-04	1.38E-04	-9.500	-6.333	-2.111	-5.98
PA4044	*dxs*	1-Deoxy-D-xylulose-5-phosphate synthase	1.9661	3.5000	1.0593	5.75E-29	5.80E-111	1.29E-04	1.966	3.500	1.059	2.18
PA4055	*ribC [ribB]*	Riboflavin synthase alpha chain {subunit alpha}	6.0000	3.8197	1.2787	6.53E-257	1.26E-87	1.76E-04	6.000	3.820	1.279	3.70
PA4057	*nrdR [ybaD]*	Transcriptional regulator NrdR	2.2857	3.5556	1.8095	1.32E-13	4.25E-37	9.11E-06	2.286	3.556	1.810	2.55

PA4063	-	Hypothetical protein	144.7000	64.2000	26.7000	0.00E+00	0.00E+00	0.00E+00	144.700	64.200	26.700	78.53
PA4064	-	Probable ATP-binding component of ABC transporter	17.2857	8.0000	7.7143	0.00E+00	3.93E-70	1.09E-61	17.286	8.000	7.714	11.00
PA4065	-	Hypothetical protein	6.8667	4.0667	3.5333	3.99E-128	6.15E-38	1.02E-24	6.867	4.067	3.533	4.82
PA4074	-	Probable transcriptional regulator	2.5000	2.3750	3.2500	3.11E-08	2.99E-07	4.78E-12	2.500	2.375	3.250	2.71
PA4076	-	Hypothetical protein	2.0741	2.2222	1.7037	1.16E-05	1.06E-06	2.54E-02	2.074	2.222	1.704	2.00
PA4078	-	Probable nonribosomal peptide synthetase	0.1325	0.1446	0.0723	2.06E-07	5.87E-13	0.00E+00	-7.545	-6.917	-13.833	-9.43
{PA4108a}	-	Intergenic region {YP_008719775.1 hypothetical protein PA4108a}	7.6286	19.9429	2.2286	5.10E-86	0.00E+00	5.69E-04	7.629	19.943	2.229	9.93
PA4112	-	Probable sensor/response regulator hybrid protein	0.0526	0.1447	0.3158	3.11E-58	3.01E-14	8.79E-12	-19.000	-6.909	-3.167	-9.69
PA4114	*[bltD]*	Lysine decarboxylase {spermidine acetyltransferase}	4.6711	3.2105	2.0789	6.63E-82	6.56E-34	3.51E-10	4.671	3.211	2.079	3.32
PA4115	-	Conserved hypothetical protein	2.6471	4.8471	1.5765	6.05E-46	3.86E-192	2.83E-11	2.647	4.847	1.576	3.02
PA4127	*hpcG [hpaH]*	2-Oxo-hepta-3-ene-1,7-dioate hydratase	0.0339	0.1356	0.0847	2.19E-84	1.39E-05	1.39E-133	-29.500	-7.375	-11.800	-16.23
PA4129	-	Hypothetical protein	0.0065	0.0858	0.0259	0.00E+00	2.43E-45	0.00E+00	-154.500	-11.660	-38.625	-68.26
PA4130	-	Probable sulfite or nitrite reductase {sulfite/nitrite reductase}	0.0107	0.0139	0.0192	0.00E+00	0.00E+00	0.00E+00	-93.800	-72.154	-52.111	-72.69
PA4132	-	Conserved hypothetical protein	0.0087	0.0194	0.0301	0.00E+00	0.00E+00	0.00E+00	-114.444	-51.500	-33.226	-66.39
PA4133	*[cytN, ccoN, fixN]*	Cytochrome c oxidase subunit I (cbb3-type)	0.0079	0.0105	0.0092	0.00E+00	0.00E+00	0.00E+00	-127.083	-95.313	-108.929	-110.44
PA4141	-	Hypothetical protein	0.0181	0.1253	0.0559	0.00E+00	5.10E-33	0.00E+00	-55.174	-7.981	-17.873	-27.01
PA4142	-	Probable secretion protein	0.1290	0.0645	0.0968	6.18E-05	6.20E-17	1.01E-120	-7.750	-15.500	-10.333	-11.19
PA4143	*[cvaB, cyaB]*	Probable toxin transporter	0.1429	0.2143	0.2143	1.01E-04	2.44E-02	2.76E-15	-7.000	-4.667	-4.667	-5.44
PA4155	-	Hypothetical protein	15.7500	4.5000	8.2500	7.26E-299	3.10E-17	2.60E-65	15.750	4.500	8.250	9.50
PA4156	*fvbA*	FvbA {TonB-dependent receptor}	21.2500	45.2500	13.7500	0.00E+00	0.00E+00	1.55E-305	21.250	45.250	13.750	26.75
PA4158	*fepC*	Ferric enterobactin transporter FepC	1.8000	4.4000	3.4000	4.40E-03	7.36E-15	3.35E-07	1.800	4.400	3.400	3.20
PA4159	*fepB*	Ferrienterobactin-binding periplasmic protein precursor FepB	3.0000	9.0000	10.0000	2.75E-04	4.45E-48	1.04E-60	3.000	9.000	10.000	7.33
PA4170	-	Hypothetical protein	4.0769	1.9231	3.5385	1.65E-26	1.56E-05	2.95E-17	4.077	1.923	3.538	3.18
PA4180	-	Probable acetolactate synthase large subunit	4.8983	3.4746	1.7627	7.88E-173	8.91E-77	3.41E-13	4.898	3.475	1.763	3.38
PA4204	*ppgL*	Periplasmic gluconolactonase, PpgL	4.9111	3.4000	1.1778	1.35E-116	1.21E-48	2.26E-02	4.911	3.400	1.178	3.16
PA4205	*mexG*	Hypothetical protein	0.0495	0.0310	0.0557	5.48E-73	1.11E-174	0.00E+00	-20.188	-32.300	-17.944	-23.48
PA4206	*mexH*	RND efflux MFP precursor	0.0432	0.0270	0.0270	5.78E-110	4.20E-247	0.00E+00	-23.125	-37.000	-37.000	-32.38
PA4207	*mexI*	RND efflux transporter	0.0315	0.0551	0.0551	1.01E-205	2.71E-121	0.00E+00	-31.750	-18.143	-18.143	-22.68
PA4209	*phzM*	Probable phenazine-specific methyltransferase	0.0023	0.0149	0.0126	0.00E+00	0.00E+00	0.00E+00	-435.500	-67.000	-79.182	-193.89
PA4211	*phzB1*	Probable phenazine biosynthesis protein PhzB1	0.0003	0.0030	0.0024	0.00E+00	0.00E+00	0.00E+00	-2957.000	-328.556	-422.429	-1235.99
PA4212	*phzC1*	Phenazine biosynthesis protein PhzC1	0.0109	0.0655	0.0135	0.00E+00	3.34E-193	0.00E+00	-91.647	-15.275	-74.190	-60.37
PA4213	*phzD1*	Phenazine biosynthesis protein PhzD1	0.0017	0.0398	0.0040	0.00E+00	0.00E+00	0.00E+00	-586.333	-25.129	-251.286	-287.58
PA4214	*phzE1*	Phenazine biosynthesis protein PhzE1	0.0029	0.0130	0.0058	0.00E+00	0.00E+00	0.00E+00	-347.000	-77.111	-173.500	-199.20
PA4215	*phzF1*	Probable phenazine biosynthesis protein PhzF1 {trans-2,3-dihydro-3-hydroxyanthranilate isomerase}	0.0062	0.0155	0.0093	0.00E+00	0.00E+00	0.00E+00	-161.667	-64.667	-107.778	-111.37
PA4216	*phzG1*	Probable pyridoxamine 5′-phosphate oxidase	0.0088	0.0113	0.0151	0.00E+00	0.00E+00	0.00E+00	-113.714	-88.444	-66.333	-89.50
PA4217	*phzS*	Flavin-containing monooxygenase	0.0033	0.0092	0.0109	0.00E+00	0.00E+00	0.00E+00	-306.833	-108.294	-92.050	-169.06
PA4221	*fptA*	Fe(III)-pyochelin outer membrane receptor precursor	2.7965	2.1858	1.8761	2.13E-94	5.80E-53	9.48E-36	2.796	2.186	1.876	2.29
PA4242	*rpmJ*	50S ribosomal protein L36	0.2061	0.1190	0.4235	3.10E-02	1.84E-17	1.91E-05	-4.852	-8.402	-2.361	-5.20
PA4245	*rpmD*	50S ribosomal protein L30	0.4542	0.3092	0.4487	4.52E-02	2.18E-02	2.41E-02	-2.202	-3.235	-2.229	-2.56
PA4280.4	-	tRNA-Ile	0.0332	0.0062	0.1867	1.03E-148	0.00E+00	5.52E-33	-30.125	-160.667	-5.356	-65.38
PA4283	*recD*	Exodeoxyribonuclease V alpha chain {subunit alpha}	0.1111	0.1270	0.4603	1.42E-10	9.49E-13	2.14E-04	-9.000	-7.875	-2.172	-6.35
PA4290	-	Probable chemotaxis transducer	2.6364	2.8182	10.7273	2.11E-21	4.61E-24	0.00E+00	2.636	2.818	10.727	5.39
PA4293	*pprA*	Two-component sensor PprA	0.1000	0.0250	0.2000	1.96E-15	5.08E-89	3.53E-45	-10.000	-40.000	-5.000	-18.33
PA4297	*tadG*	TadG {hypothetical protein}	0.1077	0.0923	0.2769	6.52E-13	4.64E-18	1.09E-21	-9.286	-10.833	-3.611	-7.91
PA4299	*tadD*	TadD {T2SS protein TadD}	0.0308	0.0154	0.1231	1.73E-71	7.86E-224	3.55E-86	-32.500	-65.000	-8.125	-35.21
PA4302	*tadA [hvbA]*	ATPase TadA	0.0093	0.0278	0.0463	0.00E+00	1.03E-187	0.00E+00	-108.000	-36.000	-21.600	-55.20
PA4303	*tadZ*	TadZ {hypothetical protein}	0.0105	0.0526	0.0737	0.00E+00	7.49E-49	8.07E-300	-95.000	-19.000	-13.571	-42.52
PA4304	*rcpA [xqhC]*	RcpA { type II/III secretion system protein}	0.0248	0.0744	0.0579	1.33E-191	4.88E-35	0.00E+00	-40.333	-13.444	-17.286	-23.69
PA4306	*flp*	Type IVb pilin, Flp	0.0058	0.0064	0.0138	0.00E+00	0.00E+00	0.00E+00	-171.273	-157.000	-72.462	-133.58
PA4309	*pctA*	Chemotactic transducer PctA	2.3571	4.3571	2.9167	2.94E-39	8.67E-166	2.28E-62	2.357	4.357	2.917	3.21
PA4310	*pctB*	Chemotactic transducer PctB	1.8387	4.0726	1.4355	6.95E-26	2.42E-164	8.09E-13	1.839	4.073	1.435	2.45

PA4315	*mvaT*	Transcriptional regulator MvaT, P16 subunit	5.6872	5.7364	2.1856	0.00E+00	0.00E+00	2.04E-56	5.687	5.736	2.186	4.54
PA4316	*sbcB*	Exodeoxyribonuclease I {exonuclease I}	4.5833	2.5500	1.7333	1.64E-137	3.21E-34	1.46E-11	4.583	2.550	1.733	2.96
PA4317	-	Hypothetical protein	40.8000	19.9000	12.4500	0.00E+00	0.00E+00	0.00E+00	40.800	19.900	12.450	24.38
PA4318	-	Hypothetical protein	2.0769	1.1154	4.5385	4.07E-09	9.43E-03	1.01E-48	2.077	1.115	4.538	2.58
PA4324	-	Hypothetical protein	0.0469	0.1080	0.5352	9.33E-69	8.45E-12	4.40E-03	-21.300	-9.261	-1.868	-10.81
PA4327	-	Hypothetical protein	4.7000	5.5500	1.9500	1.21E-44	8.79E-67	2.40E-04	4.700	5.550	1.950	4.07
PA4333	*[fumA]*	Probable fumarase	0.6791	0.2513	0.4545	7.61E-04	7.37E-04	2.85E-03	-1.472	-3.979	-2.200	-2.55
PA4353	*[yajB]*	Conserved hypothetical protein	1.6154	1.4231	3.0769	1.43E-04	1.14E-03	1.81E-14	1.615	1.423	3.077	2.04
PA4361	-	Probable oxidoreductase	2.1200	2.4400	3.5200	6.96E-10	2.77E-13	2.46E-28	2.120	2.440	3.520	2.69
PA4366	*sodB*	Superoxide dismutase	0.5558	0.6500	0.3140	9.03E-04	2.01E-03	7.04E-05	-1.799	-1.538	-3.185	-2.17
PA4376	*pncB2*	Nicotinate phosphoribosyltransferase	2.1176	1.3235	3.2647	5.27E-14	4.12E-05	1.54E-34	2.118	1.324	3.265	2.24
PA4385	*groEL [mopA]*	60-kDa chaperonin GroEL {molecular chaperone GroEL}	0.1160	0.0543	0.0373	2.24E-05	2.27E-242	0.00E+00	-8.623	-18.402	-26.837	-17.95
PA4386	*groES [mopB]*	10-kDa chaperonin GroES {co-chaperonin GroES}	0.1197	0.0742	0.0393	1.60E-07	4.15E-139	0.00E+00	-8.352	-13.473	-25.457	-15.76
PA4387	*[fxsA]*	Conserved hypothetical protein {phage exclusion suppressor FxsA}	0.1722	0.0728	0.2980	4.60E-04	2.73E-26	1.77E-16	-5.808	-13.727	-3.356	-7.63
PA4396	-	Two-component response regulator	2.8182	1.7273	2.0909	4.83E-11	2.15E-04	6.83E-04	2.818	1.727	2.091	2.21
PA4404	-	Hypothetical protein	0.1475	0.0820	0.3443	3.87E-05	8.18E-17	2.91E-11	-6.778	-12.200	-2.905	-7.29
PA4408	*ftsA*	Cell division protein FtsA	0.1838	0.2201	0.3953	1.80E-02	3.81E-07	1.60E-03	-5.442	-4.544	-2.530	-4.17
PA4413	*ftsW*	Cell division protein FtsW	0.1206	0.0993	0.4610	2.06E-10	2.06E-23	5.66E-04	-8.294	-10.071	-2.169	-6.84
PA4420	*[yabC, ylxA, mraW]*	Conserved hypothetical protein {*S*-adenosyl-methyltransferase MraW}	0.1613	0.1649	0.8996	4.16E-04	3.15E-10	5.20E-03	-6.200	-6.065	-1.112	-4.46
PA4423	*[yraM]*	Hypothetical protein	8.7742	8.0161	1.4839	0.00E+00	0.00E+00	3.85E-09	8.774	8.016	1.484	6.09
PA4425	*[yraO]*	Sedoheptulose 7-phosphate isomerase GmhA {phosphoheptose isomerase}	2.7642	3.0813	1.4878	1.21E-38	2.40E-49	1.36E-06	2.764	3.081	1.488	2.44
PA4432	*rpsI*	30S ribosomal protein S9	0.1692	0.0403	0.3761	2.78E-03	6.05E-275	1.97E-04	-5.912	-24.790	-2.659	-11.12
PA4451	*[yrbA]*	Conserved hypothetical protein	4.1524	3.1238	1.7810	4.46E-48	1.10E-24	8.23E-05	4.152	3.124	1.781	3.02
PA4460	*lptH [yhbN]*	LptH {hypothetical protein}	1.7339	3.8306	1.0484	2.70E-20	7.92E-128	7.43E-04	1.734	3.831	1.048	2.20
PA4465	*[ybhJ]*	Conserved hypothetical protein	0.1972	0.0865	0.9446	2.08E-02	9.99E-40	2.33E-03	-5.070	-11.560	-1.059	-5.90
PA4467	-	Hypothetical protein	1.3750	2.2500	15.0000	1.38E-02	1.58E-05	0.00E+00	1.375	2.250	15.000	6.21
PA4468	*sodM [sodA]*	Superoxide dismutase	4.0909	1.7727	21.5000	8.58E-29	4.47E-05	0.00E+00	4.091	1.773	21.500	9.12
PA4469	-	Hypothetical protein	15.8000	16.0000	44.4000	0.00E+00	0.00E+00	0.00E+00	15.800	16.000	44.400	25.40
PA4470	*fumC1*	Fumarate hydratase	3.7308	2.8077	24.3846	2.52E-45	1.92E-23	0.00E+00	3.731	2.808	24.385	10.31
PA4471	*[fagA]*	Hypothetical protein	33.5000	13.7500	27.5000	0.00E+00	4.64E-102	0.00E+00	33.500	13.750	27.500	24.92
PA4492	*magA*	MagA {hypothetical protein}	3.0167	2.5167	2.6667	4.03E-36	2.76E-23	3.35E-24	3.017	2.517	2.667	2.73
PA4501	*opdD [opdP]*	Glycine-glutamate dipeptide porin OpdP	1.0588	1.4706	4.1176	5.43E-03	1.53E-04	2.51E-40	1.059	1.471	4.118	2.22
PA4502	*dppA4*	Probable binding protein component of ABC transporter	1.8085	4.2553	1.7660	4.17E-15	8.49E-107	3.33E-11	1.809	4.255	1.766	2.61
PA4505	*dppD*	Dipeptide ABC transporter ATP-binding protein DppD	1.7231	7.0154	1.2769	5.07E-12	9076903527E	1.44E-03	1.723	7.015	1.277	3.34
PA4508	-	Probable transcriptional regulator	0.1053	0.1053	0.2105	1.66E-09	5.17E-07	3.75E-12	-9.500	-9.500	-4.750	-7.92
PA4509	-	Hypothetical protein	0.1154	0.0769	0.5385	9.75E-06	5.70E-16	1.14E-02	-8.667	-13.000	-1.857	-7.84
PA4524	*nadC*	Nicotinate-nucleotide pyrophosphorylase	1.9500	3.3250	1.5500	1.94E-10	2.53E-33	6.54E-04	1.950	3.325	1.550	2.28
PA4526	*pilB*	Type 4 fimbrial biogenesis protein PilB	2.5000	2.9318	3.9318	3.86E-50	9.09E-73	1.58E-144	2.500	2.932	3.932	3.12
PA4534	-	Hypothetical protein	0.7500	0.1667	0.5104	4.44E-02	5.40E-04	1.00E-03	-1.333	-6.000	-1.959	-3.10
PA4537	-	Hypothetical protein	2.1522	1.2174	3.0000	3.41E-05	3.82E-02	2.80E-08	2.152	1.217	3.000	2.12
PA4570	-	Hypothetical protein	4.3478	2.0000	7.2609	8.29E-19	6.97E-04	3.09E-59	4.348	2.000	7.261	4.54
PA4597	*oprJ*	Multidrug efflux OMP OprJ precursor	0.2105	0.0526	0.3684	3.65E-02	2.74E-48	2.42E-06	-4.750	-19.000	-2.714	-8.82
PA4605	*[ybdD]*	Conserved hypothetical protein	2.1600	1.9150	2.0000	1.03E-14	5.99E-11	1.03E-09	2.160	1.915	2.000	2.03
PA4607	-	Hypothetical protein	0.0799	0.0606	0.1740	4.58E-26	2.41E-258	3.33E-45	-12.522	-16.503	-5.748	-11.59
PA4615	*fprB*	Probable oxidoreductase FprB	3.5319	3.1702	5.4255	7.23E-41	3.02E-31	3.47E-109	3.532	3.170	5.426	4.04
PA4619	-	Probable c-type cytochrome {cytochrome c}	3.3704	3.2593	5.2222	3.65E-34	3.42E-31	1.93E-91	3.370	3.259	5.222	3.95
PA4620	-	Hypothetical protein	8.8947	8.1053	9.4211	1.92E-150	8.71E-121	3.46E-169	8.895	8.105	9.421	8.81
PA4621	-	Probable oxidoreductase	2.6842	1.9474	3.9474	3.32E-28	5.69E-13	7.89E-65	2.684	1.947	3.947	2.86
PA4625	*cdrA*	Cyclic diguanylate-regulated TPS partner A, CdrA	1.4773	2.4773	2.2955	8.80E-18	9.82E-58	1.49E-46	1.477	2.477	2.295	2.08
PA4633	-	Probable chemotaxis transducer	1.7190	1.9504	3.4050	6.85E-24	1.48E-31	2.44E-115	1.719	1.950	3.405	2.36

PA4643	-	Hypothetical protein	4.6250	4.6250	2.8438	3.16E-43	2.60E-43	5.63E-12	4.625	4.625	2.844	4.03
PA4651	*cupE4*	Pilin assembly chaperone CupE4	0.0725	0.1159	0.0870	1.37E-18	1.12E-08	2.59E-135	-13.800	-8.625	-11.500	-11.31
PA4658	-	Hypothetical protein	4.8500	8.5000	3.0500	4.33E-54	1.37E-205	8.65E-17	4.850	8.500	3.050	5.47
PA4661	*pagL*	Lipid A 3-O-deacylase	0.1163	0.0206	0.3515	4.93E-09	0.00E+00	2.11E-03	-8.598	-48.542	-2.845	-19.99
PA4662	*murI*	Glutamate racemase	0.0893	0.1250	0.5714	5.22E-11	4.30E-06	1.75E-02	-11.200	-8.000	-1.750	-6.98
PA4663	*moeB [chlN]*	Molybdopterin biosynthesis protein MoeB	0.1649	0.0722	0.5567	4.81E-04	3.59E-24	1.12E-02	-6.063	-13.857	-1.796	-7.24
PA4667	-	Hypothetical protein	2.5918	3.8673	2.7551	8.32E-50	5.54E-128	7.11E-56	2.592	3.867	2.755	3.07
PA4668	*lolB*	Lipoprotein localization protein LolB {molecular chaperone LolB}	1.5833	1.8833	2.9333	4.33E-07	7.55E-10	3.75E-24	1.583	1.883	2.933	2.13
PA4676	*[yadF]*	Probable carbonic anhydrase	0.8000	0.2000	0.2714	2.58E-03	6.11E-04	9.16E-22	-1.250	-5.000	-3.684	-3.31
PA4683	-	Hypothetical protein	2.3929	4.9643	3.3571	3.55E-10	1.99E-53	2.86E-19	2.393	4.964	3.357	3.57
PA4687	*hitA*	Ferric iron-binding periplasmic protein HitA	2.3846	1.8231	2.0538	8.77E-38	8.04E-20	1.84E-24	2.385	1.823	2.054	2.09
PA4690.4	-	tRNA-Ile	0.0332	0.0062	0.1867	1.03E-148	0.00E+00	5.52E-33	-30.125	-160.667	-5.356	-65.38
PA4692	-	Conserved hypothetical protein {sulfite oxidase subunit YedY}	2.3636	1.8182	5.4545	1.44E-12	5.62E-07	4.93E-82	2.364	1.818	5.455	3.21
PA4697	-	Hypothetical protein	4.6353	4.4000	2.0941	4.42E-81	7.64E-72	1.44E-10	4.635	4.400	2.094	3.71
PA4699	-	Hypothetical protein	2.9297	4.8438	1.2734	4.06E-53	7.39E-174	4.07E-05	2.930	4.844	1.273	3.02
PA4704	*cbpA*	cAMP-binding protein A	3.5200	1.3600	2.6000	4.13E-28	8.18E-04	5.68E-12	3.520	1.360	2.600	2.49
PA4709	*phuS*	PhuS {hemin degrading factor}	5.0000	5.4286	6.5714	6.81E-30	3.27E-36	5.12E-54	5.000	5.429	6.571	5.67
PA4724.1	-	Hypothetical protein	13.8667	13.8667	2.0000	4.76E-197	1.64E-196	2.73E-02	13.867	13.867	2.000	9.91
PA4726	*cbrB*	Two-component response regulator CbrB	5.9327	5.7404	2.6827	0.00E+00	1.9E-322	9.81E-51	5.933	5.740	2.683	4.79
PA4734	-	Hypothetical protein	1.6829	2.7805	1.8049	5.11E-08	2.76E-23	1.25E-06	1.683	2.780	1.805	2.09
PA4738	*[yjbJ]*	Conserved hypothetical protein	0.1439	0.0281	0.2899	8.74E-06	0.00E+00	4.97E-17	-6.948	-35.585	-3.449	-15.33
PA4741	*rpsO*	30S ribosomal protein S15	0.1450	0.0755	0.3003	1.66E-05	1.23E-78	7.54E-13	-6.898	-13.246	-3.330	-7.82
PA4756	*carB*	Carbamoylphosphate synthetase large subunit	3.0243	2.1068	2.1311	3.72E-128	9.38E-55	9.09E-56	3.024	2.107	2.131	2.42
PA4757	*[yeaS]*	Conserved hypothetical protein {leucine export protein LeuE}	2.6556	1.8222	2.9556	3.76E-30	1.71E-12	3.56E-36	2.656	1.822	2.956	2.48
PA4758	*carA*	Carbamoyl-phosphate synthase small subunit	5.6131	4.7560	4.0119	87339792E-[illegible]	6.16E-212	1.84E-139	5.613	4.756	4.012	4.79
PA4760	*dnaJ*	Heat shock protein DnaJ {molecular chaperone DnaJ}	0.1831	0.0387	0.1725	9.82E-03	2.59E-190	3.25E-74	-5.462	-25.818	-5.796	-12.36
PA4763	*recN*	DNA repair protein RecN [recombination protein N]	1.5507	2.0580	2.4928	8.04E-14	4.52E-25	1.16E-37	1.551	2.058	2.493	2.03
PA4765	*omlA [oprX]*	Outer membrane lipoprotein OmlA precursor	3.2541	2.5492	1.8197	1.27E-51	6.47E-29	6.95E-11	3.254	2.549	1.820	2.54
PA4770	*lldP [lctP]*	L-lactate permease	121.0000	34.1818	20.2727	0.00E+00	0.00E+00	0.00E+00	121.000	34.182	20.273	58.48
PA4771	*lldD [lctD]*	L-lactate dehydrogenase	37.0000	11.7500	17.2500	0.00E+00	0.00E+00	0.00E+00	37.000	11.750	17.250	22.00
PA4772	-	Probable ferredoxin	24.0714	4.0714	10.2857	0.00E+00	1.00E-58	0.00E+00	24.071	4.071	10.286	12.81
PA4773	*speD2*	Hypothetical protein SpeD2	5.9245	3.0566	1.8679	4.85E-111	1.16E-23	1.43E-05	5.925	3.057	1.868	3.62
PA4781	-	Cyclic di-GMP phosphodiesterase	0.0938	0.1406	0.3359	8.21E-16	5.15E-11	2.06E-12	-10.667	-7.111	-2.977	-6.92
PA4782	-	Hypothetical protein	3.7419	2.2903	2.1935	8.18E-16	9.98E-06	1.30E-03	3.742	2.290	2.194	2.74
PA4788	-	Hypothetical protein	6.0870	16.2174	1.9130	9.89E-96	0.00E+00	1.05E-04	6.087	16.217	1.913	8.07
PA4791	-	Hypothetical protein	2.3478	1.2609	2.6087	7.29E-09	8.28E-03	6.33E-09	2.348	1.261	2.609	2.07
PA4793	-	Hypothetical protein	2.3333	4.8333	2.2083	4.96E-25	3.64E-137	2.76E-19	2.333	4.833	2.208	3.13
PA4809	*fdhE*	FdhE {formate dehydrogenase subunit epsilon}	2.7500	4.1250	3.2500	5.00E-13	3.09E-32	5.47E-16	2.750	4.125	3.250	3.38
PA4811	*fdnH [fdhH]*	Nitrate-inducible formate dehydrogenase, beta subunit	4.4766	4.8281	1.9141	7.22E-157	1.84E-188	6.04E-19	4.477	4.828	1.914	3.74
PA4813	*lipC*	Lipase LipC	3.0000	2.4000	3.2000	1.10E-07	1.23E-04	3.51E-07	3.000	2.400	3.200	2.87
PA4833	-	Conserved hypothetical protein	1.9672	1.5902	2.6066	3.86E-11	8.14E-07	3.16E-18	1.967	1.590	2.607	2.05
PA4835	*cntM [zrmC]*	Hypothetical protein CntM	4.6667	1.6667	3.6667	2.53E-15	9.14E-03	2.28E-08	4.667	1.667	3.667	3.33
PA4836	*cntI [zrmB]*	Nicotianamine synthase-like enzyme ZrmB	10.2500	2.0000	7.7500	1.82E-85	4.97E-03	1.45E-43	10.250	2.000	7.750	6.67
PA4837	*cntO [zmrA]*	TonB-dependent Outer Membrane Protein ZrmA	47.0000	26.8000	10.0000	0.00E+00	0.00E+00	4.56E-175	47.000	26.800	10.000	27.93
PA4838	-	Hypothetical protein	2.0000	3.4000	2.4000	7.16E-04	3.47E-09	9.69E-04	2.000	3.400	2.400	2.60
PA4848	*accC*	Biotin carboxylase [acetyl-CoA carboxylase] {acetyl-CoA carboxylase biotin carboxylase subunit}	0.7633	0.2696	0.6991	2.35E-06	1.13E-03	2.25E-02	-1.310	-3.709	-1.430	-2.15
PA4851	-	Hypothetical protein	2.5417	2.9167	1.5000	1.96E-17	5.34E-23	4.39E-03	2.542	2.917	1.500	2.32
PA4852	*[ydhG]*	Conserved hypothetical protein	0.1698	0.1981	0.3774	4.03E-04	1.87E-04	1.38E-09	-5.889	-5.048	-2.650	-4.53
PA4855	*purD*	Phosphoribosylamine-glycine ligase	0.1806	0.2639	0.3056	1.21E-03	3.46E-02	2.50E-15	-5.538	-3.789	-3.273	-4.20
PA4858	-	Conserved hypothetical protein [urea ABC transporter substrate binding protein UrtA-like]	3.3333	6.6667	3.3333	1.87E-12	5.50E-58	9.17E-11	3.333	6.667	3.333	4.44
PA4859	-	Probable ABC transporter permease [urea permease UrtB]	2.0000	1.5000	5.0000	9.23E-04	1.29E-02	3.73E-17	2.000	1.500	5.000	2.83

PA4860	-	Probable ABC transporter permease [urea permease UrtC]	3.0000	10.0000	12.0000	9.09E-03	4.61E-20	5.17E-29	3.000	10.000	12.000	8.33
PA4861	-	Probable ABC transporter ATP-binding protein [urea, UrtD]	4.6667	2.0000	11.0000	2.61E-10	1.49E-02	4.75E-74	4.667	2.000	11.000	5.89
PA4862	-	Probable ABC transporter ATP-binding protein [urea, UrtE]	4.0000	15.5000	11.7500	4.16E-11	1.59E-225	1.39E-120	4.000	15.500	11.750	10.42
PA4868	*ureC*	Urease alpha subunit	3.6866	15.2090	1.5224	7.26E-98	0.00E+00	5.87E-10	3.687	15.209	1.522	6.81
PA4873	-	Probable heat-shock protein	0.0563	0.0282	0.1690	2.28E-40	3.27E-195	1.04E-58	-17.750	-35.500	-5.917	-19.72
PA4874	*[psiF]*	Conserved hypothetical protein	0.1113	0.1053	0.2611	6.26E-12	2.17E-30	5.00E-24	-8.987	-9.493	-3.830	-7.44
PA4878	*brlR*	Probable transcriptional regulator BrlR	0.0645	0.0188	0.1613	4.25E-47	0.00E+00	3.24E-93	-15.500	-53.143	-6.200	-24.95
PA4890	*desT [yijC]*	DesT {transcriptional regulator}	3.4211	1.1579	1.7895	1.64E-18	1.84E-02	4.38E-03	3.421	1.158	1.789	2.12
PA4891	*ureE*	Urease accessory protein UreE	7.0000	4.2500	4.0000	1.25E-27	5.15E-10	4.01E-07	7.000	4.250	4.000	5.08
PA4892	*ureF*	Urease accessory protein UreF	6.0000	18.5000	3.0000	3.41E-15	3.44E-208	1.09E-03	6.000	18.500	3.000	9.17
PA4893	*ureG*	Urease accessory protein UreG	22.3333	19.0000	8.6667	0.00E+00	0.00E+00	5.21E-75	22.333	19.000	8.667	16.67
PA4894	-	Hypothetical protein [urease accessory protein UreJ]	5.7500	7.5000	4.5000	2.25E-21	1.12E-37	1.56E-11	5.750	7.500	4.500	5.92
PA4912	-	Branched-chain amino acid ABC transporter membrane protein	3.7778	6.8889	11.0000	4.75E-16	8.91E-65	1.23E-191	3.778	6.889	11.000	7.22
PA4916	*nrtR*	Nudix-related transcriptional regulator NrtR	0.1544	0.2013	0.2013	2.38E-05	4.67E-04	6.84E-45	-6.478	-4.967	-4.967	-5.47
PA4917	*nadD2*	Nicotinate mononucleotide adenylyltransferase NadD2	0.1141	0.0604	0.2215	7.10E-11	9.77E-39	1.62E-34	-8.765	-16.556	-4.515	-9.95
PA4918	*pcnA*	Nicotinamidase, PcnA	4.8471	6.4588	1.7882	4.11E-120	3.23E-238	2.89E-09	4.847	6.459	1.788	4.36
PA4920	*nadE*	NH3-dependent NAD synthetase {NAD synthetase}	17.1176	11.2222	1.1503	0.00E+00	0.00E+00	2.83E-04	17.118	11.222	1.150	9.83
PA4929	-	Hypothetical protein	0.0769	0.0513	0.2308	8.12E-20	2.93E-31	2.64E-32	-13.000	-19.500	-4.333	-12.28
PA4937	*rnr [vacB]*	Exoribonuclease RNase R [virulence protein VacB]	2.0000	4.3532	1.5957	1.59E-48	4.72E-304	1.54E-27	2.000	4.353	1.596	2.65
PA4956	*rhdA*	Thiosulfate:cyanide sulfurtransferase	9.6500	6.0500	1.5500	0.00E+00	5.00E-136	9.16E-04	9.650	6.050	1.550	5.75
PA4973	*thiC*	Thiamine biosynthesis protein ThiC	5.2041	4.0612	1.5102	1.47E-192	1.95E-107	3.11E-08	5.204	4.061	1.510	3.59
PA4978	-	Hypothetical protein	1.0000	1.4286	6.7143	3.95E-02	3.97E-03	9.56E-85	1.000	1.429	6.714	3.05
PA4991	-	Hypothetical protein	4.1667	3.2500	1.3333	1.14E-83	6.77E-47	7.45E-04	4.167	3.250	1.333	2.92
PA4995	-	Probable acyl-CoA dehydrogenase	1.9091	3.6364	4.6364	2.42E-06	9.48E-25	2.26E-39	1.909	3.636	4.636	3.39
PA5015	*aceE [aceA]*	Pyruvate dehydrogenase complex component E1 {pyruvate dehydrogenase subunit E1}	8.3107	10.4379	1.4463	0.00E+00	0.00E+00	2.18E-23	8.311	10.438	1.446	6.73
PA5016	*aceF [aceB]*	Dihydrolipoamide acetyltransferase [pyruvate dehydrogenase complex component E2]	6.4688	7.7695	1.4336	0.00E+00	0.00E+00	1.24E-16	6.469	7.770	1.434	5.22
PA5017	*dipA*	Hypothetical protein DipA	1.3065	2.9839	1.8065	1.28E-10	2.09E-68	2.63E-19	1.306	2.984	1.806	2.03
PA5020	-	Probabale acyl-CoA dehydrogenase	2.7857	7.6429	1.4286	4.67E-19	3.42E-196	2.75E-02	2.786	7.643	1.429	3.95
PA5023	*[ydiU]*	Conserved hypothetical protein	2.2000	3.4000	2.2000	1.08E-10	3.23E-27	9.36E-08	2.200	3.400	2.200	2.60
PA5026	-	Hypothetical protein	16.8400	16.3200	3.8400	0.00E+00	0.00E+00	4.98E-20	16.840	16.320	3.840	12.33
PA5036	*gltB [aspB]*	Glutamate synthase large chain precursor [GltS alpha chain] {subunit alpha}	3.8182	3.0000	1.7273	1.45E-93	3.75E-54	9.46E-13	3.818	3.000	1.727	2.85
PA5049	*rpmE*	50S ribosomal protein L31	0.1195	0.0509	0.4320	3.34E-10	1.46E-84	5.27E-05	-8.366	-19.651	-2.315	-10.11
PA5058	*phaC2 [phaC]*	Poly(3-hydroxyalkanoic acid) synthase 2 [PHA-polymerase 2]	0.1681	0.1770	0.1681	9.48E-04	2.44E-08	2.80E-75	-5.947	-5.650	-5.947	-5.85
PA5059	*[phaD]*	Probable transcriptional regulator	0.1442	0.0673	0.2019	6.26E-06	1.02E-26	2.89E-35	-6.933	-14.857	-4.952	-8.91
PA5062	-	Conserved hypothetical protein	1.8413	3.2222	1.5873	3.09E-06	1.15E-20	1.10E-02	1.841	3.222	1.587	2.22
PA5063	*ubiE*	Ubiquinone biosynthesis methyltransferase UbiE {ubiquinone/menaquinone biosynthesis methyltransferase}	6.4692	5.8077	2.4154	0.00E+00	4.95E-269	1.70E-31	6.469	5.808	2.415	4.90
PA5069	*tatB [mttA, yigT]*	Translocation protein TatB {sec-independent translocase}	2.3535	3.4848	2.6667	3.74E-18	3.35E-44	3.90E-21	2.354	3.485	2.667	2.84
PA5070	*tatC [mttB, yigU]*	Transport protein TatC {transporter TatC}	2.3559	2.7966	1.4576	7.56E-20	8.47E-29	2.61E-04	2.356	2.797	1.458	2.20
PA5090	*vgrG5*	T6SS protein VgrG5	1.1818	3.6364	2.4545	1.79E-03	1.14E-35	2.65E-12	1.182	3.636	2.455	2.42
PA5091	*hutG*	*N*-Formylglutamate amidohydrolase	1.3048	3.2095	3.9619	4.45E-08	1.04E-58	7.34E-95	1.305	3.210	3.962	2.83
PA5094	-	Probable ATP-binding component of ABC transporter	2.3623	10.6812	3.5072	6.04E-23	0.00E+00	1.19E-54	2.362	10.681	3.507	5.52
PA5096	-	Probable binding protein component of ABC transporter {ABC transporter}	1.5273	4.3273	8.7273	1.04E-08	1.79E-89	0.00E+00	1.527	4.327	8.727	4.86
PA5097	*[hutT]*	Probable amino acid permease {histidine ammonia-lyase}	1.5152	3.3636	4.4242	1.07E-07	1.94E-44	1.97E-82	1.515	3.364	4.424	3.10
PA5110	*fbp [cfxF, cbbF]*	Fructose-1,6-bisphosphatase	8.4014	8.6395	2.2993	0.00E+00	0.00E+00	4.52E-35	8.401	8.639	2.299	6.45
PA5114	-	Hypothetical protein	3.0000	1.9310	2.9310	5.49E-59	1.59E-20	5.40E-53	3.000	1.931	2.931	2.62
PA5115	-	Conserved hypothetical protein	0.0323	0.1290	0.5161	9.33E-89	1.89E-04	6.24E-03	-31.000	-7.750	-1.938	-13.56
PA5119	*glnA*	Glutamine synthetase	4.4783	4.8230	2.4859	0.00E+00	0.00E+00	3.56E-80	4.478	4.823	2.486	3.93
PA5129	*grxC [grx]*	Glutaredoxin GrxC	11.8889	12.8519	1.8148	0.00E+00	0.00E+00	2.38E-04	11.889	12.852	1.815	8.85

PA5130	*[yibN]*	Conserved hypothetical protein	9.2642	4.8302	2.3208	1.45E-287	1.67E-61	3.40E-09	9.264	4.830	2.321	5.47
PA5132	-	Hypothetical protein	1.8333	2.3333	2.1667	2.89E-03	4.73E-05	3.67E-03	1.833	2.333	2.167	2.11
PA5139	-	Hypothetical protein	3.2857	2.7143	1.7857	7.81E-15	4.53E-10	1.07E-02	3.286	2.714	1.786	2.60
PA5144	-	Hypothetical protein	7.0000	8.0000	5.0000	4.25E-07	5.56E-10	2.78E-04	7.000	8.000	5.000	6.67
PA5149.1	-	tRNA-Phe	4.6667	3.5000	13.0833	2.42E-09	2.19E-05	1.92E-98	4.667	3.500	13.083	7.08
PA5150	-	Probable short-chain dehydrogenase	4.6087	1.6304	13.3043	9.85E-75	4.65E-07	0.00E+00	4.609	1.630	13.304	6.51
PA5151	-	Hypothetical protein	2.7143	5.2857	4.0000	1.52E-06	8.39E-28	9.02E-13	2.714	5.286	4.000	4.00
PA5152	-	Probable ATP-binding component of ABC transporter	1.8780	4.9350	1.6179	1.25E-18	1.83E-176	2.22E-10	1.878	4.935	1.618	2.81
PA5153	-	Amino acid (lysine/arginine/ornithine/ histidine/octopine) ABC transporter periplasmic binding protein {amino acid ABC transporter substrate-binding protein}	1.4925	7.1434	1.3132	3.34E-20	0.00E+00	7.48E-13	1.492	7.143	1.313	3.32
PA5159	*[emrA, pmrA]*	Multidrug resistance protein	3.2609	3.6087	1.4783	3.70E-28	2.22E-35	1.14E-02	3.261	3.609	1.478	2.78
PA5164	*rmlC [rfbC]*	dTDP-4-dehydrorhamnose 3,5-epimerase [dTDP-4-keto-6-deoxyglucose 3,5-epimerase, dTDP-L-rhamnose synthetase]	0.1964	0.2224	0.3086	2.46E-02	2.44E-05	4.58E-14	-5.092	-4.495	-3.240	-4.28
PA5167	*dctP*	DctP {C4-dicarboxylate-binding protein}	6.0348	5.2261	5.3304	3.92E-317	8.19E-226	1.16E-233	6.035	5.226	5.330	5.53
PA5168	*dctQ*	DctQ {dicarboxylate transporter}	6.1471	7.0882	4.9412	1.94E-107	1.18E-152	3.36E-62	6.147	7.088	4.941	6.06
PA5169	*dctM*	DctM {C4-dicarboxylate transporter}	2.0541	2.1081	3.0811	1.08E-14	4.62E-15	2.71E-34	2.054	2.108	3.081	2.41
PA5171	*arcA*	Arginine deiminase	4.4608	4.5676	2.1615	0.00E+00	0.00E+00	1.71E-64	4.461	4.568	2.161	3.73
PA5172	*arcB*	Ornithine carbamoyltransferase	2.7603	3.3796	2.0676	1.24E-105	5.49E-171	2.24E-53	2.760	3.380	2.068	2.74
PA5179	-	Probable transcriptional regulator	2.8947	3.2632	2.6316	2.66E-15	1.09E-20	5.08E-10	2.895	3.263	2.632	2.93
PA5181.1	*P34*	P34	3.4118	3.5294	2.4706	2.78E-12	2.05E-13	8.95E-05	3.412	3.529	2.471	3.14
PA5182	-	Hypothetical protein	4.9762	5.6429	2.5714	1.09E-65	1.06E-88	4.58E-12	4.976	5.643	2.571	4.40
PA5183	-	Hypothetical protein	4.6667	7.1429	3.4286	2.01E-28	1.09E-76	1.59E-12	4.667	7.143	3.429	5.08
PA5203	*gshA*	Glutamate--cysteine ligase	3.2090	1.9104	1.8060	1.69E-68	5.51E-20	1.97E-15	3.209	1.910	1.806	2.31
PA5206	*argE*	Acetylornithine deacetylase	3.2593	2.0617	4.1975	7.61E-66	2.46E-22	1.06E-118	3.259	2.062	4.198	3.17
PA5212	-	Hypothetical protein	1.6614	4.5787	1.1024	2.97E-13	1.34E-136	1.02E-02	1.661	4.579	1.102	2.45
PA5213	*gcvP1*	Glycine cleavage system protein P1 [glycine decarboxylase] {glycine dehydrogenase}	0.1048	0.1619	0.2762	1.70E-12	5.58E-11	2.08E-19	-9.545	-6.176	-3.621	-6.45
PA5220	-	Hypothetical protein	0.0128	0.0495	0.0351	0.00E+00	6.13E-169	0.00E+00	-78.250	-20.194	-28.455	-42.30
PA5226	-	Hypothetical protein	4.2632	13.3860	2.4123	1.62E-63	0.00E+00	1.52E-14	4.263	13.386	2.412	6.69
PA5227	*[ygfE]*	Conserved hypothetical protein	4.0925	10.3006	2.6069	1.74E-79	0.00E+00	4.74E-25	4.092	10.301	2.607	5.67
PA5227.1	*ssrS*	6S RNA	3.4472	3.6273	3.0435	1.38E-34	6.25E-39	1.98E-23	3.447	3.627	3.043	3.37
PA5228	*[ygfA]*	Conserved hypothetical protein {5-formyltetrahydrofolate cyclo-ligase}	2.0204	2.4490	3.2041	2.30E-10	1.09E-15	3.50E-26	2.020	2.449	3.204	2.56
PA5246	*[yigI]*	Conserved hypothetical protein	2.1389	2.3056	2.3889	1.55E-08	5.87E-10	3.50E-08	2.139	2.306	2.389	2.28
PA5247	*[yaiI]*	Conserved hypothetical protein	2.1778	3.3333	3.0000	3.63E-10	4.31E-26	3.30E-18	2.178	3.333	3.000	2.84
PA5260	*hemC [popE]*	Porphobilinogen deaminase [hydroxymethylbilane synthase]	2.3566	4.3427	2.9441	4.53E-37	1.03E-155	1.05E-60	2.357	4.343	2.944	3.21
PA5263	*argH*	Argininosuccinate lyase	2.6200	1.4000	2.2700	2.29E-48	6.32E-11	9.37E-33	2.620	1.400	2.270	2.10
PA5267	*hcpB*	T6SS secreted protein Hcp	7.3333	65.5556	5.7778	4.01E-53	0.00E+00	2.34E-28	7.333	65.556	5.778	26.22
PA5269	-	Hypothetical protein	1.6420	1.4691	3.4938	7.82E-06	1.63E-04	1.05E-26	1.642	1.469	3.494	2.20
PA5270	-	Hypothetical protein	1.3953	2.9070	1.7442	8.23E-06	4.03E-27	3.42E-06	1.395	2.907	1.744	2.02
PA5276	*lppL*	Lipopeptide LppL precursor	3.0957	3.4043	3.1915	9.06E-16	1.13E-19	1.35E-14	3.096	3.404	3.191	3.23
PA5277	*lysA*	Diaminopimelate decarboxylase	1.8944	1.5915	2.5352	4.62E-24	1.46E-15	3.73E-45	1.894	1.592	2.535	2.01
PA5285	*sutA*	Hypothetical protein SutA	0.0710	0.1065	0.2288	3.57E-38	1.62E-20	6.25E-39	-14.083	-9.389	-4.371	-9.28
PA5289	-	Hypothetical protein	4.3088	6.2941	4.1176	8.01E-41	2.12E-99	4.94E-34	4.309	6.294	4.118	4.91
PA5304	*dadA*	D-Amino acid dehydrogenase, small subunit	3.9204	2.1903	2.8230	3.56E-173	7.96E-44	2.43E-78	3.920	2.190	2.823	2.98
PA5308	*lrp [dadR, dadAX]*	Leucine-responsive regulatory protein [transcriptional activator of *dadAX/dadR* , Lrp protein]	4.3816	3.9211	2.8816	1.30E-68	1.06E-52	2.60E-23	4.382	3.921	2.882	3.73
PA5309	*pauB4*	FAD-dependent oxidoreductase {oxidoreductase}	2.4412	1.5882	3.1471	2.08E-20	1.69E-07	4.70E-33	2.441	1.588	3.147	2.39
PA5311	-	Probable MFS transporter	3.3333	1.9167	4.4167	2.26E-17	1.68E-05	4.46E-31	3.333	1.917	4.417	3.22
PA5312	*pauC [kauB]*	Aldehyde dehydrogenase	8.1746	4.4841	4.9881	0.00E+00	6.37E-253	0.00E+00	8.175	4.484	4.988	5.88
PA5313	*gabT2 [paaT]*	Transaminase {omega amino acid--pyruvate transaminase}	13.4107	9.2857	6.3750	0.00E+00	0.00E+00	1.83E-282	13.411	9.286	6.375	9.69
PA5314	-	Hypothetical protein	3.8214	1.0893	6.5714	1.54E-33	1.38E-02	3.35E-116	3.821	1.089	6.571	3.83

PA5317	*dppA5*	Probable binding protein component of ABC dipeptide transporter	6.7397	8.3562	1.3836	0.00E+00	0.00E+00	1.67E-07	6.740	8.356	1.384	5.49
PA5320	*coaC [coaB, dfp, coaBC]*	Phosphopantothenoylcysteine synthase/(R)-4′-phospho-*N*-pantothenoylcysteine decarboxylase [CoaBC protein, Dfp protein] {bifunctional phosphopantothenoylcysteine decarboxylase/phosphopantothenate synthase}	3.6744	2.4186	1.7442	1.45E-58	5.11E-22	4.76E-08	3.674	2.419	1.744	2.61
PA5324	*sphR*	Sphingosine-responsive regulator, SphR {transcriptional regulator}	1.4722	3.3333	2.5000	1.96E-06	1.78E-37	8.17E-17	1.472	3.333	2.500	2.44
PA5344	*oxyR*	OxyR {transcriptional regulator}	3.8561	3.2446	2.0360	6.23E-116	3.65E-77	7.16E-24	3.856	3.245	2.036	3.05
PA5359	-	Hypothetical protein	0.1411	0.1290	0.4234	1.57E-06	2.82E-11	3.28E-06	-7.086	-7.750	-2.362	-5.73
PA5366	*pstB*	ATP-binding component of ABC phosphate transporter	3.6571	1.6714	1.2429	9.28E-64	3.03E-10	4.10E-03	3.657	1.671	1.243	2.19
PA5368	*pstC*	Membrane protein component of ABC phosphate transporter {phosphate ABC transporter permease}	13.8750	6.2500	2.7500	0.00E+00	5.75E-138	9.64E-19	13.875	6.250	2.750	7.63
PA5369	*pstS*	Phosphate ABC transporter, periplasmic phosphate-binding protein, PstS	8.8074	1.1407	2.1407	0.00E+00	1.68E-06	3.20E-27	8.807	1.141	2.141	4.03
PA5369.4	-	tRNA-Ile	0.0332	0.0062	0.1867	1.03E-148	0.00E+00	5.52E-33	-30.125	-160.667	-5.356	-65.38
PA5395	-	Conserved hypothetical protein	0.2000	0.2000	0.4571	4.01E-02	4.74E-02	1.88E-03	-5.000	-5.000	-2.188	-4.06
PA5403	-	Probable transcriptional regulator	4.1667	6.7500	2.0833	8.49E-12	4.94E-34	2.97E-02	4.167	6.750	2.083	4.33
PA5406	-	Hypothetical protein	2.2308	2.7692	2.3846	3.54E-04	3.27E-06	1.60E-03	2.231	2.769	2.385	2.46
PA5413	*ltaA*	Low specificity L-threonine aldolase	0.6543	0.8354	0.3045	2.33E-03	8.37E-04	3.20E-15	-1.528	-1.197	-3.284	-2.00
PA5431	-	Probable transcriptional regulator	1.7500	3.0000	3.2500	8.24E-04	3.87E-09	2.58E-09	1.750	3.000	3.250	2.67
PA5433	-	Conserved hypothetical protein	1.6111	1.0556	3.6667	3.50E-04	1.65E-03	1.57E-19	1.611	1.056	3.667	2.11
PA5437	-	Probable transcriptional regulator	9.9412	4.2941	2.7647	1.64E-264	2.28E-37	4.77E-11	9.941	4.294	2.765	5.67
PA5445	*[psecoA]*	Probable coenzyme A transferase	8.0000	3.7619	2.0238	0.00E+00	9.20E-71	3.20E-14	8.000	3.762	2.024	4.60
PA5460	-	Hypothetical protein	6.1136	12.4318	5.8182	7.40E-57	7.48E-303	8.76E-49	6.114	12.432	5.818	8.12
PA5469	-	Conserved hypothetical protein	1.7000	3.8000	2.6000	1.47E-03	3.06E-17	7.14E-06	1.700	3.800	2.600	2.70
PA5479	*gltP*	Proton-glutamate symporter [glutamate-aspartate symporter] {glutamate/aspartate: proton symporter}	0.1111	0.1389	0.4167	1.39E-09	2.51E-08	1.23E-06	-9.000	-7.200	-2.400	-6.20
PA5481	-	Hypothetical protein	0.1146	0.9108	0.4841	1.12E-10	5.15E-03	2.34E-04	-8.722	-1.098	-2.066	-3.96
PA5486	*[yghN]*	Conserved hypothetical protein	2.0500	4.4000	2.2500	7.82E-06	4.76E-31	2.93E-05	2.050	4.400	2.250	2.90
PA5498	*znuA*	ZnuA [probable adhesin] {adhesin}	4.0625	4.1875	4.4063	3.73E-49	1.80E-52	5.72E-57	4.063	4.188	4.406	4.22
PA5499	*zur [np20]*	Zinc uptake regulator, Zur [transcriptional regulator np20]	2.2016	2.3468	5.5806	1.16E-20	2.54E-23	8.89E-180	2.202	2.347	5.581	3.38
PA5500	*znuC [yebM]*	Zinc transport protein ZnuC	3.0000	2.9455	5.3818	4.10E-32	9.35E-31	1.88E-124	3.000	2.945	5.382	3.78
PA5514	-	Probable beta-lactamase	2.3846	4.4615	2.0000	6.53E-07	4.38E-28	2.14E-03	2.385	4.462	2.000	2.95
PA5515	-	Hypothetical protein	1.9231	2.6538	3.6154	1.38E-05	6.82E-11	3.51E-19	1.923	2.654	3.615	2.73
PA5522	*pauA6*	Glutamylpolyamine synthetase {glutamine synthetase}	3.6176	1.6176	4.7353	2.82E-51	3.02E-08	2.70E-94	3.618	1.618	4.735	3.32
PA5523	-	Probable aminotransferase	7.6364	8.5455	4.4545	0.00E+00	0.00E+00	2.39E-116	7.636	8.545	4.455	6.88
PA5524	-	Probable short-chain dehydrogenase	1.2000	3.0400	2.8000	3.59E-03	1.50E-19	6.89E-14	1.200	3.040	2.800	2.35
PA5530	-	C5-dicarboxylate transporter {MFS dicarboxylate transporter}	7.8000	87.2000	17.0000	3.73E-63	0.00E+00	0.00E+00	7.800	87.200	17.000	37.33
PA5531	*tonB1 [tonB]*	TonB1 { transporter TonB}	1.8387	3.9677	1.9032	7.66E-09	2.76E-48	1.03E-06	1.839	3.968	1.903	2.57
PA5536	*dksA2*	DksA2	12.0000	15.0000	10.0000	2.73E-56	1.31E-88	4.86E-35	12.000	15.000	10.000	12.33
PA5553	*atpC [papG, uncC]*	ATP synthase epsilon chain {subunit epsilon}	0.1284	0.0163	0.1723	8.98E-08	0.00E+00	2.71E-75	-7.786	-61.313	-5.805	-24.97
PA5564	*gidB*	Glucose inhibited division protein B [GidB] {16S rRNA methyltransferase GidB}	0.2174	0.1043	0.3652	3.70E-02	1.66E-12	1.35E-09	-4.600	-9.583	-2.738	-5.64
PA5567	*[thdF]*	Conserved hypothetical protein {tRNA modification GTPase TrmE}	3.0303	2.5455	3.0303	4.65E-33	3.16E-22	8.63E-31	3.030	2.545	3.030	2.87

Expression of genes by *P. aeruginosa* PAO1 grown in WBHVs for 4 h was compared with their expression when PAO1 was grown in LBB for 4 h. Genes were considered significantly differentially expressed when the average fold-change was ≥ 2.00 in the same direction and the *q* value for all three samples from healthy volunteers was ≤0.05. Light blue shading indicates genes that were upregulated in WBHV compared to LBB; those in light red shading were downregulated. MFS, major facilitator superfamily; OMP, outer membrane protein; ECF, extracytoplasmic function; MFP, membrane fusion protein; RND, resistance-nodulation-cell division; TAM, translocation and assembly; FAD; flavin adenine dinucleotide

*Gene numbers, names, and products were obtained from the *Pseudomonas* Genome DB; http://www.pseudomonas.com/

†Additional annotations from the Protein Table for *Pseudomonas aeruginosa* from the NIH; https://www.ncbi.nlm.nih.gov/genome/browse/#!/proteins/187/299953%7CPseudomonas%20aeruginosa%20PAO1/

‡Synonyms for gene names and/or products from the *Pseudomonas* Genome DB
