# Supplementary material for: During bacteremia, Pseudomonas aeruginosa PAO1 adapts by altering the expression of numerous virulence genes including those involved in quorum sensing
Source: PLoS One. 2020 Oct 15;15(10):e0240351. doi: 10.1371/journal.pone.0240351 (PMC7561203; doi:10.1371/journal.pone.0240351)
Supplement: S2 Table — (PDF) [file pone.0240351.s009.pdf]

S2 Table. PAO1 genes whose expression was inconsistent following growth in WBHVs compared to growth in LBB

Gene Number*	Gene Name*	Product*	Relative Expression WBHV:LBB			*q* Value			Fold Change (FC) WBHV:LBB		
			HV1	HV2	HV3	LBB:HV1	LBB:HV2	LBB:HV3	HV1	HV2	HV3
PA0033	*hptC*	Histidine phosphotransfer protein HptC	1.0238	0.0714	3.2143	2.47E-02	8.82E-19	2.02E-16	1.024	-14.000	3.214
PA0061	-	Hypothetical protein	0.9375	0.0833	2.1667	2.93E-02	1.59E-13	2.12E-07	-1.067	-12.000	2.167
PA0085	*hcp1*	T6SS Hcp1 {protein secretion apparatus assembly protein}[†]	2.3597	0.6827	5.5458	2.88E-53	2.67E-02	0.00E+00	2.360	-1.465	5.546
PA0106	*coxA [col]*	Cytochrome c oxidase subunit I	0.1494	2.0115	0.4138	1.95E-05	3.23E-25	6.60E-06	-6.692	2.011	-2.417
PA0107	-	Conserved hypothetical protein {cytochrome c oxidase assembly protein}	0.0964	2.3012	0.3735	3.85E-11	4.21E-18	2.27E-08	-10.375	2.301	-2.677
PA0194	-	Hypothetical protein	1.4000	0.2000	0.4000	3.90E-02	2.34E-03	6.99E-03	1.400	-5.000	-2.500
PA0210	*mdcC*	Malonate decarboxylase delta subunit	1.4333	0.2000	16.2000	7.54E-03	2.01E-02	0.00E+00	1.433	-5.000	16.200
PA0443	-	Probable transporter	0.2000	1.2000	0.4000	2.75E-02	3.80E-02	4.20E-02	-5.000	1.200	-2.500
PA0451	-	Conserved hypothetical protein	0.0000	0.2000	0.5000	0.00E+00	3.15E-02	5.48E-04	0.000	-5.000	-2.000
PA0460	-	Hypothetical protein	0.4909	0.1405	1.3777	4.94E-02	1.31E-16	2.66E-13	-2.037	-7.117	1.378
PA0557	-	Hypothetical protein	0.1176	0.0000	0.5294	1.95E-06	0.00E+00	5.41E-03	-8.500	0.000	-1.889
PA0602	-	Probable binding protein component of ABC transporter	1.1818	0.0303	1.5909	4.41E-06	9.32E-77	9.00E-08	1.182	-33.000	1.591
PA0614	-	Hypothetical protein	0.0278	0.0833	1.9722	3.28E-55	8.52E-14	7.59E-05	-36.000	-12.000	1.972
PA0617	-	Probable bacteriophage protein	0.0185	0.0802	1.2654	0.00E+00	1.48E-18	5.04E-03	-54.000	-12.462	1.265
PA0618	-	Probable bacteriophage protein	0.0850	0.0980	1.3464	2.70E-21	5.55E-21	1.13E-07	-11.769	-10.200	1.346
PA0619	-	Probable bacteriophage protein	0.1786	0.0774	1.3393	1.39E-03	6.67E-25	1.48E-05	-5.600	-12.923	1.339
PA0628	-	Conserved hypothetical protein	0.0375	0.0625	1.2125	2.67E-65	3.56E-35	8.95E-04	-26.667	-16.000	1.213
PA0636	-	Hypothetical protein	0.0532	0.2553	1.6489	4.32E-53	4.57E-03	2.17E-15	-18.800	-3.917	1.649
PA0638	-	Probable bacteriophage protein	0.1395	0.1977	1.4767	2.38E-06	4.72E-03	1.15E-05	-7.167	-5.059	1.477
PA0640	-	Probable bacteriophage protein	0.1170	0.1702	1.2234	1.43E-08	2.64E-04	7.49E-03	-8.545	-5.875	1.223
PA0653	*[yhfA]*	Conserved hypothetical protein	0.8902	0.1220	1.3293	1.45E-02	1.86E-06	2.25E-02	-1.123	-8.200	1.329
PA0668.1	-	16S ribosomal RNA	30.8116	306.9748	0.4937	1.14E-42	0.00E+00	1.12E-02	30.812	306.975	-2.025
PA0668.4	-	23S ribosomal RNA	297.4192	656.0873	0.4616	0.00E+00	0.00E+00	3.87E-02	297.419	656.087	-2.166
PA0787	-	Hypothetical protein	0.1250	0.1563	1.3125	3.79E-05	9.41E-04	1.73E-02	-8.000	-6.400	1.313
PA0837	*slyD*	Peptidyl-prolyl cis-trans isomerase SlyD [rotamase][‡]	0.9342	0.0932	2.0986	1.35E-05	5.96E-28	9.98E-29	-1.070	-10.735	2.099
PA0965	*ruvC*	Holliday junction resolvase RuvC {crossover junction endodeoxyribonuclease RuvC}	0.1980	0.0891	1.1881	6.01E-03	2.33E-13	2.03E-02	-5.050	-11.222	1.188
PA0996	*pqsA*	PqsA, probable coenzyme A ligase {anthranilate--CoA ligase}	1.6267	0.5760	0.0115	2.42E-30	4.03E-02	0.00E+00	1.627	-1.736	-86.800
PA1089	-	Conserved hypothetical protein	0.1154	0.1154	1.1154	1.60E-09	2.48E-10	9.81E-03	-8.667	-8.667	1.115
PA1131	-	Probable MFS transporter	0.0000	0.0333	0.1000	5.99E-224	2.69E-106	9.80E-121	0.000	-30.000	-10.000
PA1168	-	Hypothetical protein	0.0667	3.1778	0.0667	7.55E-17	7.36E-18	2.37E-114	-15.000	3.178	-15.000
PA1221	-	Hypothetical protein	0.0000	0.0588	0.0000	0.00E+00	2.46E-27	0.00E+00	0.000	-17.000	0.000
PA1274	*[bluB]*	Conserved hypothetical protein {5,6-dimethylbenzimidazole synthase}	0.2059	0.0000	0.4706	3.63E-02	0.00E+00	1.88E-03	-4.857	0.000	-2.125
PA1356	-	Hypothetical protein	0.0800	0.0000	0.1200	2.81E-08	0.00E+00	9.58E-74	-12.500	0.000	-8.333
PA1384	*galE*	UDP-glucose 4-epimerase [galactowaldenase]	0.2500	0.0000	0.2500	2.35E-02	7.14E-10	5.47E-03	-4.000	0.000	-4.000
PA1465	-	Hypothetical protein	0.1000	0.2000	1.5333	2.65E-12	6.42E-03	3.42E-05	-10.000	-5.000	1.533
PA1571	-	Hypothetical protein	3.2078	5.3290	0.5238	3.33E-36	3.24E-119	1.86E-03	3.208	5.329	-1.909
PA1632	*kdpF*	KdpF {Potassium-transporting ATPase F}	0.0000	6.0000	5.0000	0.00E+00	8.04E-06	3.18E-05	0.000	6.000	5.000
PA1746	-	Hypothetical protein	0.7438	0.0787	3.5746	3.73E-05	5.97E-70	4.38E-160	-1.344	-12.701	3.575
PA1796.2	-	tRNA-His	0.1489	0.0851	2.1489	4.32E-05	4.61E-15	6.83E-03	-6.714	-11.750	2.149
PA1821	-	Probable enoyl-CoA hydratase/isomerase	0.6667	0.1543	1.0617	9.90E-03	2.22E-08	2.69E-03	-1.500	-6.480	1.062
PA1844	*tse1*	T6SS effector protein Tse1	6.5714	0.0000	9.7143	1.18E-33	1.28E-09	2.27E-79	6.571	0.000	9.714
PA1896	-	Hypothetical protein	0.1463	1.0732	0.0976	2.28E-04	1.30E-03	4.37E-104	-6.833	1.073	-10.250
PA1897	-	Hypothetical protein	0.1509	1.4717	0.0943	2.68E-04	3.22E-06	3.56E-138	-6.625	1.472	-10.600
PA1958	-	Probable transporter	0.1111	0.2222	2.3333	6.69E-09	2.17E-02	1.70E-03	-9.000	-4.500	2.333
PA1963	-	Hypothetical protein	1.3390	0.0678	3.1864	2.19E-03	1.71E-16	1.09E-16	1.339	-14.750	3.186
PA2006	-	Probable MFS transporter	0.2105	0.0000	0.2105	1.34E-02	0.00E+00	9.10E-30	-4.750	0.000	-4.750
PA2016	*liuR [gnyR]*	Regulator of *liu* genes LiuR	2.4844	10.8542	0.4323	1.49E-31	0.00E+00	3.54E-06	2.484	10.854	-2.313
PA2069	-	Probable carbamoyl transferase	0.0035	0.0000	0.0069	0.00E+00	0.00E+00	0.00E+00	-289.000	0.000	-144.500
PA2070	-	Hypothetical protein	0.0000	0.1111	0.2222	3.78E-17	6.31E-03	2.58E-14	0.000	-9.000	-4.500
PA2098	-	Probable esterase/deacetylase	0.0000	0.2000	0.6000	5.63E-57	2.44E-02	1.81E-02	0.000	-5.000	-1.667
PA2196	-	TetR family transcriptional regulator	0.0000	0.0143	0.2143	0.00E+00	1.45E-245	2.90E-25	0.000	-70.000	-4.667
PA2300	*chiC*	Chitinase	0.0000	0.0515	0.0147	0.00E+00	4.70E-86	0.00E+00	0.000	-19.429	-68.000
PA2308	-	Probable ATP-binding component of ABC transporter	30.0000	0.0000	17.0000	6.84E-212	4.23E-02	2.99E-54	30.000	0.000	17.000

PA2331	-	Hypothetical protein	0.8364	0.1000	1.2955	4.07E-04	2.45E-18	2.32E-06	-1.196	-10.000	1.295
PA2536	*[ynbB]*	Probable phosphatidate cytidylyltransferase	0.7067	0.1333	2.5733	2.14E-02	1.08E-06	3.52E-29	-1.415	-7.500	2.573
PA2542	*[ytfN]*	Conserved hypothetical protein	0.1579	0.0526	1.0526	3.85E-04	1.51E-26	4.60E-02	-6.333	-19.000	1.053
PA2601	-	Probable transcriptional regulator	1.0000	0.0455	2.1818	1.74E-02	8.52E-92	5.64E-07	1.000	-22.000	2.182
PA2662	-	Conserved hypothetical protein	0.2059	0.0882	1.2941	1.05E-02	5.75E-13	1.66E-02	-4.857	-11.333	1.294
PA2701	-	Probable MFS transporter	0.0000	0.2273	0.1364	0.00E+00	3.09E-02	7.49E-57	0.000	-4.400	-7.333
PA2738	*himA*	Integration host factor subunit alpha	0.7090	0.1641	1.4303	6.98E-05	2.49E-14	9.92E-18	-1.410	-6.094	1.430
PA2743	*infC*	Translation initiation factor IF-3	0.5316	0.1130	1.0593	4.00E-03	5.37E-44	4.10E-10	-1.881	-8.849	1.059
PA2799	-	Hypothetical protein	0.1772	0.1456	1.1835	1.24E-03	1.24E-05	3.65E-02	-5.643	-6.870	1.184
PA2937	-	Hypothetical protein	0.1053	1.2719	0.3596	1.93E-09	2.85E-04	2.07E-08	-9.500	1.272	-2.780
PA2977	*murB*	UDP-*N*-acetylenolpyruvoylglucosamine reductase	0.1724	0.0000	0.4310	2.22E-03	0.00E+00	4.96E-06	-5.800	0.000	-2.320
PA3046	*[yggK]*	Conserved hypothetical protein	1.1812	0.0507	1.5507	7.18E-05	6.03E-53	2.61E-05	1.181	-19.714	1.551
PA3099	*xcpV [pddC]*	General secretion pathway protein I {T2SS pathway protein I}	0.0000	0.0676	0.3243	0.00E+00	1.70E-22	6.04E-09	0.000	-14.800	-3.083
PA3132	-	Probable hydrolase	0.0000	0.0385	0.5385	0.00E+00	3.01E-40	7.79E-03	0.000	-26.000	-1.857
PA3161	*himD*	Integration host factor subunit beta	0.9122	0.1048	1.6062	1.89E-04	3.92E-15	1.15E-10	-1.096	-9.541	1.606
PA3222	-	Hypothetical protein	0.1200	0.1200	2.2800	4.24E-07	1.44E-04	6.06E-09	-8.333	-8.333	2.280
PA3232	-	Probable nuclease {DNA polymerase III subunit epsilon}	4.4000	0.8667	27.2667	9.36E-26	1.23E-01	0.00E+00	4.400	-1.154	27.267
PA3245	*minE*	Cell division topological specificity factor MinE	0.6332	0.1293	1.2296	2.98E-02	1.18E-09	1.99E-04	-1.579	-7.735	1.230
PA3266	*capB [cspA]*	Cold acclimation protein B {major cold shock protein CspA}	0.6357	0.0342	5.2103	3.69E-02	5.50E-124	1.47E-187	-1.573	-29.214	5.210
PA3315	-	Probable ABC transporter permease {phosphonates ABC transporter permease}	0.0000	0.0345	0.2759	0.00E+00	2.26E-48	4.53E-11	0.000	-29.000	-3.625
PA3361	*lecB*	Fucose-binding lectin PA-IIL	0.0127	0.0000	0.0109	0.00E+00	0.00E+00	0.00E+00	-78.857	0.000	-92.000
PA3458	-	Probable transcriptional regulator	0.1737	0.1455	1.3803	5.41E-04	3.40E-08	8.78E-07	-5.757	-6.871	1.380
PA3465	*[yfiS]*	Conserved hypothetical protein	0.2000	0.1778	1.0741	2.15E-02	3.41E-08	5.50E-05	-5.000	-5.625	1.074
PA3530	*bfd*	Bacterioferritin-associated ferredoxin Bfd	2.2353	0.0588	8.5294	3.60E-04	1.58E-40	4.84E-75	2.235	-17.000	8.529
PA3549	*algJ*	Alginate o-acetyltransferase AlgJ {alginate o-acetylase AlgJ}	0.2000	0.0000	0.2000	3.78E-03	3.63E-05	3.91E-06	-5.000	0.000	-5.000
PA3568	*[ymmS]*	Probable acetyl-coa synthetase {propionyl-CoA synthetase}	0.7857	0.1327	1.5816	4.99E-04	2.36E-14	4.15E-14	-1.273	-7.538	1.582
PA3572	-	hypothetical protein	0.7034	0.1551	1.5483	5.20E-03	1.73E-08	2.86E-11	-1.422	-6.447	1.548
PA3688	-	Hypothetical protein	0.1603	0.1576	1.0625	9.77E-05	4.45E-08	3.08E-03	-6.237	-6.345	1.063
PA3690	-	Probable metal-transporting P-type ATPase	9.1233	6.8219	0.4110	0.00E+00	0.00E+00	3.48E-06	9.123	6.822	-2.433
PA3892	-	Conserved hypothetical protein	0.0000	0.1765	0.2941	1.50E-119	4.02E-03	2.84E-08	0.000	-5.667	-3.400
PA3909	*eddB*	Extracellular DNA degradation protein EddB	0.1250	0.0000	0.3750	3.85E-05	4.27E-25	2.31E-04	-8.000	0.000	-2.667
PA3931	-	Conserved hypothetical protein	12.2593	9.4074	0.3704	0.00E+00	4.00E-282	2.33E-06	12.259	9.407	-2.700
PA3956	-	Hypothetical protein	1.4286	0.0714	3.0000	1.93E-02	4.33E-13	4.09E-07	1.429	-14.000	3.000
PA4120	-	Probable transcriptional regulator	0.1667	0.0000	0.3333	6.27E-02	4.46E-44	3.34E-03	-6.000	0.000	-3.000
PA4131	-	Probable iron-sulfur protein	0.0000	0.0191	0.0090	0.00E+00	0.00E+00	0.00E+00	0.000	-52.471	-111.500
PA4154	*[ygiM]*	Conserved hypothetical protein	0.1429	0.0000	0.4286	6.89E-03	0.00E+00	5.24E-04	-7.000	0.000	-2.333
PA4160	*fepD*	Ferric enterobactin transporter FepD	0.0000	8.0000	7.0000	5.50E-84	1.00E-31	4.48E-23	0.000	8.000	7.000
PA4176	*ppiC2*	Peptidyl-prolyl cis-trans isomerase C2	0.8371	0.0950	1.4208	4.58E-03	1.44E-14	4.13E-05	-1.195	-10.524	1.421
PA4280.2	-	23S ribosomal RNA	331.2524	730.3691	0.4560	0.00E+00	0.00E+00	1.31E-02	331.252	730.369	-2.193
PA4280.5	-	16S ribosomal RNA	30.8116	306.9745	0.4937	1.14E-42	0.00E+00	1.12E-02	30.812	306.974	-2.025
PA4300	*tadC*	TadC {T2SS protein TacC}	0.0000	0.0405	0.0811	0.00E+00	1.25E-86	1.79E-227	0.000	-24.667	-12.333
PA4305	*rcpC*	RcpC {hypothetical protein}	0.0000	0.0000	0.0769	0.00E+00	0.00E+00	4.65E-219	0.000	0.000	-13.000
PA4351	*olsA*	OlsA {acyltransferase}	0.0833	0.0804	1.5298	6.79E-25	8.44E-46	2.28E-16	-12.000	-12.444	1.530
PA4352	-	Conserved hypothetical protein	0.1713	0.0664	1.1464	3.84E-02	2.19E-184	3.94E-13	-5.838	-15.066	1.146
PA4357	*[yhgG]*	Conserved hypothetical protein	0.0968	0.0269	1.2366	7.38E-12	3.12E-177	2.07E-02	-10.333	-37.200	1.237
PA4463	*[yhbH]*	Conserved hypothetical protein	0.5103	0.1660	1.5707	4.00E-03	6.66E-16	2.77E-28	-1.960	-6.024	1.571
PA4642	-	Hypothetical protein	1.7778	0.0833	1.9722	1.95E-04	1.25E-18	1.59E-03	1.778	-12.000	1.972
PA4680	-	Hypothetical protein	0.1000	1.2000	1.6500	7.02E-08	2.14E-02	4.85E-02	-10.000	1.200	1.650
PA4690.2	-	23S ribosomal RNA	297.4192	656.0876	0.4616	0.00E+00	0.00E+00	3.87E-02	297.419	656.088	-2.166
PA4690.5	-	16S ribosomal RNA	35.2443	351.9395	0.4946	1.77E-64	0.00E+00	9.18E-03	35.244	351.940	-2.022
PA4748	*tpiA [tpi]*	Triosephosphate isomerase	0.1493	0.0398	1.0597	2.89E-05	5.89E-176	1.78E-06	-6.700	-25.125	1.060
PA4755	*greA*	Transcription elongation factor GreA	0.5541	0.1081	1.2793	3.88E-02	2.46E-23	2.84E-08	-1.805	-9.250	1.279
PA4762	*grpE*	Heat shock protein GrpE [Hsp24]	1.4695	1.2542	0.0831	4.41E-18	5.22E-12	0.00E+00	1.469	1.254	-12.041
PA4843	*gcbA*	GcbA {two-component response regulator}	0.0945	1.3189	0.3110	6.29E-18	4.17E-15	2.49E-12	-10.583	1.319	-3.215
PA4919	*pncB1*	Nicotinate phosphoribosyltransferase	5.9340	3.7358	0.9906	.651245E-31	1.61E-106	1.87E-02	5.934	3.736	-1.010
PA5053	*hslV*	Heat shock protein HslV {ATP-dependent protease peptidase subunit}	1.1990	0.8827	0.1122	1.11E-07	3.09E-03	2.08E-145	1.199	-1.133	-8.909
PA5104	-	Conserved hypothetical protein	0.1667	0.0417	1.7396	5.02E-04	6.89E-56	1.25E-08	-6.000	-24.000	1.740

PA5127	*[yibK]*	Probable rRNA methylase	1.6087	0.0870	1.6087	8.18E-04	7.15E-08	3.83E-02	1.609	-11.500	1.609
PA5200	*amgR*	AmgR {osmolarity response regulator}	0.7029	0.0971	1.2571	5.43E-03	6.12E-20	5.29E-06	-1.423	-10.294	1.257
PA5202	-	Hypothetical protein	0.0811	1.2162	0.3514	1.40E-10	1.04E-02	4.56E-06	-12.333	1.216	-2.846
PA5240	*trxA*	Thioredoxin	0.6443	0.0891	1.4913	7.03E-04	4.31E-52	3.10E-18	-1.552	-11.221	1.491
PA5287	*amtB*	Ammonium transporter AmtB	4.6724	0.9483	20.7241	4.32E-138	1.65E-03	0.00E+00	4.672	-1.055	20.724
PA5333	-	Conserved hypothetical protein	0.7870	0.0185	1.6389	3.10E-02	0.00E+00	2.29E-05	-1.271	-54.000	1.639
PA5369.2	-	23S ribosomal RNA	297.4192	656.0876	0.4616	0.00E+00	0.00E+00	3.87E-02	297.419	656.088	-2.166
PA5369.5	-	16S ribosomal RNA	35.2319	351.8232	0.4945	1.96E-64	0.00E+00	9.17E-03	35.232	351.823	-2.022
PA5407	-	Hypothetical protein	0.1765	0.0980	2.4706	5.78E-03	9.31E-10	3.75E-08	-5.667	-10.200	2.471
PA5416	*soxB*	Sarcosine oxidase beta subunit	0.1333	1.1600	0.3200	2.10E-06	8.69E-06	1.02E-13	-7.500	1.160	-3.125
PA5436	-	Probable biotin carboxylase subunit of a transcarboxylase {acetyl-CoA carboxylase subunit alpha}	6.7462	1.7100	0.8278	0.00E+00	2.85E-29	1.00E-03	6.746	1.710	-1.208
PA5508	*pauA7*	Glutamylpolyamine synthetase homologue {glutamine synthetase}	0.0000	0.1111	0.3333	6.26E-87	6.37E-07	2.28E-04	0.000	-9.000	-3.000
PA5551	-	Hypothetical protein	0.1667	0.0333	1.4500	2.45E-03	6.77E-56	6.15E-03	-6.000	-30.000	1.450

Expression of genes by *P. aeruginosa* PAO1 grown in WBHVs for 4 h was compared with their expression when PAO1 was grown in LBB for 4 h. Evaluation of the genes originally found to be significantly up- or downregulated (1208 genes) revealed these 123 genes whose pattern of expression was not consistent among PAO1 grown in each of the three blood samples. Light blue shading indicates upregulation in WBHV compared to LBB; light red shading indicates downregulation; green shading indicates value of 0.

*Gene numbers, names, and products were obtained from the *Pseudomonas* Genome DB; http://www.pseudomonas.com/

†Additional annotations from the Protein Table for *Pseudomonas aeruginosa* from the NIH; https://www.ncbi.nlm.nih.gov/genome/browse/#!/proteins/187/299953%7CPseudomonas%20aeruginosa%20PAO1/

‡Synonyms for gene names and/or products from the *Pseudomonas* Genome DB
