# Supplementary material for: During bacteremia, Pseudomonas aeruginosa PAO1 adapts by altering the expression of numerous virulence genes including those involved in quorum sensing
Source: PLoS One. 2020 Oct 15;15(10):e0240351. doi: 10.1371/journal.pone.0240351 (PMC7561203; doi:10.1371/journal.pone.0240351)
Supplement: S3 Table — A. Nitrogen metabolism: amino acids, proteins, urea, and heterocyclic compounds. B. Sulfur metabolism and coenzyme, cofactor, and vitamin metabolism and biosynthesis. C. Carbon metabolism: glucose, other carbohydrates, tricarboxylic acid cycle, fatty acids and lipids, and carbon (general). D. Energy metabolism, respiration, and stress responses. (PDF) [file pone.0240351.s010.pdf]

S3 Table. PAO1 genes for metabolic and biosynthetic processes that were upregulated or downregulated by growth in WBHVs compared to growth in LBB.

Expression of genes by *P. aeruginosa* PAO1 grown in whole blood from healthy volunteers (WBHV) was compared with their expression when PAO1 was grown in LB broth (LBB) to an early log phase (reported in columns labeled "Average"). Blue shading, genes indicates whose expression was upregulated in WBHV compared to LBB; red shading, genes whose expression was downregulated. Changes in expression of all genes in the table were considered significant (fold change ≥ 2.00, *q* value ≤ 0.05). Gene numbers, names, and product functions were obtained from the *Pseudmonas* Genome DB (http://www.pseudomonas.com/); [ ], indicate function from *Pseudomonas* ortholog.

S3A Table. Nitrogen metabolism: amino acids, proteins, urea, and heterocyclic compounds

Amino Acid Metabolism and Biosynthesis

Gene #	*Name*	*Product Function*	*Average*
PA0018	*fmt*	Methionyl-tRNA formyltransferase	2.14
PA0035	*trpA*	Tryptophan synthase alpha chain	3.39
PA0036	*trpB*	Tryptophan synthase beta chain	6.14
PA0130	*bauC*	3-Oxopropanoate dehydrogenase	10.87
PA0131	*bauB*	BauB [beta-alanine degradation protein]	21.78
PA0132	*bauA*	Beta-alanine:pyruvate transaminase	24.31
PA0265	*davD*	Glutaric semialdehyde dehydrogenase	7.59
PA0266	*davT*	Delta-aminobutyrate aminotransferase	4.88
PA0292	*aguA*	Agmatine deiminase	4.23
PA0296	*spuI*	Glutamylpolyamine synthetase	4.56
PA0297	*spuA*	Probable glutamine amidotransferase	4.93
PA0298	*spuB*	Glutamylpolyamine synthetase	7.88
PA0299	*spuC*	Polyamine:pyruvate transaminase	4.95
PA0353	*ilvD*	Dihydroxy-acid dehydratase	2.86
PA0402	*pyrB*	Aspartate carbamoyltransferase	3.54
PA0407	*gshB*	Glutathione synthetase	2.97
PA0530	-	Probable pyridoxal PO4-dependent aminotransferase	3.59
PA0649	*trpG*	Anthranilate synthase component II	3.20
PA0838	*[btuE]*	Probable glutathione peroxidase	2.00
PA0879	-	Probable acyl-CoA dehydrogenase	3.33
PA1001	*phnA*	Anthranilate synthase component I, PhnA	-29.42
PA1002	*phnB*	Anthranilate synthase component II, PhnB	-9.61
PA1162	*dapE*	Succinyl-diaminopimelate desuccinylase	3.26
PA1187	-	Probable acyl-CoA dehydrogenase	8.87
PA1195	*ddaH*	Dimethylarginine dimethylaminohydrolase DdaH	4.13
PA1200	-	Conserved hypothetical protein	-3.87
PA1217	-	Probable 2-isopropylmalate synthase	-67.03
PA1254	*lhpC*	δ1-Pyrroline-4-OH-2-carboxylate deaminase, LhpC	16.78
PA1255	*lhpK*	D-Hydroxyproline epimerase, LhpK	9.11
PA1338	*ggt*	γ-Glutamyltranspeptidase precursor	2.32
PA1562	*acnA*	Aconitate hydratase 1	2.84
PA1566	*pauA3*	Glutamylpolyamine synthetase	9.67
PA1580	*gltA*	Citrate synthase	9.73
PA1681	*aroC*	Chorismate synthase	2.80
PA1736	-	Probable acetyl-CoA acetyltransferase	3.47
PA1737	-	Probable 3-hydroxyacyl-CoA dehydrogenase	3.84
PA1754	*cysB*	Transcriptional regulator CysB	5.24
PA1757	*thrH*	Homoserine kinase	3.38
PA1821	-	Probable enoyl-CoA hydratase/isomerase	-2.31
PA1843	*metH*	Methionine synthase	2.48
PA1901	*phzC2*	Phenazine biosynthesis protein PhzC2	-58.95
PA1927	*metE*	5-Methyltetrahydropteroyltriglutamate-homocysteine S-methyltransferase	59.07
PA2001	*atoB*	Acetyl-CoA acetyltransferase	-10.84
PA2007	*maiA*	Maleylacetoacetate isomerase	-9.18
PA2016	*liuR*	Regulator of *liu* genes LiuR	3.68
PA2025	*gor*	Glutathione reductase	2.68
PA2040	*pauA4*	Glutamylpolyamine synthetase	8.73
PA2069	-	Probable carbamoyl transferase	-144.50
PA2193	*hcnA*	Hydrogen cyanide synthase subunit HcnA	-120.12
PA2194	*hcnB*	Hydrogen cyanide synthase subunit HcnB	-80.21
PA2195	*hcnC*	Hydrogen cyanide synthase subunit HcnC	-113.66
PA2253	*ansA*	L-Asparaginase I	2.05
PA2317	-	Oxidoreductase	2.94
PA2397	*pvdE*	Pyoverdine biosynthesis protein PvdE	9.80
PA2413	*pvdH*	L-2,4-Diaminobutyrate:2-KG 4-aminotransferase, PvdH	13.1
PA2446	*gcvH2*	Glycine cleavage system protein H2	3.70
PA2470	*gtdA*	Gentisate 1,2-dioxygenase	2.87
PA2471	-	Conserved hypothetical protein	3.00
PA2473	-	Maleylpyruvate isomerase	3.33
PA2531	-	Probable aminotransferase	4.83
PA2550	-	Probable acyl-CoA dehydrogenase	4.83
PA2624	*idh*	Isocitrate dehydrogenase	
PA2667	*mvaU*	Transcriptional regulator MvaU	3.04
PA2776	-	Hypothetical protein	14.02
PA2796	*tal*	Transaldolase B	2.07
PA3001	-	Probable glyceraldehyde-3-phosphate dehydrogenase	-2.95
PA3068	*gdhB*	NAD-dependent glutamate dehydrogenase	-4.47
PA3082	*gbt*	Glycine betaine transmethylase	15.41
PA3117	*asd*	Aspartate-semialdehyde dehydrogenase	13.04
PA3118	*leuB*	3-Isopropylmalate dehydrogenase	10.62
PA3120	*leuD*	3-Isopropylmalate dehydratase small subunit	9.73
PA3121	*leuC*	3-Isopropylmalate dehydratase large subunit	10.34
PA3134	*gltX*	Glutamyl-tRNA synthetase	-2.65
PA3139	*[aspC]*	Probable amino acid aminotransferase	3.99
PA3151	*hisF2*	Imidazoleglycerol-phosphate synthase subunit HisF2	-12.54
PA3152	*hisH2*	Glutamine amidotransferase HisH2	-8.22
PA3175	*hutE*	Formimidoylglutamase HutE	6.44
PA3183	*zwf*	Glucose-6-phosphate 1-dehydrogenase	5.77
PA3195	*gapA*	Glyceraldehyde 3-phosphate dehydrogenase	3.13
PA3356	*pauA5*	Glutamylpolyamine synthetase	4.83
PA3366	*amiE*	Aliphatic amidase	-39.83
PA3418	*ldh*	Leucine dehydrogenase	-2.80
PA3506	-	Probable decarboxylase	7.83
PA3516	-	Probable lyase	-8.40
PA3517	-	Probable lyase	-20.50
PA3525	*argG*	Argininosuccinate synthase	5.46

Amino Acid Metabolism and Biosynthesis, continued

Gene #	*Name*	*Product Function*	*Average*
PA4758	*carA*	Carbamoyl-phosphate synthase small subunit	4.79
PA4773	*speD2*	Hypothetical protein SpeD2	3.62
PA4843	*gcbA*	GcbA, probable two-component response regulator	-4.16
PA4920	*nadE*	NH3-Dependent NAD synthetase	9.83
PA4956	*rhdA*	Thiosulfate:cyanide sulfurtransferase	5.75
PA5015	*aceE*	Pyruvate dehydrogenase complex component E1	6.73
PA5017	*dipA*	Hypothetical protein DipA	2.03
PA5036	*gltB*	Glutamate synthase large chain precursor	2.85
PA5091	*hutG*	*N*-Formylglutamate amidohydrolase	2.83
PA5119	*glnA*	Glutamine synthetase	3.93
PA5171	*arcA*	Arginine deiminase	3.73
PA5172	*arcB*	Ornithine carbamoyltransferase	2.74
PA5202	-	Hypothetical protein	-4.65
PA5203	*gshA*	Glutamate--cysteine ligase	2.31
PA5206	*argE*	Acetylornithine deacetylase	3.17
PA5213	*gcvP1*	Glycine cleavage system protein P1	-6.45
PA5263	*argH*	Argininosuccinate lyase	2.10
PA5277	*lysA*	Diaminopimelate decarboxylase	2.01
PA5304	*dadA*	D-Amino acid dehydrogenase, small SU	2.98
PA5312	*pauC*	Aldehyde dehydrogenase	5.88
PA5313	*gabT2*	Transaminase	9.69
PA5416	*soxB*	Sarcosine oxidase beta SU	-3.16
PA5436	-	Probable biotin transcarboxylase, carboxylase SU	2.42
PA5508	*pauA7*	Glutamylpolyamine synthetase homologue	-4.00
PA5522	*pauA6*	Glutamylpolyamine synthetase	3.32

Protein Processing and Metabolism

Gene #	*Name*	*Product Function*	*Average*
PA0074	*ppkA*	T6SS Ser/Thr protein kinase PpkA	2.18
PA0090	*clpV1*	T6SS protein ClpV1	2.89
PA0404	*[yggF]*	[Holliday junction resolvase]	4.50
PA0837	*slyD*	Peptidyl-prolyl cis-trans isomerase SlyD	-3.24
PA1162	*dapE*	Succinyl-diaminopimelate desuccinylase	3.26
PA1204	*[yieF]*	NAD(P)H quinone oxidoreductase	2.00
PA1338	*ggt*	Gamma-glutamyltranspeptidase precursor	2.32
PA1376	*aceK*	Bifunctional isocitrate dehydrogenase kinase/ phosphatase	4.89
PA1596	*htpG*	Heat shock protein HtpG	-20.08
PA1670	*stp1*	T6SS Ser/Thr phosphoprotein phosphatase Stp1	-25.76
PA1671	*stk1*	T6SS Ser/Thr kinase Stk1	-11.48
PA1736	-	Probable acetyl-CoA acetyltransferase	3.47
PA1796	*folD*	Bifunctional 5,10-methylene-tetrahydrofolate/5,10-methylene-	2.82
PA1801	*clpP*	ClpP [ATP-dependent Clp protease proteolytic subunit]	2.00
PA1847	*nfuA*	NfuA [Fe/S biogenesis protein]	2.64
PA2371	*clpV3*	T6SS probable ClpA/B-type protease ClpV3	-11.65
PA2578	-	Probable acetyltransferase	-18.78
PA2738	*himA*	Integration host factor alpha subunit	-2.02
PA2742	*rpmI*	50S Ribosomal protein L35	-5.44
PA2743	*infC*	Translation initiation factor IF-3	-3.22
PA2907	*cobL*	Precorrin-6y-dependent methyltransferase CobL	2.11
PA2939	*[pepB]*	Probable aminopeptidase [PepB]	-44.49
PA2975	*rluC*	Ribosomal large subunit pseudouridine synthase C	-2.17
PA2978	*ptpA*	Phosphotyrosine protein phosphatase	-14.40
PA3030	*mobA*	Molybdopterin-guanine dinucleotide biosynthesis, MobA	3.22
PA3126	*ibpA*	Heat-shock protein IbpA	-18.14
PA3161	*himD*	Integration host factor subunit beta	-3.01
PA3262	-	Probable peptidyl-prolyl cis-trans isomerase, FkbP-type	2.70
PA3326	*clpP2*	Probable ATP-dependent Clp protease ClpP2	-11.43
PA3363	*amiR*	Aliphatic amidase regulator	-13.75
PA3364	*amiC*	Aliphatic amidase expression-regulating protein	-11.00
PA3600	*[rpl36]*	[50S Ribosomal protein L36]	12.11
PA3601	*[ykgM]*	[50S Ribosomal protein L31]	37.73
PA3657	*map*	Methionine aminopeptidase	2.65
PA3810	*hscA*	Heat shock protein HscA	-5.02
PA3870	*moaA1*	Molybdopterin biosynthetic protein A1	4.93
PA3871	*[nifM]*	Probable PpiC-type peptidyl-prolyl cis-trans isomerase	10.85
PA4176	*ppiC2*	Peptidyl-prolyl cis-trans isomerase C2	-3.43
PA4216	*phzG1*	Probable pyridoxamine 5'-phosphate oxidase	-89.50
PA4242	*rpmJ*	50S Ribosomal protein L36	-5.20
PA4245	*rpmD*	50S Ribosomal protein L30	-2.56
PA4385	*groEL*	60-kDa Chaperonin GroEL	-17.95
PA4386	*groES*	10-kDa Chaperonin GroES	-15.76
PA4387	*[fxsA]*	[Possible phage exclusion suppressor FxsA]	-7.63
PA4420	*[mraW]*	[*S*-Adenosyl-methyltransferase MraW]	-4.46
PA4432	*rpsI*	30S Ribosomal protein S9	-11.12
PA4661	*pagL*	Lipid A 3-O-deacylase	-19.99
PA4668	*lolB*	Lipoprotein localization protein LolB	2.13
PA4741	*rpsO*	30S Ribosomal protein S15	-7.82
PA4760	*dnaJ*	Heat shock protein DnaJ	-12.36
PA4762	*grpE*	Heat shock protein GrpE	-3.11
PA4868	*ureC*	Urease alpha subunit	6.81
PA4873	-	Probable heat-shock protein	-19.72
PA4891	*ureE*	Urease accessory protein UreE	5.08
PA4892	*ureF*	Urease accessory protein UreF	9.17
PA4893	*ureG*	Urease accessory protein UreG	16.67
PA4894	-	Hypothetical protein [urease accessory protein UreJ]	5.92
PA5049	*rpmE*	50S Ribosomal protein L31	-10.11
PA5053	*hslV*	Heat shock protein HslV	-2.95
PA5132	-	Hypothetical protein	2.11

PA3570	*mmsA*	Methylmalonate-semialdehyde dehydrogenase	-3.06
PA3629	*adhC*	Alcohol dehydrogenase class III	-2.20
PA3658	*glnD*	Protein-PII uridylyltransferase	2.37
PA3659	*[dapC]*	Probable succinyldiaminopimelate transaminase	2.09
PA3710	-	Probable GMC-type oxidoreductase	-8.50
PA3792	*leuA*	2-Isopropylmalate synthase	14.19
PA3935	*tauD*	Taurine dioxygenase	5.88
PA4114	*[bltD]*	Lysine decarboxylase	3.32
PA4127	*hpcG*	2-oxo-hepta-3-ene-1,7-dioate hydratase	-16.23
PA4180	-	Probable acetolactate synthase large subunit	3.38
PA4212	*phzC1*	Phenazine biosynthesis protein PhzC1	-60.37
PA4309	*pctA*	Chemotactic transducer PctA	3.21
PA4310	*pctB*	Chemotactic transducer PctB	2.45
PA4315	*mvaT*	Transcriptional regulator MvaT, P16 subunit	4.54
PA4662	*murI*	Glutamate racemase	-6.98
PA4726	*cbrB*	Two-component response regulator CbrB	4.79
PA4748	*tpiA*	Triosephosphate isomerase	-10.26
PA4756	*carB*	Carbamoylphosphate synthetase large subunit	2.42

PA5240	*trxA*	Thioredoxin	-3.76
PA5260	*hemC*	Porphobilinogen deaminase	3.21
		Urea metabolism genes	
		**Heterocyclic Compound Metabolism**	
PA0660	-	NAD(P)H:quinone reductase	3.33
PA1546	*hemN*	Oxygen-dependent coproporphyrinogen III oxidase	2.33
PA1779	*[nasC]*	Assimilatory nitrate reductase	2.11
PA1780	*nirD*	Assimilatory nitrite reductase small subunit	4.44
PA1786	*nasS*	NasS [probable nitrate-binding protein]	2.33
PA2907	*cobL*	Precorrin-6y-dependent methyltransferase CobL	2.11
PA3872	*narI*	Respiratory nitrate reductase gamma chain	20.48
PA3873	*narJ*	Respiratory nitrate reductase delta chain	21.72
PA3874	*narH*	Respiratory nitrate reductase beta chain	17.89
PA3875	*narG*	Respiratory nitrate reductase alpha chain	20.17
PA3876	*narK2*	Nitrite extrusion protein 2	6.50
PA4676	*[yadF]*	Probable carbonic anhydrase	-3.31
PA5260	*hemC*	Porphobilinogen deaminase	3.21
PA5523	-	Probable aminotransferase	6.88

KG, ketoglutarate; Ser/Thr, serine/threonine; T6SS, type VI secretion system

S3B Table. Sulfur metabolism and coenzyme, cofactor, and vitamin metabolism and biosynthesis

**Sulfur Metabolism**			
Gene #	*Name*	*Product Function*	*Average*
PA0353	*ilvD*	Dihydroxy-acid dehydratase	2.86
PA0407	*gshB*	Glutathione synthetase	2.97
PA0500	*bioB*	Biotin synthase	9.17
PA0501	*bioF*	8-Amino-7-oxononanoate synthase	4.93
PA0589	*[glpE]*	[Thiosulfate sulfurtransferase]	2.70
PA0887	*acsA*	Acetyl-CoA synthetase	13.22
PA1338	*ggt*	γ-Glutamyltranspeptidase precursor	2.32
PA1546	*hemN*	Oxygen-dependent coproporphyrinogen III oxidase	2.33
PA1562	*acnA*	Aconitate hydratase 1	2.84
PA1796	*folD*	Bifunctional 5,10-methylene-tetrahydrofolate/5,10-methylene-	2.82
PA1813	*[gloB]*	Probable hydroxyacylglutathione hydrolase	3.05
PA1839	-	Hypothetical protein [RNA methyltransferase]	-11.27
PA1843	*metH*	Methionine synthase [5-methyltetrahydrofolate--homocysteine	2.48
PA1847	*nfuA*	NfuA [Fe/S biogenesis protein NfuA]	2.64
PA1927	*metE*	5-Methyltetrahydropteroyltriglutamate-homocysteine S-	59.07
PA2025	*gor*	Glutathione reductase	2.68
PA2193	*hcnA*	Hydrogen cyanide synthase subunit HcnA	-120.12
PA2566	-	Hypothetical protein	-34.62
PA3117	*asd*	Aspartate-semialdehyde dehydrogenase	5.34
PA3121	*leuC*	3-Isopropylmalate dehydratase large subunit	10.34
PA3444	*[ssuD]*	[Alkanesulfonate monooxygenase]	10.72
PA3445	-	Conserved hypothetical protein	4.80
PA3449	-	Conserved hypothetical protein	3.33
PA3639	*accA*	Acetyl-CoA carboxylase carboxyltransferase alpha SU	2.07
PA3667	-	Probable pyridoxal-phosphate dependent protein [cysteine	-5.69
PA3809	*fdx2*	Ferredoxin [2Fe-2S]	-4.16
PA3870	*moaA1*	Molybdopterin biosynthetic protein A1	4.93
PA3935	*tauD*	Taurine dioxygenase	5.88
PA3976	*thiE*	Thiamine-phosphate pyrophosphorylase	3.90
PA4044	*dxs*	1-Deoxy-D-xylulose-5-phosphate synthase	2.18
PA4130	-	Probable sulfite or nitrite reductas	-72.69
PA4692	-	[Sulfite oxidase subunit YedY]	3.21
PA4773	*speD2*	Hypothetical protein SpeD2	3.62
PA4848	*accC*	Biotin carboxylase	-2.15
PA4956	*rhdA*	Thiosulfate:cyanide sulfurtransferase	5.75
PA4973	*thiC*	Thiamine biosynthesis protein ThiC	3.59
PA5129	*grxC*	Glutaredoxin GrxC	8.85
PA5203	*gshA*	Glutamate--cysteine ligase	2.31
PA5240	*trxA*	Thioredoxin	-3.76
		**Cofactor, Coenzyme, and Vitamin Metabolism**	
PA0420	*bioA*	Adenosylmethionine-8-amino-7-oxononanoate aminotransferase	2.66
PA0500	*bioB*	Biotin synthase	9.17

**Cofactor, Coenzyme, and Vitamin Metabolism, continued**			
Gene #	*Name*	*Product Function*	*Average*
PA0501	*bioF*	8-Amino-7-oxononanoate synthase	4.93
PA1204	*[yieF]*	NAD(P)H quinone oxidoreductase	2.00
PA1546	*hemN*	Coproporphyrinogen III oxidase	2.33
PA1598	-	[3-Methyl-2-oxobutanoate hydroxymethyltransferase]	-9.59
PA1602	-	Probable oxidoreductase	22.50
PA1609	*fabB*	β-Oxoacyl-ACP synthase I FabB	3.54
PA1796	*folD*	Bifunctional 5,10-methylene-tetrahydrofolate dehydrogenase/	2.82
PA2025	*gor*	Glutathione reductase	2.68
PA2796	*tal*	Transaldolase B	2.07
PA2907	*cobL*	Precorrin-6y-dependent methyltransferase CobL	2.11
PA2951	*etfA*	Electron transfer flavoprotein subunit alpha	-2.71
PA3030	*mobA*	Molybdopterin-guanine dinucleotide biosynthesis protein MobA	3.22
PA3182	*pgl*	6-Phosphogluconolactonase	6.56
PA3387	*rhlG*	Beta-ketoacyl reductase {3-oxoacyl-ACP reductase}	8.17
PA3437	*folM*	Dihydromonapterin reductase, FolM	4.19
PA3438	*folE1*	GTP cyclohydrolase I	2.94
PA3439	*folX*	D-Erythro-7,8-dihydroneopterin triphosphate 2'-epimerase	6.93
PA3493	*[rnfG]*	Electron transport complex subunit G]	-11.25
PA3506	-	Probable decarboxylase	7.83
PA3570	*mmsA*	Methylmalonate-semialdehyde dehydrogenase	-3.06
PA3645	*fabZ*	(3R)-Hydroxymyristoyl-[acyl carrier protein] dehydratase	2.74
PA3667	-	Probable pyridoxal-phosphate dependent protein	-5.69
PA3870	*moaA1*	Molybdenum cofactor biosynthesis protein A	4.93
PA3975	*thiD*	Phosphomethylpyrimidine kinase	2.29
PA3976	*thiE*	Thiamine-phosphate pyrophosphorylase	3.90
PA3977	*hemL*	Glutamate-1-semialdehyde aminotransferase	2.16
PA4044	*dxs*	1-Deoxy-D-xylulose-5-phosphate synthase	2.18
PA4180	-	Probable acetolactate synthase large subunit	3.38
PA4376	*pncB2*	Nicotinate phosphoribosyltransferase	2.24
PA4524	*nadC*	Nicotinate-nucleotide pyrophosphorylase	2.28
PA4663	*moeB*	Molybdopterin biosynthesis protein MoeB	-7.24
PA4692	-	[Sulfite oxidase subunit YedY]	3.21
PA4835	*cntM*	Hypothetical protein	3.33
PA4836	*cntL*	Nicotianamine synthase-like enzyme	6.67
PA4837	*cntO*	TonB-dependent OMP ZmaA	27.93
PA4852	*[ydhG]*	Conserved hypothetical protein	-4.53
PA4918	*pcnA*	Nicotinamidase, PcnA	4.36
PA4919	*pncB1*	Nicotinate phosphoribosyltransferase	2.89
PA4920	*nadE*	NAD synthetase	9.83
PA4973	*thiC*	Thiamine biosynthesis protein ThiC	3.59
PA5260	*hemC*	Porphobilinogen deaminase	3.21
PA5320	*coaC*	Phosphopantothenoylcysteine synthase/(R)-4'-phospho-*N*-pantothenoylcysteine decarboxylase	2.61

S3C Table. Carbon metabolism: glucose, other carbohydrates, tricarboxylic acid cycle, fatty acids and lipids, and carbon (general)

**Glucose and Other Carbohydrate Metabolism and Biosynthesis**			
Gene #	*Name*	*Product Function*	*Average*
PA0130	*bauC*	3-Oxopropanoate dehydrogenase	10.87
PA0210	*mdcC*	Malonate decarboxylase delta SU	4.21
PA0211	*mdcD*	Malonate decarboxylase beta SU	3.46
PA0482	*glcB*	Malate synthase G	4.57
PA0763	*mucA*	Anti-sigma factor MucA	3.65
PA0887	*acsA*	Acetyl-CoA synthetase	13.22
PA1217	-	Probable 2-isopropylmalate synthase	-67.03
PA1376	*aceK*	Bifunctional isocitrate dehydrogenase kinase/phosphatase	4.89
PA1384	*galE*	UDP-glucose 4-epimerase	-2.67
PA1736	-	Probable acetyl-CoA acetyltransferase	3.47
PA1813	-	Probable hydroxyacylglutathione hydrolase	3.05
PA1950	*rbsK*	Ribokinase	-7.87
PA2001	*atoB*	Acetyl-CoA acetyltransferase	-10.84
PA2108	-	Probable decarboxylase	-8.00
PA2160	*[glgX]*	Probable glycosyl hydrolase	2.08
PA2199	-	Probable dehydrogenase	-7.01
PA2300	*chiC*	Chitinase	-29.14
PA2321	*gntK*	Gluconokinase GntK	7.11
PA2323	*gapN*	Glyceraldehyde-3-phosphate dehydrogenase GapN	6.77
PA2796	*tal*	Transaldolase B	2.07
PA2977	*murB*	UDP-*N*-Acetylenolpyruvoylglucosamine reductase	-2.71
PA3001	-	Probable glyceraldehyde-3-phosphate dehydrogenase	-2.95
PA3024	-	Probable carbohydrate kinase	2.36
PA3064	*pelA*	PelA protein	2.67
PA3118	*leuB*	3-Isopropylmalate dehydrogenase	13.04
PA3120	*leuD*	3-Isopropylmalate dehydratase small SU	9.73

**Tricarboxylic Acid Cycle Metabolism, continued**			
Gene #	*Name*	*Product Function*	*Average*
PA3121	*leuC*	3-Isopropylmalate dehydratase large SU	10.34
PA3181	*[edaA]*	2-Keto-3-deoxy-6-phosphogluconate aldolase	5.01
PA3356	*pauA5*	Glutamylpolyamine synthetase	4.83
PA3452	*mqoA*	Malate:quinone oxidoreductase	3.31
PA3506	-	Probable decarboxylase	7.83
PA3568	*[ymmS]*	Probable acetyl-coa synthetase	-2.41
PA3570	*mmsA*	Methylmalonate-semialdehyde dehydrogenase	-3.06
PA3639	*accA*	Acetyl-CoA carboxylase carboxyltransferase subunit alpha	2.07
PA3792	*leuA*	2-Isopropylmalate synthase	14.19
PA4180	-	Probable acetolactate synthase large subunit	3.38
PA4333	*[fumA]*	Probable fumarase	-2.55
PA4470	*fumC1*	Fumarate hydratase	10.31
PA4809	*fdhE*	FdhE [formate dehydrogenase epsilon SU]	3.38
PA4811	*fdnH*	Nitrate-inducible formate dehydrogenase, beta SU	3.74
PA5015	*aceE*	Pyruvate dehydrogenase complex component E1	6.73
PA5016	*aceF*	Dihydrolipoamide acetyltransferase	5.22
PA5020	-	Probabale acyl-CoA dehydrogenase	3.95
PA5119	*glnA*	Glutamine synthetase	3.93
PA5313	*gabT2*	Transaminase	9.69
PA5445	*[psecoA]*	Probable coenzyme A transferase	4.60
PA5508	*pauA7*	Glutamylpolyamine synthetase homologue	-4.00
PA5522	*pauA6*	Glutamylpolyamine synthetase	3.32
		**Fatty Acid and Lipid Metabolism and Biosynthesis**	
PA0879	-	Probable acyl-CoA dehydrogenase	3.33
PA1130	*rhlC*	Rhamnosyltransferase 2	-17.17
PA1187	-	Probable acyl-CoA dehydrogenase	8.87

PA3121	*leuC*	3-Isopropylmalate dehydratase large SU	10.34
PA3148	*wbpI*	UDP-*N*-Acetylglucosamine 2-epimerase WbpI	-11.42
PA3156	*wbpD*	UDP-2-Acetamido-3-amino-2,3-dideoxy-d-glucuronic acid N-acetyltransferase WbpD	-4.13
PA3181	*[edaA]*	2-Keto-3-deoxy-6-phosphogluconate aldolase	5.01
PA3182	*pgl*	6-Phosphogluconolactonase	6.56
PA3183	*zwf*	Glucose-6-phosphate 1-dehydrogenase	5.77
PA3194	*edd*	Phosphogluconate dehydratase	7.28
PA3195	*gapA*	Glyceraldehyde 3-phosphate dehydrogenase	3.13
PA3363	*amiR*	Aliphatic amidase regulator	-13.75
PA3506	-	Probable decarboxylase	7.83
PA3552	*arnB*	ArnB [UDP-4-amino-4-deoxy-L-arabinose--oxoglutarate	39.39
PA3553	*arnC*	ArnC, putative glycosyl transferase	41.02
PA3554	*arnA*	ArnA, [bifunctional UDP-glucuronic acid decarboxylase/UDP-4-amino-4-deoxy-L-arabinose formyltransferase]	17.58
PA3555	*arnD*	ArnD [4-deoxy-4-formamido-L-arabinose-	11.69
PA3559	-	Probable nucleotide sugar dehydrogenase	3.39
PA3570	*mmsA*	Methylmalonate-semialdehyde dehydrogenase	-3.06
PA3629	*adhC*	Alcohol dehydrogenase class III	-2.20
PA3639	*accA*	Acetyl-CoA carboxylase carboxyltransferase subunit alpha	2.07
PA3792	*leuA*	2-Isopropylmalate synthase	14.19
PA3896	-	Probable 2-hydroxyacid dehydrogenase	2.22
PA4180	-	Acetolactate synthase	3.38
PA4425	*[yraO]*	Sedoheptulose 7-phosphate isomerase GmhA	2.44
PA4695	*ilvH*	Acetolactate synthase 3 regulatory subunit	2.39
PA4748	*tpiA*	Triosephosphate isomerase	-10.26
PA4771	*lldD*	L-Lactate dehydrogenase	22.00
PA4848	*accC*	Biotin carboxylase [acetyl-CoA carboxylase]	-2.15
PA5015	*aceE*	Pyruvate dehydrogenase complex component E1	6.73
PA5016	*aceF*	Dihydrolipoamide acetyltransferase	5.22
PA5110	*fbp*	Fructose-1,6-bisphosphatase	6.45
		Lactate metabolism genes	
		**Tricarboxylic Acid Cycle Metabolism**	
PA0130	*bauC*	3-Oxopropanoate dehydrogenase	10.87
PA0132	*bauA*	Beta-alanine:pyruvate transaminase	24.31
PA0353	*ilvD*	Dihydroxy-acid dehydratase	2.04
PA0482	*glcB*	Malate synthase G	4.57
PA0530	-	Probable pyridoxal phosphate-dependent aminotransferase	3.59
PA0796	*prpB*	Carboxyphosphonoenolpyruvate phosphonomutase	-4.20
PA0854	*fumC2*	Fumarate hydratase	2.18
PA0879	-	Probable acyl-CoA dehydrogenase	3.33
PA0887	*acsA*	Acetyl-CoA synthetase	13.22
PA1187	-	Probable acyl-CoA dehydrogenase	8.87
PA1217	-	Probable 2-isopropylmalate synthase	-67.03
PA1376	*aceK*	Bifunctional isocitrate dehydrogenase kinase/phosphatase	4.89
PA1562	*acnA*	Aconitate hydratase 1	2.84
PA1566	*pauA3*	Glutamylpolyamine synthetase	9.67
PA1580	*gltA*	Citrate synthase	9.73
PA1736	-	Probable acetyl-CoA acetyltransferase	3.47
PA1737	-	Probable 3-hydroxyacyl-CoA dehydrogenase	3.84
PA1779	*[nasC]*	Assimilatory nitrate reductase	2.11
PA1821	-	Probable enoyl-CoA hydratase/isomerase	-2.31
PA1880	-	Probable oxidoreductase	-4.68
PA2001	*atoB*	Acetyl-CoA acetyltransferase	-10.84
PA2040	*pauA4*	Glutamylpolyamine synthetase	8.73
PA2446	*gcvH2*	Glycine cleavage system protein H2	3.70
PA2550	-	Probable acyl-CoA dehydrogenase	4.83
PA2564	*[tam]*	Hypothetical protein [trans-aconitate 2-methyltransferase]	-84.27
PA2624	*idh*	Isocitrate dehydrogenase	8.74
PA3117	*asd*	Aspartate-semialdehyde dehydrogenase	5.34
PA3118	*leuB*	3-Isopropylmalate dehydrogenase	13.04
PA3120	*leuD*	3-Isopropylmalate dehydratase small SU	9.73

PA1487	-	Probable carbohydrate kinase	2.82
PA1609	*fabB*	β-Oxoacyl-ACP synthase I FabB	3.54
PA1610	*fabA*	β-Hydroxydecanoyl-ACP dehydrase	5.16
PA1736	-	Probable acetyl-CoA acetyltransferase	3.47
PA1737	-	Probable 3-hydroxyacyl-CoA dehydrogenase	3.84
PA1821	-	Probable enoyl-CoA hydratase/isomerase	-2.31
PA1880	-	Probable oxidoreductase	-4.68
PA1897	-	Hypothetical protein	-5.25
PA1907	-	Hypothetical protein	-22.56
PA2001	*atoB*	Acetyl-CoA acetyltransferase	-10.84
PA2011	*liuE*	3-Hydroxy-3-methylglutaryl-CoA lyase	-4.51
PA2122	-	Hypothetical protein	3.08
PA2199	-	Probable dehydrogenase	-7.01
PA2393	-	Pyoverdine biosynthesis putative dipeptidase	25.00
PA2424	*pvdL*	Non-ribosomal peptide synthase PvdL	25.39
PA2536	*[ynbB]*	Probable phosphatidate cytidylyltransferase	-2.11
PA2537	-	Probable acyltransferase	2.02
PA2541	-	Probable CDP-alcohol phosphatidyltransferase	5.25
PA2550	-	Probable acyl-CoA dehydrogenase	4.83
PA2862	*lipA*	Lactonizing lipase precursor	11.93
PA3025	*[glpD2]*	Probable FAD-dependent glycerol-3-phosphate	2.53
PA3092	*fadH1*	2,4-Dienoyl-CoA reductase FadH1	6.08
PA3150	*wbpG*	LPS biosynthesis protein WbpG	-6.10
PA3153	*wzx*	O-Antigen translocase	-8.28
PA3154	*wzy*	B-Band O-antigen polymerase	-32.35
PA3328	-	Probable FAD-dependent monooxygenase	-239.25
PA3330	-	Probable short-chain dehydrogenase	-248.44
PA3333	*fabH2*	3-Oxoacyl-[acyl-carrier-protein] synthase III	-45.05
PA3334	*acp3*	Probable acyl carrier protein Acp3	-178.53
PA3387	*rhlG*	β-Ketoacyl reductase	8.17
PA3452	*mqoA*	Malate:quinone oxidoreductase	3.31
PA3478	*rhlB*	Rhamnosyltransferase chain B (RhlB)	-86.89
PA3506	-	Probable decarboxylase	7.83
PA3552	*arnB*	ArnB [UDP-4-amino-4-deoxy-L-arabinose--oxoglutarate aminotransferase]	39.39
PA3553	*arnC*	ArnC, putative glycosyl transferase	41.02
PA3554	*arnA*	ArnA, [bifunctional UDP-glucuronic acid decarboxylase/UDP-4-	17.58
PA3555	*arnD*	ArnD, [4-deoxy-4-formamido-L-arabinose-	11.69
PA3556	*arnT*	Inner membrane L-Ara4N transferase ArnT	3.75
PA3582	*glpK*	Glycerol kinase	4.35
PA3584	*glpD*	Glycerol-3-phosphate dehydrogenase	21.63
PA3639	*accA*	Acetyl-CoA carboxylase carboxyltransferase alpha SU	2.07
PA3645	*fabZ*	(3R)-Hydroxymyristoyl-[acyl carrier protein] dehydratase	2.74
PA3924	-	Probable medium-chain acyl-CoA ligase	4.15
PA3942	*tesB*	Acyl-CoA thioesterase II	4.67
PA4180	-	Probable acetolactate synthase large subunit	3.38
PA4351	*olsA*	OlsA [acyltransferase]	-7.64
PA4353	*[yajB]*	Conserved hypothetical protein	2.04
PA4661	*pagL*	Lipid A 3-O-deacylase	-19.99
PA4788	-	Hypothetical protein	8.07
PA4813	*lipC*	Lipase LipC	2.87
PA4890	*desT*	DesT [transcriptional regulator of FA metabolism]	2.12
PA5020	-	Probable acyl-CoA dehydrogenase	3.95
PA5023	*[ydiU]*	Conserved hypothetical protein	2.60
PA5058	*phaC2*	Poly(3-hydroxyalkanoic acid) synthase 2	-5.85
PA5445	*[psecoA]*	Probable coenzyme A transferase	4.60
PA5524	-	Probable short-chain dehydrogenase	2.35
		**Carbon Metabolism (General)**	
PA1757	*thrH*	Homoserine kinase	5.24
PA1796	*folD*	Bifunctional 5,10-methylene-tetrahydrofolate/5,10-methylene-	2.82
PA5213	*gcvP1*	Glycine cleavage system protein P1	-6.45

LPS, lipopolysaccharide; SU, subunit; UDP, uridine diphosphate

S3D Table. Energy metabolism, respiration, and stress responses

		**Generation of Energy Metabolites and Respiration**	
Gene #	*Name*	*Product Function*	*Average*
PA0007	-	Hypothetical protein	-6.86
PA0106	*coxA*	Cytochrome c oxidase subunit I	-2.417
PA0107	-	Conserved hypothetical protein	-2.677
PA0482	*glcB*	Malate synthase G	4.57
PA0541	-	Hypothetical protein	-11.03
PA0854	*fumC2*	Fumarate hydratase	2.18
PA1104	*fliI*	Flagellum-specific ATP synthase	-10.05
PA1317	*cyoA*	Cytochrome o ubiquinol oxidase subunit II	-6.71
PA1319	*cyoC*	Cytochrome o ubiquinol oxidase subunit III	-5.56
PA1320	*cyoD*	Cytochrome o ubiquinol oxidase subunit IV	-8.03
PA1376	*aceK*	Bifunctional isocitrate dehydrogenase kinase/phosphatase	4.89
PA1482	*ccmH*	Cytochrome c biogenesis protein CcmH	-4.45
PA1483	*cycH*	Cytochrome c biogenesis protein CycH	-5.90
PA1546	*hemN*	Oxygen-dependent coproporphyrinogen III oxidase	2.33
PA1562	*acnA*	Aconitate hydratase 1	2.84
PA1580	*gltA*	Citrate synthase	9.73
PA1600	-	Probable cytochrome C	10.89
PA1601	-	Probable aldehyde dehydrogenase	15.67
PA1602	-	Probable oxidoreductase	22.50
PA1779	*[nasC]*	Assimilatory nitrate reductase	2.11
PA1780	*nirD*	Assimilatory nitrite reductase small subunit	4.44
PA1847	*nfuA*	NfuA [Fe/S biogenesis protein NfuA]	2.64
PA2025	*gor*	Glutathione reductase	2.68
PA2379	-	Probable oxidoreductase	-6.76
PA2193	*hcnA*	Hydrogen cyanide synthase subunit HcnA	-120.12
PA2194	*hcnB*	Hydrogen cyanide synthase subunit HcnB	-80.21
PA2195	*hcnC*	Hydrogen cyanide synthase subunit HcnC	-113.66
PA2481	-	Hypothetical protein [probable cytochrome c class I]	4.81

		**Generation of Energy Metabolites and Respiration, continued**	
Gene #	*Name*	*Product Function*	*Average*
PA4917	*nadD2*	Nicotinate mononucleotide adenylyltransferase NadD2	-9.95
PA4918	*pcnA*	Nicotinamidase, PcnA	4.36
PA4919	*pncB1*	Nicotinate phosphoribosyltransferase	2.89
PA4920	*nadE*	NH3-dependent NAD synthetase	9.83
PA5015	*aceE*	Pyruvate dehydrogenase complex component E1	6.73
PA5016	*aceF*	Dihydrolipoamide acetyltransferase	5.22
PA5063	*ubiE*	Ubiquinone biosynthesis methyltransferase UbiE	4.90
PA5129	*grxC*	Glutaredoxin GrxC	8.85
PA5240	*trxA*	Thioredoxin	-3.76
PA5531	*tonB1*	TonB1 transporter	-11.52
PA5553	*atpC*	ATP synthase epsilon chain {subunit epsilon}	-24.97
PA5559	*atpE*	ATP synthase F0F1 subunit C	-7.12
		Aerobic respiration	
		Aerobic respiratory chain	
		Aerobic electron transport chain	
		Anaerobic respiration	
		ATP generation	
		Electron transport chain	
		**Responses to Stress**	
PA0404	*[yggF]*	[Holliday junction resolvase]	4.50
PA0961	-	Probable cold-shock protein	3.06
PA0965	*ruvC*	Holliday junction resolvase RuvC	-5.03
PA0968	*[ybgC]*	Conserved hypothetical protein	-9.75
PA1159	-	Probable cold-shock protein	-24.09
PA1518	-	Conserved hypothetical protein	3.49
PA1562	*acnA*	Aconitate hydratase 1	2.84
PA1596	*htpG*	Heat shock protein HtpG	-20.08
PA1602	-	Probable oxidoreductase	22.50

PA2482	-	Probable cytochrome C	7.15
PA2951	*etfA*	Electron transfer flavoprotein subunit alpha	-2.71
PA3032	*snr1*	Cytochrome C Snr1	-23.99
PA3195	*gapA*	Glyceraldehyde 3-phosphate dehydrogenase	3.13
PA3331	-	Cytochrome P450	-66.30
PA3452	*mqoA*	Malate:quinone oxidoreductase	3.31
PA3493	*[rnfG]*	[Electron transport complex subunit G]	-11.25
PA3500	-	Conserved hypothetical protein	4.17
PA3525	*argG*	Argininosuccinate synthase	5.46
PA3530	*bfd*	Bacterioferritin-associated ferredoxin Bfd	-2.08
PA3582	*glpK*	Glycerol kinase	4.35
PA3639	*accA*	Acetyl-CoA carboxylase carboxyltransferase subunit alpha	2.07
PA3809	*fdx2*	Ferredoxin [2Fe-2S]	-4.16
PA3872	*narI*	Respiratory nitrate reductase gamma chain	20.48
PA3873	*narJ*	Respiratory nitrate reductase delta chain	21.72
PA3874	*narH*	Respiratory nitrate reductase beta chain	17.89
PA3875	*narG*	Respiratory nitrate reductase alpha chain	20.17
PA3876	*narK2*	Nitrite extrusion protein 2	6.50
PA4130	-	Probable sulfite or nitrite reductase	-72.69
PA4131	-	Probable iron-sulfur protein	-54.66
PA4132	-	Conserved hypothetical protein	-66.39
PA4133	*[ccoN]*	Cytochrome c oxidase subunit I (cbb3-type)*	-110.44
PA4057	*nrdR*	Transcriptional regulator NrdR	2.55
PA4376	*pncB2*	Nicotinate phosphoribosyltransferase	2.24
PA4465	*[ybhJ]*	Conserved hypothetical protein	-5.90
PA4470	*fumC1*	Fumarate hydratase	10.31
PA4524	*nadC*	Nicotinate-nucleotide pyrophosphorylase	2.28
PA4619	-	Probable c-type cytochrome	3.95
PA4620	-	Hypothetical protein	8.81
PA4748	*tpiA [tpi]*	Triosephosphate isomerase	-10.26
PA4756	*carB*	Carbamoylphosphate synthetase large subunit	2.42
PA4758	*carA*	Carbamoyl-phosphate synthase small subunit	4.79
PA4772	-	Probable ferredoxin	12.81
PA4916	*nrtR*	Nudix-related transcriptional regulator NrtR	-5.47

PA1933	*[yagR]*	Probable hydroxylase large subunit	-5.24
PA2507	*catA*	Catechol 1,2-dioxygenase	5.50
PA2622	*cspD*	Cold-shock protein CspD	-2.34
PA3266	*capB*	Cold acclimation protein B	-8.53
PA3331	-	Cytochrome P450	-66.30
PA3810	*hscA*	Heat shock protein HscA	-5.02
PA3871	*[nifM]*	Probable PpiC-type peptidyl-prolyl cis-trans isomerase	10.85
PA4010	-	[3-methyladenine DNA glycosylase]	3.06
PA4366	*sodB*	Superoxide dismutase	-2.17
PA4385	*groEL*	60-kDa chaperonin GroEL	-17.95
PA4386	*groES*	10-kDa chaperonin GroES	-15.76
PA4468	*sodM*	Superoxide dismutase	9.12
PA4470	*fumC1*	Fumarate hydratase	10.31
PA4760	*dnaJ*	Heat shock protein DnaJ	-12.36
PA4762	*grpE*	Heat shock protein GrpE	-3.11
PA4873	-	Probable heat-shock protein	-19.72
PA4874	*[psiF]*	[Phosphate starvation-induced PsiF]	-7.44
PA5053	*hslV*	Heat shock protein HslV	-2.95
PA5240	*trxA*	Thioredoxin	-3.76
		**Homeostasis**	
PA2025	*gor*	Glutathione reductase	2.68
PA2398	*fpvA*	Ferripyoverdine receptor	10.92
PA3581	*glpF*	Glycerol uptake facilitator protein	47.50
PA4034	*aqpZ*	Aquaporin Z	2.09
PA4221	*fptA*	Fe(III)-pyochelin outer membrane receptor precursor	2.29
PA5129	*grxC*	Glutaredoxin GrxC	8.85
PA5240	*trxA*	Thioredoxin	-3.76
PA5531	*tonB1*	TonB1 transporter	2.57

Cold shock proteins
Heat shock proteins and chanperones
Homeostasis
Oxidative stress
Response to diverse environments
Response to stress in general
