# Supplementary material for: During bacteremia, Pseudomonas aeruginosa PAO1 adapts by altering the expression of numerous virulence genes including those involved in quorum sensing
Source: PLoS One. 2020 Oct 15;15(10):e0240351. doi: 10.1371/journal.pone.0240351 (PMC7561203; doi:10.1371/journal.pone.0240351)
Supplement: S4 Table — (PDF) [file pone.0240351.s011.pdf]

**S4 Table. PAO1 genes encoding virulence factors upregulated or downregulated by growth in WBHVs compared to growth in LBB.**

| Gene Number | Gene Name | Product / function [from orthologs] | Average Fold change | *q* Value |
|---|---|---|---|---|
| | | *Hydrogen cyanide synthesis* | | |
| PA2193 | ***hcnA*** | Hydrogen cyanide synthase HcnA | **-120.12** | **0.00E+00** |
| PA2194 | ***hcnB*** | Hydrogen cyanide synthase HcnB | **-80.21** | **0.00E+00** |
| PA2195 | ***hcnC*** | Hydrogen cyanide synthase HcnC | **-113.66** | **0.00E+00** |
| *PA2196** | **-** | TetR family transcriptional regulator | **-37.33** | **1.45E-25** |
| *Values based on three replicates each from two HVs. | | | | |
| | | *Proteases* | | |
| PA0372 | **-** | Zinc protease | **6.34** | **2.76E-06** |
| PA0423 | ***pasP*** | Putative extracellular protease PasP | **-3.38** | **6.84E-03** |
| PA0779 | *asrA* | AsrA [aminoglycoside-induced stress response ATP-dependent protease] | -9.25 | 0.297 |
| **PA1130** | *rhlC* | Rhamnosyltransferase 2 | **-17.17** | **4.39E-40** |
| PA1245 | ***aprX*** | Hypothetical protein AprX | **-2.48** | **0.033** |
| PA1246 | *aprD* | Alkaline protease secretion protein AprD | -21.17 | 0.140 |
| PA1247 | ***aprE*** | Alkaline protease secretion protein AprE | **-27.61** | **0.030** |
| PA1248 | ***aprF*** | Alkaline protease secretion OMP AprF | **-6.62** | **0.010** |
| PA1249 | ***aprA*** | Alkaline metalloproteinase | **-13.87** | **0.001** |
| PA1250 | ***aprI*** | Alkaline proteinase inhibitor AprI | **-11.83** | **0.010** |
| PA1871 | ***lasA*** | LasA protease | **-39.44** | **3.40E-104** |
| *PA2300** | ***chiC*** | Chitinase* | **-43.71** | **2.35E-86** |
| PA2862 | ***lipA*** | Lactonizing lipase, extracellular | **11.93** | **2.46E-30** |
| PA2939 | **-** | Aminopeptidase, extracellular | **-44.49** | **0.00E+00** |
| PA3478 | ***rhlB*** | Rhamnosyltransferase subunit B | **-86.89** | **0.00E+00** |
| PA3479 | ***rhlA*** | Rhamnosyltransferase subunit A | **-96.34** | **0.00E+00** |
| PA3724 | ***lasB*** | Elastase LasB | **-223.32** | **0.00E+00** |
| *Values based on three replicates each from two HVs. | | | | |
| | | *Toxins and Extracellular Secreted Proteins* | | |
| PA0041 | - | Probable hemagglutinin | -2.22 | 0.055 |
| PA0044 | ***exoT*** | Exoenzyme T | **21.78** | **0.00E+00** |
| PA0122 | ***rahU*** | Hemolysin | **-27.04** | **1.55E-16** |
| PA0843 | *plcR* | Phospholipase accessory protein PlcR | 2.21 | 0.273 |
| PA0844 | ***plcH*** | Hemolytic phospholipase C | **4.17** | **0.033** |
| PA1148 | *toxA* | Exotoxin A | 4.47 | 0.229 |
| PA2191 | ***exoY*** | Exoenzyme Y adenylate cyclase | **9.00** | **1.50E-09** |
| PA2302* | ***ambE*** | AmbE [nonribosomal peptide synthetase] [1] | **-29.94** | **4.23E-03** |
| PA2303 | *ambD* | AmbD [putative regulatory protein] [1] | -14.05 | 0.144 |
| PA2304 | ***ambC*** | AmbC [putative regulatory protein] [1] | **-10.42** | **0.013** |
| PA2305 | ***ambB*** | AmbB [nonribosomal peptide synthetase] [1] | **-8.38** | **0.021** |
| *PA2308* † | **-** | Probable ABC transporter ATP-binding protein | **23.50** | **1.49E-54** |
| PA2309 | **-** | Probable ABC transporter substrate-binding protein | **6.25** | **1.04E-04** |
| *PA2570* † | *lecA* | LecA protein (PA-I galactophilic lectin) | **-24.75** | **3.22E-76** |
| *PA3361* † | ***lecB*** | Fucose-binding lectin PA-IIL, LecB | **-85.43** | **0.00E+00** |
| PA3841 | ***exoS*** | Exoenzyme S | **15.36** | **0.00E+00** |
| *PA3909* † | ***eddB*** | Extracellular DNA degradation protein EddB | **-5.33** | **1.35E-04** |
| PA3296 | ***phoA*** | alkaline phosphatase | **5.83** | **3.91E-02** |
| PA4142 | **-** | Probable secretion protein [2] | **-11.19** | **2.06E-05** |
| PA4143 | **-** | Probable toxin transporter [2] | **-3.11** | **0.008** |
| PA4144 | - | Probable outer membrane protein precursor [2] | -4.00 | 0.606 |
| PA4833 | **-** | Putative hemolysin III | **2.05** | **2.71E-07** |
| *Genes 2302-2305 and 2308-2309 encode putative antimetabolite toxin synthesis and secretion operons, respectively. | | | | |
| †Values based on three replicates each from two HVs. | | | | |
| | | *Alginate and Exopolysaccharide* | | |
| PA0763 | ***mucA*** | Anti-sigma factor MucA | **3.65** | **1.39E-49** |
| PA3548 | *algI* | Alginate o-acetyltransferase AlgI | 2.11 | 0.325 |
| *PA3549** | ***algJ*** | Alginate o-acetyltransferase AlgJ* | **-5.00** | **0.002** |
| *Values based on three replicates each from two HVs. | | | | |
| | | *Drug Resistance Proteins (not related to Mex efflux pumps)* | | |
| PA0004 | ***gyrB*** | DNA gyrase subunit B | **2.21** | **2.65E-28** |

| | | | | |
|---|---|---|---|---|
| PA0779 | *asrA* | Aminoglycoside-induced stress response ATP-dependent | -9.25 | 0.297 |
| PA1000 | ***pqsE*** | Quinolone signal response protein | **-54.83** | **3.76E-23** |
| PA1001 | ***phnA*** | Anthranilate synthase component I | **-29.42** | **1.60E-08** |
| PA1002 | ***phnB*** | Anthranilate synthase component II | **-9.61** | **0.001** |
| PA1179 | ***phoP*** | Two-component response regulator PhoP [3] | **5.02** | **5.89E-163** |
| PA1180 | ***phoQ*** | Two-component sensor PhoQ [3] | **2.84** | **6.39E-52** |
| PA1801 | ***clpP*** | ATP-dependent Clp protease proteolytic subunit | **2.00** | **3.11E-29** |
| PA1847 | ***nfuA*** | NfuA | **2.64** | **6.39E-52** |
| PA1874 | **-** | Ortholog of probable serine protease PA3535 [4] | **-7.46** | **1.14E-03** |
| PA1875 | ***[ompL]*** | Probable OMP precursor [4] | **-23.92** | **4.41E-30** |
| PA1876 | **-** | Probable ATP-binding/permease fusion ABC transporter [4] | **-36.83** | **2.15E-142** |
| PA1877 | **-** | Probable secretion protein [4] | **-13.78** | **1.93E-17** |
| PA1899 | ***phzA2*** | Phenazine biosynthesis protein | **-126.30** | **2.40E-18** |
| PA1900 | ***phzB2*** | Phenazine biosynthesis protein | **-338.27** | **0.00E+00** |
| PA2272 | ***pbpC*** | Penicillin-binding protein 3A | **-7.78** | **5.99E-03** |
| PA2385 | ***pvdQ*** | 3-Oxo-C12-homoserine lactone acylase PvdQ | **41.00** | **0.00E+00** |
| PA3552 | ***arnB*** | ArnB [UDP-4-amino-4-deoxy-L-arabinose--oxoglutarate aminotransferase] [5, 6] | **39.39** | **1.66E-238** |
| PA3553 | ***arnC*** | ArnC [undecaprenyl-phosphate 4-deoxy-4-formamido-L-arabinose transferase] [7, 8] | **41.02** | **1.15E-65** |
| PA3554 | ***arnA*** | ArnA [bifunctional UDP-glucuronic acid decarboxylase/UDP-4- | **17.58** | **1.10E-139** |
| PA3555 | ***arnD*** | ArnD [4-deoxy-4-formamido-L-arabinose- | **11.69** | **2.29E-112** |
| PA3556 | ***arnT*** | Inner membrane L-Ara4N transferase ArnT [78] | **3.75** | **4.34E-11** |
| PA3558 | *arnF* | Hypothetical protein [7] | 1.82 | 0.012 |
| PA3559 | **-** | Nucleotide sugar dehydrogenase | **3.39** | **1.46E-61** |
| PA4003 | *pbpA* | Penicillin-binding protein 2 | -3.73 | 0.333 |
| PA4114 | **-** | Lysine decarboxylase | **3.32** | **1.17E-10** |
| PA4211 | ***phzB1*** | Phenazine biosynthesis protein | **-1235.99** | **0.00E+00** |
| PA4215 | ***phzF1*** | Phenazine biosynthesis protein | **-111.37** | **0.00E+00** |
| PA4418 | *ftsI* | Penicillin-binding protein 3 | -8.42 | 0.105 |
| PA4777 | *pmrB* | Two-component sensor PmrB [5] | -2.47 | 0.452 |
| PA5158 | *[opmG]* | Drug resistance OMP | 1.72 | 4.35E-04 |
| PA5159 | ***[emrA, pmrA]*** | Multidrug resistance protein | **2.78** | **3.81E-03** |
| PA5514 | **-** | Beta-lactamase [OXA-50-like] | **2.95** | **7.15E-04** |
| | | Aminoglycoside resistance | | |
| | | Antibiotic resistance and susceptibility | | |
| | | Novel efflux system biofilm-specific resistance | | |
| | | Penicillin-binding proteins | | |
| | | Synthesis of antibiotics | | |
| | | Response to antibiotics | | |

Expression of genes by *P. aeruginosa* PAO1 grown in WBHVs was compared with their expression when PAO1 was grown in LBB to an early log phase. Red shading indicates genes whose expression was downregulated; blue shading, genes whose expression was upregulated; bold text indicates *q* value ≤ 0.05 and fold change ≥ 2.00; regular text, fold change ≥ 2.00, *q* value > 0.05; yellow shading indicates genes composing operons. Gene numbers, names, and products were obtained from the *Pseudomonas* Genome DB (http://www.pseudomonas.com/). OMP, outer membrane protein. Gene numbers in shades of yellow indicate genes within operons.

**References**


1. Lee X, Fox A, Sufrin J, Henry H, Majcherczyk P, Haas D, et al. Identification of the biosynthetic gene cluster for the *Pseudomonas aeruginosa* antimetabolite L-2-amino-4-methoxy-trans-3-butenoic acid. J Bacteriol. 2010 Aug;192(16):4251-5. https://doi.org/10.1128/JB.00492-10. PubMed PMID: 20543073. Epub 2010/06/15.

2. Gilson L, Mahanty HK, Kolter R. Genetic analysis of an MDR-like export system: the secretion of colicin V. EMBO J. 1990 Dec;9(12):3875-84. PubMed PMID: 2249654. Epub 1990/12/01.

3. Macfarlane EL, Kwasnicka A, Ochs MM, Hancock RE. PhoP-PhoQ homologues in *Pseudomonas aeruginosa* regulate expression of the outer-membrane protein OprH and polymyxin B resistance. Mol Microbiol. 1999 Oct;34(2):305-16. https://doi.org/10.1046/j.1365-2958.1999.01600.x. PubMed PMID: 10564474. Epub 1999/11/17.

4. Zhang L, Mah TF. Involvement of a novel efflux system in biofilm-specific resistance to antibiotics. J Bacteriol. 2008 Jul;190(13):4447-52. https://doi.org/10.1128/JB.01655-07. PubMed PMID: 18469108. Epub 2008/05/13.

5. McPhee JB, Lewenza S, Hancock RE. Cationic antimicrobial peptides activate a two-component regulatory system, PmrA-PmrB, that regulates resistance to polymyxin B and cationic antimicrobial peptides in *Pseudomonas aeruginosa*. Mol Microbiol. 2003 Oct;50(1):205-17. https://doi.org/10.1046/j.1365-2958.2003.03673.x. PubMed PMID: 14507375. Epub 2003/09/26.

6. Segev-Zarko LA, Kapach G, Josten M, Klug YA, Sahl HG, Shai Y. Deficient lipid A remodeling by the *arnB* gene promotes biofilm formation in antimicrobial peptide susceptible *Pseudomonas aeruginosa*. Biochemistry. 2018 Apr 3;57(13):2024-34. https://doi.org/10.1021/acs.biochem.8b00149. PubMed PMID: 29518324. Epub 2018/03/09.

7. Gunn JS, Lim KB, Krueger J, Kim K, Guo L, Hackett M, et al. PmrA-PmrB-regulated genes necessary for 4-aminoarabinose lipid A modification and polymyxin resistance. Mol Microbiol. 1998 Mar;27(6):1171-82. https://doi.org/10.1046/j.1365-2958.1998.00757.x. PubMed PMID: 9570402. Epub 1998/05/07.

8. Breazeale SD, Ribeiro AA, McClerren AL, Raetz CR. A formyltransferase required for polymyxin resistance in *Escherichia coli* and the modification of lipid A with 4-amino-4-deoxy-L-arabinose. Identification and function oF UDP-4-deoxy-4-formamido-L-arabinose. J Biol Chem. 2005 Apr 8;280(14):14154-67. https://doi.org/10.1074/jbc.M414265200. PubMed PMID: 15695810. Epub 2005/02/08.
