# Supplementary material for: During bacteremia, Pseudomonas aeruginosa PAO1 adapts by altering the expression of numerous virulence genes including those involved in quorum sensing
Source: PLoS One. 2020 Oct 15;15(10):e0240351. doi: 10.1371/journal.pone.0240351 (PMC7561203; doi:10.1371/journal.pone.0240351)
Supplement: S5 Table — (PDF) [file pone.0240351.s012.pdf]

S5 Table. PAO1 genes for cell wall/LPS/O antigen synthesis upregulated or downregulated by growth in WBHVs compared to growth in LBB

Gene			Average	
Number	Name	Product / function [from orthologs]	Fold change	*q* Value
*PA2977**	***murB***	UDP-*N*-acetylenolpyruvoylglucosamine reductase*	**-4.06**	**0.001**
PA2978	***ptpA***	Phosphotyrosine protein phosphatase [cell wall function]	**-14.40**	**2.77E-03**
PA2979	*kdsB*	3-Deoxy-manno-octulosonate cytidylyltransferase [cell wall function]	-4.18	0.144
PA2981	*lpxK*	Tetraacyldisaccharide 4'-kinase [lipid A biosynthesis]	-2.91	0.423
PA3145	*wbpL*	Glycosyltransferase WbpL	-4.13	0.072
PA3146	***wbpK***	Probable NAD-dependent epimerase/dehydratase WbpK	**-11.20**	**1.19E-38**
PA3147	***wbpJ***	Probable glycosyltransferase WbpJ	**-9.20**	**1.57E-23**
PA3148	***wbpI***	UDP-*N*-acetylglucosamine 2-epimerase WbpI	**-11.42**	**1.07E-14**
PA3149	***wbpH***	Probable glycosyltransferase WbpH	**-15.53**	**3.28E-55**
PA3150	***wbpG***	LPS biosynthesis protein WbpG	**-6.10**	**3.23E-03**
PA3153	***wzx***	O-antigen translocase	**-8.28**	**7.79E-09**
PA3154	***wzy***	B-band O-antigen polymerase	**-32.35**	**6.34E-09**
PA3155	*wbpE*	UDP-2-acetamido-2-dideoxy-D-ribo-hex-3-uluronic acid transaminase WbpE	-5.64	0.588
PA3156	***wbpD***	UDP-2-acetamido-3-amino-2,3-dideoxy-D-glucuronic acid *N*-acetyltransferase WbpD	**-4.13**	**0.016**
PA3157	-	Acetyltransferase	-2.74	0.242
PA3160	*wzz*	O-antigen chain length regulator	-3.14	0.414
PA3552	***arnB/pmrH***	UDP-4-amino-4-deoxy-L-arabinose--oxoglutarate aminotransferase	**39.39**	**1.66E-238**
PA3553	***arnC/pmrF***	Protein ArnC, UDP phosphate 4-deoxy-4-formamido-L-arabinose	**41.02**	**1.15E-65**
PA3554	***arnA***	Bifunctional UDP-glucuronic acid decarboxylase/UDP-4-amino-4-deoxy-L-arabinose formyltransferase	**17.58**	**1.10E-139**
PA3555	***arnD/amrJ***	Protein ArnD, 4-deoxy-4-formamido-L-arabinose-phospho-UDP	**11.69**	**2.29E-112**
PA3556	***arnT/pqaB***	Inner membrane L-Ara4N transferase ArnT	**3.75**	**4.34E-11**
PA3559	**-**	Probable nucleotide sugar dehydrogenase	**3.39**	**1.46E-61**
PA3643	*lpxB*	Lipid-A-disaccharide synthase	-2.52	0.305
PA3644	*lpxA*	UDP-N-acetylglucosamine acyltransferase	-2.47	0.387
PA3645	***fabZ***	(3R)-hydroxymyristoyl-ACP dehydratase	**2.74**	**6.20E-03**
PA3988	*lptE*	LPS-assembly lipoprotein LptE	2.37	0.181
PA4407	*ftsZ*	Cell division protein FtsZ	-2.33	0.477
PA4408	***ftsA***	Cell division protein FtsA	**-4.17**	**6.54E-03**
PA4409	*ftsQ*	Cell division protein FtsQ	-6.36	0.269
PA4410	*ddlB*	D-alanine--D-alanine ligase	-7.31	0.094
PA4411	*murC*	UDP-*N*-acetylmuramate--L-alanine ligase	-2.25	0.749
PA4412	*murG*	UDP-muramoylpentapeptide beta-*N*-acetylglucosaminyltransferase	-3.24	0.313
PA4413	***ftsW***	Celldivision protein FtsW	**-6.84**	**1.89E-04**
PA4414	*murD*	UDP-*N*-acetylmuramoyl-L-alanyl-D-glutamate synthetase	-9.06	0.287
PA4415	*mraY*	Phospho-*N*-acetylmuramoyl-pentapeptide-transferase	-3.75	0.130
PA4416	*murF*	UDP-*N*-acetylmuramoyl-tripeptide--D-alanyl-D-alanine ligase	-2.70	0.228
PA4417	***murE***	UDP-*N*-acetylmuramoylalanyl-D-glutamate--2,6-diaminopimelate ligase	**-5.74**	**0.023**
PA4418	*ftsI*	Penicillin-binding protein 3	-8.42	0.126
PA4419	*ftsL*	Cell division protein FtsL	-12.02	0.105
PA4420	**-**	[S-Adenosyl-methyltransferase MraW]	**-4.46**	**1.87E-03**
PA4479	*mreD*	Rod shape-determining protein MreD	-14.64	0.29
PA4661	***pagL***	Lipid A 3-O-deacylase	**-19.99**	**7.03E-04**
PA4662	***murI***	Glutamate racemase	**-6.98**	**5.85E-03**
PA4996	*rfaE*	LPS biosynthesis protein RfaE	-2.14	0.264
PA5000	*wapR*	Alpha-1,3-rhamnosyltransferase WapR	-5.34	0.203
PA5001	*ssg*	Cell surface-sugar biosynthetic glycosyltransferase, Ssg	-3.24	0.298
PA5002	*dnpA*	De-*N*-acetylase involved in persistence, DnpA	-2.24	0.851
PA5003	-	Mig-14-like protein	-3.93	0.121
PA5004	*wapH*	Glycosyl transferase family protein WapH	-4.18	0.274
PA5005	-	Probable carbamoyl transferase	-2.25	0.155
PA5006	-	Putative kinase	-5.24	0.139
PA5007	*wapG*	WapG	-2.72	0.334
PA5008	*wapP*	WapP	-2.18	0.421
PA5009	*waaP*	Lipopolysaccharide kinase WaaP	-4.21	0.072
PA5010	*waaG*	UDP-glucose:(heptosyl) LPS alpha 1,3-glucosyltransferase WaaG	-3.24	0.133
PA5011	*waaC*	Heptosyltransferase I	-3.14	0.089
PA5012	*waaF*	Heptosyltransferase II	-2.00	0.360
PA5448	*wbpY*	Glycosyltransferase WbpY	-3.24	0.282
PA5452	*wbpW*	Phosphomannose isomerase/GDP-mannose WbpW	-3.86	0.277

*Values based on three replicates each from two HVs.

Expression of genes by *P. aeruginosa* PAO1 grown in WBHVs was compared with their expression when PAO1 was grown in LBB to an early log phase. Red shading indicates genes whose expression was downregulated; blue shading, genes whose expression was upregulated; bold text indicates *q* value ≤ 0.05 and fold change ≥ 2.00; regular text, fold change ≥ 2.00, *q* value > 0.05; yellow shading indicates genes composing operons. Gene numbers, names, and products were obtained from the *Pseudomonas* Genome DB (http://www.pseudomonas.com). LPS, lipopolysaccharide; UDP, uridine diphosphate; UTP, uridine triphosphate. Gene numbers in shades of yellow indicate genes within operons.
