# Supplementary material for: During bacteremia, Pseudomonas aeruginosa PAO1 adapts by altering the expression of numerous virulence genes including those involved in quorum sensing
Source: PLoS One. 2020 Oct 15;15(10):e0240351. doi: 10.1371/journal.pone.0240351 (PMC7561203; doi:10.1371/journal.pone.0240351)
Supplement: S6 Table — (PDF) [file pone.0240351.s013.pdf]

S6 Table. PAO1 genes of the type VI and type II secretion systems upregulated or downregulated by growth in WBHVs compared to growth in LBB.

Gene			Average	
Number	Name	Product / function [from orthologs]	Fold change	*q* Value
		Type VI Secretion System (T6SS) Genes [1]		
PA0070*	***tagQ1***	T6SS-associated lipoprotein TagQ1	**2.19**	**1.18E-19**
PA0071	***tagR1***	T6SS posttranslational regulatory protein TagR1	**3.41**	**2.82E-25**
PA0074	***ppkA***	T6SS serine/threonine protein kinase PpkA	**2.18**	**2.15E-06**
PA0075	***pppA***	T6SS negative regulation of secretion PppA protein	**3.56**	**1.25E-23**
PA0077	***icmF1***	T6SS protein IcmF1	**2.45**	**2.34E-08**
PA0078	***tssL1***	T6SS protein TssL1	**3.79**	**7.44E-14**
PA0079	***tssK1***	T6SS protein TssK1	**6.58**	**3.92E-85**
PA0080	***tssJ1***	T6SS protein TssJ1	**4.57**	**8.03E-34**
PA0081	***fha1***	T6SS forkhead-associated protein 1	**7.02**	**6.36E-86**
PA0082	***tssA1***	T6SS protein TssA1	**16.60**	**0.00E+00**
PA0083	***tssB1***	T6SS protein TssB1	**16.68**	**0.00E+00**
PA0084	***tssC1***	T6SS protein TssC1	**7.70**	**6.55E-143**
PA0086	***tagJ1***	T6SS protein TagJ1	**8.73**	**3.71E-111**
PA0087	***tssE1***	T6SS protein TssE1	**15.57**	**1.18E-192**
PA0088	***tssF1***	T6SS protein TssF1	**5.12**	**1.13E-45**
PA0089	***tssG1***	T6SS protein TssG1	**3.42**	**9.41E-10**
PA0090	***clpV1***	T6SS ClpV1 protein	**2.89**	**3.52E-24**
PA0091	*vgrG1a*	T6SS spike VgrG1a protein	1.90	5.77E-09
PA0093	***tse6***	T6SS effector protein 6	**4.42**	**5.82E-38**
PA0094	***eagT6***	T6SS protein EagT6	**22.04**	**7.11E-168**
PA0095	***vgrG1b***	T6SS spike VgrG1b protein	**3.44**	**3.64E-52**
PA0096	**-**	Hypothetical protein, probably related to T6SS	**3.34**	**0.0004**
PA0099	***tse7***	T6SS effector protein 7	**2.00**	**9.49E-08**
PA0262	***vgrG2b***	T6SS spike protein VgrG2b	**2.74**	**8.20E-10**
PA0263	***hcpC***	T6SS hemolysin co-regulated effector protein HcpC	**13.56**	**5.27E-15**
PA0823	-	Hypothetical protein, probably related to T6SS	-3.65	0.527
PA1508	*PAAR3*	T6SS PAAR protein	-2.66	0.4153
PA1511	***vgrG2a***	T6SS spike protein VgrG2a	**2.02**	**3.13E-05**
PA1512	***hcpA***	T6SS hemolysin co-regulated effector protein HcpA	**8.05**	**7.69E-09**
PA1656*	*hsiA2*	T6SS protein HsiA2	-3.29	0.354
PA1660	*hsiG2*	T6SS protein HsiG2	-3.95	0.307
PA1661	*hsiH2*	T6SS protein HsiH2	-3.02	0.282
PA1662	***clpV2***	T6SS ClpA/B-type protease	**-4.24**	**0.004**
PA1663	***sfa2***	T6SS transcriptional regulator	**-5.18**	**0.003**
PA1664	***orfX***	T6SS secretion protein	**-6.41**	**4.61E-06**
PA1665	*fha2*	T6SS forkhead-associated protein 2 (Fha2)	-2.48	0.062
PA1666	*lip2*	T6SS protein Lip2	-4.51	0.203
PA1667	*hsiJ2*	T6SS protein HsiJ2	-3.16	0.156
PA1668	*dotU2*	T6SS protein DotU2	-1.67	0.255
PA1669	*icmF2*	T6SS protein IcmF2	-1.70	0.198
PA1670	***stp1***	T6SS serine/threonine phosphoprotein phosphatase Stp1	**-25.76**	**4.55E-29**
PA1671	***stk1***	T6SS serine-threonine kinase Stk1	**-11.48**	**0.008**
PA1844	***tse1***	T6SS effector protein Tse1	**5.43**	**4.27E-10**
PA1845	***tsi1***	T6SS immunity protein Tsi1	**36.61**	**1.36E-146**
PA2360*	***hsiA3***	T6SS protein HsiA3	**-6.99**	**0.024**
PA2361	***icmF3***	T6SS protein IcmF3	**-8.80**	**0.006**
PA2362	***dotU3***	T6SS protein DotU3	**-16.35**	**4.42E-06**

PA2363	***hsiJ3***	T6SS protein HsiJ3	**-14.85**	**1.80E-08**
PA2364	***lip3***	T6SS protein Lip3	**-6.89**	**0.020**
PA2366	***hsiC3***	T6SS protein HsiC3	**-4.05**	**0.0001**
PA2367	***hcp3***	T6SS hemolysin-coregulated secreted protein Hcp3	**-8.88**	**0.0002**
PA2368	*hsiF3*	T6SS protein HsiF3	-30.22	0.056
PA2369	*hsiG3*	T6SS protein HsiG3	-5.85	0.333
PA2370	***hsiH3***	T6SS protein HsiH3	**-16.79**	**0.021**
PA2371	***clpVe3***	T6SS ClpA/B-type protease	**-11.65**	**2.33E-16**
PA2373	***vgrG3***	T6SS spike protein VgrG3	**-13.42**	**5.11E-35**
PA2374	***tseF***	T6SS effector TseF	**-7.18**	**1.70E-06**
PA2684	***tse5***	T6SS effector proteinTse5	**2.39**	**9.77E-11**
PA2685	***vgrG4***	T6SS VgrG4 spike protein	**3.00**	**7.75E-18**
PA2702	***tse2***	T6SS effector protein Tse2	**9.48**	**5.98E-16**
PA2703	*tsi2*	T6SS immunity protein Tsi2	1.98	0.013
PA2774	*tse4*	T6SS effector protein Tse4	-2.84	0.230
PA3291[†]	*tli1 [tli1a]*	T6SS lipase immunity protein Tli1a	-3.60	0.133
PA3292[†]	*[tli1b]*	T6SS lipase immunity protein Tli1b	-2.67	0.734
PA3294	*vgrG4a*	T6SS spike protein VgrG4a	1.91	0.004
PA3484	***tse3***	T6SS effector protein Tse3	**11.82**	**9.48E-59**
PA3485	***tsi3***	T6SS immunity protein Tsi3	**4.16**	**2.61E-06**
PA3486	*vgrG4b*	T6SS spike protein VgrG4b	1.88	0.001
PA3906	-	T6SS co-chaperone co-TecT	-7.86	0.189
PA3907	*tseT*	T6SS TOX-Rease-5 domain-containing effector, TseT	-4.25	0.276
PA3908	*tsiT*	T6SS immunity protein TsiT	-9.38	0.060
PA5087[†]	*tli5b2*	T6SS lipase immunity protein Tli5b2	**-5.61**	**0.010**
PA5088	*tli5b3*	T6SS lipase immunity protein Tli5b3	-3.89	0.442
PA5090	***vgrG5***	T6SS spike protein VgrG5	**2.42**	**0.001**
PA5266	*vgrG6*	T6SS spike protein VgrG6	2.47	0.233
PA5267	***hcpB***	T6SS hemolysin co-regulated effector protein HcpB	**26.22**	**7.79E-29**

*Genes PA0070-PA0099 constitute the T6SS Hcp secretion island I (HSI-I); PA1656-PA1671, HSI-II; PA2360-PA2374, HSI-III; in boxes

[†]Values based on three replicates each from two HVs.

Type II/Sec/Tat Secretion System Genes				
PA3095	*xcpZ*	General T2SS pathway protein M	-3.20	0.162
PA3096	***xcpY***	General T2SS pathway protein L	**-10.66**	**3.57E-03**
PA3097	***xcpX***	General T2SS pathway protein K	**-9.75**	**2.69E-04**
PA3098	***xcpW***	General T2SS pathway protein J	**-17.69**	**1.78E-13**
*PA3099**	***xcpV***	General T2SS pathway protein I	**-8.94**	**3.02E-09**
PA3100	***xcpU***	General T2SS pathway outer membrane protein H	**-4.61**	**0.043**
PA3101	*xcpT*	General T2SS pathway protein G	-2.09	0.336
PA3102	*xcpS*	General T2SS pathway protein F	-3.49	0.107
PA3820	*secF*	Sec pathway secretion protein SecF	-7.56	0.098
PA3821	*secD*	Sec pathway secretion protein SecD	-2.09	0.328
PA4243	*secY*	Sec pathway secretion protein SecY	-15.34	0.118
PA4276	*secE*	Sec pathway secretion protein SecE	-2.91	0.224
PA5068	*tatA*	Tat pathway twin-arginine translocation protein TatA	1.71	9.24E-07
PA5069	***tatB***	Tat pathwaySec-independent translocase TatB	**2.84**	**1.25E-18**
PA5070	***tatC***	Tat pathwaySec-independent transporter TatC	**2.20**	**8.70E-05**

*Values based on three replicates each from two HVs.

Expression of genes by *P. aeruginosa* PAO1 grown in WBHVs was compared with their expression when PAO1 was grown in LBB to an early log phase. Red shading indicates genes whose expression was downregulated; blue shading, genes whose expression was upregulated; bold text indicates *q* value ≤ 0.05 and fold change ≥ 2.00; regular text, fold change ≥ 2.00, *q* value > 0.05; yellow shading indicates genes composing operons. Gene numbers, names, and products were obtained from the *Pseudomonas* Genome DB (http://www.pseudomonas.com/).

Reference

1. Wood TE, Howard SA, Wettstadt S, Filloux A. PAAR proteins act as the 'sorting hat' of the type VI secretion system. Microbiology. 2019 Nov;165(11):1203-18. https://doi.org/10.1099/mic.0.000842. PubMed PMID: 31380737. Epub 2019/08/06.
