# Supplementary material for: During bacteremia, Pseudomonas aeruginosa PAO1 adapts by altering the expression of numerous virulence genes including those involved in quorum sensing
Source: PLoS One. 2020 Oct 15;15(10):e0240351. doi: 10.1371/journal.pone.0240351 (PMC7561203; doi:10.1371/journal.pone.0240351)
Supplement: S7 Table — (PDF) [file pone.0240351.s014.pdf]

S7 Table. PAO1 genes related to motility, chemotaxis, and adhesion that were upregulated or downregulated by growth in WBHVs compared to growth in LBB.

Gene Number	Gene Name	Product / function [from orthologs]	Average Fold change	*q* Value
		Swarming motility		
**PA5531**	***tonB1***	TonB1	**2.57**	**3.46E-07**
PA2780	*bswR*	Bacterial swarming regulator, BswR	-4.67	0.270
		Twitching motility and adhesion		
PA0395	***pilT***	Twitching motility protein PilT	**3.57**	**5.11E-07**
PA0409	***pilH***	Twitching motility protein PilH	**3.56**	**6.35E-27**
PA4526	***pilB***	Type 4 fimbrial biogenesis protein PilB	**3.12**	**1.29E-50**
PA4293	*pprA*	Two-component sensor PprA	**-18.33**	**6.55E-16**
*PA4294**	-	[Putative pilus assembly]*	**-40.95**	**1.21E-18**
PA4296	*pprB*	Two-component response regulator, PprB	-3.34	0.164
PA4297	*tadG*	TadG [type IVb pili] [1]	**-7.91**	**2.17E-13**
PA4299	*tadD*	TadD [1]	**-35.21**	**5.77E-72**
*PA4300**	*tadC*	TadC [1]*	**-18.50**	**6.26E-87**
PA4302	*tadA*	ATPase TadA [1]	**-55.20**	**3.42E-188**
PA4303	*tadZ*	TadZ [1]	**-42.52**	**2.50E-49**
PA4304	*rcpA*	RcpA [1]	**-23.69**	**1.63E-35**
PA4306	*flp*	Type IVb pilin Flp [1]	**-133.58**	**0.00E+00**
PA0992	*cupC1*	Fimbrial subunit CupC1	-3.92	0.16
PA0994	*cupC3*	Usher CupC3	-3.31	0.167
PA2128	*cupA3*	Usher CupA3	-2.50	0.340
PA4082	*cupB5*	Adhesive protein CupB5	-3.67	0.334
*PA4084**	***cupB3***	Usher CupB3*	**-3.85**	**0.031**
PA4086	*cupB1*	Probable fimbrial subunit CupB1	-2.88	0.155
PA4648	*cupE1*	Pilin subunit CupE1	-4.83	0.321
PA4649	*cupE2*	Pilin subunit CupE2	-7.08	0.324
PA4650	*cupE3*	Pilin subunit CupE3	-5.91	0.213
PA4651	***cupE4***	Pilin assembly chaperone CupE4	**-11.31**	**3.73E-09**
*PA4652**	***cupE5***	Fimbrial usher protein CupE5*	**-6.00**	**0.032**
PA4653	***cupE6***	Adhesin-like protein CupE6	**-12.21**	**0.026**
PA4781	**-**	Cyclic di-GMP phosphodiesterase [2]	**-6.92**	**1.79E-11**
PA5498	***znuA***	Adhesin	**4.22**	**1.24E-49**
		Flagellum		
PA1080	***flgE***	Flagellar hook protein FlgE	**5.62**	**1.01E-54**
PA1081	***flgF***	Flagellar basal body rod protein FlgF	**2.24**	**9.05E-14**
PA1082	***flgG***	Flagellar basal body rod protein FlgG	**2.47**	**2.91E-29**
PA1084	*flgI*	Flagellar basal body P-ring biosynthesis protein FlgI	-2.82	0.193
PA1085	*flgJ*	Flagellar rod assembly protein/muramidase FlgJ	-2.03	0.205
PA1091	***fgtA***	Flagellar glycosyl transferase, FgtA	**-6.46**	**6.37E-03**
**PA1094**	***fliD***	Flagellar capping protein FliD	**2.54**	**5.32E-21**
**PA1104**	***fliI***	Flagellum-specific ATP synthase	**-10.05**	**0.001**
PA1105	*fliJ*	Flagellar biosynthesis chaperone	**-16.40**	0.091
PA1444	*fliN*	Flagellar motor switch protein	-2.27	0.512
PA1445	*fliO*	Flagellar protein FliO	-2.00	0.395
PA1446	*fliP*	Flagellar biosynthesis protein FliP	-3.08	0.319
PA1447	*fliQ*	Flagellar biosynthesis protein FliQ	-3.67	0.075
PA1448	*fliR*	Flagellar biosynthesis protein FliR	-2.93	0.319
PA1471	*motD*	Flagellar motor protein MotD	-2.16	0.31

		Chemotaxis		
PA0173	-	Probable methylesterase	-4.31	0.273
**PA0174**	**-**	Probable chemotaxis protein	**-7.85**	**0.008**
PA0175	*cheR2*	Probable chemotaxis protein methyltransferase	-8.26	0.123
PA0176	*aer2*	Aerotaxis transducer Aer2	-4.76	0.060
**PA0177**	**-**	Probable purine-binding chemotaxis protein	**-16.45**	**6.37E-15**
**PA0178**	**-**	Probable two-component sensor	**-22.92**	**1.38E-68**
**PA0179**	**-**	Probable two-component response regulator	**-12.52**	**6.69E-29**
PA0412	*pilK*	Methyltransferase PilK	-1.72	0.28
PA0413	*chpA*	Chemotactic signal transduction system protein	-2.16	0.28
PA0415	*chpC*	Chemotaxis protein	-2.75	0.29
**PA1251**	**-**	Probable chemotaxis transducer	**-6.38**	**1.29E-05**
**PA1423**	***bdlA***	BdlA [probable chemotaxis transducer]	**5.14**	**1.38E-41**
PA1456	*cheY*	Chemotaxis protein CheY	-2.28	0.39
**PA1608**	**-**	Probable chemotaxis transducer [methyl-accepting chemotaxis protein]	**5.26**	**1.80E-24**
**PA1646**	**-**	Probable chemotaxis transducer	**-7.55**	**1.58E-03**
**PA2652**	**-**	Methyl-accepting chemotaxis protein	**2.48**	**1.33E-07**
**PA2654**	***tlpQ***	TlpQ, probable chemotaxis transducer	**3.12**	**4.71E-53**
PA2656	*bqsS*	Two-component sensor BqsS	-11.81	0.067
PA3703	*wspF*	Probable chemotaxis-specific methylesterase	-4.94	0.334
**PA3704**	***wspE***	Probable chemotaxis sensor/effector fusion protein	**-10.33**	**4.47E-06**
**PA3706**	***wspC***	Probable protein methyltransferase	**-6.04**	**8.84E-03**
**PA3707**	***wspB***	Hypothetical protein [probable chemotaxis protein]	**-9.95**	**0.010**
**PA3708**	*wspA*	Chemotaxis transducer	-2.35	0.318
**PA4307**	***pctC***	Chemotactic transducer PctC	**-4.84**	**0.034**
**PA4309**	***pctA***	Chemotactic transducer PctA	**3.21**	**9.79E-40**
**PA4310**	***pctB***	Chemotactic transducer PctB	**2.45**	**2.70E-13**
**PA4633**	**-**	Probable chemotaxis transducer	**2.36**	**2.28E-24**
**PA4915**	**-**	Chemotaxis transducer	**3.53**	**1.20E-04**

*Values based on three replicates each from two HVs.

Expression of genes by *P. aeruginosa* PAO1 grown in WBHVs was compared with their expression when PAO1 was grown in LBB to an early log phase. Red shading indicates genes whose expression was downregulated; blue shading, genes whose expression was upregulated; bold text indicates *q* value ≤ 0.05 and fold change ≥ 2.00; regular text, fold change ≥ 2.00, *q* value > 0.05; yellow shading indicates genes composing operons. Gene numbers, names, and products were obtained from the *Pseudomonas* Genome DB (http://www.pseudomonas.com/).

References

1. de Bentzmann S, Aurouze M, Ball G, Filloux A. FppA, a novel *Pseudomonas aeruginosa* prepilin peptidase involved in assembly of type IVb pili. J Bacteriol. 2006 Jul;188(13):4851-60. https://doi.org/10.1128/JB.00345-06. PubMed PMID: 16788194. Epub 2006/06/22.

2. Ryan RP, Lucey J, O'Donovan K, McCarthy Y, Yang L, Tolker-Nielsen T, et al. HD-GYP domain proteins regulate biofilm formation and virulence in *Pseudomonas aeruginosa*. Environ Microbiol. 2009 May;11(5):1126-36. https://doi.org/10.1111/j.1462-2920.2008.01842.x. PubMed PMID: 19170727. Epub 2009/01/28.
