# Supplementary material for: During bacteremia, Pseudomonas aeruginosa PAO1 adapts by altering the expression of numerous virulence genes including those involved in quorum sensing
Source: PLoS One. 2020 Oct 15;15(10):e0240351. doi: 10.1371/journal.pone.0240351 (PMC7561203; doi:10.1371/journal.pone.0240351)
Supplement: S8 Table — (PDF) [file pone.0240351.s015.pdf]

S8 Table. PAO1 *mex* genes related to multidrug efflux were predominantly downregulated by growth in WBHVs compared to growth in LBB.

**Gene**			**Average**	
**Number**	**Name**	**Product Function**	**Fold change**	***qValue***
PA0424	*mexR*	Multidrug resistance operon repressor MexR	-2.02	0.145
PA0425	*mexA*	RND multidrug efflux MFP MexA	-1.81	0.409
PA0426	***mexB***	RND multidrug efflux transporter MexB	**-3.53**	**0.037**
PA0427	*oprM*	Multidrug ABC transporter	-1.88	7.85E-04
PA1436	*[mexN]*	Probable RND efflux transporter MexN	-4.67	0.110
PA2019	***mexX***	RND multidrug efflux MFP MexX precursor	**5.67**	**2.17E-07**
PA2020	*mexZ*	Transcriptional regulator MexZ, negative	-2.02	0.524
PA2494	*mexF*	RND multidrug efflux transporter MexF	-2.99	0.512
PA2495	*oprN*	Multidrug efflux outer membrane protein OprN	-3.10	0.301
PA3521	*opmE*	Outer membrane efflux protein	-4.67	0.334
PA3522	***mexQ***	RND efflux transporter	**-12.78**	**9.84E-06**
PA3523	***mexP***	RND efflux MFP	**-28.89**	**9.15E-50**
PA3574	***nalD***	NalD protein (repressor of *mexAB-oprM*)	**-24.76**	**4.08E-200**
PA3676	***mexK***	RND efflux transporter	**-4.18**	**0.013**
PA3677	*mexJ*	RND efflux MFP	-2.38	0.427
PA3678	*mexL*	Transcriptional regulator	-2.54	0.498
PA4205	***mexG***	Hypothetical protein, related to QS?	**-23.48**	**1.83E-73**
PA4206	***mexH***	RND efflux MFP	**-32.38**	**1.93E-110**
PA4207	***mexI***	RND efflux transporter	**-22.68**	**9.05E-122**
*PA4208**	***opmD***	Probable outer membrane protein precursor	**-74.17**	**0.00E+00**
PA4375	*mexW*	RND multidrug efflux transporter MexW	-2.44	0.316
PA4596	*esrC*	Transcriptional regulator, positive?	-4.98	0.071
PA4597	***oprJ***	Multidrug efflux outer membrane protein OprJ	**-8.82**	**0.012**
PA4598	*mexD*	RND multidrug efflux transporter MexD	-5.62	0.070
PA4599	*mexC*	RND multidrug efflux MFP MexC	-8.50	0.333
PA2525	***ompB***	Efflux transmembrane transporter activity [1]	**-5.17**	**0.001**
PA2526	*muxC*	RND efflux transporter [1]	-5.78	0.225
PA2527	*muxB*	RND efflux transporter [1]	-2.86	0.546

Expression of genes by *P. aeruginosa* PAO1 grown in WBHVs was compared with their expression when PAO1 was grown in LBB to an early log phase. Red shading indicates genes whose expression was downregulated; blue shading, genes whose expression was upregulated; bold text indicates *q* value ≤ 0.05 and fold change ≥ 2.00; regular text, fold change ≥ 2.00, *q* value > 0.05; yellow shading indicates genes composing operons. Gene numbers, names, and products were obtained from the *Pseudomonas* Genome DB (http://www.pseudomonas.com/). RND, resistance-nodulation-cell division; MFP, membrane fusion protein

*Values based on three replicates each from two HVs.

Reference

1. Mima T, Kohira N, Li Y, Sekiya H, Ogawa W, Kuroda T, et al. Gene cloning and characteristics of the RND-type multidrug efflux pump MuxABC-OpmB possessing two RND components in *Pseudomonas aeruginosa*. Microbiology. 2009 Nov;155(Pt 11):3509-17. https://doi.org/10.1099/mic.0.031260-0. PubMed PMID: 19713238. Epub 2009/08/29.
